# The BTB-zinc Finger Transcription Factor Abrupt Acts as an Epithelial Oncogene in *Drosophila melanogaster* through Maintaining a Progenitor-like Cell State

**DOI:** 10.1371/journal.pgen.1003627

**Published:** 2013-07-18

**Authors:** Nezaket Turkel, Virender K. Sahota, Jessica E. Bolden, Karen R. Goulding, Karen Doggett, Lee F. Willoughby, Enrique Blanco, Enrique Martin-Blanco, Montserrat Corominas, Jason Ellul, Toshiro Aigaki, Helena E. Richardson, Anthony M. Brumby

**Affiliations:** 1 Cell Cycle and Development Laboratory, Peter MacCallum Cancer Centre, East Melbourne, Victoria, Australia; 2 Department of Anatomy and Cell Biology, The University of Melbourne, Melbourne, Victoria, Australia; 3 Departament de Genètica i Institut de Biomedicina (IBUB), Universitat de Barcelona, Barcelona, Spain; 4 Instituto de Biología Molecular de Barcelona (CSIC), Parc Cientific de Barcelona, Barcelona, Spain; 5 Bioinformatics Core Facility, Peter MacCallum Cancer Centre, East Melbourne, Victoria, Australia; 6 Department of Biological Sciences, Tokyo Metropolitan University, Hachioji, Tokyo, Japan; 7 Sir Peter MacCallum Department of Oncology, University of Melbourne, Melbourne, Victoria, Australia; 8 Department of Biochemistry and Molecular Biology, University of Melbourne, Melbourne, Victoria, Australia; 9 Department of Genetics, The University of Melbourne, Melbourne, Victoria, Australia; University of Washington, United States of America

## Abstract

The capacity of tumour cells to maintain continual overgrowth potential has been linked to the commandeering of normal self-renewal pathways. Using an epithelial cancer model in *Drosophila melanogaster*, we carried out an overexpression screen for oncogenes capable of cooperating with the loss of the epithelial apico-basal cell polarity regulator, *scribbled* (*scrib*), and identified the cell fate regulator, Abrupt, a BTB-zinc finger protein. Abrupt overexpression alone is insufficient to transform cells, but in cooperation with *scrib* loss of function, Abrupt promotes the formation of massive tumours in the eye/antennal disc. The steroid hormone receptor coactivator, Taiman (a homologue of SRC3/AIB1), is known to associate with Abrupt, and Taiman overexpression also drives tumour formation in cooperation with the loss of Scrib. Expression arrays and ChIP-Seq indicates that Abrupt overexpression represses a large number of genes, including steroid hormone-response genes and multiple cell fate regulators, thereby maintaining cells within an epithelial progenitor-like state. The progenitor-like state is characterised by the failure to express the conserved Eyes absent/Dachshund regulatory complex in the eye disc, and in the antennal disc by the failure to express cell fate regulators that define the temporal elaboration of the appendage along the proximo-distal axis downstream of Distalless. Loss of *scrib* promotes cooperation with Abrupt through impaired Hippo signalling, which is required and sufficient for cooperative overgrowth with Abrupt, and JNK (Jun kinase) signalling, which is required for tumour cell migration/invasion but not overgrowth. These results thus identify a novel cooperating oncogene, identify mammalian family members of which are also known oncogenes, and demonstrate that epithelial tumours in *Drosophila* can be characterised by the maintenance of a progenitor-like state.

## Author Summary

Cancer is a multigenic process, involving cooperative interactions between oncogenes or tumour suppressors. In this study, in a genetic screen in the vinegar fly, *Drosophila melanogaster*, for genes that cooperate with a mutation in the cell polarity (shape) regulator, *scribbled (scrib)*, we identify a novel cooperative oncogene, *abrupt*. Expression of *abrupt* in *scrib* mutant tissue in the developing eye/antennal epithelium results in overgrown invasive tumours. *abrupt* encodes a BTB-zinc finger transcription factor, which has homology to several cancer-causing proteins in humans, such as BCL6. Analysis of the Abrupt targets and misexpressed genes in *abrupt* expressing-tissue and *abrupt*-expressing *scrib* mutant tumours, revealed cell fate regulators as a major class of targets. Thus, our results reveal that deregulation of multiple cell fate factors by Abrupt expression in the context of polarity disruption is associated with a progenitor-like cell state and the formation of overgrown invasive tumours. Our findings suggest that defective polarity may also be a critical factor in BTB-zinc finger-driven human cancers, and warrants further investigation into this issue.

## Introduction

Cancer cells with significant tumour-propagating potential are increasingly referred to as cancer stem cells. Whilst this refers to the potential of these cells to regenerate the tumour in both *in vivo* and *in vitro* assays, it also alludes to the possibility that these cells may have either hijacked self-renewal programmes involved in normal stem cell maintenance, or that they are in fact directly derived from stem or progenitor-like cells. Consistent with either of these possibilities, profiles of tumour cells show increased expression of stem cell factors and associations with progenitor-like cell states [1], [2].

In *Drosophila melanogaster*, tumours have long been known to be associated with the retention of stem cell states. Germ line tumours show continual overgrowth of progenitor cells that fail to initiate differentiation, and neuroblast-derived brain tumours are associated with defects in neuroblast (neural stem cell) divisions and an expansion of neuroblast numbers [reviewed in 3]. Furthermore, the overgrowth associated with *l(3) malignant brain tumour* mutants has been shown to depend upon the acquisition of a stem cell state associated with the germline [4]. Impaired differentiation has also been considered to be a hallmark of *Drosophila* epithelial tumours [5], although how differentiation is perturbed and what role this plays in maintaining tumour overgrowth is not yet known. Indeed the epithelial tissues of the imaginal discs are not thought to contain stem cells. Instead it appears that cells become progressively restricted in their developmental potential as patterning mechanisms drive greater elaboration and cell fate commitments across the epithelial field. The sequential nature of these elaborations means that epithelial progenitor-like states are generally associated with earlier developmental times and are not necessarily associated with spatially defined regions of the developing tissue. In the antennal disc, the early progenitor state is yet to be clearly characterised, although the early division between the more distally destined cells that express the homeodomain protein Distal-less (Dll) and the more proximal cells expressing the MEIS family transcription factor, Homothorax (Hth), is one of the earliest cell fate divisions to have been described within the developing appendage [reviewed in 6]. Downstream targets of these genes, including *atonal* (*ato*), *dachshund* (*dac*), *distal antenna* (*dan*), and *bric-a-brac 2* (*bab2*), are subsequently expressed, and gradually define further cell fate divisions along the proximo-distal axis of the appendage [7]–[9]. In the eye disc, the progenitor state has been more fully defined and is thought to be characterised by the expression of Hth, which cooperates with Yorkie (Yki, or YAP in mammals), the transcriptional coactivator of the Hippo tissue growth control pathway [reviewed in 10], to maintain cells within a proliferative state [11]. The downregulation of Hth coincides with the progressive upregulation of cell fate markers such as *dac*, *eyes absent* (*eya*), *dan*, *ato* and *embryonic lethal abnormal vision* (*elav*), that define further differentiation [reviewed in 6]. What role, if any, these sequential cell fate restrictions play in mediating the overgrowth of eye and antennal disc tumours has not yet been investigated.

Epithelial tumours can be induced in the eye/antennal disc by using a clonal system to combine loss of the cell polarity regulator and tumour suppressor *scribbled* (*scrib*) with oncogenic Ras or Notch (N) signalling. Whilst neither genetic alteration is sufficient to transform cells, in combination they cooperate to drive the formation of invasive tumours that outcompete the surrounding untransformed tissue and massively overgrow [12]. In an overexpression screen, to identify novel cooperating oncogenes that function like oncogenic Ras or Notch, we isolated the BTB-zinc finger (BTB-ZF) domain protein Abrupt (Ab). Expression arrays and ChIP-Seq analysis of Ab binding regions and immunohistochemical analysis of the tumours indicates that Ab promotes the retention of a progenitor-like cell state in *scrib* mutant cells by blocking the expression of *dac*, *eya*, *dan*, *ato* and *elav* in the eye disc, and prevents the temporal elaboration of cell fate domains, defined by *dac*, *cut* (*ct*), *senseless* (*sens*), *dan*, *bab2* and *ato* expression, along the proximo-distal axis in the antennal disc. The Hippo tissue growth control pathway transcriptional coactivator, Yki, is both required to promote tumour overgrowth, and sufficient to cooperate with Ab and maintain cells within the progenitor-like state.

## Results

### A screen for cooperating oncogenes in *Drosophila*

We have previously shown how loss of the epithelial cell polarity regulator and tumour suppressor *scrib* cooperates with oncogenic Ras (*Ras^V12^*/*Ras^ACT^*) or Notch (*Notch^intra^*/*Notch^ACT^*) signalling to promote the formation of invasive tumours [12]. To identify novel oncogenes in *Drosophila* we carried out an overexpression screen to identify additional genes that can cooperate with the loss of *scrib* to promote tumour overgrowth. This was done using a bank of *Gene Search* (*GS*) *P* element lines [13], which contain *UAS* sites to ectopically express the flanking genes. By combining this with *GAL4*-driven expression, we screened independent *GS* line insertions on the second chromosome for their ability to promote neoplastic overgrowth when combined with the loss of *scrib* in eye disc clones. Normally the generation of *scrib* mutant clones in the eye/antennal disc produces adult flies with mildly reduced and necrotic eyes due to Jun kinase (JNK)-mediated death of the mutant tissue [12]. We therefore aimed to identify genes that could either cause pupal lethality or, most importantly, act like activated alleles of either Ras or Notch to block larval pupariation and cause massive tumour overgrowth.

**Figure 1:**
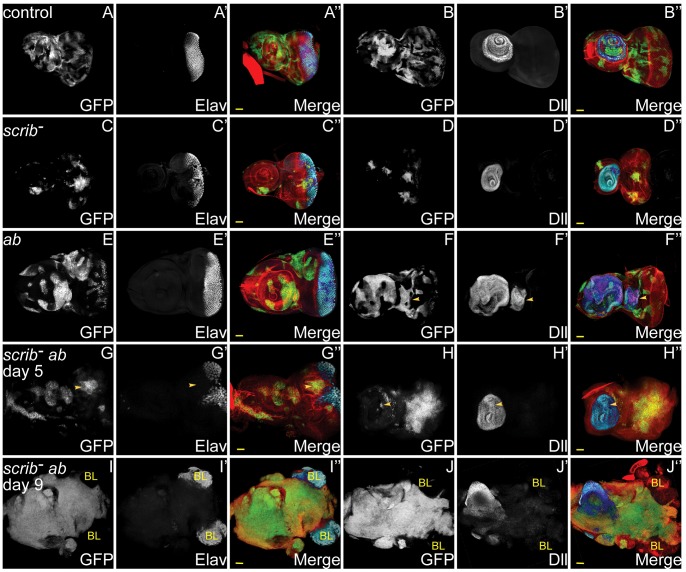
*ab* overexpression in *scrib* mutant clones promotes neoplastic overgrowth of eye/antennal epithelial tissue throughout an extended larval stage. Mosaic eye/antennal discs (anterior to the left in this and all subsequent figures) generated with *ey-FLP* and taken from larvae 5 days (A–H) or 9 days (I,J) AEL. Clones are positively marked by GFP (white, or green in merges). Tissue morphology is shown by F-actin (red in merges), and cell fate by Elav and Dll (white, or blue in merges – dark blue when overlaid with GFP). Brain lobes in I,J are marked by BL. GFP (panels A–J), Elav (panels A′,C′,E′,G′,I′), Dll (panel B′,D′,F′H′,J′) and merges (panels A″–J″). (A,B) Control mosaic eye/antennal discs show the normal pattern of Elav expression in developing photoreceptor cells, and Dll expression within the antenna. (C,D) *scrib^1^* cells still express Elav and Dll, although the normal pattern of Elav-expressing photoreceptor cells is disrupted by alterations in tissue morphology. (E,F) *ab* overexpressing clones still express Elav and Dll, but are often larger than control clones within the antennal region, and in some discs ectopic domains of Dll expression are observed (F, arrowhead). (G,H) *scrib^1^*+*ab* clones are larger than *scrib^1^* clones, and do not express Elav (G, arrowhead), although Dll expression is maintained (H, arrowhead). (I,J) *scrib^1^*+*ab* clones at day 9 are massively overgrown and the two eye/antennal discs fuse with each other and with the Elav-expressing brain lobes (I), whilst the Dll-expressing domain in the antennal disc is maintained (J). Yellow scale bar = 50 µm.

From screening ∼2000 *GS* lines, we identified over 50 that caused increased organism lethality when expressed in *scrib* mutant clones (**Table 1**). As the insertion point and expressed genes have been mapped for all *GS* lines, it was possible to determine that this corresponded to 10 different genes. Using independent transgenes we were able to confirm that overexpression of 6 of them (*abrupt* (*ab*), *dorsal* (*dl*), *escargot* (*esg*), *numb*, *charlatan* (*chn*) and *apontic* (*apt*) reproduced the lethality of the *GS* line. For the remaining 4 genes (*kismet* (*kis*), *anachronism* (*ana*), *CG3363* and *CG10543*), although we identified multiple independent *GS* lines for each, independent transgenes were not available at the time to confirm the interaction. For the confirmed interactors, we examined larval eye/antennal discs to determine the extent of clonal overgrowth induced by the transgene alone compared to the amount of overgrowth when combined with the loss of *scrib* (**Figure 1** and **Figure S1**). Some of the interactors (*numb* and *apt*) promoted very little consistent overgrowth phenotypes despite causing organism lethality at the pupal stage of development, whilst *chn* produced mild overgrowth, and both *dl* and *esg* were striking for producing very large antennal overgrowths before the larvae pupated at day 5/6 after egg laying (AEL). However, of the 6 confirmed genes, the strongest interactor was *ab*.

**Table 1:** Identified *scrib^−^* cooperating oncogenes.

GS lines	Overexpressed gene, position of GS insertion	Validating transgene	Validated/Candidate gene function	Closest human homologue
49 (GSV1) 81 (GSV1)	CG4807(*ab*)-RA, +10847 CG4807(*ab*)-RA, +10854	*UAS-ab*	BTB-C2H2 zinc finger transcription factor	ZFP161
5075 (GSV2) 5251 (GSV2)	CG6667(*dl*)-RA, +505 CG6667(*dl*)-RA, +621	*UAS-dl*	NF-kB/Rel family transcription factor	REL
2077 (GSV1) 5022 (GSV2) 9490 (GSV6) 11431 (GSV6) 11506 (GSV6) 11550 (GSV6) 13437 (GSV6) 14412 (GSV6) 14394 (GSV6)	CG3758(*esg*)-RA, −98 CG3758(*esg*)-RA, −112 CG3758(*esg*)-RA, −265 CG3758(*esg*)-RA, −89 CG3758(*esg*)-RA, −105 CG3758(*esg*)-RA, −153 CG3758(*esg*)-RA, −93 CG3758(*esg*)-RA, −105 CG3758(*esg*)-RA, −270	*UAS-esg*	Snail family C2H2 zinc finger transcription factor	SNAI2
2112 (GSV1) 11450 (GSV6)	CG11798(*chn*)-RA, +780 CG11798(*chn*)-RB, +12782	*UAS-chn*	C2H2 zinc finger transcription factor	ZNF462
9032 (GSV6) 9416 (GSV6) 10126 (GSV6) 10914 (GSV6) 12693 (GSV6) 13666 (GSV6) 14392 (GSV6)	CG5393(*apt*)-RA, −254 CG5393(*apt*)-RA, −254 CG5393(*apt*)-RA, −394 CG5393(*apt*)-RA, −182 CG5393(*apt*)-RA, −249 CG5393(*apt*)-RA, −296 CG5393(*apt*)-RA, −315	*UAS-tdf*	SANT domain transcription factor	none
2149 (GSV1) 2273 (GSV1)	CG3779(*numb*)-RB, −37 CG3779(*numb*)-RB, −35	*UAS-numb*	Membrane associated regulator of intracellular signalling	NUMB
2198 (GSV1) 14080 (GSV6)	CG10543-RA, +2050 CG10543-RA, +1620	Not validated	C2H2 zinc finger transcription factor	ZNF479
2067 (GSV1) 9929 (GSV6) 10455 (GSV6) 14364 (GSV6)	CG3696(*kis*)-RA, +28904 CG3696(*kis*)-RA, +1341 CG3696(*kis*)-RA, +28429 CG3696(*kis*)-RA, +28276	Not validated	SNF2-like chromo domain protein	CHD7
9498 (GSV6) 11321 (GSV6)	CG8084(*ana*)-RA, −52 CG8084(*ana*)-RA, −51	Not validated	Secreted glycoprotein	none
7333 (GSV2) 11052 (GSV6)	No tagged gene No tagged gene	Not validated	-	-

The overexpression of *ab* in *scrib* mutant clones was unique amongst the interactors in promoting a block to pupariation and massive tumour overgrowth throughout an extended larval stage. Both *GS* lines were inserted within the 5′ region of *ab*, and orientated so as to overexpress *ab*, and an independent *UAS-ab* line reproduced the same cooperative effect as the two *GS* lines. Analysis of *scrib^−^*+*ab* larval eye disc clones at day 5 revealed that differentiation of eye disc tissue was completely abrogated, as judged by the failure to express the photoreceptor differentiation marker Elav, although expression of the antennal cell fate marker, Dll, was retained within the growing tumour (**Figure 1A–H**). By day 9, huge invasive tumours had developed and become fused with the brain lobes (**Figure 1I,J**). In contrast, the overexpression of *ab* in otherwise wild type eye disc clones promoted antennal disc overgrowth, and sometimes resulted in the formation of ectopic Dll-positive antennal-like structures, however, it did not block photoreceptor differentiation and the larvae pupated, although most died during pupal development (**Figure 1E,F**). Analysis of proliferation by ethynyl deoxyuridine (EdU) incorporation confirmed that whilst *ab*-expressing clones exhibited a relatively normal pattern of proliferation, *scrib* ^−^+*ab* tumours ectopically proliferated and disrupted the normal pattern of cell proliferation within the eye disc (**Figure S2**). Furthermore, Terminal deoxynucleotidyl transferase dUTP nick end labeling (TUNEL) stains indicated that although *scrib* mutant cells undergo apoptosis [12], [14], cell death in *scrib^−^*+*ab* discs was mainly confined to the wild type tissue surrounding the growing tumours, although interestingly, *ab*-expressing clones alone were also characterised by increased cell death of wild type cells bordering the clones (**Figure S2**). Therefore, similar to *Ras^ACT^* or *Notch^ACT^*, the overexpression of *ab* can cooperate with the loss of *scrib* to block cell death and differentiation, and promote unrestrained and invasive tissue overgrowth, thus acting as a highly potent novel oncogene in *Drosophila*.

### Ab acts as a global transcriptional regulator at the promoters of many genes

Ab is a transcription factor with a BTB protein interaction domain and zinc finger DNA binding domains [15]–[17]. To build a comprehensive picture of how Ab functions in its oncogenic capacity, we employed Affymetrix expression arrays to identify the transcriptional changes induced by *ab* overexpression both alone and in combination with the loss of *scrib*, and combined this with Ab Chromatin Immunoprecipitation-Sequencing (ChIP-Seq) to identify those genes that were potential direct targets of Ab-mediated regulation.

**Figure 2:**
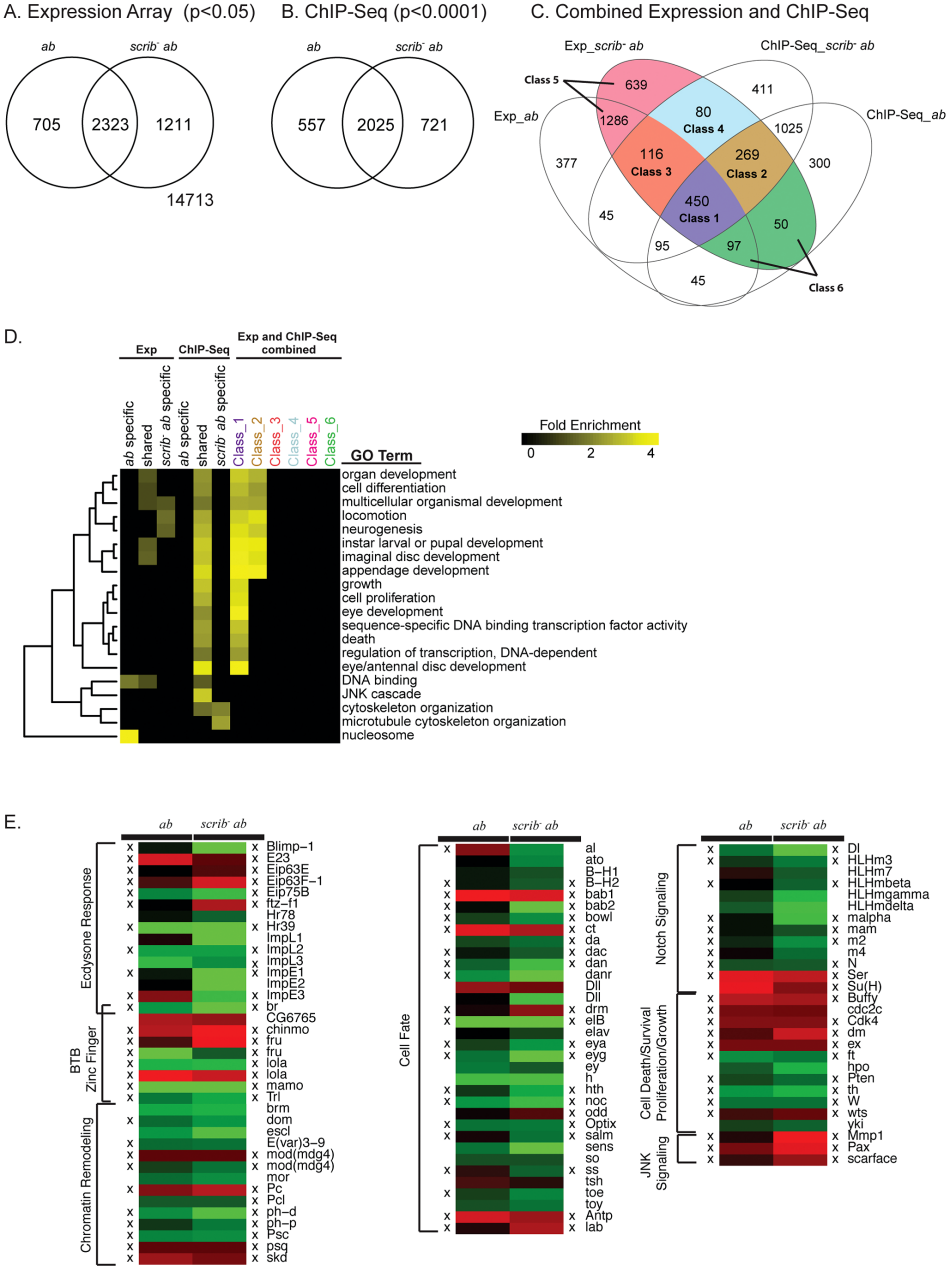
Potential tumourigenic targets of Ab identified from expression array and ChIP-Seq analysis. (A) Venn diagram showing the number of differentially expressed probe sets (log base 2 fold change >1, adjusted p value <0.05) within mosaic *ab* overexpressing eye/antennal discs compared to control mosaic discs, and *scrib^1^*+*ab* mosaic discs compared to the control mosaic discs. (B) Venn diagram showing the number genes identified as potential Ab targets based upon the occurrence of a significant peak (see Materials and Methods) either within 500 bp upstream of the transcription start site or within the introns of a gene, in either mosaic *ab*-expressing eye/antennal discs, or *scrib^1^*+*ab* mosaic discs, when compared to the respective input DNA controls. (C) Venn diagram that combines the results from the expression array and ChIP-Seq analysis. The six main classes of genes deregulated in *scrib^1^*+*ab* tumours are shown: Class 1, genes differentially expressed and associated with Ab peaks in both *ab* and *scrib^1^*+*ab* samples; Class 2, genes differentially expressed in *scrib^1^*+*ab* alone, but associated with Ab peaks in both samples; Class 3, genes differentially expressed in both *ab* and *scrib^1^*+*ab* samples, but associated with Ab peaks in *scrib^1^*+*ab* alone; Class 4, genes only differentially expressed and associated with Ab peaks in *scrib^1^*+*ab* alone; Class 5, genes deregulated in the *scrib^1^*+*ab* tumours but not associated with Ab peaks; and Class 6, genes deregulated in the *scrib^1^*+*ab* tumours but only associated with Ab peaks in the non-tumourigenic *ab*-expressing discs. Note that genes represented by multiple probe sets in the expression array are represented only once amongst the different classes, and assigned to either both genotypes if at least one probe set was significantly deregulated in both genotypes, or assigned to either *ab* or *scrib^−^*+*ab* uniques categories if at least one probe set was deregulated specifically in these genotypes. See **Dataset S1** for the complete gene lists associated with the expression array, ChIP-Seq and Classes 1–6. (D) Selected GO enrichments amongst the deregulated genes and potential Ab targets. See **Dataset S3** for the full listing of significantly enriched GO categories. (E) Heat map highlighting selected functional groups of deregulated genes identified from the expression array (red, upregulated; green, downregulated). For genes represented by multiple probe sets, the following probe sets are shown in the figure: *br* (1636931_at), *chinmo* (1636985_s_at), *Dll* (1636088_at, 1625771_at), *dom* (1628160_a_at), *Eip75B* (1635393_s_at), *elB* (1631207_at), *fru* (1641338_at, 1632859_a_at), *ImpE1* (1631375_a_at), *lola* (1633089_a_at, 1635096_at), *Mmp1* (1625761_a_at), *mod(mdg4)* (1627953_at, 1638041_at), *toy* (1633094_a_at), and *Trl* (1635305_s_at). See **Dataset S2** for a full listing of deregulated genes with multiple probe sets, and their relative expression in *ab* or *scrib* ^−^+*ab* tissue. A cross (X) denotes genes that were also identified from the ChIP-Seq analysis as potential Ab targets in either the *ab* alone, and/or *scrib^1^*+*ab* samples. See **Dataset S4** for ChIP-Seq genome alignments for these genes.

Tissue samples were prepared from mosaic eye/antennal discs overexpressing *ab* alone, or *ab* in *scrib* mutant clones, 5 days AEL. For the expression arrays, samples were compared back to control eye/antennal discs with wild type clones to identify differentially expressed probe sets (log base 2 fold change >1, adjusted p value [18] <0.05). This analysis indicated that Ab exerts a potent influence on gene expression, with 3028 and 3534 probe sets differentially expressed in *ab* and *scrib* ^−^+*ab* discs respectively, of which 2323 probe sets were shared between the two (**Figure 2A** and **Dataset S1**). The combined 4239 differentially expresssed probe sets encompassed 3549 annotated genes, 183 of which were represented by more than one probe set. The 183 genes with multiple probe sets were largely consistent in their pattern of expression changes in each genotype, although 59 of the 183 genes had probe sets that were both up and downregulated within the same genotype, possibly reflecting the existence of differentially expressed transcripts (**Dataset S2**). Quantitative real-time PCR validation of 5 representative genes confirmed the results of the expression array (**Figure S3**).

To identify genes that could be direct targets of Ab regulation, we performed ChIP-Seq after pulling down Ab-associated chromatin from *ab* alone expressing mosaic discs and *scrib* ^−^+*ab* mosaic tissue. The Ab antibody used for the pull-down has been widely used in the literature [17], [19], [20], and showed good specificity for Ab in eye/antennal disc tissue, as determined by reduced staining of endogenous Ab protein in *ab* mutant clones, and increased staining in *ab* over-expressing clones (**Figure S4**). Peak enrichments were identified by comparing each sample to input DNA controls (see Materials and Methods). Reflecting the large number of deregulated genes identified from the array, there were many peaks associated with Ab in both contexts (**Figure 2B** and **Dataset S1**). In the *ab* alone sample, 8881 peaks were identified, associated with the transcriptional start site or introns of 2582 genes; whilst in the *scrib* ^−^+*ab* tumourigenic sample, 10,892 Ab binding regions were identified, associated with 2746 genes. Validating these data, ChIP and quantitative real-time PCR for 10 candidate genes were consistent with the ChIP-Seq results (**Figure S5**), and there was also a high correlation between the *ab* and *scrib^−^*+*ab* samples (a correlation coefficient of ∼0.86). Of the potential target genes, 2025 were shared between the two samples, whilst 661 new binding site peaks, associated with 721 genes (some peaks overlapped more than one gene), were unique to the *scrib^−^*+*ab* tumourigenic sample (**Figure 2B**). Shared target genes were enriched for organismal development-related gene ontologies (GO), including “cell differentiation” (1.42E-70), “imaginal disc development” (2.39E-64), “imaginal disc morphogenesis” (1.92E-52) and “appendage development” (1.03E-42); whilst the 721 *scrib^−^*+*ab* unique genes, were enriched for the GO of “microtubule cytoskeleton” organization (4.58E-06) (**Figure 2D** and **Dataset S3**).

DNA recognition sequences for Ab have previously been suggested from its isolation as a protein capable of binding to the Engrailed binding site [15], and more recently through a bacterial one-hybrid study that defined a consensus Ab binding sequence [16], however, *in vivo* we observed no enrichments for these motifs amongst the Ab peak sequences (data not shown). Instead, the most highly represented motif amongst the most significant Ab peaks (irrespective of genomic location) from the *ab* overexpression sample, exhibited significant similarity to the recognition sequence for another BTB-ZF protein and transcriptional activator, the *Drosophila* GAGA factor, Trithorax-like (Trl) (**Figure S6**). Other motifs identified within the sequences associated with the most significant Ab peaks exhibited significant similarity to recognition sequences for Scalloped (Sd), which functions with Yki to activate target genes downstream of Hippo pathway signalling [reviewed in 10], and Brinker (Brk), which is a repressor downstream of the Dpp pathway [reviewed in 21]. We also searched the Ab peak sequences within the promoter regions or introns of potential target genes, for known transcription factor recognition site motifs. This approach also identified the Trl recognition sequence as one of the most highly represented motifs in peaks common to both the *ab* alone and *scrib* ^−^+*ab* samples (**Table S1**). Recognition sequences for the mammalian proteins MZF1 (similar to *Drosophila* Crooked legs (Crol), a zinc finger, ecdysone-induced gene that is also required for the expression of ecdysone response genes [22]) and VDR (similar to *Drosophila* hormone receptor HR96 and the ecdysone receptor EcR) were also identified, although the relevance of these sites for *Drosophila* proteins is not yet clear. In contrast, within peaks unique to the *scrib* ^−^+*ab* sample (and not within the common peaks) there was significant enrichment of binding sites for AP1 (the Jun/Fos transcription factor complex that acts downstream of the JNK signalling pathway), and REL and NF-KB (transcription factors that act downstream of the Toll-like receptor inflammatory signalling pathway), suggesting that new Ab target genes could be generated through the activation of JNK and associated inflammation pathways within the tumourigenic context.

### Prioritisation of Ab deregulated genes

To prioritise these data and focus upon those genes that could be transcriptional regulated by Ab and also critically required for tumour formation, we first removed from consideration all genes identified from the ChIP-Seq results that were not represented by probes on the array, and then combined the results from both analyses to identify those genes that were both associated with Ab peaks and differentially expressed from the microarray analysis (**Figure 2C**). This revealed that, for the *ab* alone sample, 27% of the differentially expressed genes (687 of 2511 genes), and for the *scrib* ^−^+*ab* sample, 31% of the (915 of 2987 genes), were associated with Ab peaks, and thus potentially defined primary targets of Ab-mediated regulation.

To identify potential direct targets of Ab that could be key to promoting tumour development, we focussed upon those targets that were both deregulated and associated with Ab peaks in the *scrib* ^−^+*ab* tumour sample. Of these, there were two classes of genes that were also associated with Ab peaks in the non-tumour samples, and either deregulated in both (Class 1), or just deregulated in the tumour sample alone (Class 2); and two further classes of genes only associated with Ab peaks in the tumour sample, but either also deregulated in both tumour and non-tumour (Class 3), or only deregulated in the tumour alone (Class 4). The largest of the four classes consisted of shared target genes deregulated in both tumour and non-tumour samples (Class 1, 450 genes), or in the *scrib* ^−^+*ab* sample alone (Class 2, 269 genes), whilst relatively few potential new targets of Ab (which were also deregulated genes) were generated in the tumour sample (116 genes in Class 3, and 80 genes in Class 4). In contrast, 2072 genes deregulated in the tumour were likely to be either secondary downstream targets of *ab* or targets deregulated by the loss of *scrib*, since they were not associated with Ab peaks (Class 5, 1925 genes), or were only associated with Ab peaks in the *ab* overexpression sample alone (Class 6, 147 genes).

Classes 3 and 4 did not exhibit significant GO enrichments, however, amongst the two primary classes of potential Ab target genes (Class 1 and 2) GO categories were identified that were involved in all aspects of tumour formation, from cell fate/differentiation, cell survival/growth/proliferation, and cell migration/invasion (**Figure 2D**, and **Dataset S3**). In contrast, genes deregulated, but not associated with Ab peaks in the tumour (Classes 5 and 6) did not show these GO enrichments. A heat map depicting the relative expression levels of selected genes from Classes 1 to 6 is shown in **Figure 2E** (see **Dataset S4** for ChIP-Seq peak alignments to the genome for the Class 1–4 targets depicted in this figure). The functional significance of these genes will be elaborated upon below.

### Ab binding is associated with the repression of multiple regulators of development and cell fate

The strongest GO enrichments amongst Ab targets included “multicellular organismal development” (1.76E-40 in Class 1 and 9.83E-22 in Class 2), “cell differentiation” (1.44E-28 in Class 1 and 7.65E-09 in Class 2) and “eye development” (3.72E-12 in Class 1) (**Figure 2D**). Amongst these targets were particular enrichments for ecdysone-response genes, other developmental genes involved with epigenetic control, Notch signalling, and the control of eye/antennal disc differentiation.

#### Ecdysone-response genes

**Figure 3:**
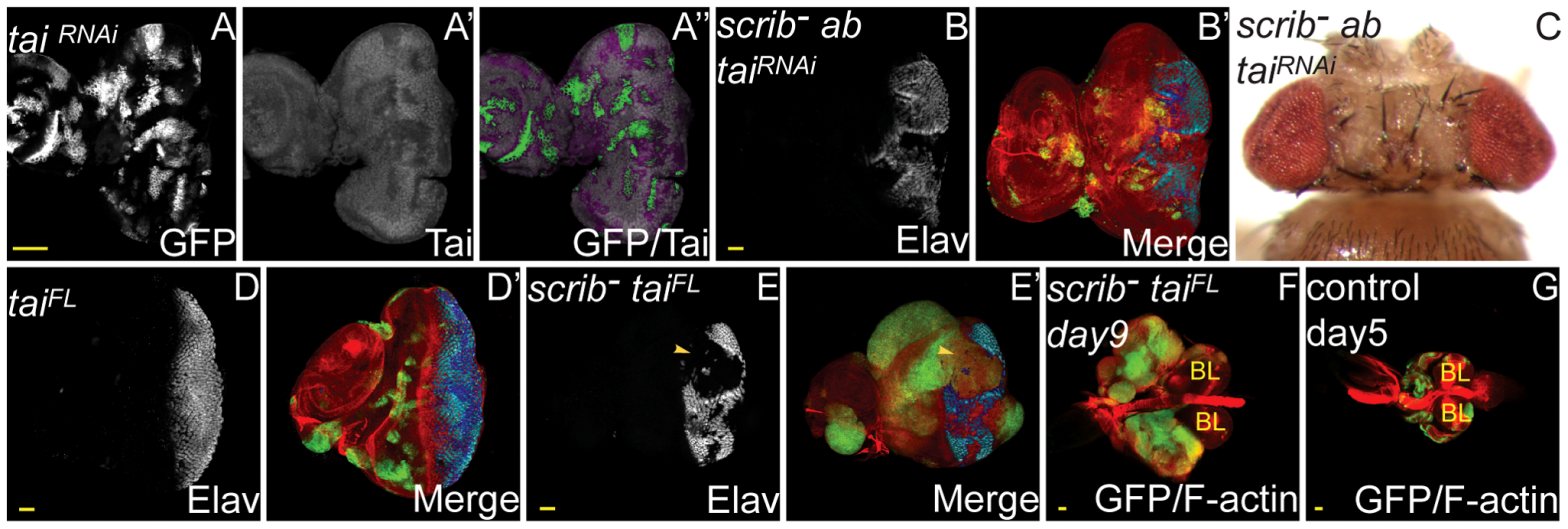
Tai is required for *scrib* ^−^+*ab* tumour overgrowth, and sufficient to cooperate with the loss of *scrib*. *ey-FLP* induced eye/antennal disc clones at 5 (A,B,D,E,G) and 9 days (F) AEL, and a dorsal view of mosaic adult eyes (C). Clones are marked by GFP (white, or green in merges). Tai (A) is white (and in merges appears magenta when overlaid with GFP), and Elav (B,D,E) is white (and blue in merges – dark blue when overlaid with GFP). F-actin is red in merges in B,D–G. Brain lobes in F,G are marked by BL. GFP (panel A), Tai (panel A′), Elav (panels B,D,E), and merges (panels A″,B′,D′,E′). (A) Expression of *tai^RNAi^* in clones reduces endogenous levels of Tai. (B–C) Expression of *tai^RNAi^* in *scrib^1^*+*ab* clones reduces clonal overgrowth (B) compared with *scrib^1^*+*ab* (see **Figure 1G–J**), and results in the eclosion of adult flies (C). (D) Overexpression of *tai^FL^* in clones does not block Elav expression, nor cause clonal overgrowth throughout an extended larval stage. (E–F) Overexpression of *tai^FL^* in *scrib^1^* clones promotes mutant tissue overgrowth and a block to Elav expression (E, arrowhead), eventually resulting in the formation of large tumours after an extended larval stage of development (F). (G) Wild type eye/antennal disc clones attached to the brain lobes at day 5, just before pupariation. Yellow scale bar = 50 µm.

Many ecdysone-response genes were identified as potential Ab targets, including *Eip75B*, *Eip78C*, *broad* (*br*), *ImpL2* (all Class 1; peaks in both *ab* and *scrib* ^−^+*ab*, and significantly deregulated in both), *ImpE1*, *ftz-f1*, *Blimp-1* (Class 2; peaks in both *ab* and *scrib* ^−^+*ab*, but significantly deregulated in *scrib* ^−^+*ab* alone), and *ImpL3* (Class 4; peaks in *scrib* ^−^+*ab* only, and only deregulated in *scrib* ^−^+*ab*). Confirming these data, protein levels of Br, a key ecdysone-induced gene, were substantially repressed in eye disc clones overexpressing *ab* (**Figure S7**). The involvement of Ab in the repression of ecdysone response genes is consistent with studies in the *Drosophila* ovary where Ab has also been shown to associate with the steroid hormone receptor coactivator, Taiman (Tai), and repress ecdysone response genes to control the timing of border cell migration [20]. Indeed, Tai is also expressed in the early 3^rd^ instar larval eye disc (**Figure 3A**), and knockdown of *tai* with RNAi in *scrib* ^−^+*ab* eye disc clones completely abrogated tumour overgrowth, restoring pupariation and resulting in the eclosion of adult flies (**Figure 3B,C**). Similar rescue of *scrib* ^−^+*ab* tumour formation was observed by ectopically expressing a form of *tai* that lacks the Ab interaction domain (Tai^ΔB^), and which has been shown to activate ecdysone response genes even in the presence of Ab [20] (**Figure S8**). Tai is therefore required for *scrib* ^−^+*ab* tumour development. Furthermore, whilst the overexpression of a wild type form of *tai* alone in clones did not induce tumour formation (**Figure 3D**) and adult flies eclosed (data not shown), overexpression of *tai* in *scrib* mutant clones was sufficient to induce clonal overgrowth and tumour formation throughout an extended larval stage, in a similar manner, albeit with less potency, as *ab* overexpression (**Figure 3E–G**). Thus, whilst it is not known if Ab directly cooperates with Tai in repressing ecdysone response genes in *scrib* ^−^+*ab* tumours, Tai is both required for *ab* to promote tumourigenesis, and sufficient to drive tumour formation in combination with the loss of *scrib*.

#### Epigenetic regulators

Many epigenetic regulators were transcriptionally deregulated in both *ab* alone and *scrib* ^−^+*ab* eye/antennal discs, including *Pc*, *ph-d*, *Psc*, *mod(mdg4)*, *psq*, *skd* (all Class 1), *ph-p* (Class 2), *Pcl* (Class 3), and a large number of other BTB-ZF transcription factors including *fru*, *chinmo*, *br*, *longitudinals lacking* (*lola*) and *Trl*. Most of these genes were repressed by *ab* overexpression, with the exception of *chinmo*, *pc*, *psq*, *skd* and some isoforms of *fru*, *lola* and *mod(mdg4)*. Other epigenetic regulators including *brm*, *mor* (both Class 5), *dom* and *E(var)3-9* (both Class 6) were also repressed in the tumour, although they were not associated with Ab peaks in the *scrib^−^*+*ab* sample, and were therefore likely to be indirect targets of Ab.

#### Notch-regulated genes

Notch signalling is a key regulator of cell fate decisions, and many Notch-associated genes were repressed in the tumour and associated with Ab peaks, including the *E(spl)* region genes *HLHm3*, *HLHmβ*, *m2* and *mα* (Class 2), the Notch transcriptional coactivator *mam* (Class 2) and the ligand *Delta* (*Dl*) (Class 1). Many other Notch targets, although not associated with Ab peaks, were also repressed in the tumourigenic state, including *HLHm7*, *HLHmγ*, *HLHmδ* (all Class 5), and *m4* (Class 6). Consistent with these data, the Notch reporter *E(spl)-lacZ*, normally activated in the differentiating portion of the eye disc, was repressed in *scrib* ^−^+*ab* tumours (data not shown). Furthermore, similarities between *ab* overexpression and *Notch* loss-of-function phenotypes have previously been reported [19].

#### Eye/antennal disc differentiation

The identification of Notch and ecdysone response genes as potential Ab targets validated our approach, however, most striking amongst the repressed Ab target genes associated with cell fate control were known regulators of eye/antennal disc differentiation, including *bab2*, *ct*, *dac*, *dan*, *daughterless* (*da*), *elbow* (*el*), *eya*, *eyegone* (*eyg*), *hairy* (*h*), *hth*, *no ocelli* (*noc*), *pannier* (*pnr*) and *spineless* (*ss*) (**Figure 2E**). This suggested that Ab could be functioning as an oncogene by maintaining cells within an undifferentiated state.

### Ab promotes tumourigenesis by blocking differentiation and maintaining *scrib* mutant cells within a progenitor-like state

**Figure 4:**
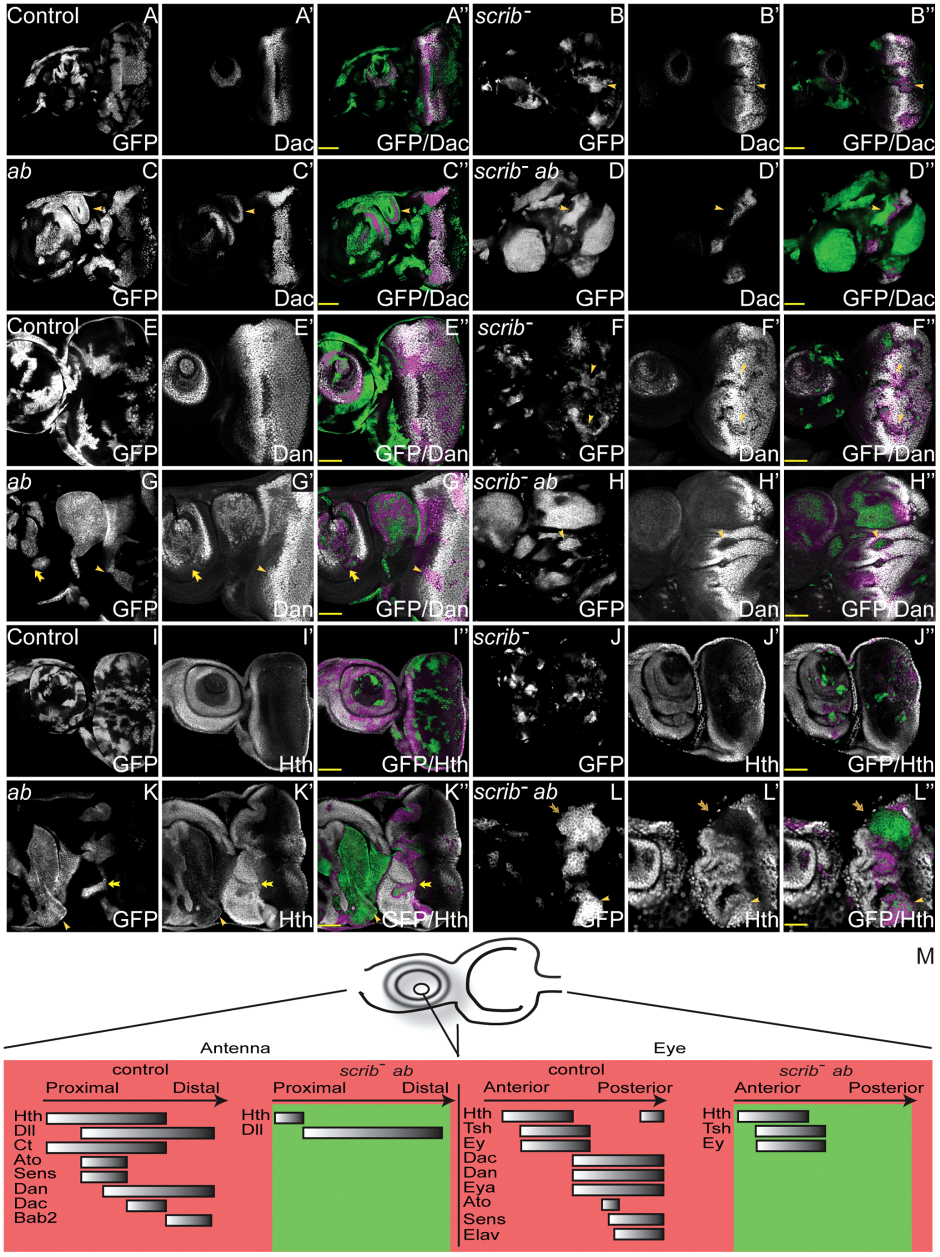
Overexpression of *ab* in *scrib* mutant clones promotes the retention of a progenitor-like state in the eye and antennal disc. *ey-FLP* induced eye/antennal disc clones at ∼5 days AEL. Clones are marked by GFP (white, or green in merges), and cell fate is shown by the expression of Dac, Dan and Hth (white, and magenta when overlaid with GFP in the merges) in wild type control clones (A,E,I), *scrib^1^* clones (B,F,J), *ab* overexpressing clones (C,G,K), and *scrib^1^*+*ab* clones (D,H,L). GFP (panels A–L), Dac (panels A′–D′), Dan (panels E′–H′), Hth (panels I′–L′) and merges (panels A″–L″). (A–D) Dac expression is only slightly reduced in *scrib^1^* clones (B, yellow arrowhead), and unaffected in *ab* overexpressing clones in the eye disc, although ectopic Dac expressing antennal-like structures are sometimes observed in the antenna (C, arrowhead). *scrib^1^*+*ab* clones do not express Dac (D, arrowhead; the magenta staining observed around some clones is derived from GFP bleed-through from underlying sections. (E–H) Dan levels are reduced in *scrib^1^* clones both in the antennal and eye disc (F, arrowheads). *ab* overexpressing clones do not affect Dan levels in the eye disc (G, arrowhead), although Dan is slightly repressed in the antenna (G, arrow), albeit ectopically expressed in the ectopic antennal-like structures. Dan is repressed in *scrib^1^*+*ab* clones (H, arrowhead). (I–L) Hth expression is generally unaffected in *scrib^1^* clones (J). In *ab* overexpressing clones, levels of Hth are slightly reduced in the eye disc (K, arrow), and large clones in the antennal disc do not express Hth (K, arrowhead). In *scrib^1^*+*ab* clones, Hth is expressed in some clones within the eye disc (L, arrowhead), but not all clones (L, arrow), and is generally reduced in antennal disc clones (L, and data not shown). (M) Diagram summarising the expression of cell fate markers in both wild type eye/antennal discs, as well as in eye/antennal disc *scrib* ^−^+*ab* tumours (green). In the antenna, proximal refers to the outer circular domains of the tissue, whilst distal refers to the inner, central domains. See **Figures S9** and **S10** for immunohistochemical images of Tsh, Ey, Eya, Ato and Sens; and **Table 2** for a summary of these results. Yellow scale bar = 50 µm.

Eye disc differentiation initiates from the posterior edge of the disc in the late 2^nd^ instar and progresses sequentially towards the anterior edge over a number of days. At the wandering third instar stage, when photoreceptor differentiation has progressed half way across the epithelium (as marked by Elav staining), the eye disc consists of a spectrum of various cellular differentiation states, from the most differentiated posterior cells to the least differentiated anterior cells. The transcription factors Hth, Eyeless (Ey) and Teashirt (Tsh) are expressed in the most anterior portion of the eye disc within the progenitor domain, whilst more posteriorly, Hth is first downregulated, followed subsequently by Ey and Tsh. The downregulation of Hth marks a transition point whereby cells begin to express Dac, Eya, Dan, Distal antenna-related (Danr), H, Da, Ato and finally Elav [reviewed in 6] (**Figure 4M**). In the antennal disc, the temporal development of the tissue is not displayed as a spatial distribution of cell fate markers at the third instar stage as it is in the eye disc, however, early to late cell fate transitions have been documented. Initial domains of Hth, Ct and Dll in the 2^nd^ instar larvae establish the early proximo-distal axis of the antenna [23], [24], and downstream targets of these genes, including the cell fate markers Ato, Dac, Dan, Bab2, Spalt major (Salm) and Ss, are subsequently expressed throughout the 2^nd^ and 3^rd^ instar to elaborate the proximo-distal axis of the appendage [7]–[9] (**Figure 4M**).

Multiple regulators of eye/antennal disc cell fate were repressed in *scrib* ^−^+*ab* tumours. Whilst some were repressed by expression of Ab alone, most of these were substantially further repressed in combination with the absence of *scrib*. Potential direct targets of Ab involved in regulating cell fate in the eye/antennal disc, and repressed in the tumour state, included *dan*, *eyg el*, *h* and *noc* (Class 1), *hth*, *dac*, *eya*, *bab2*, *pnr*, *ss* (Class 2), and *da* (Class 4). To further validate these results we examined the expression domains of the different cell fate regulators in the tumours. We had already established that *scrib* ^−^+*ab* tumours failed to express Elav in the eye disc (**Figure 1G**), however, examination of other cell fate markers, revealed that Dac, Dan, Eya, Sens and Ato were also repressed within the overgrowing eye disc tumour (**Figure 4A–H** and **Figure S9**). Importantly, all of these proteins (with the exception of Ato, which was also decreased in *scrib* mutant and *ab*-overexpressing clones) were not strongly downregulated in either *scrib* mutant clones alone, nor in *ab*-expressing clones alone, but only in cooperation with both genetic lesions (summarised in **Table 2**), thus validating the results from the expression array. In contrast, the expression of the cell fate markers that define earlier states, including Hth, Tsh and Ey were relatively unaffected in *scrib* ^−^+*ab* eye disc tumours, despite their domains of expression being enlarged and warped due to the growth of the tumour (**Figure 4I–L** and **Figure S10**), and with the exception of Hth (see below), were not identified as Ab targets. Furthermore, Hth, Tsh and Ey were all downregulated in more posterior tumour cells indicating that they were still being subject to their normal mode of repression. *scrib^−^* clones exhibited only minor perturbations in Hth, Tsh or Ey expression, whereas *ab*-expressing clones showed mildly reduced Hth and Ey, and slightly upregulated Tsh, expression levels.

**Table 2:** Expression of cell fate regulators in eye/antennal disc clones.

	*scrib^−^*	*abrupt*	*scrib^−^*+*abrupt*
**Eye**			
Elav	Disrupted and slightly reduced	Unaffected although spacing disrupted	Repressed
Ato	Disrupted and slightly reduced	Reduced and delayed	Repressed
Sens	Disrupted and slightly reduced	Unaffected	Repressed
Dac	Slightly reduced	Unaffected	Repressed
Dan	Slightly reduced	Unaffected	Repressed
Eya	Unaffected	Unaffected	Repressed
Hth	Unaffected	Slightly reduced, especially posteriorly	Variable, but generally unaffected
Tsh	Generally unaffected, although some ectopic expression extends posteriorly in large disorganised clones	Increased	Unaffected
Ey	Slightly reduced	Slightly reduced	Unaffected
**Antenna**			
Ato	-	Repressed	Repressed
Sens	Reduced	Repressed	Repressed
Dac	Slightly reduced	Ectopic in ectopic antennae-like structures	Repressed
Dan	Slightly reduced	Slightly reduced, but also ectopic in ectopic antennae-like structures	Repressed
Ct	Unaffected	Unaffected in proximal region but repressed more distally and in neck region	Repressed
Bab2	Unaffected	Slightly reduced, but also ectopic in ectopic antennae-like structures	Repressed
Hth	Unaffected	Repressed	Variable, but often repressed
Dll	Unaffected	Ectopic in ectopic antennae-like structures	Unaffected and ectopic in antennal-like overgrowths

In *scrib* ^−^+*ab* antennal disc tumours, most of the tumour tissue expressed Dll, whilst Hth and Ct expression was repressed (**Figure 4I–L**, **Figure S10** and data not shown). Furthermore, the expression of subsequent cell fate markers expressed along the proximo-distal axis, including Dan (Class 1), Bab2 and Dac (Class 2), as well as Sens and Ato, were also repressed within the tumours (**Figure 4A–H**, **Figure S9** and **Figure S10**). Their expression was only slightly perturbed in *scrib* mutant clones, whilst *ab*-expressing clones alone also repressed most of these markers, with the exception of Dac (summarised in **Table 2**). The repression of Hth in the Dll-positive tumour mass within the antenna suggested that the tissue was transformed to a more leg-like state [25], and, consistent with this, the HOX genes *Antennapedia* (*Antp*) and *labial* (*lab*) were also upregulated by *ab* overexpression (**Figure 2E** and **Dataset S1**).

The data therefore suggest that whilst *scrib* ^−^+*ab* eye/antennal disc tumours are not homogeneous, and consist of a diverse population of cells, they are characterised by the maintenance of an earlier progenitor-like cell state through the continual overgrowth of tissue that, in the eye disc, fails to transition to the expression of Dac, Eya, Ato and Elav, and in the antennal disc, fails to express differentiation markers downstream of Dll that define the elaboration of the appendage along the proximo-distal axis (summarised in **Figure 4M** and **Table 2**).

### The endogenous expression of Ab overlaps that of the progenitor state transcription factor Hth, but Hth is neither sufficient nor required for Ab-mediated tumour overgrowth

**Figure 5:**
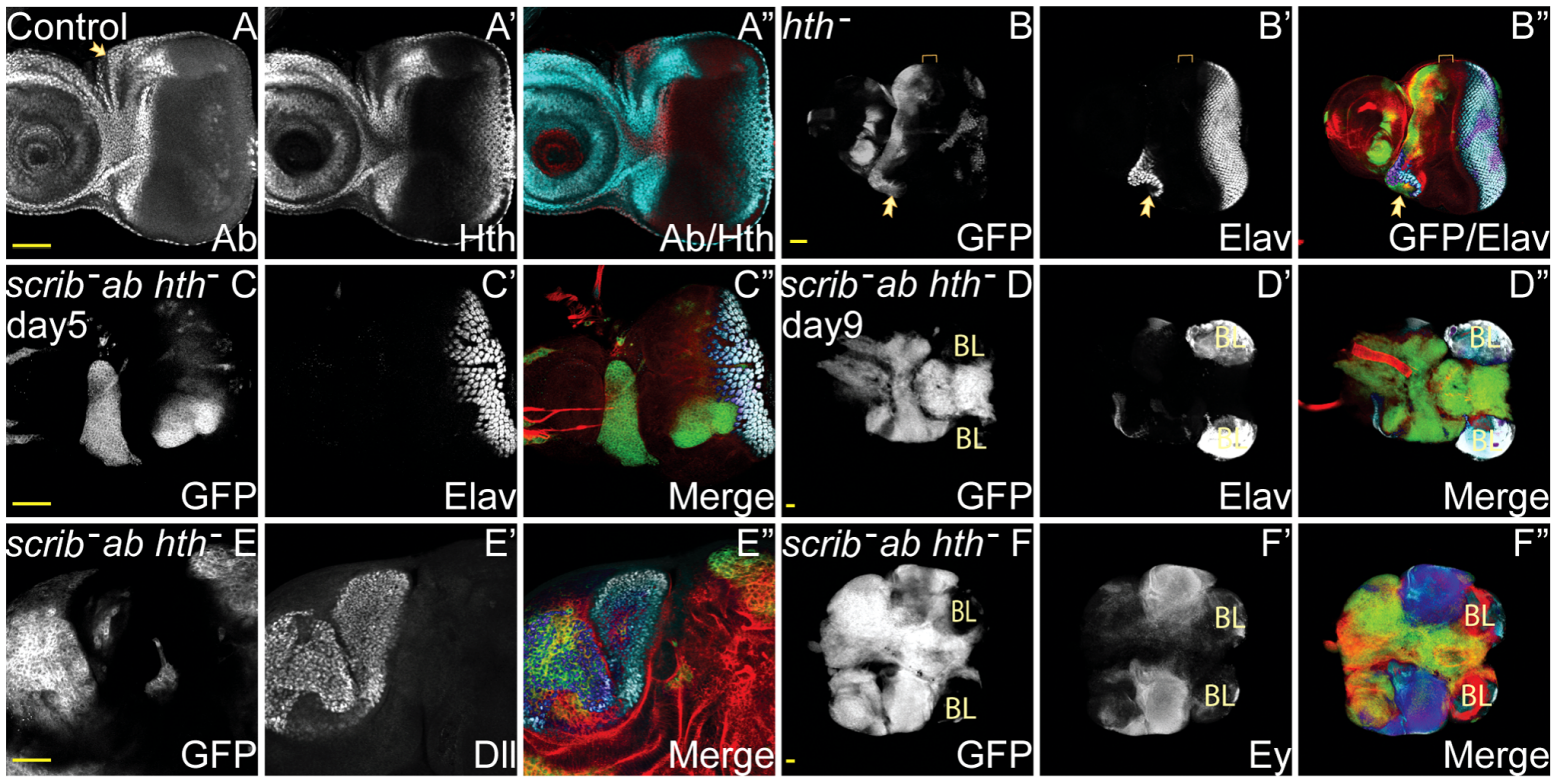
Hth is not essential for *scrib* ^−^+*ab* tumour overgrowth. *ey-FLP* induced eye/antennal disc clones at 5 (A–C) and 9 (D–F) days AEL. Clones are marked by GFP (white, or green in merges), and cell fate markers (Ab, Hth, Elav, Ey and Dll) are white (or blue in the merges, changing to dark blue when overlaid with GFP, except Ab, which is red in the merge in A). F-actin is shown in red in the merges of B–F. Brain lobes in D,F are marked by BL. Ab (panel A), Hth (panel A′), GFP (panels B–F), Elav (panels B′–D′), Dll (E′), Ey (F′), and merges (A″–F″). (A) Control discs show the endogenous expression of Ab and Hth in the eye/antennal disc, which overlap, except in the centre of the antennal disc and in the dorsally located ocelli (A, arrow), which express Ab but not Hth, and in the posterior part of the eye disc, which expresses Hth but not Ab. (B) *hth^P2^* clones are absent from the progenitor domain (bracketed), although overgrowth is sometimes observed within the neck region between the eye and antennal disc, and ectopic domains of Elav expression are sometimes generated within and adjacent to these mutant clones (B, arrow). (C–F) The overexpression of *ab* in *scrib^1^ hth^P2^* double mutant clones promotes overgrowth of clonal tissue that does not express Elav (C), and fuses with the brain lobes (BL) that are Elav positive (D). The expression of both Dll (E) and Ey (F) is maintained within the tumours. Yellow scale bar = 50 µm.

The capacity of Ab to maintain cells within a progenitor-like state suggested that its function might be linked to the eye disc progenitor state transcription factor, Hth. Indeed, the endogenous expression of Ab in the eye disc mirrored the expression of Hth (**Figure 5A**), and its downregulation in the eye disc heralds the beginning of Dac and Eya expression. Although our analysis of *scrib* ^−^+*ab* tumours indicated that Hth expression was not maintained in the tumours, and in fact was repressed in the antennal disc, it was still possible that Hth might be sufficient for tumour formation in combination with *scrib* mutants or required for *scrib* ^−^+*ab* tumour overgrowth.

To determine if Hth was sufficient to cooperate with the loss of *scrib*, we ectopically expressed Hth in *scrib* mutant clones. However, although this promoted overgrowth of the tumour tissue and pupal lethality, it did not result in a block to pupariation and massive tumour overgrowth throughout an extended larval stage of development, indicating that Hth could not substitute for Ab in a tumour-promoting role (**Figure S11**). Conversely, to test for whether Hth was required for *scrib* ^−^+*ab* tumour overgrowth, we overexpressed *ab* in *scrib^−^ hth^−^* double mutant clones and assayed for tumour formation. Examination of tumour samples at day 5 revealed that overgrowth was initially confined to regions within the neck and the ventral portion of the eye disc (**Figure 5C**), regions that correspond to tissue that is least dependent upon *hth* for cell survival and/or proliferation, and are associated with a role for *hth* in repressing ventral eye formation [26], [27]. Indeed, this tissue continued to grow in *scrib^−^ hth^−^*+*ab* tumours, so that whilst overgrowth was substantially delayed compared to *scrib* ^−^+*ab* tumours, massive and invasive tumour masses eventually overtook the larvae (**Figure 5D**). These tumours consisted of characteristic Dll-positive tumour masses within the antennal region, and Ey-positive tumour tissue within the eye disc (**Figure 5E,F**). Thus, *hth* is neither sufficient nor absolutely required for Ab-driven tumour formation.

### Yki promotes overgrowth of *scrib^−^*+*ab* tumours

To identify potential targets of Ab that could be important for maintaining tumour overgrowth, we analysed Class 1 and 2 genes for GO enrichments associated with cell survival and proliferation. Importantly, the GO categories of “cell death” (4.84 E-03), “growth” (1.09 E-06) and “cell proliferation” (2.03 E-05) were all enriched within Class 1 targets, which were genes associated with Ab peaks and deregulated in both *ab* alone and *scrib* ^−^+*ab* tumours. Amongst potential cell death targets, the pro-survival Bcl2 homologue *Buffy* was upregulated, and the cell death inducer *Hid* (*W*) was downregulated by *ab* overexpression. Furthermore, *klumpfuss* (*klu*) and *echinus* (*ec*) that promote cell death in the pupal retina [28]–[30], were also downregulated by *ab*. Notable Class 1 *ab* targets involved in cell growth and proliferation included the cell growth and G1-S phase driver *Cdk4* (upregulated), the inhibitor of the PI3K pathway *Pten* (downregulated) and a number of Hippo pathway components and/or targets, including *expanded* (*ex*), *fat* (*ft*), *thread* (*th*/*DIAP1*) and *diminutive* (*dm*), the *Drosophila* Myc gene. Whilst *th*, a survival-promoting effector of Yki activity, was repressed by *ab* overexpression, which was confirmed by immuno-histochemical analysis of *ab*-expressing larval discs (**Figure S12**), the Yki targets *dm* and *ex* were upregulated upon *ab* overexpression, and *ft* and *hippo* (Class 5), two negative tissue growth components of the Hippo pathway, were repressed. Thus, although multiple genes may contribute to *ab*-driven tumour overgrowth, *ab*-mediated impairment to the Hippo pathway could be a key factor.

**Figure 6:**
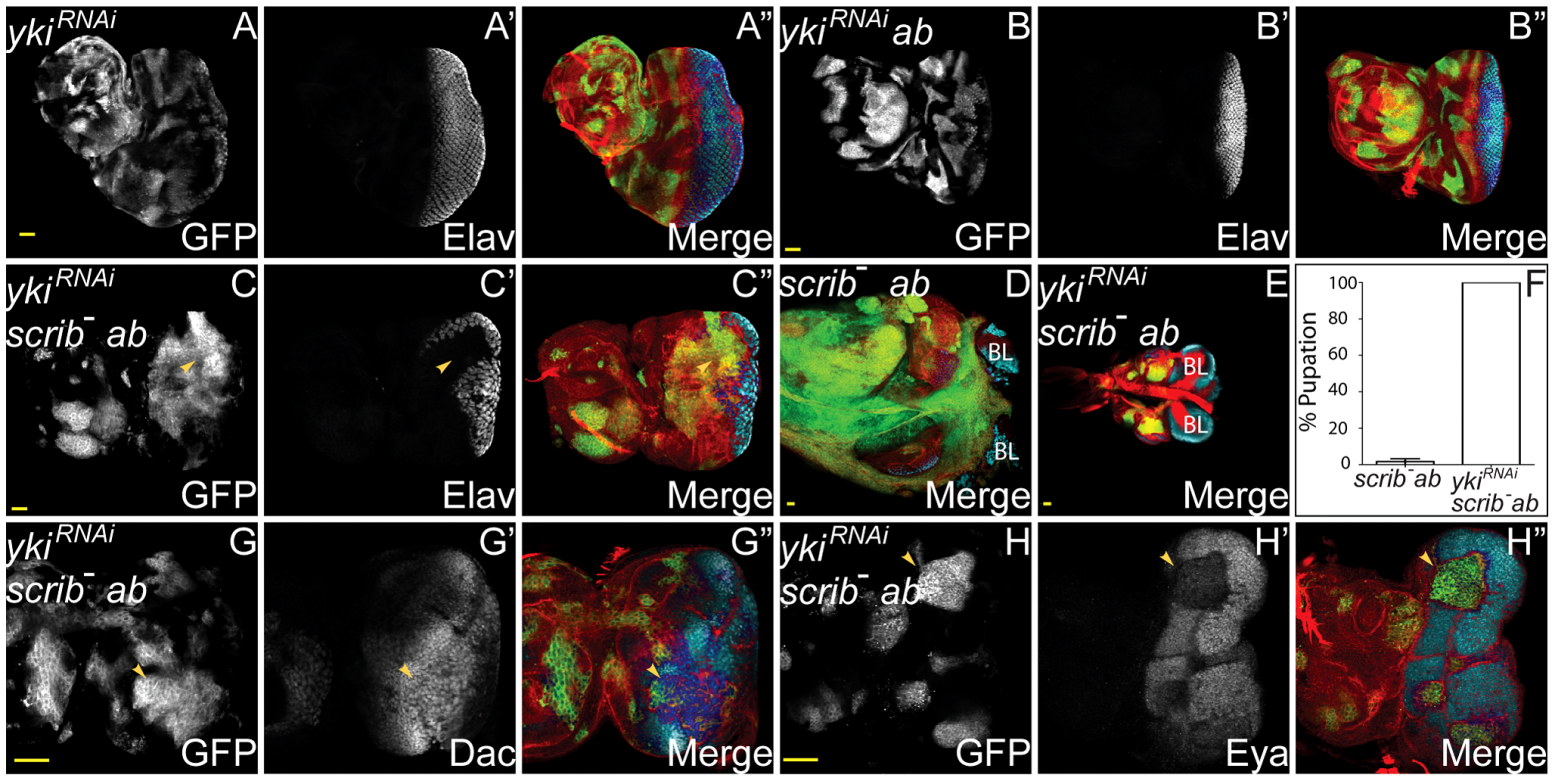
Yki is required for *scrib* ^−^+*ab* tumour overgrowth. *ey-FLP* induced eye/antennal disc clones marked by GFP (white, or green in merges). Elav, Dac and Eya are in white (blue in merged images, changing to dark blue when overlaid with GFP), and F-actin for cell morphology is in red. Brain lobes are labeled BL. GFP (panels A–C,G,H), Elav (panels A′–C′), Dac (panel G′), Eya (panel H′) and merges (panels A″–C″,D,E,G″,H″). (A) *yki^RNAi^*-expressing clones. (B) Clones overexpressing *yki^RNAi^*+*ab* are similar to *ab* overexpressing clones. (C–E) Expressing *yki^RNAi^* in *scrib^1^*+*ab* tumours does not restore Elav expression to the tumour cells (C, arrowhead), however, tumour overgrowth is substantially reduced (E, compared to *scrib^1^*+*ab* tumours in D), and larvae pupate instead of entering an extended larval stage. (F) Quantification of percentage of *scrib^1^*+*ab* and *scrib^1^*+*ab*+*yki^RNAi^* tumour-bearing larvae that had pupated by 9 days AEL. (G,H) Expressing *yki^RNAi^* in *scrib^1^*+*ab* tumours restores Dac expression in the clones (G, arrowhead) but Eya levels remain reduced (H, arrowhead). Yellow scale bar = 50 µm.

A role for the Hippo pathway in *scrib* ^−^+*ab* tumour overgrowth was tested by knocking down *yki*, a critical downstream transcriptional effector of impaired Hippo pathway signalling. Strikingly, and unlike loss of *hth*, this substantially restrained *scrib* ^−^+*ab* tumour overgrowth and restored pupariation to the tumour-bearing larvae (**Figure 6A–F**). To determine whether the rescue in tumour overgrowth was accompanied by a restoration to differentiation we examined the expression of cell fate markers. This revealed that whilst knockdown of *yki* did not restore Elav and Eya expression to *scrib^−^*+*ab* tumours, Dac levels were substantially increased (**Figure 6G,H**). It was therefore possible that the increased levels of Dac upon *yki* knockdown could account for the suppression of tumour overgrowth. However, overexpressing *dac* within *scrib* ^−^+*ab* tumours, using a *dac* transgene, failed to restrain tumour overgrowth and restore pupariation (**Figure S13**). Thus, the downregulation of Dac is not a key requirement for continual tumour overgrowth. Furthermore, *scrib^−^*+*ab* tumour cells expressing *yki^RNAi^*, could still be observed with mesenchymal morphology between the brain lobes (**Figure S14**), suggesting that whilst Yki activity is required for tumour overgrowth, it is not an essential mediator of tumour cell migration and invasion.

### Impaired Hippo signalling is sufficient to cooperate with Ab and promote tumour overgrowth

**Figure 7:**
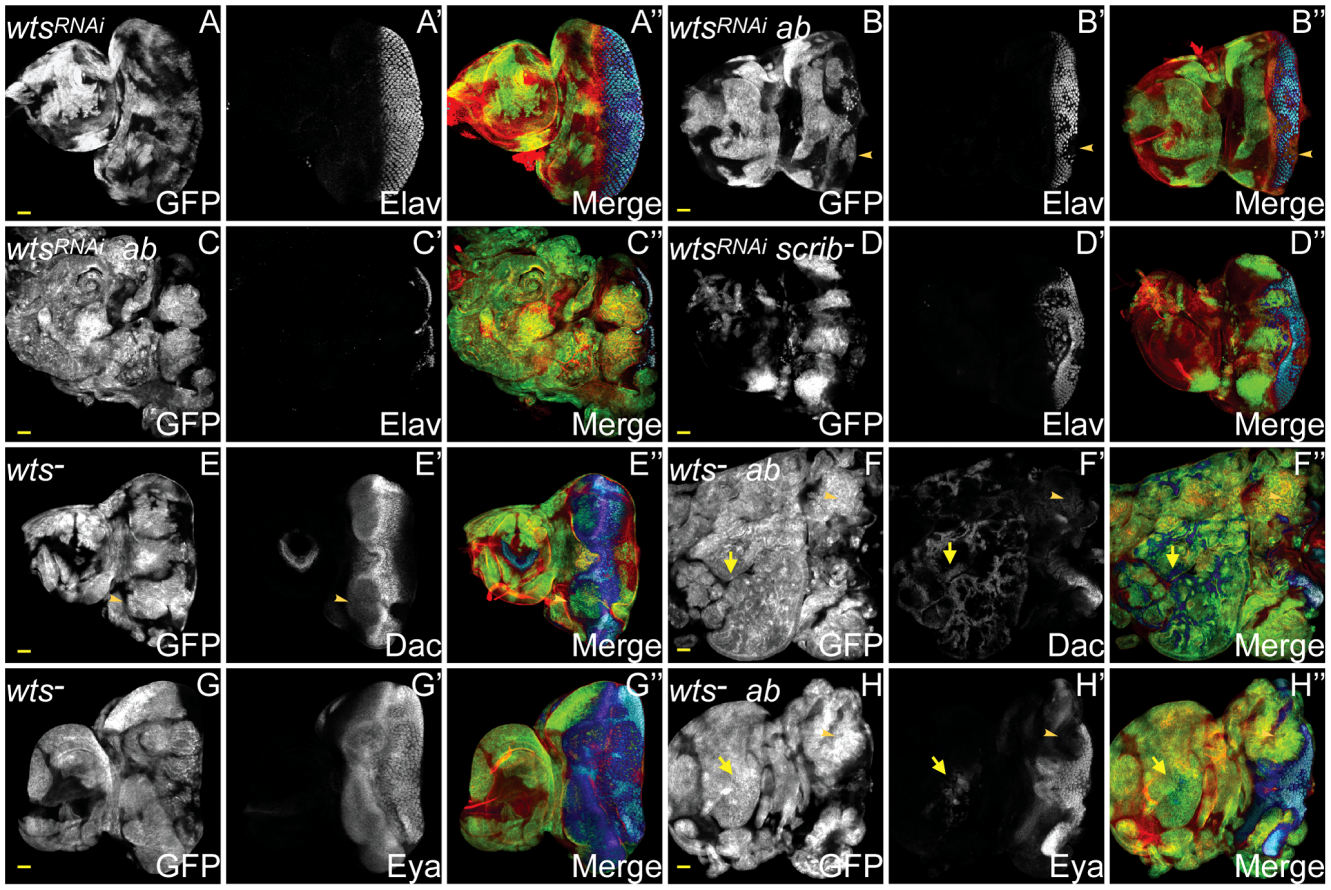
*ab* cooperates with impaired Hippo pathway signalling to drive tumour overgrowth. *ey-FLP* induced eye/antennal disc clones marked by GFP (white, or green in merges). The cell fate markers Elav, Dac and Eya are shown in white (blue in merged images, changing to dark blue when overlaid with GFP). F-actin for cell morphology is in red. GFP (panels A–H), Elav (panels A′–D′), Dac (panels E′F′), Eya (panels G′H′), and merges (panels A″–H″). (A) *wts^RNAi^*-expressing clones exhibit the normal pattern of Elav in the eye disc. (B) Coexpressing *wts^RNAi^*+*ab* in clones decreases Elav expression in some (B, arrowhead), but not all clones, and some larvae enter an extended larval stage, during which massive overgrowth of Elav-negative tissue ensues (C). (D) Overexpressing *wts^RNAi^* in *scrib* mutant clones increases *scrib* mutant clone size and reduces Elav expression, but does not result in cooperative tumour overgrowth throughout an extended larval stage. (E) *wts^X1^* clones exhibit mildly reduced Dac levels in anterior localised clonal tissue in the eye (E, arrowhead), and also reduced expression in the antennal disc. (F) In *wts^X1^*+*ab* clones, overgrowing tissue within the eye disc does not express Dac (F, arrowhead), although extensive ectopic Dac expression is observed throughout the antennal disc (F, arrow). (G) Eya expression in *wts^X1^* clones is largely unperturbed. (H) *wts^X1^*+*ab* clones overgrow in the eye disc, and do not express Eya (H, arrowhead), however, occasional Eya positive tissue is sometimes observed within the antennal disc region (H, arrow). Yellow scale bar = 50 µm.

The ChIP-Seq and expression array analysis had indicated that *ab* overexpression was capable of modulating Hippo pathway activity, however, *scrib* mutant cells also express Hippo pathway reporters, and ectopically proliferate in a Yki-dependent manner [31]. Thus both the overexpression of *ab* and the loss of *scrib* each had the potential to promote Yki activity, and either of these could be crucial in driving cooperative tumour overgrowth. To discern which of the two was more critical in mediating cooperation we tested for whether knockdown of *wts* in either *ab*-overexpressing clones or *scrib* mutant clones, was sufficient to elicit cooperative tumour overgrowth throughout an extended larval stage. Whilst knockdown of *wts* alone in clones did not perturb Elav expression and larvae pupated normally (**Figure 7A**), ectopically expressing *ab* in *wts^RNAi^* clones was sufficient to block pupariation of larvae and promote massive overgrowth of the eye/antennal discs. Examination of Elav expression indicated that although at day 5 some *wts^RNAi^*+*ab* clones were still observed to express Elav, the overgrown clonal tissue that ensued was entirely composed of Elav-negative tissue (**Figure 7B,C**). Similar cooperation was observed when *ab* was ectopically expressed within *wts^X1^* mutant clones (data not shown). In contrast, although knockdown of *wts* in *scrib* mutant clones enhanced *scrib* mutant tissue overgrowth causing pupal lethality, it was not sufficient to completely block Elav expression and drive cooperative tumour overgrowth throughout an extended larval stage of development (**Figure 7D** and data not shown). Consistent with this interpretation, *scrib*-mediated impairment to Hippo signalling has been shown to be atypical Protein Kinase C (aPKC)-dependent, since it is rescued by expressing a kinase dead dominant negative (DN) version of *aPKC* (*aPKC^DN^*) within the mutant tissue [31], and similarly, expressing *aPKC^DN^* in *scrib* ^−^+*ab* tumours also curtailed tumour overgrowth (**Figure S15**). Thus, whilst *ab* overexpression alone may impair Hippo pathway signalling, the deregulation of the Hippo pathway induced by the absence of *scrib* is likely to be a key factor in promoting susceptibility to Ab-driven tumour formation.

To determine whether expressing *ab* in *wts* mutant clones produced tumours that were similar to *scrib^−^*+*ab* tumours, we examined the expression of different cell fate markers in *wts^−^*+*ab* clones. *wts* mutant clones differentiated normally, apart from a mild downregulation of Dac (**Figure 7E,G** and **Figure S16**). However, although some of the *wt*s*^−^*+*ab* clonal tissue at day 5 expressed normal, or only mildly reduced, levels of Dac, Dan and Eya (data not shown), in older larvae, the overgrown *wts^−^*+*ab* tumours consisted predominantly of eye disc progenitor-like tissue that did not express Dac, Dan or Eya, and antennal-like tissue that ectopically expressed Dll and Dac (**Figure 7F,H** and **Figure S16**). Thus, the *wts^−^*+*ab* tumours retained a progenitor-like state that was similar to *scrib* ^−^+*ab* tumours, with the exception that Dac expression was retained within the antennal domain of the *wt*s*^−^*-derived tumours, but not in the *scrib^−^*-derived tumours. Furthermore, *wts^−^*+*ab* tumours were characterised by the generation of huge, highly-folded epithelial sheets of tissue that remained distinct and did not fuse with the brain lobes, thus indicating that cooperation between *wts* ^−^+*ab* was unable to reproduce the invasive properties of *scrib* ^−^+*ab* tumours.

### Ab targets are involved in migration and invasion, but Ab can not promote invasion without JNK signalling

The invasive properties of Ras^ACT^ and Notch^ACT^-driven tumours are dependent upon JNK signalling, since blocking *Drosophila* JNK (Basket (Bsk)), within either *scrib* ^−^+*Ras^ACT^* or *scrib* ^−^+*Notch^ACT^* tumours prevents tumour cell invasion [14], [32], [33]. The expression array of *scrib* ^−^+*ab* tumours indicated that JNK signalling was also likely to be active within these tumours, as evidenced by the upregulation of known JNK-regulated genes such as *Matrix metalloproteinase 1* (*Mmp1*) and *scarface* (*scaf*) [32], [34], which were also identified as potential Ab targets (Class 1 and 2, respectively). In addition, GO analysis of Class 1 and 2 targets of Ab indicated a significant enrichment for genes within the category of “locomotion” (6.67 E-12 in Class 1 and 2.72 E-07 in Class 2). In the *scrib* ^−^+*ab* tumours these included *wunen*, *wunen2* and *Trapped in endoderm 1* (*Tre1*) that are known to promote germ cell migration, and *jing* and *PDGF- and VEGF-related factor 1* (*Pvf1*) that are involved in border cell migration [reviewed in 35]. Thus, the data suggested that Ab could directly contribute to the invasive capability of *scrib* ^−^+*ab* tumour cells by controlling the expression of migration-associated genes, including JNK targets such as *Mmp1*.

**Figure 8:**
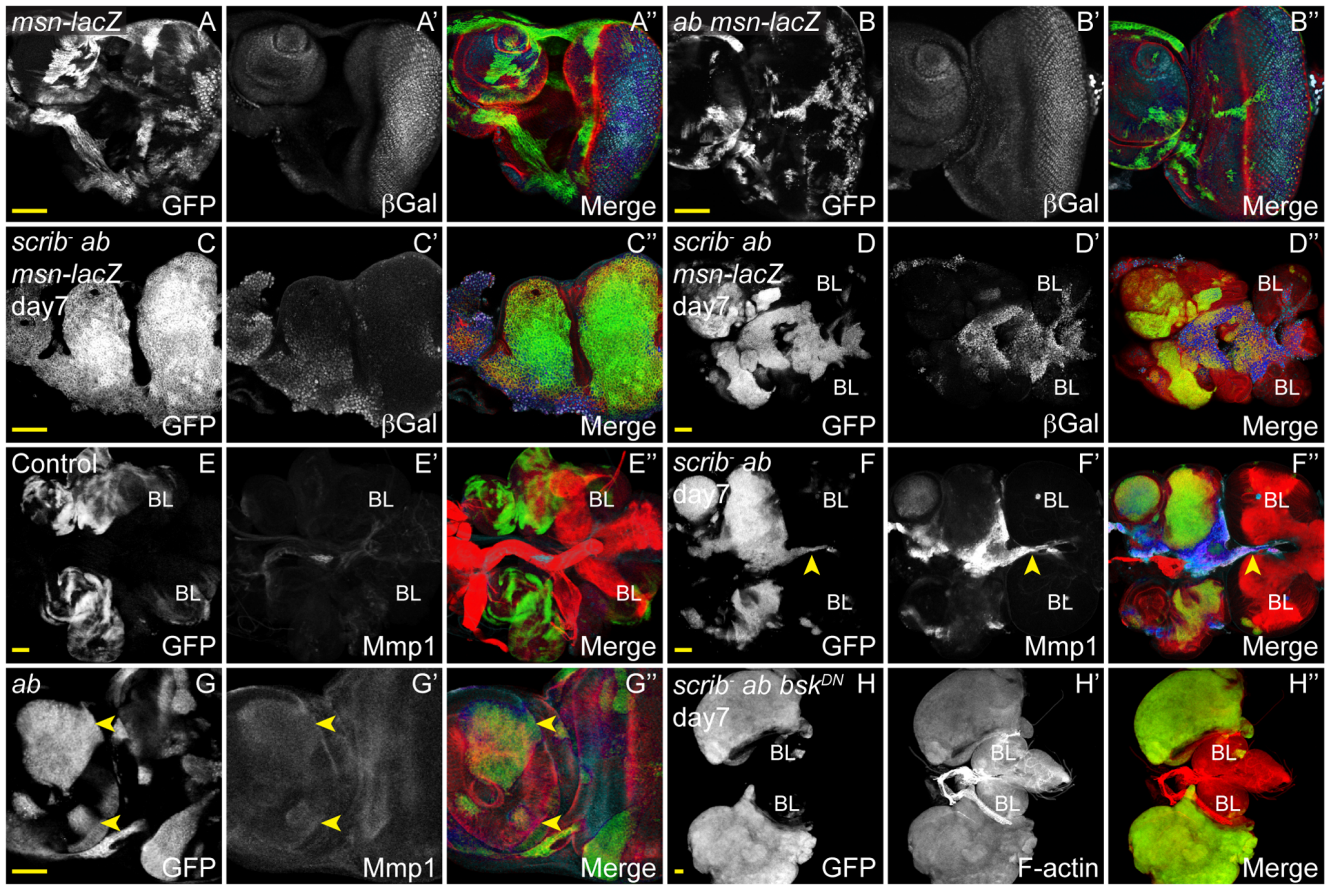
JNK signalling in *scrib^−^*+*ab* tumours is required for invasion, but not tumour overgrowth. *ey-FLP* induced eye/antennal disc clones at 5 (A,B,E,G) and 7 days (C,D,F,H) AEL. Clones are marked by GFP (white, or green in merges), and JNK signalling is indicated by β-Gal expression from the *msn^06946^-lacZ* enhancer trap or Mmp1 expression (white, and blue in the merges). Tissue morphology is shown by F-actin (red in merges). Brain lobes in D,E,F,H are marked by BL. GFP (panels A–H), β-Gal (A′–D′), Mmp1 (E′–G′), F-actin (H′) and merges (A″–H″). (A) Control clones show the normal pattern of *msn-lacZ* expression in the eye antennal disc. (B) Clones overexpressing *ab* do not alter the normal pattern of *msn-lacZ* expression. (C,D) *scrib^1^*+*ab* clones show ectopic expression of *msn-lacZ* in some cells (C), including those that are fusing with the brain lobes (D). (E,F) Mmp1 levels are elevated in *scrib* ^1^+*ab* tumour cells migrating between the brain lobes (F, arrowhead), compared to control eye discs and brain lobes (E). (G) Mmp1 levels are slightly elevated in some *ab*-expressing clones (G, arrowheads). (H) *scrib^1^*+*ab*+*bsk^DN^* clones massively overgrow similar to *scrib* ^1^+*ab* tumours, however, the eye/antennal discs do not fuse with each other or with the brain lobes, and the tumour cells show no evidence of invasive migration between the brain lobes. Yellow scale bar = 50 µm.

Using the JNK pathway reporter, *misshapen* (*msn*)*-lacZ* [36], we first determined whether JNK signalling was active in *scrib^−^*+*ab* tumours. Indeed, although *ab* overexpressing clones alone did not upregulate *msn-lacZ* expression (**Figure 8A,B**), the reporter was strongly activated within *scrib^−^*+*ab* tumours, most notably within basal portions of the tumour and in cells that appeared to be migrating between the brain lobes, consistent with a role for JNK in promoting invasion (**Figure 8C,D**). Immunohistochemical analysis also indicated that the JNK target, *Mmp1*, was ectopically expressed within *scrib* ^−^+*ab* tumours (**Figure 8E,F**), and, in agreement with the expression array, Mmp1 levels were also slightly elevated in *ab* alone overexpressing clones (**Figure 8G**). To next determine whether Ab was capable of promoting invasion, independent of JNK signalling, we then examined *scrib* ^−^+*ab* tumours in which JNK signalling was blocked, using a dominant negative JNK transgene (*bsk^DN^*). Strikingly, the expression of *bsk^DN^* in *scrib* ^−^+*ab* tumours prevented the fusion of the discs to one another and to the brain lobes, thus demonstrating a critical role for JNK in mediating the invasive properties of the tumours (**Figure 8H**). To confirm the benign nature of the overgrowths we used live cell imaging to monitor the growth of the tumours over time. The *scrib* ^−^+*ab* tumour cells were highly motile with individual cells moving rapidly into the brain (**Movie S1**). In contrast, the *scrib* ^−^+*ab*+*bsk^DN^* tumours remained compact, despite their massive growth throughout an extended larval stage of development (**Movie S2**). Thus, although *ab* overexpression may contribute to the invasive properties of the tumours by promoting the expression of targets such as *Mmp1*, it is not sufficient to promote tumour invasion in the absence of JNK signalling. In this regard, *ab*-driven tumours resemble Ras^ACT^ and Notch^ACT^-driven tumours, although, interestingly, expressing *bsk^DN^* in Ras^ACT^ and Notch^ACT^ tumours additionally restores pupariation to the tumour-bearing larvae, thus curtailing tumour overgrowth [14], [32], [33]. In contrast, the formation of massive, albeit benign, *scrib^−^*+*ab*+*bsk^DN^* tumours during an extended larval stage, indicated that *ab* blocks pupariation and promotes *scrib* ^−^ tumour overgrowth, even in the absence of JNK signalling.

### Overview of cooperating pathways in *scrib^−^*+*ab* tumours

**Figure 9:**
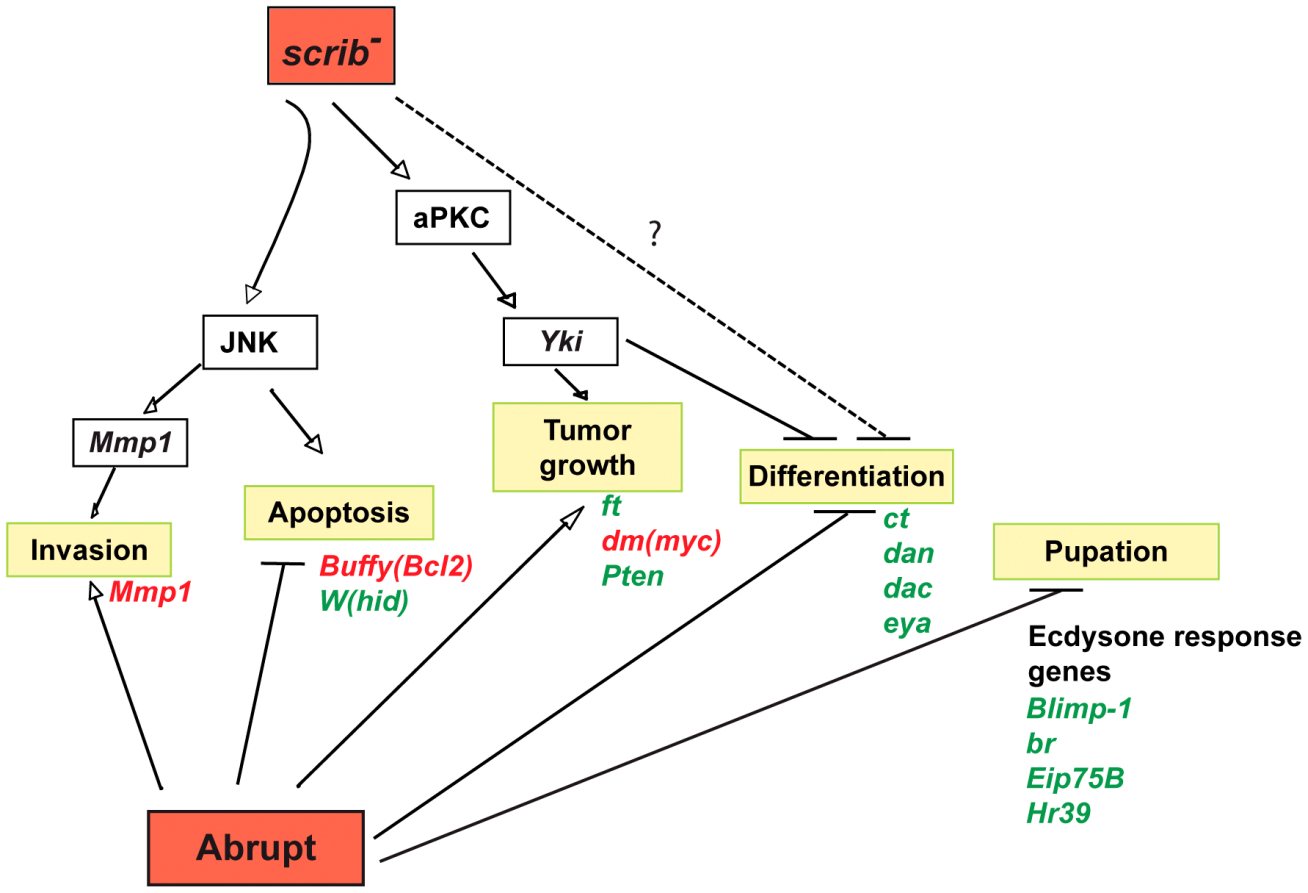
Model illustrating the pathways involved in *scrib* ^−^+*ab* cooperative tumour overgrowth. Ab cooperates with the loss of *scrib* to form invasive tumours through modulating the expression of multiple genes involved in all aspects of tumour formation. Potential targets of Ab include genes involved with blocking apoptosis and promoting tumour overgrowth (eg. *hid*, *Buffy*, *ft*, *dm*, *Pten*), genes required for eye/antennal disc differentiation (eg. *ct*, *dac*, *eya*, *dan*), genes involved in promoting cell invasion (eg. *Mmp1*), and genes involved in the ecdysone-induced pupariation response (eg. *Blimp-1*, *br*, *Eip75E*, *Hr39*). Whilst not shown on the figure, the steroid hormone receptor coactivator Tai is both required for *ab*-driven tumour overgrowth and sufficient to cooperate with the loss of *scrib*, consistent with the possibility that Ab acts in concert with Tai to drive tumour formation. Loss of *scrib* activates JNK-mediated apoptosis, however, *ab* overexpression abrogates the apoptotic response, thereby unmasking a key role for JNK in promoting tumour cell migration and invasion through the expression of JNK-induced genes such as *Mmp1*. Loss of *scrib* also promotes aPKC-dependent Yki activity that is required and sufficient to cooperate with Ab by impairing differentiation and promoting tumour overgrowth. Other pathways deregulated in *scrib* mutants may participate in the tumour phenotype and promote the full spectrum of differentiation defects seen in *scrib* ^−^+*ab* tumours (indicated by the dotted blocking arrow and question mark), such as Dac repression in the antenna. Green = downregulated genes, and red = upregulated genes.

In summary, this comprehensive analysis has identified multiple modes through which the overexpression of *ab* and loss of *scrib* cooperate to promote the retention of a progenitor-like cell state and the formation of invasive tumours (**Figure 9**). The overexpression of *ab* modulates the expression of a significant proportion of the genome to block differentiation, repress ecdysone signalling (potentially through direct association with the ecdysone receptor coactivator Tai), and promote cell survival and proliferation; whilst loss of *scrib* induces aPKC-dependent Yki activity to promote tumour overgrowth, and JNK signalling to promote invasion. Indeed, deregulation of the Hippo pathway is sufficient to cooperate with Ab and drive the formation of large, albeit benign, tumours, although the deregulation of additional pathways in *scrib* mutants may contribute to the complete spectrum of overgrowth and differentiation defects observed in *scrib^−^*+*ab* tumours.

## Discussion

In this study we show in *Drosophila* that; 1) the BTB-ZF transcription factor Abrupt acts as a potent oncogene when combined with the loss of *scrib* in the eye/antennal disc; 2) *scrib^−^*+*ab* epithelial tumours are associated with an earlier developmental state; 3) impaired Hippo signalling in *scrib* mutants is a key factor in mediating cooperative overgrowth with *ab* overexpression; and 4) *ab* can promote tumour overgrowth, but not invasion, independently of JNK signalling. Abrupt thus joins a growing list of BTB-ZF proteins with potent oncogenic potential, including another *Drosophila* member of the family, Lola, which cooperates with the ectopic expression of the Notch ligand, Delta, to form metastatic tumours in *Drosophila* [37], as well as numerous mammalian BTB-ZF proteins that are also implicated as human oncogenes [reviewed in 38].

Underpinning the conclusions of this study is a description of the transcriptional changes and potential direct targets of Ab in *scrib^−^*+*ab* tumourigenesis. This has revealed a complex picture of widespread transcriptional deregulation upon Ab overexpression, and a multitude of potential Ab target genes. The *in vivo* binding sequence for Ab is not known, however, the most highly enriched motif from the ChIP-Seq has similarity to the binding sequence of another BTB-ZF protein, Trl. Whether this reflects a predilection for Ab to bind a similar recognition sequence as Trl, or whether Ab competes or cooperates with Trl to regulate transcription, will require further analysis. Interestingly, mammalian BTB-ZF proteins can heterodimerise, and are also known to associate with histone deacetylases (HDACs) or other corepressors to control of cell fate through transcriptional repression [reviewed in 39]. It is not known if Ab associates with HDACs, however, there was not a clear bias in Ab targets for genes that were specifically downregulated in the *scrib^−^*+*ab* tumour. Nevertheless, many of the most notable Ab targets were repressed in the tumourigenic state, and this included numerous Notch pathway targets and ecdysone response genes, as well as a suite of transcription factors responsible for orchestrating the differentiation of the eye and antennal disc.

### Hormone signalling and developmental timing in tumourigenesis

Repressed ecdysone response genes were enriched amongst potential Ab targets, consistent with the known capacity of Ab to directly associate with the steroid hormone receptor coactivator Tai and repress the expression of ecdysone response genes in the *Drosophila* ovary [20]. Indeed, we show that Tai is both required for Ab to exert its oncogenic effect in the eye/antennal disc, and sufficient when overexpressed to cooperate with *scrib* ^−^ and promote the formation of large tumours throughout an extended larval stage. Thus, it is possible that Ab also associates with Tai in its oncogenic role, although further work will be required to determine if this is the case. The human homologue of Tai, SRC3/AIB1, is also a transcriptional coactivator of steroid hormone receptors and an oncogene [reviewed in 40], although whether it associates with BTB-ZF proteins is not yet known.

The repression of multiple ecdysone-response genes within *scrib^−^*+*ab* tumours is striking, yet whether this plays a cell autonomous role in promoting tumour overgrowth is unclear. However, the tumour-bearing larvae also fail to undergo an ecdysone-induced pupariation response, and this non-cell autonomous block in organismal development functions to extend the time frame available for continual tumour overgrowth. Indeed, an extended larval stage is a phenotype elicited by both neoplastic tumour overgrowth [41] and tissue damage [42], whereby it functions to give time for tissue regeneration before initiating pupariation. A key factor in mediating this delay is *Drosophila* insulin-like peptide 8 (Dilp8), which is secreted from tumours or damaged tissues, and acts as a diffusible signal to repress the biosynthesis of ecdysone [43], [44]. *dilp8* expression can be induced by JNK signalling [43], which is consistent with previous studies indicating that JNK signalling within *scrib^−^*+*Ras^ACT^* and *scrib^−^*+*Notch^ACT^* tumours is essential for the failure of the tumour-bearing larvae to pupate [14], [32], [33]. In contrast, we show here that *ab*-driven tumour overgrowth throughout an extended larval stage does not require JNK signalling. Possibly this reflects a capacity of Ab to directly or indirectly regulate *dilp8* expression, independent of JNK. Indeed, the expression array indicated that *dilp8* was upregulated in both *ab*-expressing eye/antennal discs, and in *scrib* ^−^+*ab* tumours, although the ChIP analysis indicated that *dilp8* was only associated with Ab peaks in the *ab* alone expressing sample (Class 6). Why *ab*-expressing larvae pupate (unlike *scrib* ^−^+*ab* larvae), despite the elevated levels of *dilp8* expression, remains to be determined. Interestingly, known endogenous functions of Ab are also associated with regulating the timing of hormone-induced developmental transitions, including the correct timing of border cell migration in the *Drosophila* ovary [20], and neuromuscular junction formation during metamorphosis [45]. In both contexts, and similar to its oncogenic role, Ab expression is associated with earlier developmental states, and its ectopic expression can inhibit temporal progression towards differentiation.

### The role of Ab in mediating impaired differentiation during tumourigenesis

The cells of the adult eye are derived from progenitor cells within the 3^rd^ instar eye disc that are characterised by the expression of a number of transcription factors including Hth, Tsh, Ey, ElB and Noc. The endogenous expression of Ab overlaps with Hth, and both Hth and Ab are downregulated prior to the downregulation of Tsh, Ey, ElB and Noc, and coincident with the upregulation of Dac, Eya, So and Dan. In *ab*-driven tumours, the expression of *dac*, *eya* and *dan* was blocked, as were other downstream posteriorly-expressed differentiation markers (*ato*, *Elav*, *sens*), thereby maintaining cells within an earlier progenitor-like state. The expression array also indicated that *elB* and *noc* were repressed in *ab*-driven tumours, and although these genes are normally expressed within the progenitor region, *elB* and *noc* mutants promote overgrowth of Hth-positive progenitor cells [46]. As the ChIP analysis indicated that Ab binding was associated with many of these genes, including *eya*, *dac*, *dan*, *elB* and *noc*, we suggest that Ab may promote the maintenance of a progenitor-like state by directly repressing many of these differentiation-promoting genes, although further work will be required to verify this hypothesis. Interestingly, the failure to transition to Eya, Dac and Dan expression and the maintenance of a progenitor-like state may be sufficient to promote over-proliferation of tumour cells in the eye disc, since not only is loss of *elB* and *noc* associated with over-proliferation of progenitor cells, but also loss of *eya* promotes tissue overgrowth in the eye, although this is eventually restrained through the induction of cell death [47], [48], and the ectopic expression of *hth* and *tsh* can block *eya* and *dac* expression and also promote eye disc overgrowth [49]. Ab, however, appears capable of maintaining eye disc tumour overgrowth independent of both *hth* and *tsh*, since Hth and Tsh levels are repressed in the posteriorly localised tumour cells, and *ab* overexpression can promote overgrowth of eye disc tumour tissue, even in the absence of *hth*. Thus, whilst the repression of multiple differentiation-promoting genes in *ab*-driven eye disc tumours might cooperate to elicit a default over-proliferative progenitor-like state, this state does not appear to be defined by simply maintaining the expression of the known progenitor state factors, Hth and Tsh.

Progenitor cells in the antennal disc are not as well defined as in the eye disc. However, overgrowing tumour tissue in the antennal region was characterised by the expression of Dll and Hth, whilst all other cell fate markers examined were repressed in the tumour, including Ct, Dan, Bab2, Ato and Sens. A number of other antennal cell fate markers, although not examined by immunohistochemistry, were also identified from the expression array as significantly repressed within the tumour including *aristaless* (*al*), *brother of odd with entrails limited* (*bowl*), *danr*, *salm* and *ss*. Although the 3^rd^ instar antennal disc, unlike the eye disc, does not present itself as a spectrum of early to late cell fate states marked by the expression of different transcription factors, significant detail is known concerning the temporal development of the appendage from the embryonic stage onwards. The expression of *hth* and *dll*, defining the proximal and distal domains respectively, are one of the first divisions to be established in the developing appendage. Neither are required for each others expression [50], however, the expression of most other cell fate regulators that define the elaboration of the appendage along the proximodistal axis are dependent upon either or both of their activities [7], [9], [51]–[53]. Thus, in *scrib* ^−^+*ab* tumours, the expression of cell fate markers downstream of *dll* and *hth* are repressed resulting in the overgrowth of antennal primordia tissue that has a defined proximo-distal axis that fails to transition towards a further differentiated state. In this regard, the tissue becomes indistinguishable from the developmentally related leg appendages in their early state. Indeed, within the tumours, most of the Dll-positive tissue does not express Hth, making the tumour tissue more characteristic of a leg-like, as opposed to an antennal-like, state. Consistent with this, the two HOX genes *Antp*, a repressor of *hth* [25], and *lab* were ectopically expressed within the tumours, and both are capable of transforming the antennae to a leg-like fate [54]. We propose that similar to the eye disc, this alteration in cell fate and block in expression of downstream cell fate regulators is directly mediated by Ab, since Ab binding was associated with most of the downstream genes. Whether this block is sufficient for tumour cells to be maintained within a proliferative state is not yet known. However, as in the eye disc, it is possible that multiple mechanisms cooperate to promote the full spectrum of tumour overgrowth. Furthermore, both *ab* overexpression and loss of *bowl* (which was repressed in the tumours) can induce the development of ectopic antennae within the eye/antennal disc [19], and whilst the cause of these phenomena is not yet clear, the eye/antennal disc is derived from the fusion of multiple segments, and it has been suggested that ectopic appendages might arise from reawakened appendage primordia that have been cryptically retained within the composite tissue [55]. Thus it is possible that within *ab*-driven tumours, multiple segmental appendage primordia could be contributing to tumour overgrowth.

In summary, parallels emerge between *scrib^−^*+*ab* tumour overgrowth in the eye and antennal disc, in that both are characterised by the maintenance of an earlier developmental state downstream of Hth but upstream of Dac. However, neither Hth activity, nor the downregulation of *dac*, appear to be essential for tumour overgrowth, thus indicating that further work is required to identify what key transcription factors define the progenitor-like state in *ab*-driven tumours. As many of the eye/antennal disc transcription factors targeted by Ab have human orthologues that are also implicated in cell fate regulation, organogenesis and tumourigenesis (eg. Hth (MEIS family), Dac (DACH family), Eya (EYA family), Dll (DLX family)), it is likely that this work will promote a deeper understanding of how cell fate control also influences the formation of human cancers.

### The role of the Hippo pathway in maintaining the progenitor-like state

The proliferation of progenitor cells within the eye disc is *yki* dependent [11], and although the requirement for Yki activity in antennal disc cells has not been examined, *yki* is required for *scrib* ^−^+*ab* tumour overgrowth in both the eye and antennal disc. The requirement for Yki in *scrib* ^−^+*ab* tumours could solely reflect a basal need for Yki activity in progenitor cell proliferation, however, loss of *scrib* impairs Hippo pathway signalling [31], and we show here that blocking Hippo signalling is sufficient to cooperate with *ab* and sustain massive tumour overgrowth. Furthermore, the expression array indicated that *ab* overexpression may also deregulate the Hippo pathway, as can the BTB-ZF protein Trl [56], which is known to directly associate with Yki [57]. Interestingly, the mode of Hippo pathway deregulation induced by the overexpression of *ab* is likely to be different to that induced by the loss of *scrib*. The Yki targets activated in *scrib* mutants include CycE, DIAP1, *fj-lacZ* and *ex-lacZ* [12], [31], however, *ab* overexpression upregulated the Yki targets *dm* (Myc) and *ex*, but both *th* (DIAP1) and *fj-lacZ* (data not shown) were mildly repressed. As both *fj* and *th* are also targets of the JAK/STAT pathway [58], [59], Ab may additionally function to repress JAK/STAT signalling, as does another *Drosophila* BTB-ZF protein, Ken and barbie (Ken) [60]. Increasing complexity is being recognised in the variety of transcriptional outputs of the Hippo pathway. In the progenitor domain, Yki associates with Hth and Tsh, and instead of promoting *th* expression, it drives expression of the pro-survival micro-RNA *bantam*, which represses translation of the cell death inducer *hid* (*W*) [11]. A similar capacity could be shared by *ab* overexpression, and potentially it might be the bringing together of two different modes of Hippo pathway deregulation (both *scrib* mutant and *ab* overexpression dependent) that makes the combining of these two oncogenic forces so potent.

Although loss of *wts* was sufficient to cooperate with *ab* and promote massive overgrowth of undifferentiated tissue, it was not sufficient to reproduce the entire spectrum of defects in *scrib* ^−^+*ab* tumours. The non-invasive nature of *wts* ^−^+*ab* tumours is likely to reflect the lack of JNK pathway activity and/or the maintenance of epithelial cell polarity within the tumours. However, whether these additional defects also account for the differences in expression of cell fate regulators is not clear. Whilst overgrowth of *wts* ^−^+*ab* tumours was characterised by the failure to express Eya and Dan, Dac was ectopically expressed within the antennal tumours. Interestingly, Dac defines the medial domain of the appendage and is one of the first markers to be expressed downstream of Hth and Dll. Thus it may be the least refractory to inhibition, relative to slightly later acting cell fate regulators. This contrasts with the eye disc in which knockdown of *yki* in *scrib* ^−^+*ab* tumours restored Dac expression, but not Eya or Elav. Whilst this could indicate that, unlike the antennal disc, Dac alone is repressed by Yki activity in the eye, an alternative explanation could be that in both the antennal and eye discs Dac repression requires substantially higher levels of Yki activity than repression of the other cell fate markers. This might make Dac particularly susceptible to restoration when *yki* is knocked down in the tumours, and conversely, only subject to repression in *scrib* ^−^+*ab* tumours when Yki activity is especially high.

Even though Hippo pathway mutants are not usually associated with a failure to differentiate, the potential for the Hippo pathway to elicit effects upon cell fate is not without precedence. Impaired Hippo signalling can synergise with loss of *Drosophila* Retinoblastoma gene (*rbf*) to cause dedifferentiation of photoreceptor cells in the eye disc, independent of effects on cell proliferation [61]; and in the larval brain Yki overexpression can delay differentiation of the neuroepithelia and promote overgrowth of the progenitor cells, although in this case the effects are likely to be a consequence of accelerated cell cycle progression [62]. However, most pertinent to this study, Yki overexpression throughout the eye disc is sufficient to block Eya expression and expand Hth expression [63]. Interestingly, these effects upon Hth levels were specifically linked to exceptionally high Yki activity, since it could not be reproduced by knockdown of *wts* alone, but only by combining *wts* knockdown with additional loss of *ft* and *ex* [63]. Thus, additive effects that escalate Yki activity elicit qualitatively different effects, and this is likely to be relevant to both our own analysis of *ab*-driven tumours, as well as more generally to understanding how cooperating pathways synergise to drive tumourigenesis.

### Parallels to mammalian BTB-ZF transcription factors

There are over 40 human BTB-ZF family members, many of which are implicated in both haematopoietic and epithelial cancers, where they act as oncogenes (e.g. BCL6, ZBTB7) or tumour suppressors (e.g. PLZF, HIC1) [reviewed in 38]. They are key regulators of cell fate, most notably within the immune system [reviewed in 64], and a number of studies are also consistent with roles in regulating self-renewal and differentiation. A specific orthologue of *ab* is difficult to ascertain because of the low sequence conservation between *Drosophila* and mammalian family members, although ZFP161 exhibits the greatest amino acid sequence similarity. ZFP161 is not known to exert oncogenic activity within humans, and indeed its expression in some tumours is more consistent with a potential tumour suppressor role [65], however, Bcl6, one of the best-characterised mammalian oncogenic family members, offers striking parallels to the function of Ab. Mainly implicated in lymphomas, Bcl6 expression is also associated with epithelial cancers, and can promote self-renewal and repress differentiation of both B cells [66] and mammary epithelia [67]. A key oncogenic target of Bcl6-mediated repression in lymphomas is *blimp-1*, and the two proteins antagonise each other's expression to regulate lymphocyte differentiation [reviewed in 68]. Importantly, *Drosophila Blimp-1* is induced by ecdysone [69], and was also identified as an Ab target, being one of the most highly repressed genes within the tumour. Bcl6 can also repress Notch signalling in *Xenopus* [70], similar to the repression of Notch targets by Ab in *Drosophila*. Although it is not yet known whether Bcl6 associates with the Tai orthologue, the activity of other BTB-ZF proteins are linked with various nuclear hormone receptors and their corepressors including NCoR and SMRT, suggesting that integration with hormone signalling pathways is a feature shared by mammalian family members. Overall, our identification of *ab* as an oncogene that cooperates with *scrib* loss of function in tumourigenesis, and analysis of cooperating pathways in *Drosophila* tumours, have uncovered striking parallels to mammalian tumourigenesis. It highlights the potent oncogenic potential of this class of proteins, supports prevailing views of the importance of impaired differentiation of progenitor cells as key drivers of neoplastic overgrowth, and raises the possibility that mammalian regulators of epithelial cell polarity could also act as important restraints upon the oncogenic potential of the BTB-ZF family of proteins.

## Materials and Methods

### *Drosophila* stocks

The following *Drosophila* stocks were used: *ey-FLP1,UAS-mCD8-GFP;;Tub-GAL4,FRT82B,Tub-GAL80* [71]; *y,w,hs-FLP*; *FRT82B,Ubi-GFP*; *UAS-ab^55^* [72]; *UAS-ab^79^* [72]; *FRT40A,ab^1D^* [17]; *UAS-bsk^DN^* [73]; *UAS-chn* [74]; *UAS-DaPKC^CAAXDN^* [75]; *UAS-dac* [76]; *UAS-dl* [77]; *UAS-esg* [78]; *E(spl)m8 2.61-lacZ* [79]; *UAS-hth* [27]; *hth^P2^* [80]; *msn^06946^ (msn-lacZ)* [81]; *UAS-numb-GFP* [82]; *FRT82B,scrib^1^* [83]; *UAS-tai^FL^* [84]; *UAS-Tai^DB^* [20]; *UAS-tai^RNAi^* (VDRC #15709); *UAS-tdf* (also known as *apt*) [85]; *wts^X1^* [86]; *UAS-wts^RNAi^* (NIG #12072R-1); *UAS-yki^RNAi^* [87].

### Mosaic analysis

Clonal analysis utilised MARCM (mosaic analysis with repressible cell marker) [88] with *FRT82B* and *ey*-*FLP1* to induce clones and *mCD8-GFP* expression to mark mutant tissue. All fly crosses were carried out at 25°C and grown on standard fly media.

### F3 screen for cooperating oncogenes

A random selection of *GS*(mini-*w^+^*) insertions on the second chromosome (obtained from the NIG stock centre in Kyoto), carrying *GAL4* sites to overexpress flanking genes, were screened for their oncogenic potential by initially crossing to *w* ^−^;;*TM3/TM6B* flies. Male progeny of the genotype *w^−^*;+/*GS*(mini-*w^+^*);*+*/*TM6B* were then selected to cross to virgin *w^−^*;+/*CyO*;*FRT82B,scrib^1^*/*TM3*,Sb flies. From their progeny, *w^−^*;*GS*(mini-*w* ^+^)/*CyO*;*FRT82B,scrib^1^*/*TM6B* male flies were then crossed to *ey-FLP1*,*UAS-mCD8.GFP*;;*tub-GAL4*,*FRT82B*,*tub-GAL80*/*TM6B* virgins to generate progeny containing *scrib^1^* eye disc clones overexpressing the gene(s) downstream of the *UAS* sites and flanking the *GS* insertion point. The adult flies (at least 50) of the resulting progeny were examined to determine whether the expression of the *GS* line in *scrib^1^* clones was lethal, or whether it enhanced the *scrib^1^* mosaic adult eye phenotype in the non-*TM6B* progeny. Any crosses exhibiting lethality were then examined under fluorescent light to determine whether the non-*TM6B* progeny exhibited GFP-positive tumour overgrowth or a failure to pupate. The insertion point and over-expressed genes of any identified *GS* lines was obtained by referring to the *Drosophila* Gene Search Project web site (http://kyotofly.kit.jp/stocks/documents/GS_lines.html).

### Immunohistochemistry

Imaginal discs were dissected in phosphate-buffered saline (PBS) from either wandering 3^rd^ instar larvae or from staged lays for larvae of genotypes that failed to pupate and entered an extended larval stage of development. Tissues were fixed in 4% formaldehyde in PBS, and blocked in 2% goat serum in PBT (PBS 0.1% Triton X-100). For the detection of S phase cells, EdU labelling was performed for 30 min at room temperature according to the manufacturers protocol (Invitrogen). TUNEL assays were performed as described in the manufacturers protocol (Roche Applied Science). Primary antibodies were incubated with the samples in block overnight at 4°C, and were used at the following concentrations; rabbit anti-Ab (S. Crews [17], 1/200), rabbit anti-Ato ([89], 1/1000), rat anti-Bab2 ([90], 1/1000), mouse anti-β-galactosidase (Rockland, 1/400), mouse anti-Br-core (Developmental Studies Hybridoma Bank (DSHB), 1/200), mouse anti-Ct (DSHB, 1/100), mouse anti-Dac (DSHB, 1/10), rat anti-Dan ([9], 1/300), mouse anti-DIAP1 (B. Hay, 1/100), mouse anti-Dll ([8], 1/500), mouse anti-Elav (DSHB, 1/20), mouse anti-Ey ([91], 1/20), mouse anti-Eya (DSHB, 1/20), rabbit anti-GFP (Invitrogen, 1/1000), guinea pig anti-Hth ([92], 1/100), mouse anti-Mmp1 (DSHB, 1/20), guinea pig anti-Sens ([93], 1/1000), rabbit anti-Tai ([84], 1/500), rabbit anti-Tsh ([94], 1/2000). Secondary antibodies used were; anti-mouse/rat Alexa647 (Invitrogen) and anti- rabbit Alexa488 (Invitrogen) at 1/400 dilution. F-actin was detected with phalloidin–tetramethylrhodamine isothioblueate (TRITC; Sigma, 0.3 µM, 1/1000). Samples were mounted in 80% glycerol.

### Microscopy and image processing

All samples were analysed by confocal microscopy on an Olympus FV1000 or Leica TCS SP5 microscope. Single optical sections were selected in FluoView software before being processed in Adobe Photoshop CS2 and assembled into figures in Adobe Illustrator CS2.

### Expression array, ChIP-Seq and bioinformatic analysis

Eye/antennal discs were dissected from ∼5 day old larvae bearing *ab*-expressing clones, *scrib^1^*+*ab*-expressing clones, or *FRT82B* control clones. For the expression array, 20 pairs of discs for the *ab* and *scrib^−^*+*ab* samples, and 50 pairs of discs for the control *FRT82B* genotype, were used to prepare RNA. Samples were prepared in triplicate, and the RNA isolated using TRIZOL, before being column purified (Qiagen). Probes were hybridised to GeneChip *Drosophila* 2.0 Genome Arrays (Affymetrix).

For ChIP-Seq, eye/antennal discs were dissected and samples cross-linked in a 1.8% formaldehyde solution on a rotating wheel for 5 mins, prior to DNA being sheared by sonication (15 cycles of 20 seconds, 35% amplitude) to produce fragments of ∼500 bp [95]. 100 µl of extract (corresponding to ∼100 discs) was used for each immunoprecipitation. 35 µl of 50% Protein A-Sepharose CL4B was added to each sample, and cleared after 1.5 hours incubation. 2 µl of polyclonal rabbit anti-Abrupt antibody [17] was then added per sample (or, for the input DNA controls, no antibody was added), and incubated overnight with rotation. Immunocomplexes were recovered by adding 35 µl Protein A-Sepharose to each sample, incubating for 3 hours at 4°C, and then harvesting by centrifugation. Chromatin was decrosslinked by RNase and Proteinase K incubations, and the DNA column purified (Qiagen). For each sequencing sample, 3 to 6 immunoprecipitations were pooled.

High throughput sequencing was performed for the ChIP-Seq from the *ab*-expressing (8,643,591 reads) and *scrib^−^*+*ab* (8,508,640 reads) samples. ChIP-Seq reads in all cases were aligned to the *Drosophila* genome (dm3 genome assembly, BDGP Release 5) using Bowtie with default parameters [96]. Correlation between ChIP-Seq experiments was computed with UCSC Table browser [97]. We used PeakSeq [98] to identify the regions significantly enriched on ChIP-Seq reads from each sample in comparison to the normalised input control (READLENGTH = 40, MAXGAP = 40, MINFDR = 0.01 and PVALTHRESH = 0.0001). The resulting read maps and target lists were visualised as custom tracks in the University of California Santa Cruz (UCSC) Genome Browser [97]. Using RefSeq [99], potential Ab target genes in each ChIP-Seq experiment were identified by the presence of significant peaks either within the promoter region (from the transcriptional start sites to 500 bp upstream) or within the introns of each gene. Expression arrays, ChIP-Seq profiles and target regions were deposited in the National Center for Biotechnology Information (NCBI) Gene Expression Omnibus (GEO) repository as CEL, wiggle (WIG) and Browser Extensible Data (BED) files, respectively, under the accession numbers GSE42938 (expression arrays) and GSE42928 (ChIP-Seq profiles and target regions). Gene Ontology (GO) enrichments were identified with the “GO Term Enrichment” v1.8 from AmiGo. To annotate the list of putative transcription factor binding sites on each set of ChIP-Seq binding regions (*ab* and *scrib* ^−^+*ab*), we used the MatScan program [100] with the full collection of 827 predictive matrices available in Jaspar and Transfac [101], [102]. We ranked each matrix on the basis of the number of hits in both experiments, normalizing with the total size for each set of sequences and the number of occurrences identified in the whole genome. To build the list of overrepresented matrices, we selected those models that presented a difference in each ranking of at least 200 positions and a positive normalised fold-change value. To identify a potential DNA recognition sequence for Ab binding we focussed upon peaks with a height of 40 or more reads from the ChIP-Seq profile of the *ab* overexpression experiment (irrespective of its genomic location), and used MEME [103] to identify enriched motifs. Potential transcription factors capable of recognizing enriched motifs were identified using the TOMTOM program (MEME suite).

### Quantitative RT-PCR

Total RNA for each genotype was prepared in replicates using TRIZOL and column purification (Qiagen). After cDNA synthesis, qRT-PCR on each replicate was assayed in triplicate using StepOnePlus Real Time PCR System (Applied Biosystems). For the expression array validations, we were unable to use standard house keeping mRNAs to normalise the results since many, such as actin and GAPDH, were changed in the expression profiling. Instead, the expression levels were normalised with respect to *CG6044*, which is expressed in the eye/antennal disc but was not significantly deregulated in the expression arrays, and the average for the triplicates determined. The following primers were used: *Blimp-1*, forward TTGCGACAAGAAGTACATCAG, reverse GATGGTCTTTATCCAAACACTC; *bab2*, forward CAAGTTCGACATACCCATTCC, reverse GATATAGGTACCATGACCCTG; *CG6044*, forward ACTCAGCTTCCTCTACTTCC, reverse CGCAATACTAAAGCAATCACAC; *Eip78C*, forward CTAATAAAGCTGGGCTTCTTCG, reverse GTTGACAAAGTCAGAATCGTAGAG; *fru*, forward TCAGATACTCAGAGATGCGA, reverse TGTTGTTATCTGTGAGACCA; *H15*, forward GTGACTTTGATAGGGATCCCA, reverse AGGAGTCAATTGGGACATCAG. The fold changes for each sample were determined using the 2(−Delta Delta C(T)) method [104].

For the ChIP validation of a selection of representative genes, chromatin was immunoprecipitated with the Ab antibody from *ab* and *scrib^−^*+*ab* samples (as described above), and then used for quantitative real-time PCR, with rabbit IgG immunoprecipitation as a control. Primers were designed around peak regions identified from ChIP-Seq analysis by using PerlPrimer application. The following primers were used: *Antp*, forward AGGATCACCTATTTAACTGGAC, reverse ATGTACGTGGCATACTTTCAG; *Blimp-1*, forward CAAGAACCTGAGACACCTGA, reverse CAAGAACCTGAGACACCTGA; *br*, forward ACACATTCGCAACCAACAAT, reverse CCCTTCCAGTACCCTACTCT; *Buffy*, forward GGGATACATTCACCTTATATGCAC, reverse ACCGAAGTTGAAGTAAGCGA; *chinmo*, forward CATCTTCAACTTCCTTGCTAA, reverse TGAATACGAAATTGAGCGAA; *eya*, forward CACAGACAACACTCGAATCAG, reverse GCAGCAGAAGAGACAAAGAG; *fru*, forward GCTCTTCCATTATCGTTCTC, reverse TATACATGTGAATAGGGCAAG: *ftz-f1*, forward AGAGATACGAGTATCCGAGTG, reverse GACATGCACATACATATAGACGG; *HLHmβ*, forward CCTCCCTCCTTATGTATGTG, reverse GCACAATCAGAAGAAGTCAG; *Mmp1*, forward GGATAAGTGCCTATTACTAGCTG, reverse GAATAGCTTATTAGCACGGGTC.

## Supporting Information

Dataset S1 Lists of differentially expressed probe sets, identified ChIP-Seq target genes, and genes within Classes 1 to 6. **Sheet 1.** Probe sets deregulated in *ab* and *scrib^1^*+*ab* mosaic discs (compared to control *FRT82B* discs). Out of 18952 probe sets, 4239 were differentially expressed (log base 2 fold change (logFC) >1, adjusted p value <0.05) within *ab* and *scrib^1^*+*ab* mosaic discs. In *ab*-expressing discs, 3028 probe sets were deregulated, comprising 705 probe sets (pattern 3) unique to *ab*-expressing discs and 2323 probe sets (pattern 4) shared with *scrib^1^*+*ab* discs. In *scrib^1^*+*ab* mosaic discs, 3534 probe sets were deregulated, comprising 1211 unique to *scrib^1^*+*ab* (pattern 2), together with the 2323 shared probe sets. 14713 probe sets (pattern 1) were not significantly deregulated in either genotype. **Sheet 2.** Genes identified from ChIP-Seq peak enrichments from *ab* and *scrib^1^*+*ab* mosaic discs. 557 genes (pattern 2) were unique to *ab*; 721 genes (pattern 3) were unique to *scrib^1^*+*ab*; and 2025 genes (pattern 1) were shared by *ab* and *scrib^1^*+*ab* samples. **Sheets 3–8.** Gene lists of classes 1–6 (see **Figure 2C** for a Venn diagram depicting the different classes).
(XLS)

Dataset S2 List of the 183 differentially expressed genes in *ab* or *scrib* ^−^+*ab* mosaic eye discs compared to control discs, that are represented by more than one probe set. Of the 3549 annotated genes deregulated in *ab* and *scrib^1^*+*ab* mosaic eye discs, 183 were represented by more than one probe set. Individual probe sets showing upregulation or downregulation (log base 2 fold change >1, adjusted p value <0.05) in either *ab* or *scrib^1^*+*ab* mosaic eye discs compared to control *FRT82B* discs are indicated by “+1” or “−1” respectively. A “0” indicates no significant deregulation. The 59 genes that are represented by probes with conflicting expression (i.e. with probe sets showing opposite regulation in a particular genotype) are shown in red. The reasons for conflicting expression are not known, but could indicate the existence of more than one differentially regulated transcript.
(PDF)

Dataset S3 GO enrichments (p<0.01) for differentially expressed genes, ChIP-Seq candidate genes and Classes 1, 2 and 5. **Sheets 1–3.** GO enrichments for differentially expressed genes unique to *ab* overexpression (“Abrupt Not Scrib”), unique to *scrib^1^*+*ab* (“Scrib Not Abrupt”), and shared between the two samples (“Abrupt and Scrib”). For the analysis, deregulated genes were identified from probe sets that could be assigned FlyBase Gene IDs. For genes with multiple deregulated probe sets, each FlyBase Gene ID was used only once per enrichment analysis. **Sheets 3–6.** GO enrichments for potential Ab target genes, identified by ChIP-Seq, unique to *ab* overexpression (“Abrupt Not Scrib”), unique to *scrib^1^*+*ab* (Scrib Not Abrupt”), and shared between the two samples (“Abrupt and Scrib”). **Sheets 7–9.** GO enrichments for genes within Classes 1, 2 and 5 (see **Figure 2C** for a Venn diagram depicting the different classes). Classes 3, 4 and 6 exhibited no significant GO enrichments.
(XLS)

Dataset S4 ChIP-Seq peaks aligned to the genome for selected genes within Classes 1 to 4. Only genes depicted in **Figure 2E** are shown. Genes are in alphabetical order, and highlight bars beneath the peak landscape indicate significant peaks in each genotype.
(PDF)

Figure S1 Overexpression phenotypes of confirmed *scrib* ^−^ interactors in eye/antennal disc clones. Mosaic eye/antennal discs (anterior to the left in this and all subsequent figures) generated with *ey-FLP* and taken from larvae ∼5 days AEL. Clones are positively marked by GFP (white, or green in merges), tissue morphology is shown by F-actin (red in merges), and cell fate by Elav expression (white or pale blue, changing to magenta or dark blue when overlaid with GFP). GFP (panels A–J), GFP/Elav merges (panels A′,B″,C′,D″,E′,F″,G′,H″,I′J″), F-actin (B′,D′,F′,H′,J′) and GFP/Elav/F-actin merges (panels A″,B″′,C″,D″′,E″,F″′,G″,H″′,I″J″′). (A,B) *dl*-expressing clones and mosaic discs are overgrown, especially within the antennal region (A). The expression of *dl* in *scrib^1^* clones also promotes overgrowth within the antennal region, and in the eye disc, clonal tissue also overgrows and does not express Elav (B). (C,D) *esg*-expressing clones are not overgrown (C), however, the expression of *esg* in *scrib^1^* clones promotes large overgrowths especially within the antennal region (D). (E,F) *chn*-expressing clones are not overgrown (E), however, the expression of *chn* in *scrib^1^* clones promotes antennal disc overgrowth, as well as overgrowth of eye disc tissue that does not express Elav (F, arrow). (G,H) *apt*-expressing clones are not overgrown (G), however, the expression of *apt* in *scrib^1^* clones promotes mild clonal overgrowth although differentiation is not completely blocked (H). (I,J) Neither *numb*-expressing clones (I), nor *scrib^1^* clones expressing *numb*, are overgrown (J). Yellow scale bar = 50 µm.
(JPG)

Figure S2 Proliferation and apoptosis in *scrib^−^*+*ab* tumours. *ey-FLP* induced eye/antennal disc clones marked by GFP (green). EdU (A–C) labeling is white (and magenta when overlayed with GFP in merged images), and TUNEL (D–F) is white (red in merged images, and appears yellow when overlayed with GFP in merged images). Arrowheads in A,B indicate the second mitotic wave. EdU (panel C), GFP/EdU merges (panels A,B,C′), GFP/TUNEL merges (panel D,E,F′), and TUNEL (panel F). (A,D) Wild type mosaic discs show the normal pattern of cell proliferation (A) and cell death (D). (B,E) *ab* overexpressing eye disc clones do not ectopically proliferate (B), but induce increased cell death in wild type cells along the clonal borders (E). (C,F) *scrib^1^+ab* clones ectopically proliferate, and disrupt the normal pattern of cell proliferation in the eye disc (C), and induce increased cell death in surrounding wild type tissue (F, arrowhead). Yellow scale bar = 50 µm.
(JPG)

Figure S3 Validation of the expression array by quantitative real-time PCR of selected genes. Expression levels, as determined by quantitative real-time PCR (see Materials and Methods), are shown for 5 genes (*Eip78C*, *bab2*, *H15*, *Blimp-1*, *fru*) in *ab*-expressing discs and in *scrib^1^*+*ab* discs, compared to the expression level in control discs containing wild type *FRT82B* clones (assigned an expression level of 1). The expression levels of *CG6044*, a gene that is expressed in the eye/antennal disc but did not significantly change in expression across the arrays, were used for normalisation. All five genes confirm the results from the expression array, with *Eip78C*, *bab2*, *H15* and *Blimp-1* being repressed in *ab*-expressing discs, and even further repressed in *scrib^1^*+*ab* tumours, whilst *fru* expression is increased upon *ab* overexpression, and even further increased in *scrib^1^*+*ab* tumours. ANOVA was performed for each primer pair; * p<0.01, ** p<0.001, *** p<0.0001 compared to the *FRT82B* control (n = 3). The changes in expression between *ab* and *scrib^−^*+*ab* samples were also all highly significant (p<0.0001). Error bars indicate 1 s.d.
(JPG)

Figure S4 The Ab antibody shows high specificity. Mosaic eye/antennal disc clones generated with *ey-FLP*. Clones are positively marked by GFP (white or green in merges), and Ab protein levels are shown in white (magenta when overlaid with GFP in the merges). Ab (panels A,B), GFP (panel A′B′) and merges (panels A″,B″). Tissue morphology in B is shown by F-actin (white, panel B″′). (A) Ab is endogenously expressed in the antennal disc, the anterior portion of the eye disc and the peripodial membrane, except in *ab^1D^* mutant clones, which show greatly reduced levels of Ab. (B) Clones of tissue overexpressing *ab* show greatly elevated levels of Ab protein. Yellow scale bar = 50 µm.
(JPG)

Figure S5 ChIP validation for selected representative genes. Chromatin was immunoprecipitated with the Ab antibody from *ab* and *scrib* ^−^+*ab* mosaic eye/antennal discs, and then used for quantitative real-time PCR. Fold enrichment for all target genes was determined compared to rabbit IgG control immunoprecipitations, which were assigned an expression level of 1 (represented by the line on the graph). Representative genes were included from most functional categories; ecdysone response (*Blimp-1*, *ftz-F1*), BTB-ZF (*br*, *chinmo*, *fru*), cell fate (*Antp*, *eya*), Notch signalling (*HLHmβ*), cell death/survival (*Buffy*) and JNK signaling (*Mmp1*). Enrichment of all genes was observed in the Ab antibody immunoprecipitation compared to the IgG control from *ab* and *scrib^−^*+*ab* mosaic eye/antennal imaginal discs. T-test comparing each Ab immunoprecipitation to the IgG control; * p<0.01, ** p<0.001, *** p<0.0001 (n = 3 for both Ab immunoprecipitation and the IgG control). Error bars indicate 1 s.d.
(JPG)

Figure S6 Enriched sequence motifs amongst the top peaks from the *ab* alone overexpression sample. The strongest peaks were selected with a height of 40 or more reads from the ChIP-Seq profile, resulting in 1629 regions with an average length of 84.8 bp. Enriched motifs were identified using MEME, and potential transcription factors capable of recognizing the enriched motifs were identified using the TOMTOM program (MEME suite).
(JPG)

Figure S7 Br is repressed in *ab*-expressing cells. Mosaic eye/antennal disc clones generated with *ey-FLP*. Clones are positively marked by GFP (white, or green in merges), and tissue morphology is shown by F-actin (white). Br levels are shown in white (magenta when overlaid with GFP in the merges). GFP (panels A–C), F-actin (panels A′–C′), Br (panels A″–C″), and merges (panels A″′–C″′). (A) Br is expressed throughout the eye/antennal disc and peripodial membrane. (B,C) Br is repressed in *ab*-expressing clones (B, arrowheads) and in *scrib^1^*+*ab* clones (C, arrowheads). Yellow scale bar = 50 µm.
(JPG)

Figure S8 Overexpression of *tai^ΔB^* in *scrib^−^*+*ab* tumours prevents tumour overgrowth. Mosaic eye/antennal disc clones generated with *ey-FLP*. Clones are positively marked by GFP (white, or green in merges), tissue morphology is shown by F-actin (red in merges), and cell fate by Elav expression (white or pale blue, changing to magenta or dark blue when overlaid with GFP). GFP (panels A–C), F-actin (panels A′–C′), Elav (panels A″–C″), GFP/Elav merges (panels A″′–C″′) and GFP/Elav/F-actin merges (panels A″″–C″″). (A) Expression of *tai^ΔB^* in clones results in small clones. (B,C) Expression of *tai^ΔB^* in *scrib^1^* clones (B) or *scrib^1^*+*ab* clones (C) reduces clonal overgrowth and results in the eclosion of adult flies (data not shown).
(JPG)

Figure S9 Eya, Sens and Ato expression are reduced in *scrib* ^−^+*ab* tumours. *ey-FLP* induced eye/antennal disc clones at ∼5 days AEL. Clones are marked by GFP (white, or green in merges), and cell fate is shown by the expression of Eya, Sens and Ato (all white, and magenta when overlaid with GFP in the merges) in control clones (A,E,I), *scrib^1^* clones (B,F,J), *ab*-expressing clones (C,G,K), and *scrib^1^*+*ab* clones (D,H,L). GFP (panels A–L), Eya (panels A′–D′), Sens (panels E′–H′), Ato (panels I′–L′), and merges (panels A″–L″). (A–D) Eya expression is not altered in *scrib^1^* clones (B, arrowhead), or *ab* overexpressing clones (C, arrowhead). *scrib^1^*+*ab* clones have greatly reduced levels of Eya (D, arrowhead). (E–H) Sens expression is disrupted and reduced in *scrib^1^* clones, and repressed in *ab* overexpressing clones in the antennal but not in the eye disc (G, arrowheads). *scrib^1^*+*ab* clones do not express Sens (H, arrowhead). (I–L) Ato expression is disrupted and slightly reduced in *scrib^1^* clones (J, arrowhead), and repressed in *ab* overexpressing clones within the antennal disc (K, arrowhead), and reduced in eye disc clones (K, arrow). *scrib^1^*+*ab* clones do not express Ato (L, arrowhead). Yellow scale bar = 50 µm.
(JPG)

Figure S10 Tsh and Ey expression is not substantially altered in *scrib* ^−^+*ab* tumours, however, Bab2 and Ct expression is reduced. *ey-FLP* induced eye/antennal disc clones at ∼5 days AEL. Clones are marked by GFP (white, or green in merges), and cell fate is shown by the expression of Tsh, Ey, Bab2 and Ato (all white, and magenta when overlaid with GFP in the merges) in control clones (A,E,I,M), *scrib^1^* clones (B,F,J,N), *ab*-expressing clones (C,G,K,O), and *scrib^1^*+*ab* clones (D,H,L,P). GFP (panels A–P), Tsh (panels A′–D′), Ey (panels E′–H′), Bab2 (panels I′–L′), Ct (panels M′–P′), and merges (panels A″–P″). (A–D) Tsh expression exhibits only slight perturbations in *scrib^1^* clones, sometimes extending more posteriorly within large mutant clones spanning the normal expression domain (B), and is slightly increased in *ab* overexpressing clones (C, arrowhead). *scrib^1^*+*ab* clones express Tsh in the anterior portion of the eye disc, although it is repressed more posteriorly, as in control clones (D, arrowhead). (E–H) Ey expression is slightly reduced in *scrib^1^* clones (F, arrowhead), and *ab* overexpressing clones (G, arrowhead). *scrib^1^*+*ab* clones express Ey in the anterior portion of the eye disc, although its expression is repressed, as it is in control clones, more posteriorly (H, arrow). (I–L) Bab2 expression is not altered in *scrib^1^* clones (J), and although expanded, or ectopic, domains of Bab2 expression are sometimes associated with *ab* overexpressing clones in the antenna, the levels of Bab2 within the clones are slightly reduced compared to adjacent wild type tissue (K; arrowhead showing slightly enlarged Bab2 domain of expression, although levels of Bab2 in the *ab*-expressing clone are lower than the more highly expressing wild type tissue adjacent to the clone). *scrib^1^*+*ab* clones do not express Bab2 (L, arrowhead). (M–P) Ct expression is not altered in *scrib^1^* clones (N), and is repressed in more distally located *ab* overexpressing clones (O, arrowhead), although expression is unaffected in the proximal region (O, arrow). *scrib^1^*+*ab* clones do not express Ct (P, arrowheads). Yellow scale bar = 50 µm.
(JPG)

Figure S11 Hth is not sufficient or required for Ab-mediated tumour overgrowth. *ey-FLP* induced eye/antennal disc clones at 5 days AEL. Clones are marked by GFP (white, or green in merges), and Elav is shown in white (blue in merges, and dark blue when overlaid with GFP). GFP (panels A–D), Elav (panels A′–D′), and merges (panels A″–D″). (A) *hth* overexpressing clones do not express Elav. (B) Overexpressing *hth* in *scrib^1^* clones blocks Elav expression and promotes clonal overgrowth, however, the larvae pupate and do not undergo an extended larval stage of development. (C) *scrib^1^ hth^P2^* mutant clones are similar to *hth^P2^* mutant clones alone (see **Figure 5B**), although photoreceptor differentiation is disrupted in posteriorly localised clones. (D) *hth^P2^* mutant clones overexpressing *ab* are similar to *hth^P2^* mutant clones (see **Figure 5B**).
(JPG)

Figure S12 *ab* overexpression downregulates DIAP1 levels. Mosaic antennal disc clones (A) and eye disc clones (B) overexpressing *ab*, and positively marked by GFP (white, or green in the merges). Diap1 is shown in white (or red in merges). GFP (panels A,B), Diap1 (panels A′B′), and merges (panels A″,B″). (A,B) *ab*-overexpressing clones downregulate DIAP1 (red in merges), in both the antennal disc (A) and eye disc (B). Yellow scale bar = 50 µm.
(JPG)

Figure S13 Overexpression of *dac* does not restrain *scrib* ^−^+*ab* tumour overgrowth. *ey-FLP* clones are positively marked by GFP (white, or green in merges) in all panels. Elav is shown in white (magenta when overlaid with GFP in the merges). Yellow lines indicate the positions of virtual cross sections (shown on the right). GFP (panels A–D), Elav (panels A′–D′), merges (panels A″–D″), and virtual cross sections of the merges (panels A″′–D”′). (A) Overexpressing *dac* in mosaic discs results in small cyst-like clones that disrupt the normal pattern of photoreceptor differentiation. (B) Overexpressing *dac* in *scrib^1^* mutant clones produces a similar phenotype as *scrib^1^* clones alone (the mutant clone failing to express Elav is located above the disc proper). (C) Overexpressing *ab* and *dac* together in clones generates large clones in antenna, similar to *ab* overexpressing clones alone, but very small clones in the eye disc. (D) Overexpressing *dac* in *scrib^1^*+*ab* clones does not restrain clone overgrowth, and large neoplastic tumours are produced, which do not express Elav. Yellow scale bar = 50 µm.
(JPG)

Figure S14 Knockdown of *yki* in *scrib* ^−^+*ab* tumours does not prevent cell migration between the brain lobes. Eye/antennal discs still attached to the brain lobes (BL) containing *ey-FLP* induced *scrib^1^* clones that express both *ab* and *yki^RNAi^* (A, and a higher magnification of the region between the brain lobes in B). *ey-FLP* clones are positively marked by GFP (white, or green in merges) and F-actin is shown in white (red in the merges). Elav is shown in blue in the merges. GFP (panels A,B), F-actin (panels A′,B′), and GFP/F-actin/Elav merges (panels A″,B″). Mutant tissue overgrowth is restrained compared with *scrib^−^*+*ab* tumours, however tissue is still observed between the brain lobes (arrows), consistent with it migrating and merging with the brain lobes. Yellow scale bar = 50 µm.
(JPG)

Figure S15 Expression of *aPKC^DN^* in *scrib* ^−^+*ab* tumours restrains tumour overgrowth. *ey-FLP* clones at day 5 AEL (A–C), and with brain lobes (BL) attached at day 7 (D). Mutant clones are positively marked by GFP (white, or green in merges). Elav is shown in white (magenta when overlaid with GFP in the merges), and cell morphology is indicated by F-actin (white). GFP (panels A–D), F-actin (panels A′–D′), Elav (panels A″–D″) and GFP/Elav merges (panels A″′–D′″). (A) Overexpressing *aPKC^CAAX-DN^* in clones does not produce a discernible phenotype. (B) Clones co-overexpressing *aPKC^CAAX-DN^* and *ab* are similar to *ab*-expressing clones alone (compare to **Figure 1E**). (C,D) Overexpressing *aPKC^CAAX-DN^* in *scrib^1^*+*ab* clones restrains clonal tissue overgrowth and GFP specks are observed, consistent with cells undergoing cell death (C). Larvae still enter an extended larval stage of development, however, tumour overgrowth (D) is reduced compared to *scrib^1^*+*ab* tumours (eg. **Figure 8F**). Yellow scale bar = 50 µm.
(JPG)

Figure S16 *wts^X1^*+*ab* tumours retain Dll expression, but do not express Dan. *ey-FLP* induced eye/antennal disc clones marked by GFP (white, or green in merges). The cell fate markers Dll and Dan are shown in white (magenta when overlaid with GFP in merged images). F-actin (white) shows cell morphology. GFP (panels A–D), F-actin (panels A′–D′), Dll (panels A″,B″), Dan (panels C″,D″), GFP/Dll merges (panels A″′,B″′) and GFP/Dan merges (panels C″′,D′″). (A) *wts^X1^* clones exhibit the normal pattern of Dll in the eye/antennal disc. (B) *wts^X1^*+*ab* clones retain Dll expression, often resulting in ectopic domains of Dll-expressing tissue (B, arrowhead), similar to *ab* expressing clones, or *scrib^1^*+*ab* tumours. (C) *wts^X1^* clones exhibit the normal pattern of Dan in the eye/antennal disc. (D) *wts^X1^*+*ab* clones do not express Dan in the antenna, and the overgrowths in the eye disc are also characterised by a loss of Dan (D, arrowhead). Yellow scale bar = 50 µm.
(JPG)

Movie S1 Live cell imaging of *scrib* ^−^+*ab* tumours. Movie taken at 2 minute intervals over a 20 hour period at 10× magnification. *scrib^1^*+*ab* tumour cells are highly motile, moving between, and over, the brain lobes. Note that, although motile hemocytes have been reported be associated with the tumours, and may be associated with tumour cell engulfment, the intensity and distribution of GFP fluorescence within the motile cells is not consistent with them being phagocytic hemocytes.
(ASF)

Movie S2 Live cell imaging of *scrib* ^−^+*ab*+*bsk^DN^* tumours. Movie taken at 2 minute intervals over a 20 hour period at 10× magnification. *scrib^1^*+*ab*+*bsk^DN^* tumour cells are non invasive, and the overall tumour mass, whilst growing, remains compact.
(ASF)

Table S1 Transcription factor matrices enriched amongst peak sequences associated with the promoter regions or introns of potential target genes.
(DOC)
