# Supplementary material for: The BTB-zinc Finger Transcription Factor Abrupt Acts as an Epithelial Oncogene in Drosophila melanogaster through Maintaining a Progenitor-like Cell State
Source: PLoS Genet. 2013 Jul 18;9(7):e1003627. doi: 10.1371/journal.pgen.1003627 (PMC3715428; doi:10.1371/journal.pgen.1003627)
Supplement: Dataset S2 — List of the 183 differentially expressed genes in ab or scrib −+ab mosaic eye discs compared to control discs, that are represented by more than one probe set. Of the 3549 annotated genes deregulated in ab and scrib1+ab mosaic eye discs, 183 were represented by more than one probe set. Individual probe sets showing upregulation or downregulation (log base 2 fold change >1, adjusted p value <0.05) in either ab or scrib1+ab mosaic eye discs compared to control FRT82B discs are indicated by “+1” or “−1” respectively. A “0” indicates no significant deregulation. The 59 genes that are represented by probes with conflicting expression (i.e. with probe sets showing opposite regulation in a particular genotype) are shown in red. The reasons for conflicting expression are not known, but could indicate the existence of more than one differentially regulated transcript. (PDF) [file pgen.1003627.s002.pdf]

Alh

	Ab.vs.FRT	AbSCRIB.vs.FRT	Alignments
1623218_s_at	-1	-1	chr3R:2923389-2949907 // 100.0
1630415_at	1	0	chr3R:2920321-2934139 // 100.0

Aly

	Ab.vs.FRT	AbSCRIB.vs.FRT	Alignments
1630198_at	-1	-1	chr3R:2905385-2906126 // 100.0
1634966_a_at	-1	-1	chr3R:2905385-2906126 // 100.0
1640178_at	0	0	chr3R:2903617-2906126 // 100.0

Ank2

	Ab.vs.FRT	AbSCRIB.vs.FRT	Alignments
1623451_at	0	0	chr3L:7710448-7711439 // 100.0
1631662_a_at	-1	-1	chr3L:7649589-7657189 // 100.0
1633313_at	0	-1	chr3L:7648488-7690761 // 99.87
1640337_a_at	-1	-1	chr3L:7697917-7706720 // 99.89

B52

	Ab.vs.FRT	AbSCRIB.vs.FRT	Alignments
1624547_s_at	-1	-1	chr3R:9487021-9489304 // 100.0
1628399_s_at	-1	-1	chr3R:9487021-9492617 // 100.0
1631082_at	0	0	chr3R:9487021-9488913 // 100.0
1632447_at	0	-1	chr3R:9487021-9489304 // 100.0
1633821_at	0	1	chr3R:9487021-9492617 // 100.0

bbg

	Ab.vs.FRT	AbSCRIB.vs.FRT	Alignments
1630921_at	0	-1	chr3L:14500705-14506348 // 100.0
1636947_at	-1	-1	chr3L:14405922-14410432 // 100.0

beat-IIIc

	Ab.vs.FRT	AbSCRIB.vs.FRT	Alignments
1625349_at	0	-1	chr2L:17189809-17260732 // 100.0
1633927_a_at	-1	-1	chr2L:17189829-17260732 // 98.86

br

	Ab.vs.FRT	AbSCRIB.vs.FRT	Alignments
1626996_s_at	0	-1	chrX:1504544-1543426 // 99.9
1629683_at	-1	-1	chrX:1506373-1553964 // 100.0
1635691_at	0	0	chrX:1518170-1555575 // 100.0
1636931_at	-1	-1	chrX:1506373-1549402 // 100.0
1637312_a_at	0	0	chrX:1506373-1539198 // 100.0

btsz

	Ab.vs.FRT	AbSCRIB.vs.FRT	Alignments
1624207_at	-1	-1	chr3R:10632901-10635844 // 100.0
1631518_at	-1	-1	chr3R:10639720-10679621 // 100.0

CalpA

	Ab.vs.FRT	AbSCRIB.vs.FRT	Alignments
1628628_at	0	1	chr2R:15312577-15318068 // 100.0
1633524_a_at	-1	0	chr2R:15312577-15316555 // 100.0
1638939_at	-1	0	chr2R:15312577-15316555 // 100.0

caps

	Ab.vs.FRT	AbSCRIB.vs.FRT	Alignments
1632508_s_at	-1	-1	chr3L:13221788-13270860 // 100.0
1638580_at	-1	-1	chr3L:13270415-13271131 // 100.0

Cbp80

	Ab.vs.FRT	AbSCRIB.vs.FRT	Alignments
1623101_at	1	0	chrX:4544053-4552292 // 100.0
1636071_a_at	0	-1	chrX:4544053-4552041 // 100.0

CG10344

	Ab.vs.FRT	AbSCRIB.vs.FRT	Alignments
1622933_at	1	0	chr2R:18313255-18314459 // 99.65
1634822_a_at	1	1	chr2R:18313236-18314470 // 100.0

CG10874

	Ab.vs.FRT	AbSCRIB.vs.FRT	Alignments
1633055_at	0	-1	chr2L:2189912-2192372 // 100.0
1638122_a_at	0	-1	chr2L:2190706-2192372 // 100.0

CG11727 /// DsimCG11727

	Ab.vs.FRT	AbSCRIB.vs.FRT	Alignments
1626533_a_at	-1	-1	chrX:11328690-11348335 // 99.87
1638996_at	0	1	chrX:11326944-11348335 // 100.0

CG11905

	Ab.vs.FRT	AbSCRIB.vs.FRT	Alignments
1632514_s_at	1	0	chr3L:16924267-16928632 // 100.0
1637724_s_at	1	0	chr3L:16923216-16928632 // 100.0
1638986_s_at	0	-1	chr3L:16922056-16928632 // 100.0

CG12054

	Ab.vs.FRT	AbSCRIB.vs.FRT	Alignments
1628688_at	-1	-1	chr3R:27046425-27054213 // 100.0

1637162_at	1	1	chr3R:27054418-27054846 // 100.0

CG12038

	Ab.vs.FRT	AbSCRIB.vs.FRT	Alignments
1627417_s_at	0	-1	chr3L:994005-995549 // 100.0
1636312_at	1	0	chr3L:993991-995711 // 100.0

CG12250

	Ab.vs.FRT	AbSCRIB.vs.FRT	Alignments
1629978_at	1	1	chr3R:21418906-21434850 // 100.0
1637790_a_at	0	0	chr3R:21417854-21434850 // 100.0
1640067_at	1	0	chr3R:21417854-21434850 // 100.0

CG13722

	Ab.vs.FRT	AbSCRIB.vs.FRT	Alignments
1625261_x_at	-1	0	chr3L:4485408-4488013 // 100.0
1625282_at	-1	0	chr3L:4485408-4488013 // 100.0

CG13737

	Ab.vs.FRT	AbSCRIB.vs.FRT	Alignments
1634287_at	1	-1	chr3L:13872095-13876136 // 100.0
1636750_s_at	1	-1	chr3L:13872095-13876136 // 100.0

CG14408

	Ab.vs.FRT	AbSCRIB.vs.FRT	Alignments
1625335_at	1	1	chrX:14722783-14726090 // 100.0
1628948_a_at	1	1	chrX:14722783-14726090 // 100.0
1634311_at	1	1	chrX:14721086-14724504 // 100.0

CG15059

	Ab.vs.FRT	AbSCRIB.vs.FRT	Alignments
1628534_at	0	1	chrX:18152866-18153489 // 100.0
1638767_at	0	0	chrX:18152974-18153481 // 100.0
1638769_a_at	0	1	chrX:18152866-18153489 // 100.0

CG15744

	Ab.vs.FRT	AbSCRIB.vs.FRT	Alignments
1634342_s_at	0	-1	chrX:13182395-13187911 // 100.0
1639266_at	0	-1	chrX:13185615-13188469 // 100.0

CG1637

	Ab.vs.FRT	AbSCRIB.vs.FRT	Alignments
1623285_at	0	0	chrX:10764614-10771840 // 100.0
1624797_at	0	-1	chrX:10766373-10771840 // 100.0

1633563_at	-1	0	chrX:10768506-10771840 // 100.0

CG16885

	Ab.vs.FRT	AbSCRIB.vs.FRT	Alignments
1631302_at	-1	-1	chr2L:13932558-13934691 // 99.11
1638475_a_at	0	-1	chr2L:13932789-13934691 // 100.0

CG17124

	Ab.vs.FRT	AbSCRIB.vs.FRT	Alignments
1633795_a_at	0	1	chr2L:10745114-10756316 // 100.0
1640465_at	0	1	chr2L:10744074-10756316 // 100.0

CG17754

	Ab.vs.FRT	AbSCRIB.vs.FRT	Alignments
1628712_at	1	0	chrX:9144459-9149853 // 100.0
1637504_at	0	-1	chrX:9144459-9149075 // 99.75
1638327_a_at	-1	-1	chrX:9144459-9149853 // 100.0

CG17816

	Ab.vs.FRT	AbSCRIB.vs.FRT	Alignments
1637250_at	1	0	chr3R:3683895-3685159 // 100.0
1638984_s_at	1	0	chr3R:3686683-3712454 // 99.5

CG17834

	Ab.vs.FRT	AbSCRIB.vs.FRT	Alignments
1626011_at	-1	0	chr2L:8535800-8537932 // 100.0
1630097_s_at	-1	-1	chr2L:8529142-8541462 // 100.0

CG1969

	Ab.vs.FRT	AbSCRIB.vs.FRT	Alignments
1626181_at	0	0	chr3R:25565814-25566470 // 100.0
1636543_a_at	-1	-1	chr3R:25565814-25566470 // 100.0
1637778_a_at	0	-1	chr3R:25565565-25567631 // 100.0

CG30080

	Ab.vs.FRT	AbSCRIB.vs.FRT	Alignments
1627759_at	0	-1	chr2R:11586223-11589199 // 100.0
1628387_s_at	0	-1	chr2R:11586520-11586658 // 100.0

CG31001

	Ab.vs.FRT	AbSCRIB.vs.FRT	Alignments
1624737_at	1	1	chrUextra:5186009-5186854 // 61.07 /// chrUextra:5186109-5186897 // 56.11 /// chr3R:27806689-27808022 // 100.0 ///

			chr3R:27806123-27809152 // 98.8 ///<br>chr3R:27807843-27810289 // 93.25 ///<br>chr3R:27805810-27807414 // 75.02 ///<br>chr3R:27805778-27806610 // 59.79 ///<br>chrUextra:23175062-23175709 // 46.36
1628230_x_at	1	1	chr3R:27806689-27808022 // 100.0 ///<br>chr3R:27806123-27809152 // 98.8 ///<br>chr3R:27807843-27810289 // 93.25 ///<br>chr3R:27805810-27807414 // 75.02 ///<br>chr3R:27805778-27806610 // 59.79 ///<br>chrUextra:5186009-5186854 // 61.07 ///<br>chrUextra:5186109-5186897 // 56.11 ///<br>chrUextra:23175062-23175709 // 46.36

CG31012 /// DmadCG31012 /// DsubCG31012

	Ab.vs.FRT	AbSCRIB.vs.FRT	Alignments
1633711_a_at	-1	0	chr3R:26648194-26652542 // 100.0
1640850_at	1	1	chr3R:26648194-26652542 // 100.0

CG31038

	Ab.vs.FRT	AbSCRIB.vs.FRT	Alignments
1625178_at	0	-1	chr3R:25702804-25728953 // 100.0
1632360_s_at	-1	-1	chr3R:25702804-25724533 // 100.0

CG31688

	Ab.vs.FRT	AbSCRIB.vs.FRT	Alignments
1628583_at	-1	-1	chr2L:20429026-20443945 // 100.0 ///<br>chr2L:20451413-20453063 // 51.39
1635310_s_at	-1	-1	chr2L:20429026-20443945 // 100.0 ///<br>chr2L:20451413-20453063 // 51.39

CG32141

	Ab.vs.FRT	AbSCRIB.vs.FRT	Alignments
1629928_a_at	1	1	chr3L:14196188-14196842 // 100.0
1641101_at	1	1	chr3L:14196533-14197309 // 100.0

CG32626

	Ab.vs.FRT	AbSCRIB.vs.FRT	Alignments
1631487_s_at	0	0	chrX:13733563-13733803 // 100.0
1632081_s_at	-1	-1	chrX:13737986-13739508 // 100.0
1636030_s_at	0	1	chrX:13731635-13732118 // 100.0
1640932_s_at	0	1	chrX:13724781-13739863 // 100.0

CG32694

	Ab.vs.FRT	AbSCRIB.vs.FRT	Alignments
1634339_at	0	1	chrX:9883727-9896198 // 100.0

1635605_at	0	0	chrX:9883727-9895227 // 100.0
1636772_s_at	0	1	chrX:9883727-9900918 // 100.0

CG32982

	Ab.vs.FRT	AbSCRIB.vs.FRT	Alignments
1634315_a_at	0	1	chr2L:9125750-9163716 // 100.0
1634406_at	0	0	chr2L:9151200-9163727 // 100.0
1636907_at	-1	-1	chr2L:9133861-9135888 // 100.0

CG33054

	Ab.vs.FRT	AbSCRIB.vs.FRT	Alignments
1625443_a_at	1	1	chr3L:21190581-21195978 // 100.0
1628251_at	1	1	chr3L:21190581-21195978 // 100.0
1637050_at	0	0	chr3L:21190510-21191272 // 100.0

CG33057 /// mkg-p

	Ab.vs.FRT	AbSCRIB.vs.FRT	Alignments
1622892_s_at	0	1	chr3L:8395406-8398372 // 100.0
1635157_at	1	1	chr3L:8395266-8398372 // 100.0

CG33096

	Ab.vs.FRT	AbSCRIB.vs.FRT	Alignments
1639117_a_at	-1	-1	chr3R:20923363-20925087 // 100.0
1640060_at	-1	-1	chr3R:20923395-20925087 // 100.0

CG33169

	Ab.vs.FRT	AbSCRIB.vs.FRT	Alignments
1631473_at	1	1	chr3L:22856965-22858309 // 99.6
1632606_a_at	0	0	chr3L:22856963-22857596 // 100.0
1641489_at	0	1	chr3L:22856963-22857596 // 100.0

CG33174 /// DmirCG33174

	Ab.vs.FRT	AbSCRIB.vs.FRT	Alignments
1625176_at	0	-1	chrX:13677781-13705534 // 100.0
1627706_a_at	-1	-1	chrX:13677781-13699676 // 100.0
1637006_at	0	0	chrX:13677781-13699676 // 100.0
1640287_s_at	0	0	chrX:13703397-13705206 // 100.0

CG33960

	Ab.vs.FRT	AbSCRIB.vs.FRT	Alignments
1623684_at	-1	-1	chr2R:12310488-12316320 // 100.0
1635739_at	-1	-1	chr2R:12286431-12287015 // 100.0

CG33967

	Ab.vs.FRT	AbSCRIB.vs.FRT	Alignments
1624053_at	0	1	chr3R:10549028-10549978 // 100.0
1632884_at	1	1	chr3R:10524605-10529231 // 100.0

CG33993 /// DwilCG33993

	Ab.vs.FRT	AbSCRIB.vs.FRT	Alignments
1628447_at	-1	-1	chr3L:5930378-5943755 // 100.0
1639605_at	-1	-1	chr3L:5926869-5927778 // 100.0

CG34104

	Ab.vs.FRT	AbSCRIB.vs.FRT	Alignments
1635113_at	0	-1	chrX:10040218-10041405 // 100.0
1636704_at	0	0	chrX:9980565-10032744 // 99.85
1637474_at	1	1	chrX:9982221-10005780 // 99.3

CG34348

	Ab.vs.FRT	AbSCRIB.vs.FRT	Alignments
1626123_at	1	1	chrX:11234484-11234782 // 100.0
1626316_at	1	1	chrX:11237288-11238128 // 100.0

CG34360

	Ab.vs.FRT	AbSCRIB.vs.FRT	Alignments
1624009_at	-1	-1	chr3R:5701396-5702032 // 100.0
1629573_at	-1	-1	chr3R:5812358-5813521 // 100.0
1640397_at	-1	-1	chr3R:5754637-5769634 // 100.0

CG34377

	Ab.vs.FRT	AbSCRIB.vs.FRT	Alignments
1625709_at	0	0	chr3R:18144055-18144439 // 100.0
1629892_at	1	1	chr3R:18137664-18137908 // 100.0
1633871_at	1	0	chr3R:18140585-18141527 // 100.0

CG34379

	Ab.vs.FRT	AbSCRIB.vs.FRT	Alignments
1627487_at	-1	-1	chr2R:10213330-10217323 // 100.0
1629937_a_at	0	0	chr2R:10219899-10239312 // 100.0
1630913_at	1	1	chr2R:10202834-10203193 // 100.0

CG34383

	Ab.vs.FRT	AbSCRIB.vs.FRT	Alignments
1623585_at	-1	0	chr3R:9571396-9573727 // 100.0
1625511_at	0	0	chr3R:9582097-9587375 // 100.0
1627413_at	1	0	chr3R:9575117-9581360 // 100.0
1633756_at	0	0	chr3R:9560232-9561689 // 81.78

CG34398

	Ab.vs.FRT	AbSCRIB.vs.FRT	Alignments
1628538_at	-1	-1	chr2L:8915834-8916065 // 100.0
1635127_at	1	1	chr2L:8932900-8933084 // 90.2
1640585_at	0	-1	chr2L:8922471-8931938 // 100.0

CG34417

	Ab.vs.FRT	AbSCRIB.vs.FRT	Alignments
1628465_a_at	0	0	chrX:6465955-6483205 // 100.0
1636450_at	1	1	chrX:6474073-6479688 // 99.86
1638063_at	-1	-1	chrX:6434803-6456481 // 100.0

CG3655

	Ab.vs.FRT	AbSCRIB.vs.FRT	Alignments
1625537_at	1	0	chrX:967938-976472 // 99.83
1628031_at	0	0	chrX:1013723-1036400 // 100.0
1639872_at	1	0	chrX:990527-996570 // 100.0

CG3857

	Ab.vs.FRT	AbSCRIB.vs.FRT	Alignments
1623351_at	-1	-1	chrX:1832523-1834040 // 100.0
1638469_s_at	1	1	chr2RHet:2204719-2212330 // 99.77 /// chr3L:22796867-22804474 // 99.63 /// chr3L:24265247-24272857 // 99.63 /// chr3L:23365306-24265765 // 99.81 /// chr3R:13858554-13866170 // 99.83 /// chrX:3072555-3080161 // 99.87 /// chr2L:14984452-14991975 // 100.0 /// chr2R:2839725-2847248 // 100.0 /// chr2R:1267561-1275169 // 99.84 /// chr2R:9615693-9623293 // 99.72 /// chrU:2246520-2253957 // 97.41 /// chr3L:23378273-23385891 // 99.8 /// chr3R:19676834-19684444 // 99.87 /// chrX:1824624-1832138 // 99.85 /// chrX:14445731-14453333 // 99.81 /// chr2L:4661879-4669402 // 100.0 /// chr2L:19657679-19665288 // 99.88 /// chr2L:2112167-2119772 // 99.84 /// chr2L:10592896-10600501 // 99.8 /// chrU:1442881-1450490 // 99.79

CG40115

	Ab.vs.FRT	AbSCRIB.vs.FRT	Alignments
1630184_at	1	1	chrUextra:1674993-1675537 // 96.8 /// chrUextra:1337447-1337991 // 96.44 /// chrUextra:2559334-2559890 // 96.44 ///

			chrUextra:2058066-2155830 // 96.44 /// chrUextra:11472559-11473104 // 94.31 /// chrUextra:14246965-14247518 // 94.84 /// chrUextra:481866-847647 // 94.66 /// chr3RHet:572203-572767 // 96.8 /// chr3RHet:1256112-1256676 // 96.8 /// chrYHet:194512-195048 // 91.28 /// chrU:7748781-7749342 // 98.93 /// chrUextra:9061472-9062034 // 99.82 /// chr2RHet:2266658-2267220 // 99.82 /// chr2R:2185813-2186358 // 94.48 /// chrU:295072-295635 // 97.69 /// chrU:1211900-1212456 // 96.44 /// chrU:1219672-1220228 // 96.44 /// chrU:1225448-1226004 // 96.44
1641245_a_at	1	1	chr3RHet:572203-572767 // 96.8 /// chr3RHet:1256112-1256676 // 96.8 /// chrYHet:194512-195048 // 91.28 /// chrU:7748781-7749342 // 98.93 /// chrUextra:1674993-1675537 // 96.8 /// chrUextra:1337447-1337991 // 96.44 /// chrUextra:2559334-2559890 // 96.44 /// chrUextra:2058066-2155830 // 96.44 /// chrUextra:11472559-11473104 // 94.31 /// chrUextra:14246965-14247518 // 94.84 /// chrUextra:481866-847647 // 94.66 /// chr2RHet:2266658-2267220 // 99.82 /// chr2R:2185813-2186358 // 94.48 /// chrU:295072-295635 // 97.69 /// chrU:1211900-1212456 // 96.44 /// chrU:1219672-1220228 // 96.44 /// chrU:1225448-1226004 // 96.44 /// chrUextra:9061472-9062034 // 99.82

CG41284

	Ab.vs.FRT	AbSCRIB.vs.FRT	Alignments
1634671_a_at	1	1	chr3LHet:1703266-1703515 // 100.0 /// chr3LHet:1652596-1652845 // 99.6
1637236_at	1	0	chr3LHet:1702041-1703582 // 100.0 /// chr3LHet:1646351-1652912 // 99.75

CG42232

	Ab.vs.FRT	AbSCRIB.vs.FRT	Alignments
1635452_a_at	0	0	chr3R:12292740-12295350 // 100.0
1636205_at	-1	0	chr3R:12284001-12288309 // 100.0
1640737_at	1	1	chr3R:12290226-12295342 // 100.0

CG42271

	Ab.vs.FRT	AbSCRIB.vs.FRT	Alignments

1632756_at	-1	0	chrX:14122267-14128085 // 100.0
1641372_at	1	1	chrX:14126314-14128085 // 100.0

CG42327

	Ab.vs.FRT	AbSCRIB.vs.FRT	Alignments
1629723_at	-1	0	chr3R:7498039-7499184 // 100.0
1633732_at	-1	-1	chr3R:7493326-7496707 // 100.0

CG42336

	Ab.vs.FRT	AbSCRIB.vs.FRT	Alignments
1629625_at	0	1	chr2R:7182048-7184980 // 100.0
1633547_a_at	1	1	chr2R:7182048-7184980 // 100.0
1633572_at	0	0	chr2R:7182903-7183317 // 100.0
1638448_at	0	0	chr2R:7182048-7182859 // 100.0

CG42342

	Ab.vs.FRT	AbSCRIB.vs.FRT	Alignments
1631471_at	-1	-1	chr3R:12381876-12394335 // 100.0
1633321_at	-1	-1	chr3R:12339171-12339918 // 100.0
1635779_at	-1	-1	chr3R:12375758-12378280 // 100.0
1639558_at	0	0	chr3R:12394665-12396133 // 100.0

CG4655

	Ab.vs.FRT	AbSCRIB.vs.FRT	Alignments
1626639_a_at	0	0	chr3R:6663114-6667710 // 100.0
1628333_at	-1	-1	chr3R:6665789-6667768 // 99.75
1638442_at	-1	-1	chr3R:6663114-6667710 // 100.0

CG4662

	Ab.vs.FRT	AbSCRIB.vs.FRT	Alignments
1626421_at	0	1	chr3R:15673044-15678698 // 100.0
1627462_a_at	0	0	chr3R:15673044-15678698 // 100.0
1635307_at	1	1	chr3R:15673044-15680125 // 100.0

CG5091 /// CG5096

	Ab.vs.FRT	AbSCRIB.vs.FRT	Alignments
1636630_s_at	0	1	chr2L:10380117-10382865 // 100.0
1639478_at	1	1	chr2L:10382252-10384730 // 100.0

CG5174

	Ab.vs.FRT	AbSCRIB.vs.FRT	Alignments
1623053_a_at	-1	0	chr2R:14308914-14313515 // 100.0
1624146_at	0	0	chr2R:14308914-14312388 // 100.0
1634112_a_at	-1	-1	chr2R:14310438-14313433 // 100.0
1640233_at	0	0	chr2R:14310438-14313433 // 100.0

CG5325

	Ab.vs.FRT	AbSCRIB.vs.FRT	Alignments
1626588_a_at	-1	-1	chr2L:12093770-12094635 // 100.0
1639148_at	-1	-1	chr2L:12093770-12095252 // 100.0

CG5326

	Ab.vs.FRT	AbSCRIB.vs.FRT	Alignments
1623589_a_at	-1	0	chr3R:18359027-18365639 // 100.0
1629511_at	1	1	chr3R:18357893-18365639 // 100.0

CG6000

	Ab.vs.FRT	AbSCRIB.vs.FRT	Alignments
1625179_at	1	1	chr3R:19880234-19880822 // 100.0
1631899_a_at	1	0	chr3R:19880139-19880822 // 100.0

CG6340

	Ab.vs.FRT	AbSCRIB.vs.FRT	Alignments
1631096_a_at	1	1	chrX:15376399-15378755 // 100.0
1632665_a_at	-1	-1	chrX:15376409-15380642 // 100.0
1633367_at	0	0	chrX:15376399-15378755 // 100.0
1634931_at	1	1	chrX:15376409-15378413 // 100.0

CG6791

	Ab.vs.FRT	AbSCRIB.vs.FRT	Alignments
1629690_at	-1	-1	chr3R:7411419-7415864 // 100.0
1635430_a_at	-1	-1	chr3R:7411075-7415864 // 100.0
1639787_at	0	0	chr3R:7411075-7415864 // 100.0

CG6854

	Ab.vs.FRT	AbSCRIB.vs.FRT	Alignments
1633318_at	0	0	chr3L:15097848-15098997 // 100.0
1639986_at	1	0	chr3L:15098983-15103837 // 100.0
1640191_a_at	-1	-1	chr3L:15091236-15106074 // 99.79

CG6982

	Ab.vs.FRT	AbSCRIB.vs.FRT	Alignments
1625880_at	1	1	chr3R:18552702-18554721 // 98.96
1630752_at	-1	0	chr3R:18554739-18555829 // 100.0

CG7630

	Ab.vs.FRT	AbSCRIB.vs.FRT	Alignments
1627402_a_at	-1	-1	chr3L:17418587-17419387 // 100.0
1632440_at	-1	0	chr3L:17418587-17419387 // 100.0

CG7650

	Ab.vs.FRT	AbSCRIB.vs.FRT	Alignments
1627214_s_at	-1	-1	chr3L:15602435-15603463 // 100.0
1633191_at	-1	-1	chr3L:15602435-15603463 // 100.0
1633616_at	-1	-1	chr3L:15602553-15603488 // 100.0

CG7777

	Ab.vs.FRT	AbSCRIB.vs.FRT	Alignments
1625950_a_at	0	-1	chr2R:7323750-7329728 // 100.0
1630030_at	1	0	chr2R:7323750-7329728 // 100.0

CG8086

	Ab.vs.FRT	AbSCRIB.vs.FRT	Alignments
1623894_a_at	0	1	chr2L:8244544-8257502 // 100.0
1635348_at	0	1	chr2L:8245372-8250357 // 100.0

CG8765

	Ab.vs.FRT	AbSCRIB.vs.FRT	Alignments
1627570_a_at	-1	0	chr3L:19679550-19680459 // 100.0
1634934_at	-1	-1	chr3L:19677037-19680459 // 100.0

CG9027

	Ab.vs.FRT	AbSCRIB.vs.FRT	Alignments
1622906_at	0	-1	chr2R:7268044-7268717 // 100.0
1636961_a_at	-1	-1	chr2R:7268821-7271613 // 100.0

CG9062

	Ab.vs.FRT	AbSCRIB.vs.FRT	Alignments
1624010_a_at	0	-1	chr2R:7168484-7171461 // 100.0
1634001_at	-1	-1	chr2R:7168484-7171461 // 100.0

CG9449

	Ab.vs.FRT	AbSCRIB.vs.FRT	Alignments
1628150_a_at	1	1	chr3L:19486535-19489801 // 100.0
1633200_at	1	1	chr3L:19485515-19489791 // 100.0

CG9932

	Ab.vs.FRT	AbSCRIB.vs.FRT	Alignments
1638653_a_at	1	1	chr2L:13022670-13031333 // 100.0
1640518_at	0	1	chr2L:13035355-13065175 // 100.0

chinmo

	Ab.vs.FRT	AbSCRIB.vs.FRT	Alignments
1628005_at	1	1	chr2L:1667846-1698617 // 99.76
1629484_s_at	1	1	chr2L:1653260-1697851 // 99.89
1636985_s_at	1	1	chr2L:1667846-1678921 // 100.0

cpo

	Ab.vs.FRT	AbSCRIB.vs.FRT	Alignments
1624608_s_at	0	-1	chr3R:13838879-13839188 // 100.0
1632644_s_at	-1	-1	chr3R:13757594-13841501 // 99.76
1639802_at	0	0	chr3R:13762983-13764042 // 100.0

dlg1

	Ab.vs.FRT	AbSCRIB.vs.FRT	Alignments
1624021_a_at	1	1	chrX:11283726-11301780 // 100.0
1627882_at	0	0	chrX:11263669-11283324 // 100.0
1635382_at	0	0	chrX:11269795-11296894 // 99.3
1640119_a_at	1	0	chrX:11269795-11296894 // 99.3

Dll

	Ab.vs.FRT	AbSCRIB.vs.FRT	Alignments
1625771_at	0	-1	chr2R:20702352-20722125 // 100.0
1630237_a_at	1	0	chr2R:20702352-20722125 // 100.0
1636088_at	1	1	chr2R:20702352-20722686 // 100.0

DnaJ-60

	Ab.vs.FRT	AbSCRIB.vs.FRT	Alignments
1623119_at	0	0	chr2R:20045188-20046819 // 100.0
1633399_at	1	1	chr2R:20045837-20046819 // 100.0
1636379_a_at	1	1	chr2R:20045837-20046819 // 100.0

dom

	Ab.vs.FRT	AbSCRIB.vs.FRT	Alignments
1628160_a_at	-1	-1	chr2R:17210948-17229346 // 100.0
1633331_at	0	-1	chr2R:17210948-17224453 // 100.0
1636034_at	-1	-1	chr2R:17210948-17229346 // 100.0

dsx

	Ab.vs.FRT	AbSCRIB.vs.FRT	Alignments
1623776_s_at	0	-1	chr3R:3760203-3793130 // 99.89
1640799_at	1	1	chr3R:3750044-3793130 // 100.0

ec

	Ab.vs.FRT	AbSCRIB.vs.FRT	Alignments
1626919_at	0	0	chrX:3713180-3715204 // 96.22
1629348_at	1	0	chrX:3730696-3735750 // 100.0

1631485_a_at	-1	-1	chrX:3744315-3750618 // 100.0

Ect4

	Ab.vs.FRT	AbSCRIB.vs.FRT	Alignments
1623244_at	0	1	chr3L:8094137-8101606 // 100.0
1624876_at	0	0	chr3L:8086158-8086587 // 100.0
1629571_at	0	0	chr3L:8067574-8068340 // 100.0
1631613_at	0	0	chr3L:8089646-8092739 // 100.0
1632067_at	1	1	chr3L:8094137-8101938 // 100.0
1635126_a_at	0	1	chr3L:8094137-8101938 // 100.0

egh

	Ab.vs.FRT	AbSCRIB.vs.FRT	Alignments
1631621_s_at	1	1	chrX:2482596-2490218 // 99.72
1634558_s_at	0	1	chrX:2482533-2492638 // 100.0

egr

	Ab.vs.FRT	AbSCRIB.vs.FRT	Alignments
1629442_at	-1	-1	chr2R:5966516-5969137 // 100.0
1639671_at	-1	-1	chr2R:5970119-5971666 // 100.0

eIF-4B

	Ab.vs.FRT	AbSCRIB.vs.FRT	Alignments
1629261_at	1	1	chr2RHet:2901815-2902500 // 100.0
1636358_a_at	-1	-1	chr2RHet:2901815-2902500 // 100.0
1640194_a_at	1	1	chr2RHet:2901751-2917318 // 100.0
1640653_at	1	0	chr2RHet:2901751-2917318 // 100.0

Eip75B

	Ab.vs.FRT	AbSCRIB.vs.FRT	Alignments
1623164_a_at	-1	-1	chr3L:17946457-18052698 // 100.0
1635393_s_at	-1	-1	chr3L:17946097-17946705 // 100.0
1640781_a_at	-1	-1	chr3L:17945062-17992921 // 100.0

elB

	Ab.vs.FRT	AbSCRIB.vs.FRT	Alignments
1623877_a_at	-1	-1	chr2L:14391177-14407758 // 100.0
1631207_at	-1	-1	chr2L:14391177-14407758 // 100.0
1640000_at	0	0	chr2L:14387121-14407758 // 100.0

Fancd2

	Ab.vs.FRT	AbSCRIB.vs.FRT	Alignments
1629311_at	-1	0	chr3R:16640912-16641815 // 100.0
1640096_at	0	1	chr3R:16636525-16640799 // 100.0

Fas3

	Ab.vs.FRT	AbSCRIB.vs.FRT	Alignments
1628543_a_at	0	-1	chr2L:18320100-18392575 // 99.29
1641473_at	1	0	chr2L:18320100-18392575 // 99.4

fau

	Ab.vs.FRT	AbSCRIB.vs.FRT	Alignments
1628880_at	0	0	chr3R:6598399-6603083 // 100.0
1630161_at	0	0	chr3R:6600265-6603083 // 100.0
1633459_a_at	0	-1	chr3R:6599253-6603083 // 100.0
1638789_at	0	-1	chr3R:6599253-6603083 // 100.0
1640350_at	0	0	chr3R:6593938-6603083 // 100.0

Fili

	Ab.vs.FRT	AbSCRIB.vs.FRT	Alignments
1626772_at	-1	-1	chr2R:17817024-17818166 // 100.0
1628113_at	-1	-1	chr2R:17830450-17831981 // 100.0
1631503_at	0	0	chr2R:17771005-17813856 // 100.0

Fmr1

	Ab.vs.FRT	AbSCRIB.vs.FRT	Alignments
1632612_s_at	-1	-1	chr3R:5929821-5935844 // 100.0
1634028_s_at	1	1	chr3R:5927135-5935843 // 100.0

fru

	Ab.vs.FRT	AbSCRIB.vs.FRT	Alignments
1624575_a_at	0	0	chr3R:14248359-14371244 // 100.0
1629904_at	0	1	chr3R:14256948-14261513 // 100.0
1631498_a_at	1	1	chr3R:14251664-14371244 // 99.86
1632859_a_at	-1	-1	chr3R:14242314-14371244 // 100.0
1634379_a_at	-1	-1	chr3R:14256429-14261513 // 100.0
1638111_at	0	0	chr3R:14371199-14371631 // 100.0
1641338_at	0	1	chr3R:14256429-14261513 // 100.0

Galpha49B

	Ab.vs.FRT	AbSCRIB.vs.FRT	Alignments
1624798_s_at	1	0	chr2R:8500245-8507806 // 100.0
1639773_s_at	1	1	chr2R:8503292-8506485 // 100.0

Gapdh2

	Ab.vs.FRT	AbSCRIB.vs.FRT	Alignments
1627915_a_at	0	-1	chrX:15762222-15763695 // 100.0
1632667_s_at	-1	-1	chrX:15762335-15763662 // 100.0

garz

	Ab.vs.FRT	AbSCRIB.vs.FRT	Alignments
1626339_at	1	1	chr2R:8214178-8221327 // 100.0
1636686_at	0	0	chr2R:8214178-8219975 // 100.0
1639361_a_at	0	-1	chr2R:8214178-8219975 // 100.0

Gfat1

	Ab.vs.FRT	AbSCRIB.vs.FRT	Alignments
1634707_s_at	-1	-1	chr3RHet:2474288-2481648 // 100.0
1635696_s_at	1	1	chr2LHet:330880-337332 // 99.4 /// chr3RHet:2458022-2464475 // 99.63 /// chr3RHet:2463977-2470430 // 99.63 /// chr3L:21365875-21372325 // 100.0 /// chr4:34274-40712 // 99.32 /// chr3LHet:336972-343625 // 95.57

Glut1

	Ab.vs.FRT	AbSCRIB.vs.FRT	Alignments
1628757_at	0	-1	chr3L:914073-982582 // 99.41
1634033_s_at	0	-1	chr3L:959661-984440 // 100.0

Gpdh

	Ab.vs.FRT	AbSCRIB.vs.FRT	Alignments
1616608_a_at	-1	-1	chr2L:5943681-5948313 // 100.0
1625949_at	0	0	chr2L:5943681-5948313 // 100.0
1634893_at	0	0	chr2L:5943681-5949092 // 100.0
1636311_at	-1	-1	chr2L:5943681-5947625 // 100.0

grp

	Ab.vs.FRT	AbSCRIB.vs.FRT	Alignments
1625416_x_at	1	1	chr2L:16685836-16687059 // 100.0
1629880_at	1	1	chr2L:16685836-16687059 // 100.0
1634230_s_at	0	0	chr2L:16680087-16699926 // 100.0

GV1

	Ab.vs.FRT	AbSCRIB.vs.FRT	Alignments
1626287_at	1	1	chr3L:1810588-1822903 // 100.0
1631252_a_at	0	0	chr3L:1810588-1822220 // 100.0
1635266_at	1	1	chr3L:1810588-1822220 // 100.0

His1:CG31617 /// His1:CG33801 /// His1:CG33804 /// His1:CG33807 /// His1:CG33810 /// His1:CG33813 /// His1:CG33816 /// His1:CG33819 /// His1:CG33822 /// His1:CG33825 /// His1:CG33828 /// His1:CG33831 /// His1:CG33834 /// His1:CG33837 /// His1:CG33840 /// His1:CG33843 /// His1:CG33846 /// His1:CG33849 /// His1:CG33852 /// His1:CG33855 /// His1:CG33858 /// His1:CG33861 /// His1:CG33864

	Ab.vs.FRT	AbSCRIB.vs.FRT	Alignments

1629740_at	1	1	chrUextra:10958515-11389945 // 100.0 /// chrUextra:17519559-17974119 // 99.89 /// chrUextra:10798897-11720181 // 99.89 /// chr2L:21456232-21457110 // 100.0 /// chr2L:21421974-21422852 // 99.89 /// chr2L:21437130-21438008 // 99.89 /// chr2L:21451171-21452049 // 99.89 /// chr2L:21461292-21462170 // 99.89 /// chr2L:21466335-21467213 // 99.89 /// chr2L:21471378-21472256 // 99.89 /// chr2L:21476421-21477299 // 99.89 /// chr2L:21481464-21482342 // 99.89 /// chr2L:21491660-21492538 // 99.89 /// chr2L:21496705-21497583 // 99.89 /// chr2L:21501752-21502630 // 99.89 /// chr2L:21506803-21507681 // 99.89 /// chr2L:21511847-21512725 // 99.89 /// chr2L:21536465-21537343 // 99.89 /// chrUextra:16984316-17104264 // 100.0 /// chrUextra:10801500-10986150 // 99.77
1631321_s_at	1	1	chrUextra:10958515-11029897 // 97.1 /// chrUextra:18013913-18251413 // 96.96 /// chrUextra:17519559-17973946 // 96.96 /// chrUextra:10798897-11389772 // 97.1 /// chr2L:21542983-21543706 // 100.0 /// chr2L:21437130-21437835 // 97.1 /// chr2L:21456232-21456937 // 97.1 /// chr2L:21461292-21461997 // 97.1 /// chr2L:21466335-21467040 // 97.1 /// chr2L:21471378-21472083 // 97.1 /// chr2L:21476421-21477126 // 97.1 /// chr2L:21481464-21482169 // 97.1 /// chr2L:21521735-21522440 // 97.1 /// chr2L:21526578-21527283 // 97.1 /// chr2L:21531421-21532126 // 97.1 /// chr2L:21421974-21422679 // 96.96 /// chr2L:21427035-21427740 // 96.96 /// chr2L:21451171-21451876 // 96.96 /// chrUextra:12271498-12279438 // 97.1 /// chrUextra:16984489-17104264 // 97.1

His3:CG31613 /// His3:CG33803 /// His3:CG33806 /// His3:CG33809 /// His3:CG33812 /// His3:CG33815 /// His3:CG33818 /// His3:CG33821 /// His3:CG33824 /// His3:CG33827 /// His3:CG33830 /// His3:CG33833 /// His3:CG33836 /// His3:CG33839 /// His3:CG33842 /// His3:CG33845 /// His3:CG33848 /// His3:CG33851 /// His3:CG33854 /// His3:CG33857 /// His3:CG33860 /// His3:CG33863 /// His3:CG33866

	Ab.vs.FRT	AbSCRIB.vs.FRT	Alignments
1626867_at	-1	0	chr2L:21420185-21420596 // 100.0 /// chr2L:21425249-21425660 // 100.0 /// chr2L:21430311-21430722 // 100.0 /// chr2L:21435355-21435766 // 100.0 /// chr2L:21440243-21440654 // 100.0 ///

			chr2L:21454444-21454855 // 100.0 /// chr2L:21484737-21485148 // 100.0 /// chr2L:21489886-21490297 // 100.0 /// chr2L:21494931-21495342 // 100.0 /// chr2L:21499976-21500387 // 100.0 /// chr2L:21505029-21505440 // 100.0 /// chr2L:21510074-21510485 // 100.0 /// chr2L:21515118-21515529 // 100.0 /// chr2L:21520165-21520576 // 100.0 /// chr2L:21525008-21525419 // 100.0 /// chr2L:21529851-21530262 // 100.0 /// chrUextra:1208196-1208607 // 100.0 /// chrUextra:7733671-7734082 // 100.0 /// chrUextra:7958547-7958958 // 100.0 /// chrUextra:9788102-9788513 // 100.0
1638308_s_at	-1	0	chrUextra:1208196-1208607 // 100.0 /// chrUextra:7733671-7734082 // 100.0 /// chrUextra:7958547-7958958 // 100.0 /// chrUextra:9788102-9788513 // 100.0 /// chrUextra:10534527-10534938 // 100.0 /// chrUextra:12095133-12095544 // 100.0 /// chrUextra:12188206-12188617 // 100.0 /// chrUextra:13391895-13392306 // 100.0 /// chrUextra:17944964-17945375 // 100.0 /// chrUextra:19464124-19464535 // 100.0 /// chrUextra:9266712-9403271 // 100.0 /// chr2L:21420185-21420596 // 100.0 /// chr2L:21425249-21425660 // 100.0 /// chr2L:21430311-21430722 // 100.0 /// chr2L:21435355-21435766 // 100.0 /// chr2L:21440243-21440654 // 100.0 /// chr2L:21454444-21454855 // 100.0 /// chrUextra:8670477-8670888 // 100.0 /// chrUextra:10813270-10813681 // 100.0 /// chrUextra:10937176-10937587 // 100.0

Hrb98DE

	Ab.vs.FRT	AbSCRIB.vs.FRT	Alignments
1638912_s_at	1	1	chr3R:24426108-24431499 // 100.0
1640169_s_at	0	-1	chr3R:24425528-24430817 // 100.0

Hsp67Bb

	Ab.vs.FRT	AbSCRIB.vs.FRT	Alignments
1629061_s_at	-1	-1	chr3L:9366030-9368070 // 100.0
1630487_s_at	0	-1	chr3L:9366030-9366749 // 100.0

hts

	Ab.vs.FRT	AbSCRIB.vs.FRT	Alignments
1632191_s_at	-1	-1	chr2R:15294404-15312454 // 100.0
1633226_at	0	0	chr2R:15296359-15312454 // 100.0

1634736_at	0	0	chr2R:15287021-15312454 // 100.0
1634962_s_at	0	0	chr2R:15284839-15312454 // 100.0
1641531_at	1	1	chr2R:15294404-15312454 // 100.0

Idgf3

	Ab.vs.FRT	AbSCRIB.vs.FRT	Alignments
1623643_s_at	1	1	chr2L:16451070-16453176 // 100.0
1626301_at	1	1	chr2L:16451070-16453176 // 100.0

ImpE1

	Ab.vs.FRT	AbSCRIB.vs.FRT	Alignments
1625625_at	0	-1	chr3L:8365508-8384271 // 99.83
1631375_a_at	0	-1	chr3L:8365508-8373250 // 100.0
1634617_at	0	0	chr3L:8363024-8365524 // 100.0
1634661_at	0	-1	chr3L:8365508-8373250 // 100.0

jing

	Ab.vs.FRT	AbSCRIB.vs.FRT	Alignments
1628528_at	1	1	chr2R:2390272-2391223 // 100.0
1636066_s_at	-1	-1	chr2R:2501283-2506901 // 100.0

l(1)G0196

	Ab.vs.FRT	AbSCRIB.vs.FRT	Alignments
1624265_at	0	0	chrX:21889018-21909287 // 100.0
1625785_at	0	0	chrX:21889018-21905765 // 100.0
1632658_a_at	0	1	chrX:21889018-21904734 // 100.0
1633081_at	0	0	chrX:21889018-21906262 // 100.0
1641542_at	0	1	chrX:21889018-21904734 // 100.0

l(2)efl

	Ab.vs.FRT	AbSCRIB.vs.FRT	Alignments
1626416_a_at	0	-1	chr2R:19572320-19573044 // 100.0
1627242_at	0	-1	chr2R:19572173-19573063 // 100.0
1635952_at	0	0	chr2R:19572320-19573044 // 100.0

lilli

	Ab.vs.FRT	AbSCRIB.vs.FRT	Alignments
1623171_s_at	1	1	chr2L:2885951-2945194 // 99.8
1635264_s_at	1	0	chr2L:2885951-2952597 // 100.0

Lim1

	Ab.vs.FRT	AbSCRIB.vs.FRT	Alignments
1629733_at	1	0	chrX:8651267-8699808 // 100.0
1633262_s_at	1	1	chrX:8699236-8699979 // 100.0

lola

	Ab.vs.FRT	AbSCRIB.vs.FRT	Alignments
1623411_at	0	0	chr2R:6375366-6422583 // 100.0
1624729_at	0	0	chr2R:6412903-6422583 // 100.0
1625768_s_at	0	0	chr2R:6407124-6430787 // 100.0
1627324_at	0	0	chr2R:6389585-6422583 // 100.0
1628421_at	1	1	chr2R:6383760-6384236 // 96.75
1628946_at	1	1	chr2R:6403044-6422583 // 100.0
1629523_at	0	0	chr2R:6409839-6422583 // 100.0
1630936_at	0	0	chr2R:6414184-6422583 // 100.0
1633089_a_at	-1	-1	chr2R:6379446-6422582 // 100.0
1633422_a_at	-1	-1	chr2R:6395851-6430794 // 100.0
1634495_s_at	0	0	chr2R:6419508-6421862 // 100.0
1635096_at	1	1	chr2R:6405425-6422583 // 100.0
1637581_at	-1	-1	chr2R:6370444-6422583 // 100.0
1640280_at	0	0	chr2R:6392363-6422583 // 100.0
1640945_at	0	-1	chr2R:6388140-6422583 // 100.0
1641609_at	0	0	chr2R:6411001-6429162 // 100.0

mbl

	Ab.vs.FRT	AbSCRIB.vs.FRT	Alignments
1631120_at	0	0	chr2R:13153057-13248991 // 100.0
1636099_s_at	0	0	chr2R:13238598-13263374 // 100.0
1639930_at	1	1	chr2R:13153057-13186059 // 99.85
1640686_s_at	-1	-1	chr2R:13261444-13262572 // 50.0
1641114_at	1	1	chr2R:13153057-13241068 // 100.0

MED9

	Ab.vs.FRT	AbSCRIB.vs.FRT	Alignments
1627646_at	1	1	chr2R:14054781-14055885 // 100.0
1634176_a_at	1	1	chr2R:14054781-14055885 // 100.0

Mical

	Ab.vs.FRT	AbSCRIB.vs.FRT	Alignments
1623984_s_at	0	1	chr3R:5828032-5867720 // 99.68
1636412_at	1	0	chr3R:5843964-5867720 // 98.93

mirr

	Ab.vs.FRT	AbSCRIB.vs.FRT	Alignments
1635669_a_at	0	-1	chr3L:12686847-12702491 // 100.0
1639798_at	1	0	chr3L:12686847-12703243 // 100.0

Mmp1

	Ab.vs.FRT	AbSCRIB.vs.FRT	Alignments
1623160_at	0	1	chr2R:20558816-20574897 // 100.0
1625761_a_at	0	1	chr2R:20562188-20574897 // 100.0

1632204_at	1	1	chr2R:20562188-20574897 // 100.0

mod(mdg4)

	Ab.vs.FRT	AbSCRIB.vs.FRT	Alignments
1622976_at	0	0	chr3R:17180941-17203121 // 100.0
1624020_at	0	0	chr3R:17197044-17203121 // 100.0
1625283_at	0	0	chr3R:17187909-17203121 // 100.0
1626650_at	1	1	chr3R:17184011-17203121 // 100.0
1627483_at	0	0	chr3R:17176748-17177077 // 100.0
1627603_at	0	0	chr3R:17200781-17203121 // 82.21
1627953_at	1	1	chr3R:17182071-17203121 // 100.0
1628562_s_at	0	0	chr3R:17186109-17187009 // 37.8 /// chr3R:17200781-17203121 // 62.29
1628921_s_at	0	0	chr3R:17185909-17187851 // 100.0
1628932_at	0	-1	chr3R:17200781-17203121 // 66.62
1630536_at	0	0	chr3R:17188688-17203121 // 100.0
1631500_at	0	0	chr3R:17177330-17203121 // 100.0
1631742_s_at	0	0	chr3R:17180159-17203121 // 100.0
1631833_at	0	0	chr3R:17190173-17203121 // 100.0
1632973_at	1	1	chr3R:17183242-17203121 // 100.0
1633152_at	0	0	chr3R:17177760-17203121 // 100.0
1634052_s_at	0	0	chr3R:17177760-17203121 // 100.0
1634258_at	0	0	chr3R:17197884-17203121 // 100.0
1636471_at	0	0	chr3R:17200781-17203121 // 80.27
1636765_at	0	0	chr3R:17182776-17203121 // 100.0
1637723_at	0	0	chr3R:17200780-17203121 // 80.61
1638041_at	0	-1	chr3R:17180159-17203121 // 100.0
1638829_s_at	1	1	chr3R:17184021-17203121 // 100.0
1639308_at	1	0	chr3R:17178092-17203121 // 100.0
1640268_at	1	1	chr3R:17195821-17203121 // 100.0
1640554_at	0	0	chr3R:17179070-17203121 // 100.0

mRpS11

	Ab.vs.FRT	AbSCRIB.vs.FRT	Alignments
1625994_a_at	1	1	chr3R:12898452-12899531 // 100.0
1628902_at	-1	-1	chr3R:12898452-12899531 // 100.0

msl-3

	Ab.vs.FRT	AbSCRIB.vs.FRT	Alignments
1627738_a_at	-1	-1	chr3L:7116570-7118580 // 100.0
1630477_at	0	1	chr3L:7116309-7118623 // 100.0

Msp-300

	Ab.vs.FRT	AbSCRIB.vs.FRT	Alignments
1625210_a_at	0	1	chr2L:5121290-5158264 // 100.0
1627250_at	0	1	chr2L:5152119-5152283 // 96.47
1632945_at	-1	-1	chr2L:5187675-5205389 // 100.0
1641672_at	0	0	chr2L:5158457-5186211 // 99.98

1641717_s_at	-1	0	chr2L:5109145-5111039 // 100.0

mthl4

	Ab.vs.FRT	AbSCRIB.vs.FRT	Alignments
1627967_a_at	1	1	chrUextra:13596162-13596889 // 42.13 /// chrUextra:13016046-13016713 // 38.25 /// chr2R:13332990-13334733 // 100.0
1641282_at	1	1	chrUextra:13596162-13596889 // 39.12 /// chrUextra:13016046-13016713 // 35.52 /// chr2R:13332810-13334733 // 100.0

mthl8

	Ab.vs.FRT	AbSCRIB.vs.FRT	Alignments
1629362_at	1	1	chr3L:22946-26316 // 100.0
1633386_s_at	1	1	chr3L:22946-23592 // 100.0

mud

	Ab.vs.FRT	AbSCRIB.vs.FRT	Alignments
1624624_at	0	1	chrX:14141875-14149287 // 99.97
1626073_a_at	0	-1	chrX:14141875-14149287 // 99.97
1628717_a_at	0	0	chrX:14141875-14152263 // 99.97

Neu3

	Ab.vs.FRT	AbSCRIB.vs.FRT	Alignments
1627649_at	1	1	chr3R:10493568-10498427 // 100.0
1635984_at	-1	-1	chr3R:10491558-10493505 // 100.0

Nmnat

	Ab.vs.FRT	AbSCRIB.vs.FRT	Alignments
1625565_at	1	1	chr3R:20770958-20772555 // 100.0
1627165_a_at	-1	0	chr3R:20770958-20772555 // 100.0
1635881_at	1	1	chr3R:20770958-20773122 // 100.0

nod

	Ab.vs.FRT	AbSCRIB.vs.FRT	Alignments
1626438_at	1	1	chrX:11480262-11482941 // 100.0 /// chrX:11484448-11487127 // 100.0 /// chrX:11486541-11489220 // 100.0 /// chrX:11488634-11493406 // 100.0 /// chrX:11478884-11480848 // 96.44
1639689_at	0	-1	chrX:11474850-11479787 // 100.0

norpA

	Ab.vs.FRT	AbSCRIB.vs.FRT	Alignments
1630119_s_at	1	1	chrX:4257042-4258910 // 100.0

1636576_s_at	1	1	chrX:4216658-4256487 // 100.0

oaf

	Ab.vs.FRT	AbSCRIB.vs.FRT	Alignments
1624913_s_at	-1	-1	chr2L:2492954-2497357 // 100.0
1626218_s_at	-1	-1	chr2L:2492954-2498234 // 100.0
1627494_s_at	0	0	chr2L:2492954-2498846 // 100.0

obst-E

	Ab.vs.FRT	AbSCRIB.vs.FRT	Alignments
1628498_at	0	1	chr2L:5762673-5766141 // 100.0
1638612_at	0	1	chr2L:5763459-5766141 // 100.0

olf186-F

	Ab.vs.FRT	AbSCRIB.vs.FRT	Alignments
1625514_s_at	0	1	chr2R:13730949-13748075 // 100.0
1637534_at	1	1	chr2R:13738383-13743293 // 100.0

Pabp2

	Ab.vs.FRT	AbSCRIB.vs.FRT	Alignments
1629694_at	1	1	chr2R:4018917-4020985 // 100.0
1631244_a_at	0	-1	chr2R:4018917-4020985 // 100.0
1639719_at	1	0	chr2R:4018917-4021879 // 100.0

par-1

	Ab.vs.FRT	AbSCRIB.vs.FRT	Alignments
1625544_s_at	0	0	chr2R:15371296-15371608 // 100.0
1625561_s_at	0	0	chr2R:15350488-15370547 // 100.0
1627121_s_at	0	1	chr2R:15349952-15353274 // 100.0
1628849_at	1	1	chr2R:15347437-15350136 // 100.0

PhKgamma

	Ab.vs.FRT	AbSCRIB.vs.FRT	Alignments
1623842_a_at	-1	-1	chrX:11589185-11597790 // 99.84
1624139_at	0	0	chrX:11589166-11598444 // 100.0
1632955_at	1	0	chrX:11589163-11597778 // 100.0

Pis

	Ab.vs.FRT	AbSCRIB.vs.FRT	Alignments
1625595_a_at	-1	-1	chrX:15596545-15597798 // 100.0
1627102_at	0	1	chrX:15595477-15597798 // 100.0
1636027_at	0	0	chrX:15596545-15597798 // 100.0

PNUTS

	Ab.vs.FRT	AbSCRIB.vs.FRT	Alignments
1623583_at	-1	0	chr2L:875037-877041 // 98.67
1633750_s_at	1	1	chr2L:870463-874149 // 99.85

ps

	Ab.vs.FRT	AbSCRIB.vs.FRT	Alignments
1625967_s_at	1	1	chr3R:5243663-5258261 // 100.0
1633612_at	1	1	chr3R:5259987-5272628 // 100.0
1635728_a_at	1	1	chr3R:5258323-5273597 // 100.0

Ptp4E

	Ab.vs.FRT	AbSCRIB.vs.FRT	Alignments
1627599_at	1	1	chrX:4833164-4848123 // 99.74
1631741_a_at	0	1	chrX:4833164-4848123 // 99.74
1637627_at	1	1	chrX:4833164-4849458 // 100.0

pyd

	Ab.vs.FRT	AbSCRIB.vs.FRT	Alignments
1627360_at	0	0	chr3R:4658908-4672009 // 100.0
1630791_a_at	-1	-1	chr3R:4658908-4672009 // 100.0
1631905_a_at	-1	-1	chr3R:4661310-4756978 // 99.74
1637322_at	0	0	chr3R:4661310-4756978 // 99.74
1637428_a_at	0	0	chr3R:4653199-4672010 // 100.0

RFeSP

	Ab.vs.FRT	AbSCRIB.vs.FRT	Alignments
1631856_a_at	-1	-1	chr2L:1612685-1614182 // 100.0
1641174_at	0	1	chr2L:1612685-1614182 // 100.0

rho-4

	Ab.vs.FRT	AbSCRIB.vs.FRT	Alignments
1623158_s_at	1	1	chr3RHet:378289-378693 // 94.39 /// chrU:518982-622483 // 96.1 /// chrX:11499722-11500110 // 90.24 /// chr3RHet:818221-819725 // 91.95 /// chr3RHet:212884-576594 // 92.44 /// chrU:3409251-3409650 // 92.2 /// chrU:1497764-1918534 // 90.98
1623692_s_at	0	0	chrX:11493873-11500034 // 100.0
1632924_at	1	1	chr3RHet:378289-378693 // 94.39 /// chrX:11499722-11500110 // 90.24 /// chrU:518982-622483 // 96.1 /// chr3RHet:818221-819725 // 91.95 /// chr3RHet:212884-576594 // 92.44 /// chrU:3409251-3409650 // 92.2 /// chrU:1497764-1918534 // 90.98

1623158_s_at
1623692_s_at
1632924_at

RhoGAP71E

	Ab.vs.FRT	AbSCRIB.vs.FRT	Alignments
1637051_at	-1	-1	chr3L:15581979-15596237 // 100.0
1640295_a_at	1	1	chr3L:15581979-15596172 // 100.0

RpL3

	Ab.vs.FRT	AbSCRIB.vs.FRT	Alignments
1625889_at	-1	-1	chr3R:7047890-7049171 // 100.0
1630270_s_at	0	0	chr3R:7047617-7050897 // 100.0
1634685_at	-1	-1	chr3R:7047617-7048738 // 100.0

RpL35

	Ab.vs.FRT	AbSCRIB.vs.FRT	Alignments
1626209_a_at	0	0	chrX:5566474-5567589 // 100.0
1627206_at	0	1	chrX:5566690-5567589 // 100.0
1635917_at	1	1	chrX:5566474-5567589 // 100.0

sbb

	Ab.vs.FRT	AbSCRIB.vs.FRT	Alignments
1624001_at	1	0	chr2R:14177077-14177347 // 100.0
1635211_at	-1	-1	chr2R:14166484-14176255 // 100.0

Sh

	Ab.vs.FRT	AbSCRIB.vs.FRT	Alignments
1623917_a_at	1	0	chrX:17833763-17933532 // 100.0
1623933_at	1	0	chrX:17844011-17909268 // 100.0
1626802_a_at	1	0	chrX:17844011-17909268 // 100.0
1641501_a_at	1	0	chrX:17823291-17909268 // 100.0

SK

	Ab.vs.FRT	AbSCRIB.vs.FRT	Alignments
1625065_s_at	1	1	chrX:5234270-5295175 // 100.0
1627752_s_at	1	1	chrX:5234270-5291171 // 100.0
1635657_s_at	0	0	chrX:5235489-5247049 // 100.0

sno

	Ab.vs.FRT	AbSCRIB.vs.FRT	Alignments
1625819_at	1	1	chrX:13089528-13105454 // 100.0
1635992_at	-1	-1	chrX:13097202-13105699 // 100.0

SP1070

	Ab.vs.FRT	AbSCRIB.vs.FRT	Alignments
1623259_at	1	1	chr2L:6984735-7004375 // 96.77
1638227_at	-1	-1	chr2L:6973529-6985013 // 100.0

spas

	Ab.vs.FRT	AbSCRIB.vs.FRT	Alignments
1628019_a_at	-1	-1	chr3R:19863939-19867573 // 100.0
1629123_at	0	1	chr3R:19863939-19868376 // 100.0

spir

	Ab.vs.FRT	AbSCRIB.vs.FRT	Alignments
1631571_a_at	0	0	chr2L:20311236-20344970 // 100.0
1635630_a_at	-1	0	chr2L:20311218-20348395 // 100.0
1637962_at	1	1	chr2L:20311236-20344970 // 100.0

Spn

	Ab.vs.FRT	AbSCRIB.vs.FRT	Alignments
1630417_at	0	-1	chr3L:2508119-2554277 // 100.0
1633021_s_at	1	0	chr3L:2541144-2554277 // 100.0

spri

	Ab.vs.FRT	AbSCRIB.vs.FRT	Alignments
1626771_at	0	0	chrX:10386494-10387770 // 100.0
1627037_s_at	-1	-1	chrX:10390753-10404675 // 100.0
1629648_s_at	-1	-1	chrX:10384499-10440769 // 100.0
1631646_at	0	0	chrX:10469418-10470210 // 100.0

ssp2

	Ab.vs.FRT	AbSCRIB.vs.FRT	Alignments
1632670_at	-1	0	chr3L:13958328-13965120 // 100.0
1634047_a_at	-1	-1	chr3L:13958304-13959026 // 100.0

Sucb

	Ab.vs.FRT	AbSCRIB.vs.FRT	Alignments
1626745_at	0	-1	chr3L:5554788-5556784 // 100.0
1635682_at	0	0	chr3L:5556037-5556784 // 100.0
1637251_a_at	-1	-1	chr3L:5556037-5556784 // 100.0

svp

	Ab.vs.FRT	AbSCRIB.vs.FRT	Alignments
1626594_s_at	1	1	chr3R:8126904-8127572 // 100.0
1628779_a_at	0	-1	chr3R:8084470-8128509 // 100.0
1635192_at	0	0	chr3R:8106831-8121860 // 100.0

Timp

	Ab.vs.FRT	AbSCRIB.vs.FRT	Alignments
1632533_at	1	1	chr3R:6029589-6038710 // 100.0
1641476_a_at	1	1	chr3R:6029615-6038710 // 100.0

tinc

	Ab.vs.FRT	AbSCRIB.vs.FRT	Alignments
1632529_at	1	1	chr3R:13678095-13693223 // 99.65
1636047_s_at	1	0	chr3R:13678095-13693223 // 99.65
1637201_s_at	-1	-1	chr3R:13678095-13696493 // 100.0
1641243_at	1	1	chr3R:13673047-13677925 // 100.0

Tl

	Ab.vs.FRT	AbSCRIB.vs.FRT	Alignments
1624769_s_at	0	1	chr3R:22624764-22653391 // 100.0
1639321_s_at	0	-1	chr3R:22624764-22668125 // 100.0

tmod

	Ab.vs.FRT	AbSCRIB.vs.FRT	Alignments
1626016_s_at	-1	-1	chr3R:26358335-26404627 // 100.0
1639114_at	-1	-1	chr3R:26400018-26400567 // 100.0

toy

	Ab.vs.FRT	AbSCRIB.vs.FRT	Alignments
1623314_at	0	0	chr4:1010353-1019500 // 100.0
1633094_a_at	0	-1	chr4:1010353-1019500 // 100.0
1633512_at	0	-1	chr4:1010353-1027011 // 100.0

Trc8

	Ab.vs.FRT	AbSCRIB.vs.FRT	Alignments
1632303_a_at	-1	-1	chr3R:25324887-25329519 // 100.0
1639025_at	-1	-1	chr3R:25324565-25329519 // 99.9

Trl

	Ab.vs.FRT	AbSCRIB.vs.FRT	Alignments
1628275_at	0	-1	chr3L:14741028-14750430 // 100.0
1629754_s_at	0	0	chr3L:14745933-14751041 // 99.54
1635305_s_at	-1	-1	chr3L:14743341-14750982 // 100.0

trol

	Ab.vs.FRT	AbSCRIB.vs.FRT	Alignments
1625116_at	0	0	chrX:2374590-2399583 // 100.0
1640189_at	0	0	chrX:2415552-2418402 // 100.0
1640223_a_at	-1	-1	chrX:2374590-2399583 // 100.0
1641442_a_at	0	1	chrX:2365015-2399583 // 100.0

Tsp

	Ab.vs.FRT	AbSCRIB.vs.FRT	Alignments
1636251_at	1	0	chr2L:6686818-6709080 // 100.0
1641490_s_at	1	1	chr2L:6689617-6709085 // 100.0

tutl

	Ab.vs.FRT	AbSCRIB.vs.FRT	Alignments
1627602_at	1	1	chr2L:4283150-4318712 // 100.0
1636076_a_at	1	0	chr2L:4283150-4318638 // 100.0
1637631_at	0	-1	chr2L:4283150-4316071 // 100.0
1639004_at	0	0	chr2L:4283150-4305695 // 99.36
1641128_a_at	0	0	chr2L:4283150-4305695 // 99.36

Usf

	Ab.vs.FRT	AbSCRIB.vs.FRT	Alignments
1626375_a_at	0	-1	chrX:4440552-4443291 // 100.0
1639393_at	0	1	chrX:4440552-4443291 // 100.0

yemalpha

	Ab.vs.FRT	AbSCRIB.vs.FRT	Alignments
1629017_s_at	-1	-1	chr3R:24944306-24944537 // 100.0
1634784_at	-1	-1	chr3R:24944051-24948597 // 99.91

yuri

	Ab.vs.FRT	AbSCRIB.vs.FRT	Alignments
1636288_at	1	1	chr2L:15257336-15264690 // 100.0
1638821_a_at	1	0	chr2L:15261205-15264690 // 100.0

zormin

	Ab.vs.FRT	AbSCRIB.vs.FRT	Alignments
1623197_a_at	0	0	chr3L:2122618-2126614 // 100.0
1626603_at	-1	-1	chr3L:2149711-2150089 // 100.0
1627379_at	0	0	chr3L:2139571-2142104 // 100.0
1628799_at	0	0	chr3L:2137174-2138470 // 100.0
1636349_at	0	0	chr3L:2126924-2128622 // 100.0
1636934_at	0	1	chr3L:2118537-2119187 // 100.0
1640817_at	1	1	chr3L:2128800-2136057 // 100.0
