# Supplementary material for: The BTB-zinc Finger Transcription Factor Abrupt Acts as an Epithelial Oncogene in Drosophila melanogaster through Maintaining a Progenitor-like Cell State
Source: PLoS Genet. 2013 Jul 18;9(7):e1003627. doi: 10.1371/journal.pgen.1003627 (PMC3715428; doi:10.1371/journal.pgen.1003627)
Supplement: Dataset S4 — ChIP-Seq peaks aligned to the genome for selected genes within Classes 1 to 4. Only genes depicted in Figure 2E are shown. Genes are in alphabetical order, and highlight bars beneath the peak landscape indicate significant peaks in each genotype. (PDF) [file pgen.1003627.s004.pdf]

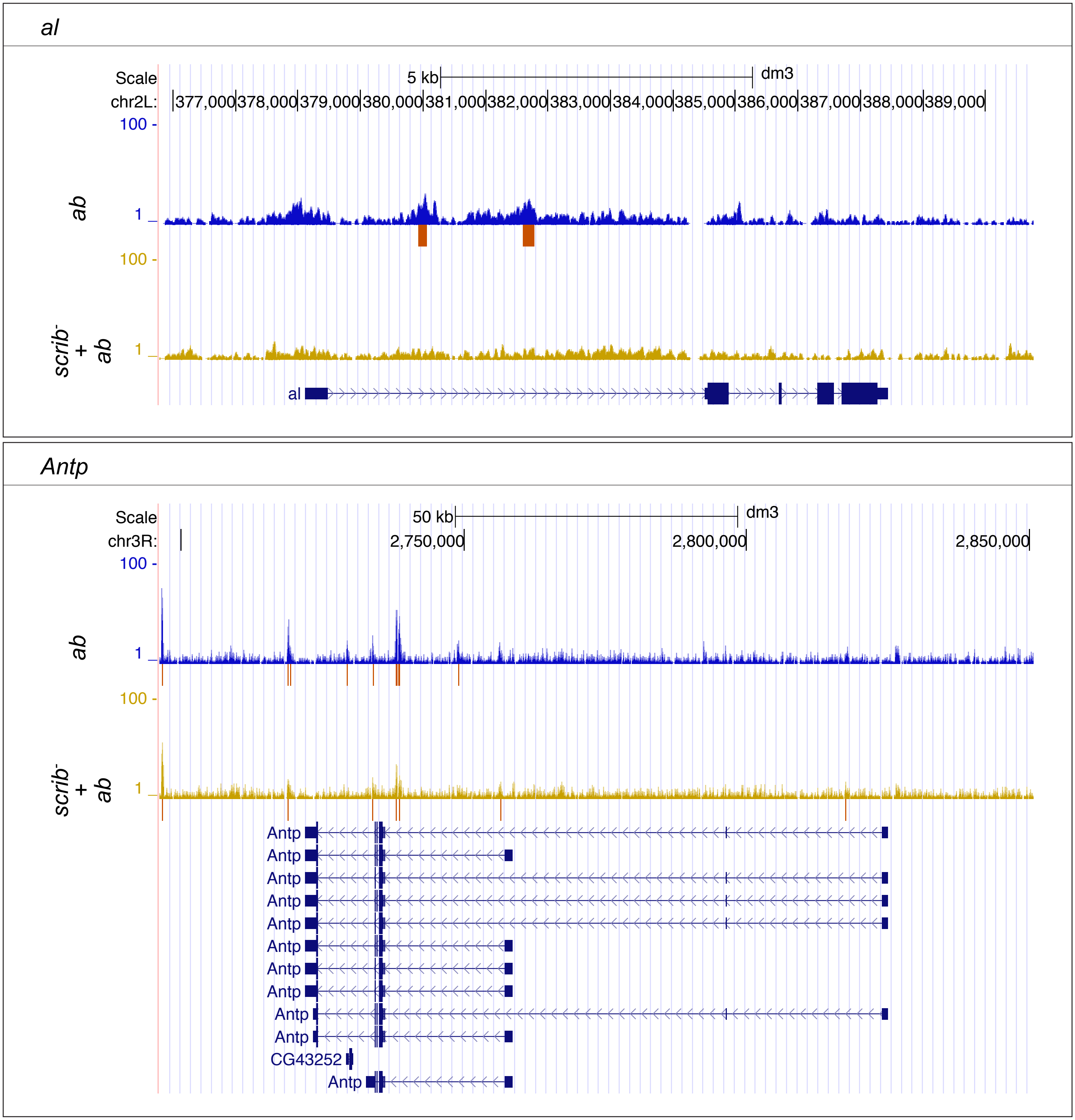
al
Scale
5 kb
dm3
chr2L:
377,000
378,000
379,000
380,000
381,000
382,000
383,000
384,000
385,000
386,000
387,000
388,000
389,000
100 -
ab
1
100 -
scrib⁻ + ab
1
al
Antp
Scale
50 kb
dm3
chr3R:
2,750,000
2,800,000
2,850,000
100 -
ab
1
100 -
scrib⁻ + ab
1
Antp
Antp
Antp
Antp
Antp
Antp
Antp
Antp
Antp
Antp
CG43252
Antp

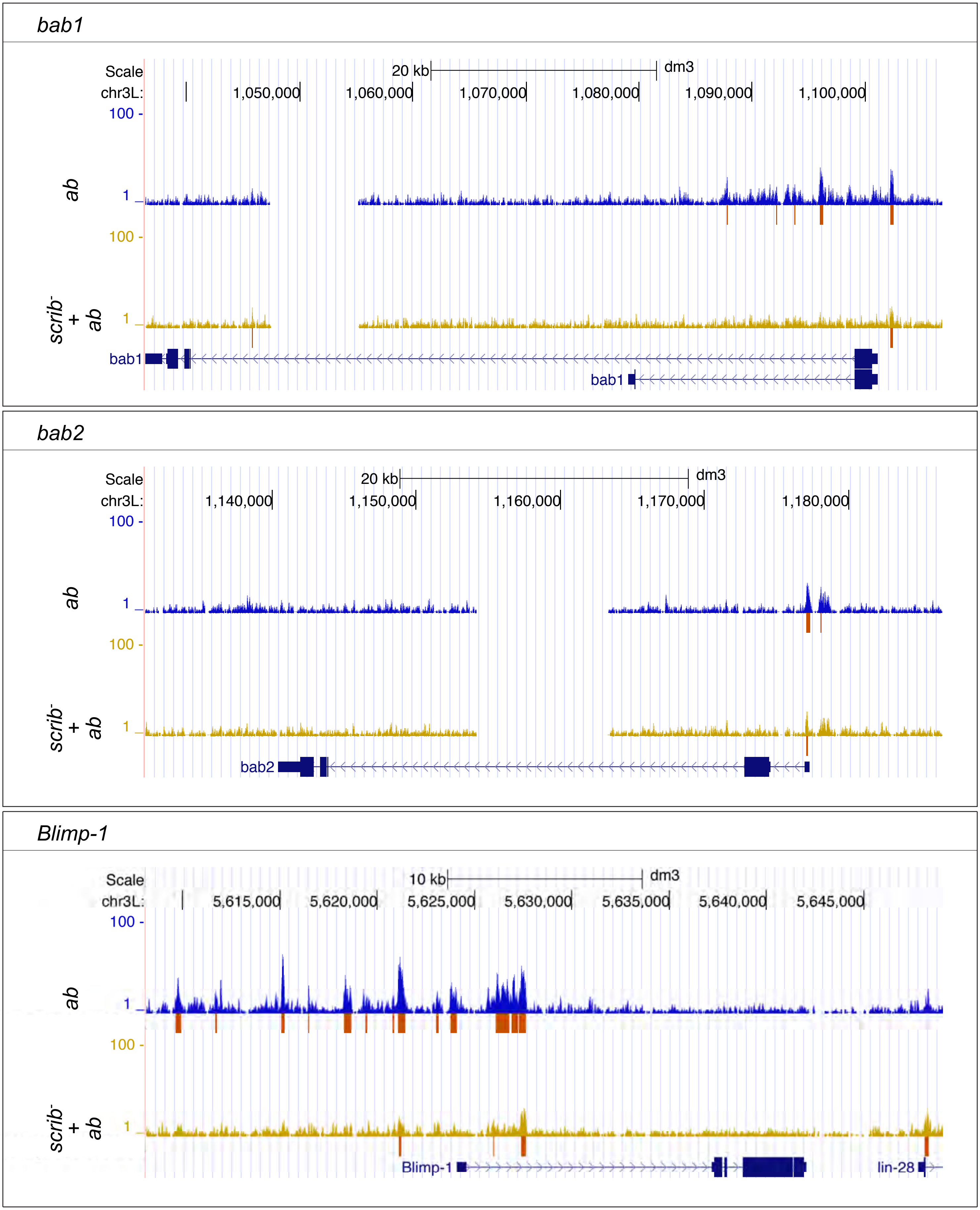
bab1
Scale
chr3L:
20 kb
dm3
1,050,000
1,060,000
1,070,000
1,080,000
1,090,000
1,100,000
100 -
1 _
ab
100 -
1 _
scrib⁻ + ab
bab1
bab1
bab2
Scale
chr3L:
20 kb
dm3
1,140,000
1,150,000
1,160,000
1,170,000
1,180,000
100 -
1 _
ab
100 -
1 _
scrib⁻ + ab
bab2
Blimp-1
Scale
chr3L:
10 kb
dm3
5,615,000
5,620,000
5,625,000
5,630,000
5,635,000
5,640,000
5,645,000
100 -
1 _
ab
100 -
1 _
scrib⁻ + ab
Blimp-1
lin-28

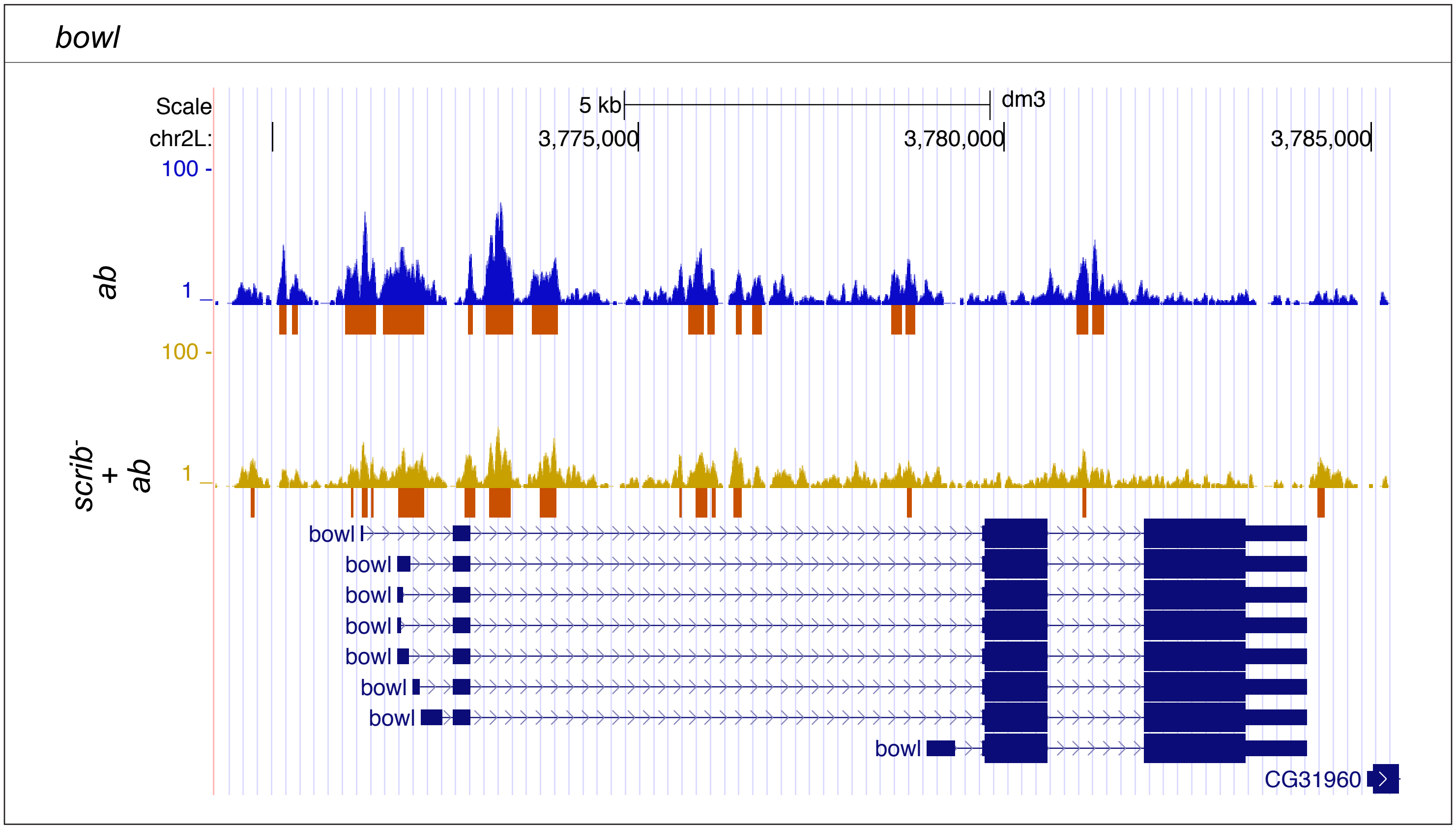
bowl
Scale
5 kb
dm3
chr2L:
3,775,000
3,780,000
3,785,000
100 -
ab
1 _
100 -
scrib- + ab
1 _
bowl
bowl
bowl
bowl
bowl
bowl
bowl
bowl
CG31960

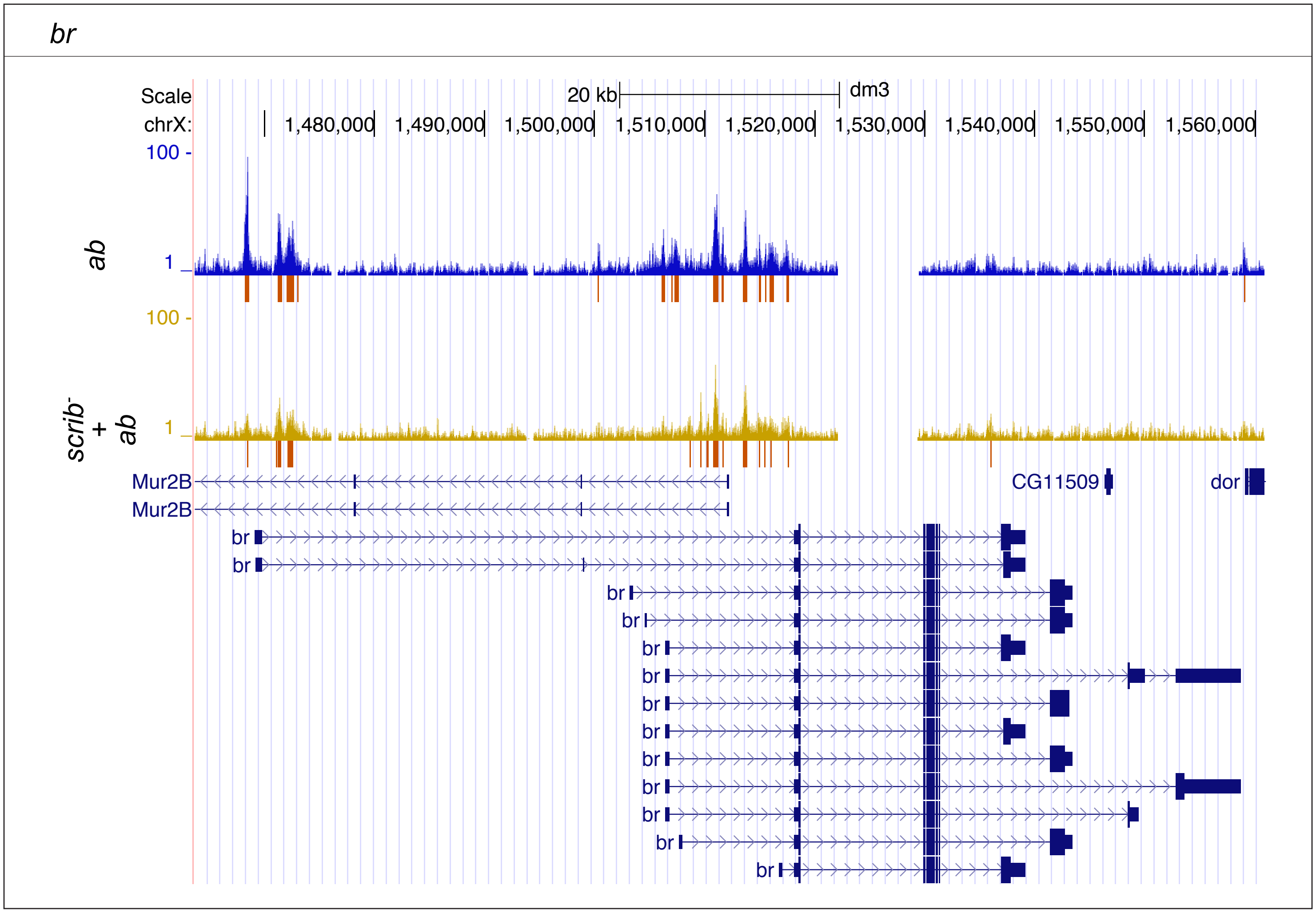
br
Scale
20 kb
dm3
chrX:
1,480,000
1,490,000
1,500,000
1,510,000
1,520,000
1,530,000
1,540,000
1,550,000
1,560,000
100 -
ab
1 _
100 -
scrib- + ab
1 _
Mur2B
Mur2B
CG11509
dor
br
br
br
br
br
br
br
br
br
br
br
br
br

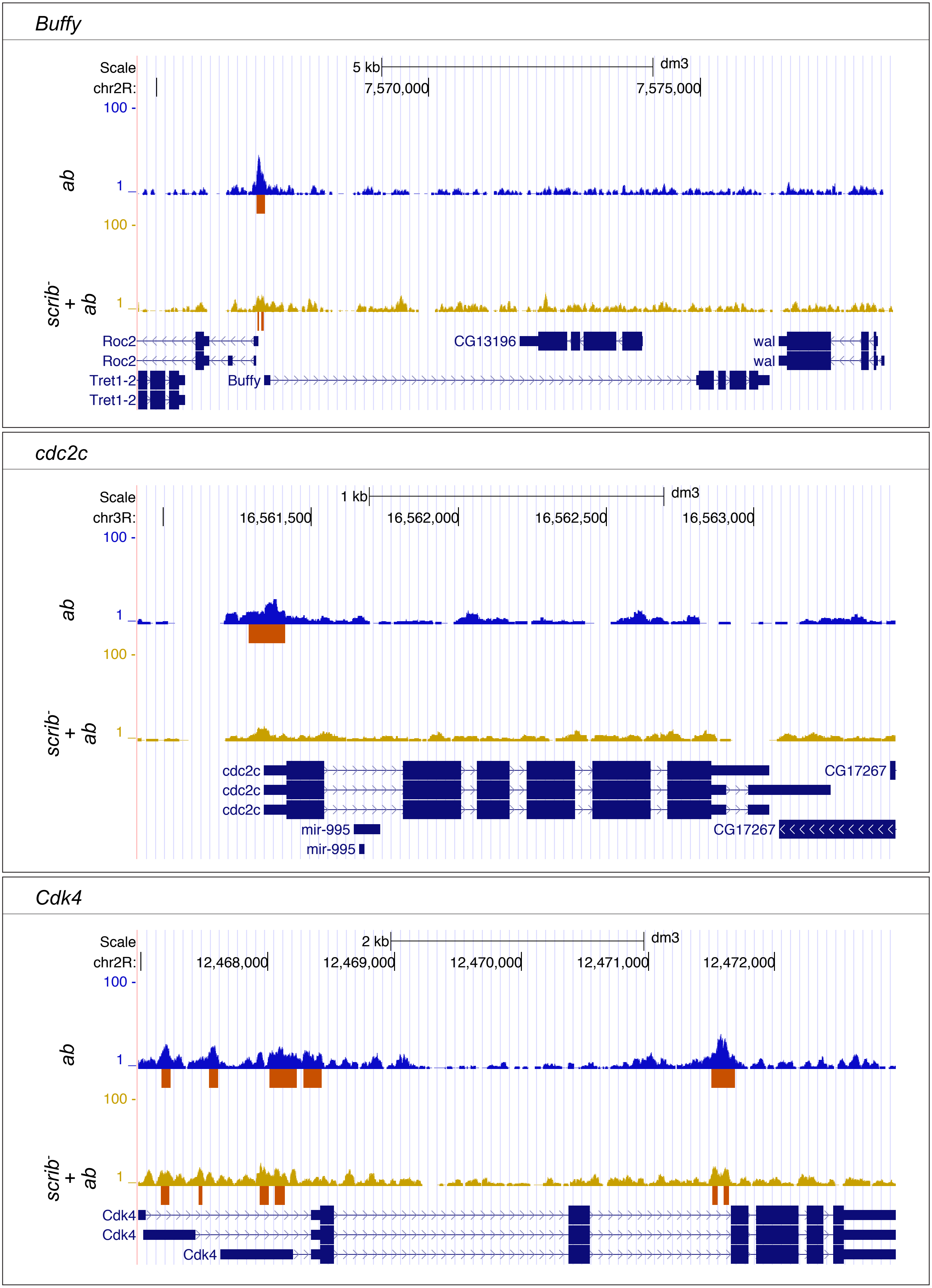

Buffy
Scale
5 kb
dm3
chr2R:
7,570,000
7,575,000
100 -
ab
1
100 -
scrib- + ab
1
Roc2
Roc2
Tret1-2
Tret1-2
Buffy
CG13196
wal
wal
cdc2c
Scale
1 kb
dm3
chr3R:
16,561,500
16,562,000
16,562,500
16,563,000
100 -
ab
1
100 -
scrib- + ab
1
cdc2c
cdc2c
cdc2c
mir-995
mir-995
CG17267
CG17267
Cdk4
Scale
2 kb
dm3
chr2R:
12,468,000
12,469,000
12,470,000
12,471,000
12,472,000
100 -
ab
1
100 -
scrib- + ab
1
Cdk4
Cdk4
Cdk4

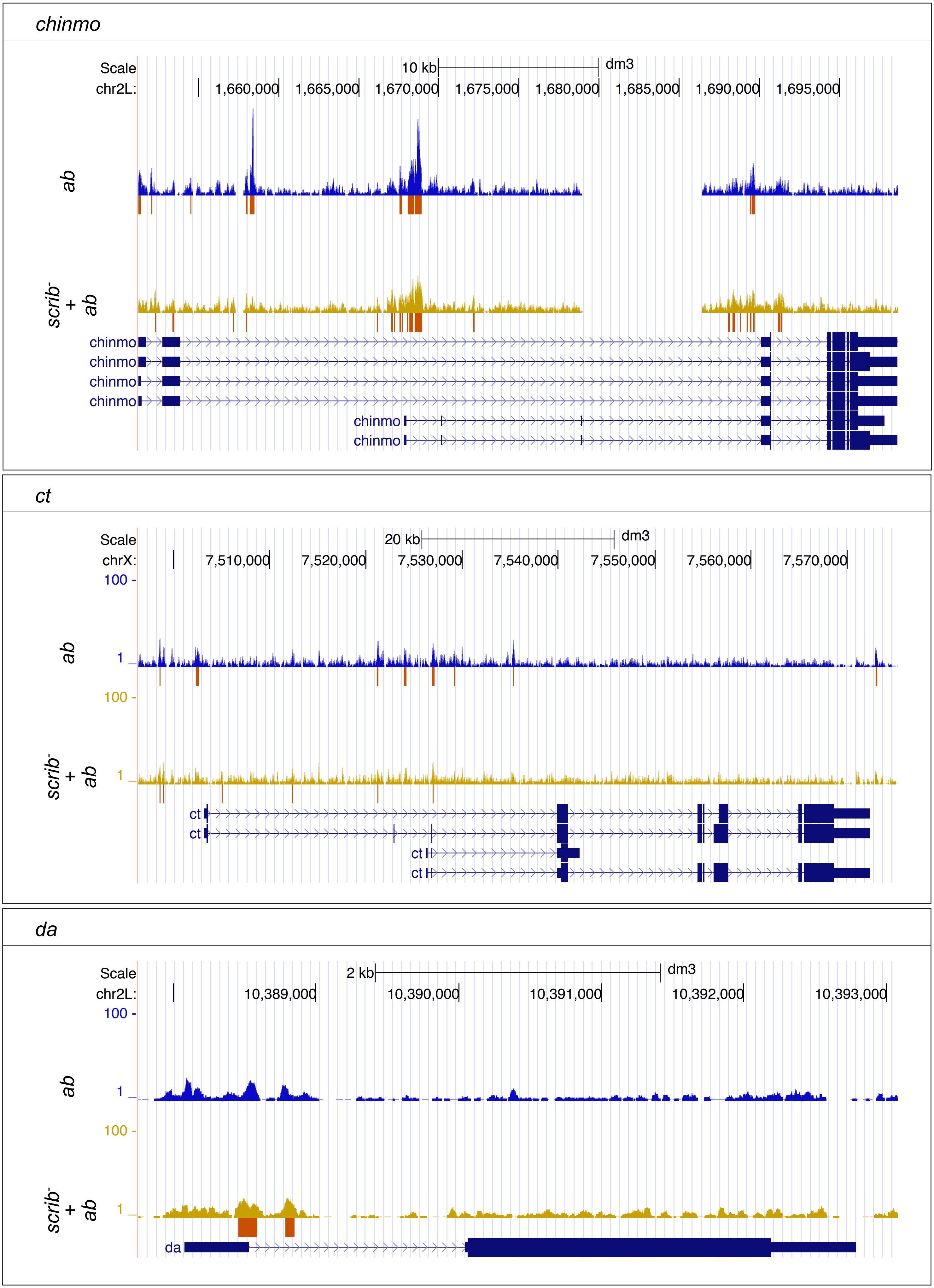
chinmo
Scale
10 kb
dm3
chr2L:
1,660,000
1,665,000
1,670,000
1,675,000
1,680,000
1,685,000
1,690,000
1,695,000
ab
scrib- + ab
chinmo
chinmo
chinmo
chinmo
chinmo
chinmo
ct
Scale
20 kb
dm3
chrX:
7,510,000
7,520,000
7,530,000
7,540,000
7,550,000
7,560,000
7,570,000
100 -
ab
1
100 -
scrib- + ab
1
ct
ct
ct
ct
da
Scale
2 kb
dm3
chr2L:
10,389,000
10,390,000
10,391,000
10,392,000
10,393,000
100 -
ab
1
100 -
scrib- + ab
1
da

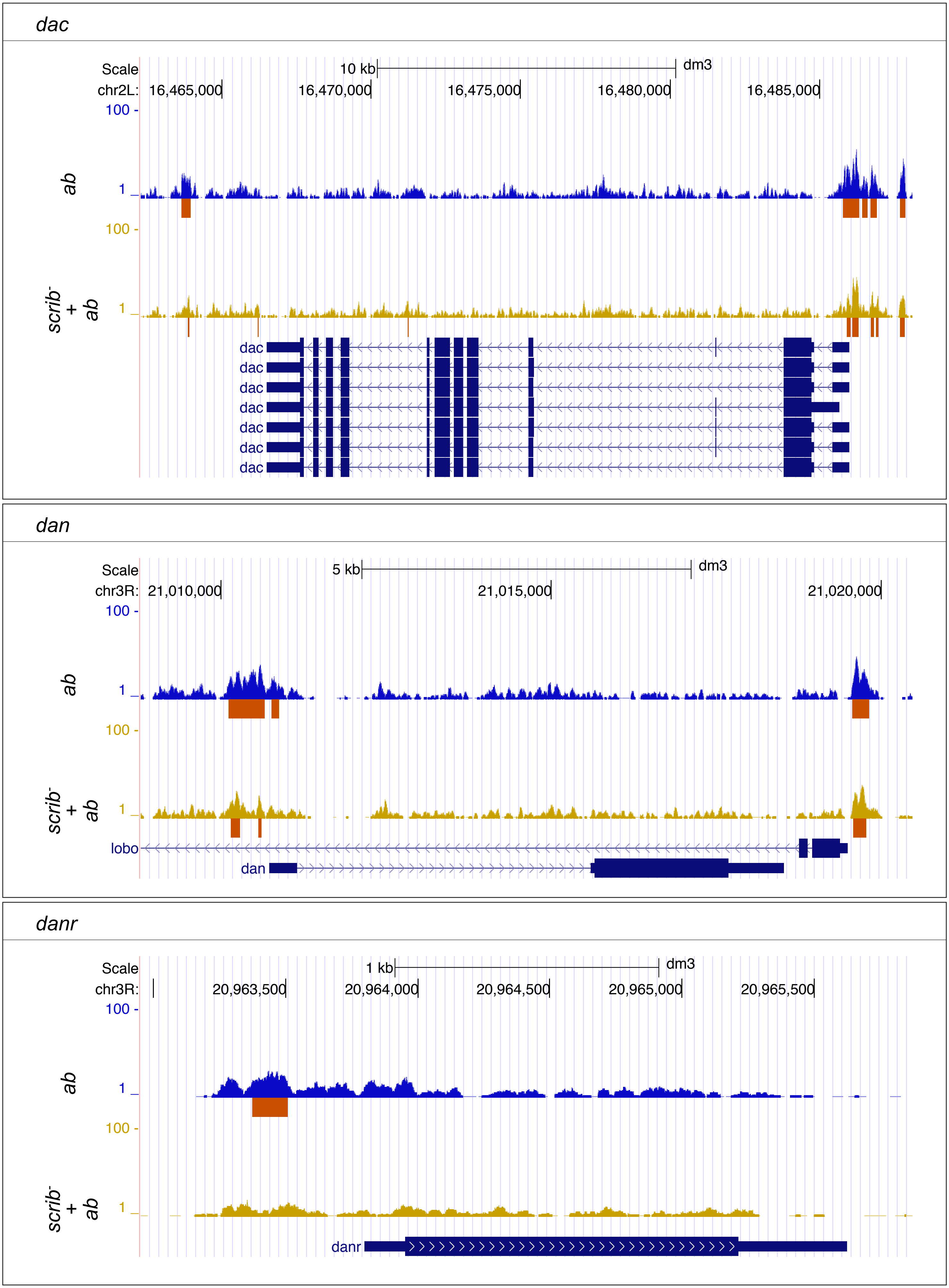

dac
Scale
chr2L:
10 kb
dm3
16,465,000
16,470,000
16,475,000
16,480,000
16,485,000
100 -
ab
1
100 -
scrib- + ab
1
dac
dac
dac
dac
dac
dac
dac
dan
Scale
chr3R:
5 kb
dm3
21,010,000
21,015,000
21,020,000
100 -
ab
1
100 -
scrib- + ab
1
lobo
dan
danr
Scale
chr3R:
1 kb
dm3
20,963,500
20,964,000
20,964,500
20,965,000
20,965,500
100 -
ab
1
100 -
scrib- + ab
1
danr

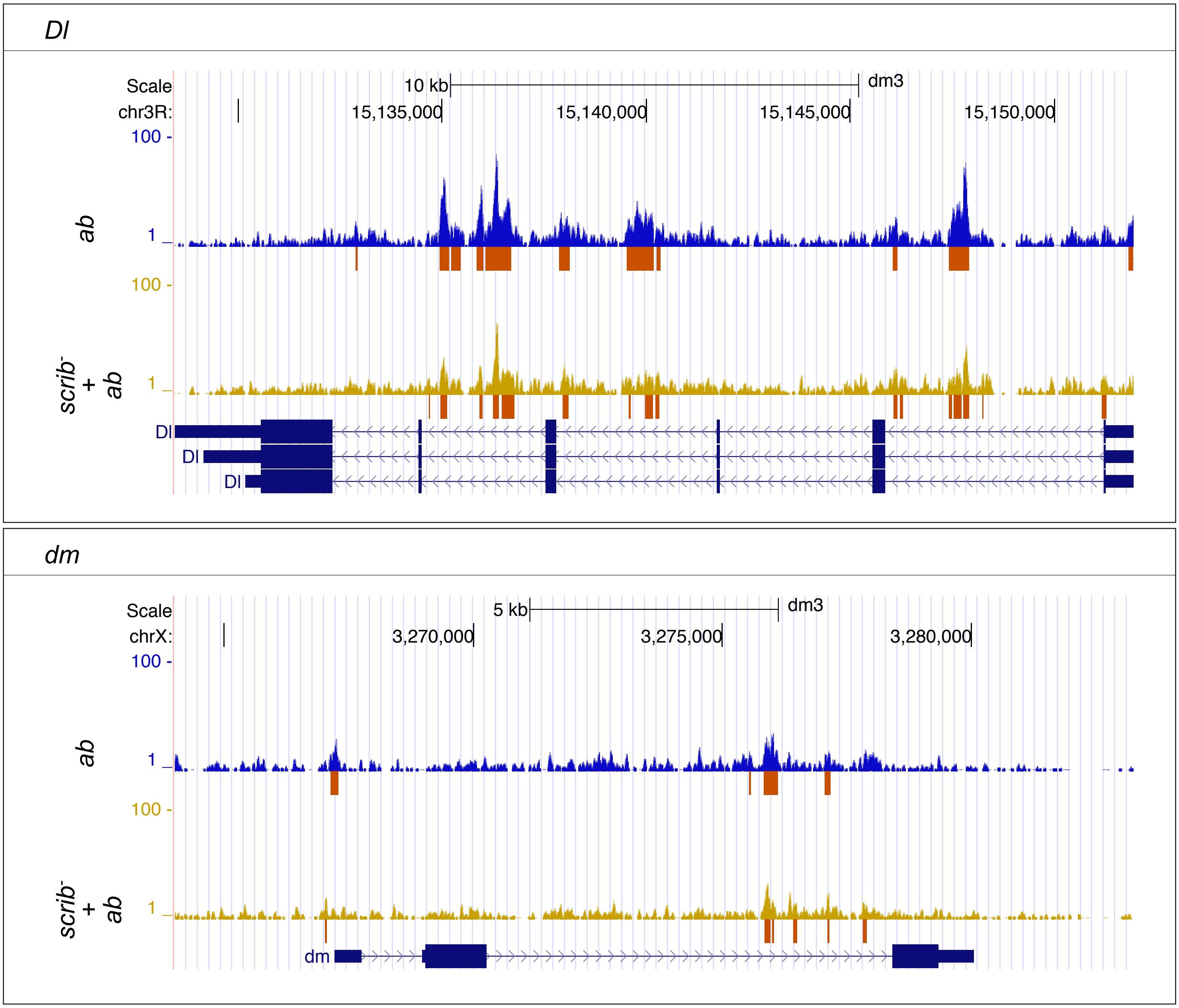

Dl
Scale
10 kb
dm3
chr3R:
15,135,000
15,140,000
15,145,000
15,150,000
100 -
ab
1
100 -
scrib- + ab
1
Dl
Dl
Dl
dm
Scale
5 kb
dm3
chrX:
3,270,000
3,275,000
3,280,000
100 -
ab
1
100 -
scrib- + ab
1
dm

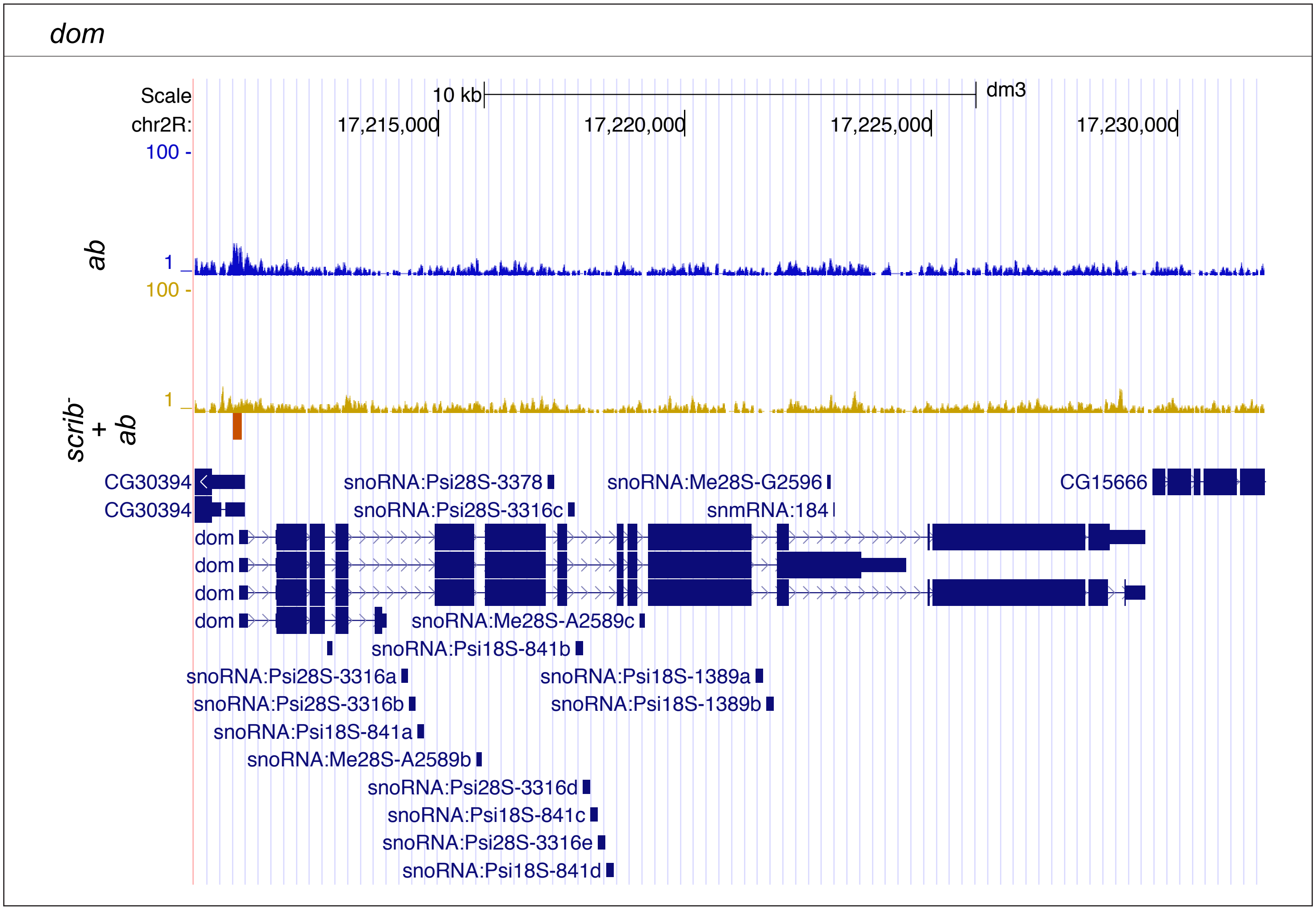
dom
Scale
10 kb
dm3
chr2R:
17,215,000
17,220,000
17,225,000
17,230,000
100 -
ab
1
100 -
scrib- + ab
1
CG30394
CG30394
snoRNA:Psi28S-3378
snoRNA:Psi28S-3316c
snoRNA:Me28S-G2596
snmRNA:184
CG15666
dom
dom
dom
dom
snoRNA:Me28S-A2589c
snoRNA:Psi18S-841b
snoRNA:Psi28S-3316a
snoRNA:Psi28S-3316b
snoRNA:Psi18S-841a
snoRNA:Me28S-A2589b
snoRNA:Psi28S-3316d
snoRNA:Psi18S-841c
snoRNA:Psi28S-3316e
snoRNA:Psi18S-841d
snoRNA:Psi18S-1389a
snoRNA:Psi18S-1389b

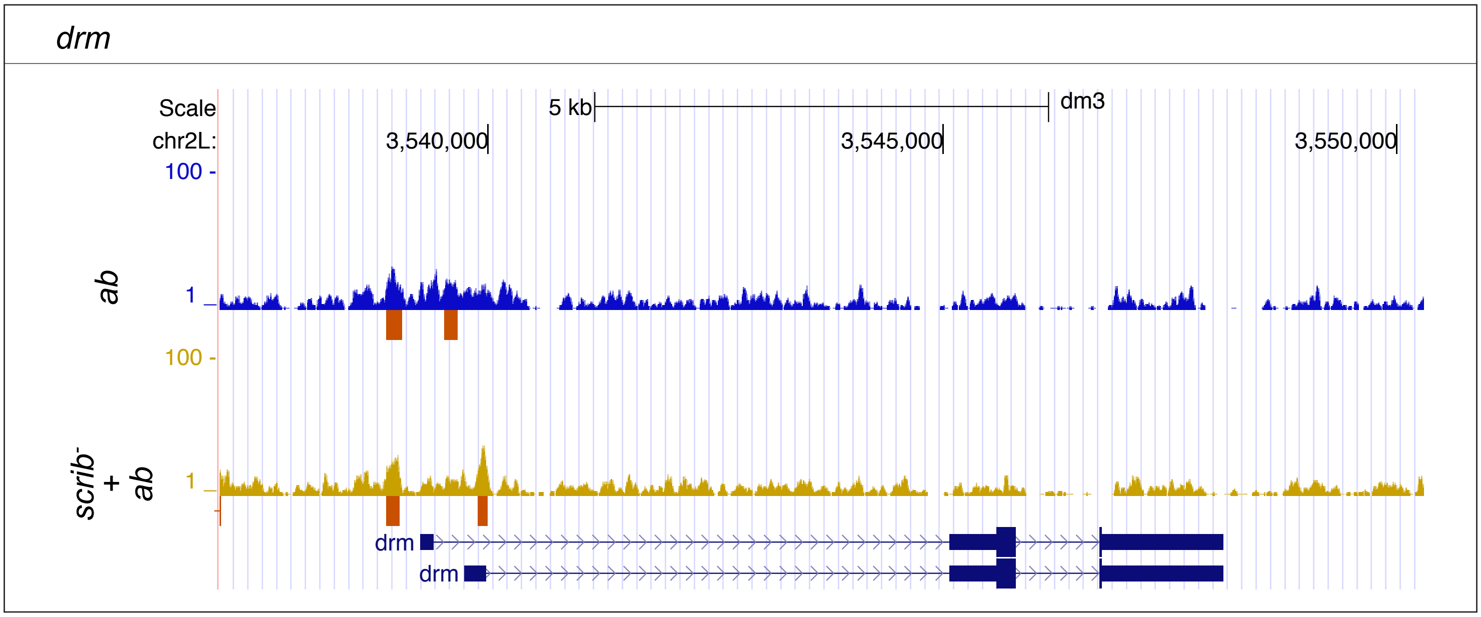
drm
Scale
5 kb
dm3
chr2L:
3,540,000
3,545,000
3,550,000
100 -
ab
1
100 -
scrib- + ab
1
drm
drm

E23

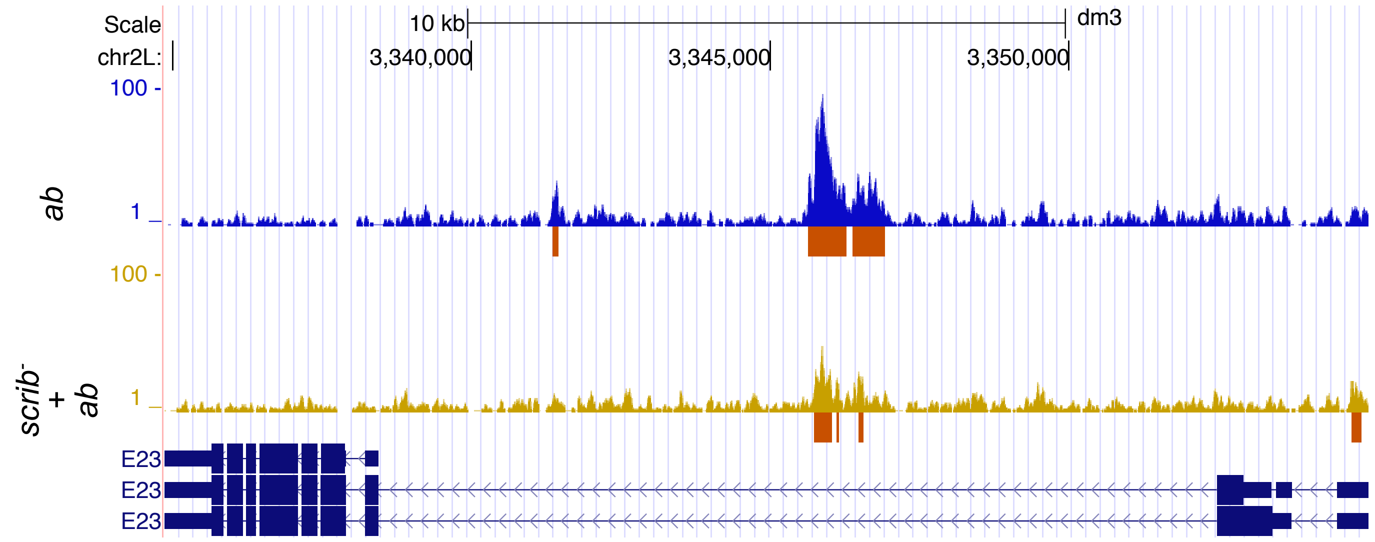
Scale
10 kb
dm3
chr2L:
3,340,000
3,345,000
3,350,000
100 -
1
ab
100 -
1
scrib- + ab
E23
E23
E23

Eip63E

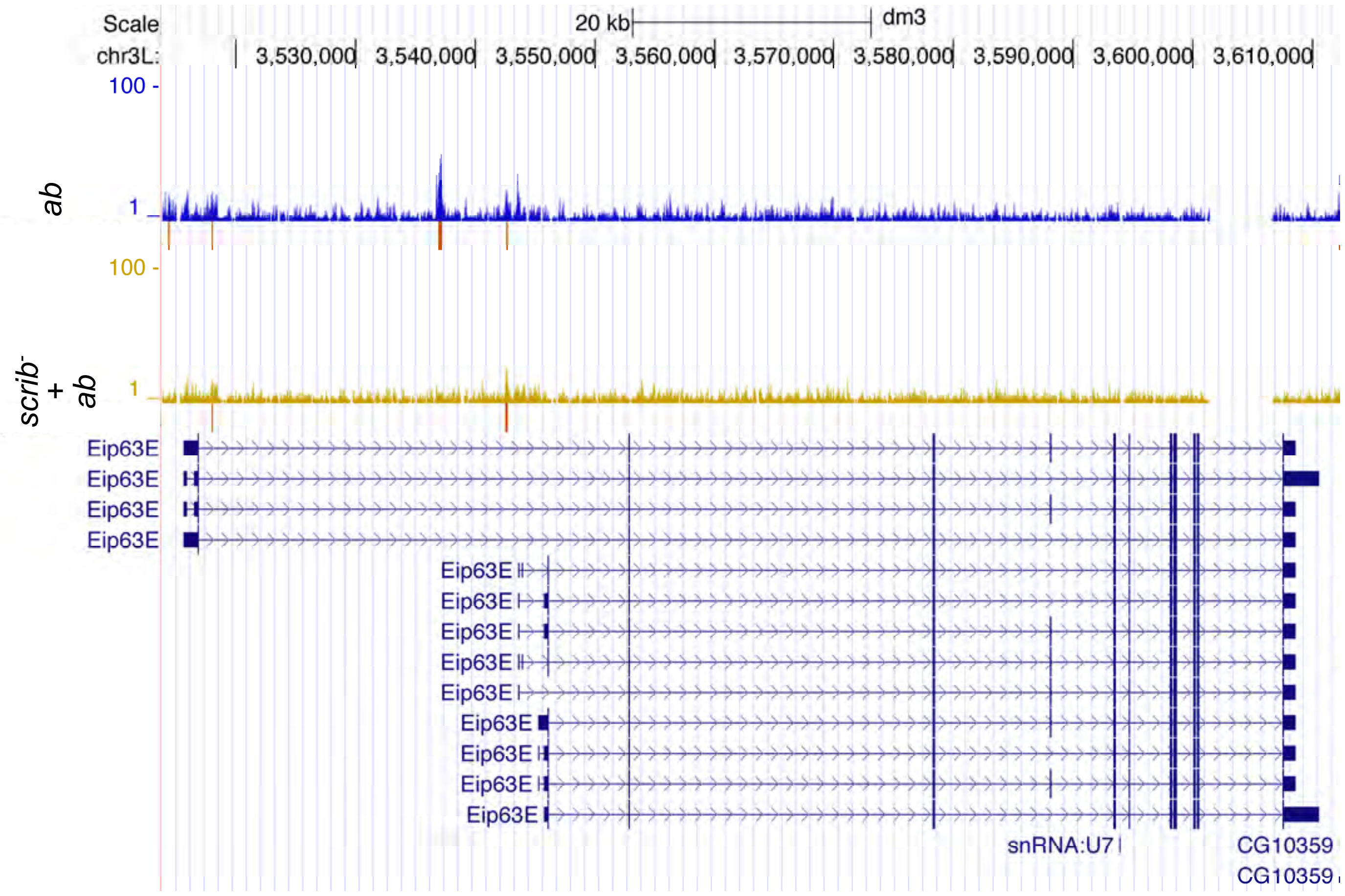
Scale
20 kb
dm3
chr3L:
3,530,000
3,540,000
3,550,000
3,560,000
3,570,000
3,580,000
3,590,000
3,600,000
3,610,000
100 -
1
ab
100 -
1
scrib- + ab
Eip63E
Eip63E
Eip63E
Eip63E
Eip63E
Eip63E
Eip63E
Eip63E
Eip63E
Eip63E
Eip63E
Eip63E
Eip63E
snRNA:U7
CG10359
CG10359

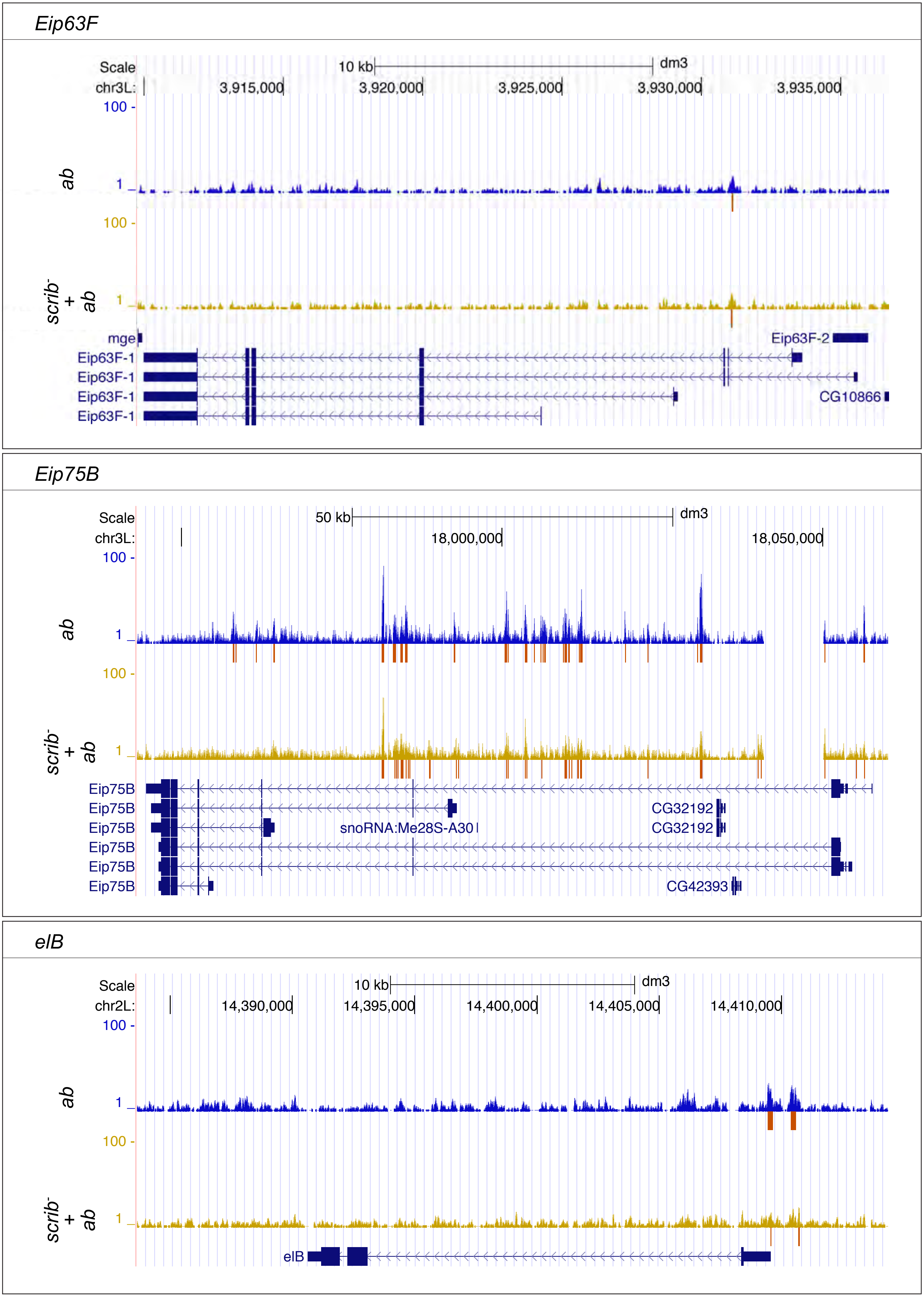
Eip63F
Scale
10 kb
dm3
chr3L:
3,915,000
3,920,000
3,925,000
3,930,000
3,935,000
100 -
ab
1 _
100 -
scrib- + ab
1 _
mge
Eip63F-2
Eip63F-1
Eip63F-1
Eip63F-1
Eip63F-1
CG10866
Eip75B
Scale
50 kb
dm3
chr3L:
18,000,000
18,050,000
100 -
ab
1 _
100 -
scrib- + ab
1 _
Eip75B
Eip75B
Eip75B
Eip75B
Eip75B
Eip75B
CG32192
CG32192
snoRNA:Me28S-A30
CG42393
eIB
Scale
10 kb
dm3
chr2L:
14,390,000
14,395,000
14,400,000
14,405,000
14,410,000
100 -
ab
1 _
100 -
scrib- + ab
1 _
eIB

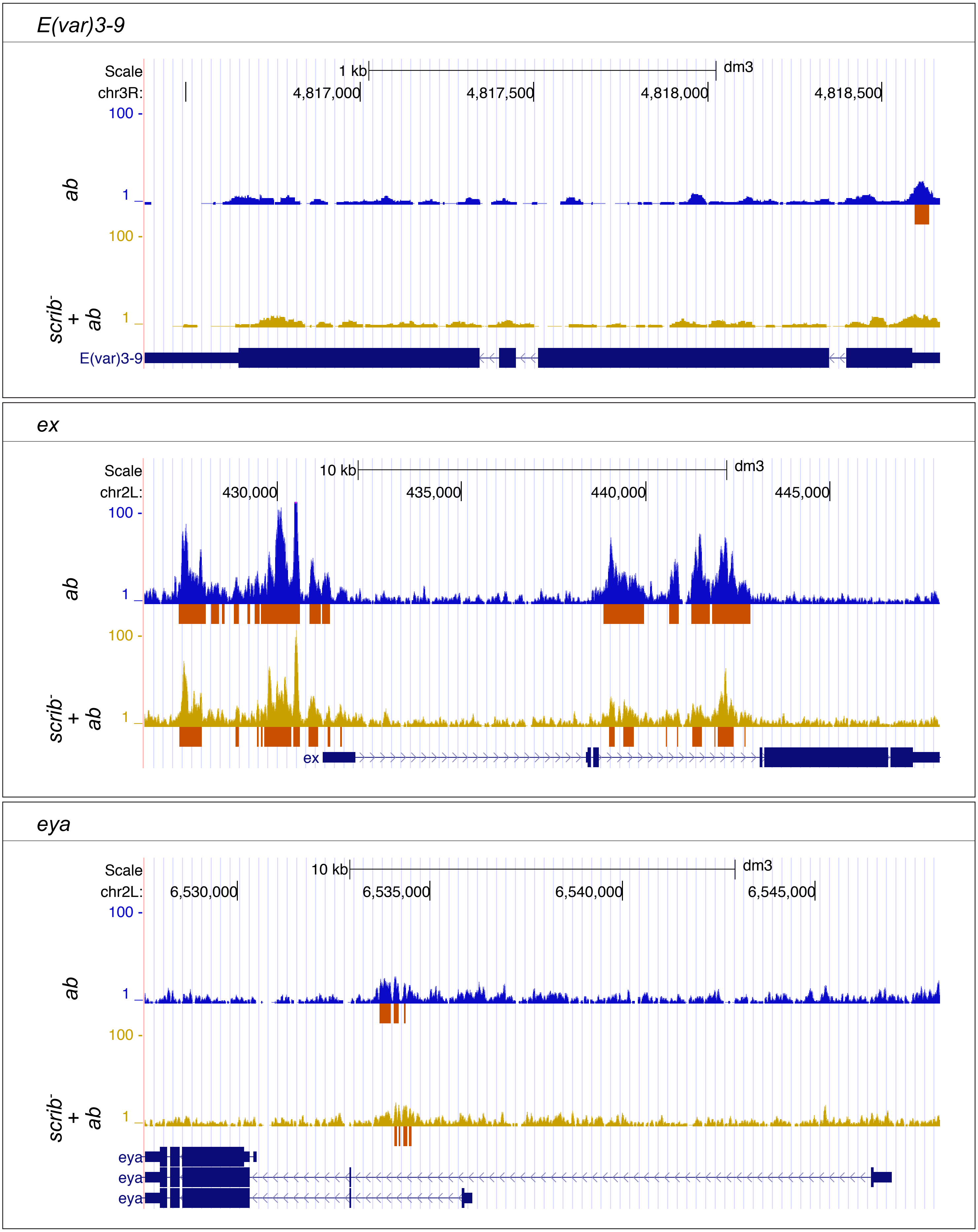
E(var)3-9
Scale
1 kb
dm3
chr3R:
4,817,000
4,817,500
4,818,000
4,818,500
100 -
ab
1
100 -
scrib- + ab
1
E(var)3-9
ex
Scale
10 kb
dm3
chr2L:
430,000
435,000
440,000
445,000
100 -
ab
1
100 -
scrib- + ab
1
ex
eya
Scale
10 kb
dm3
chr2L:
6,530,000
6,535,000
6,540,000
6,545,000
100 -
ab
1
100 -
scrib- + ab
1
eya
eya
eya

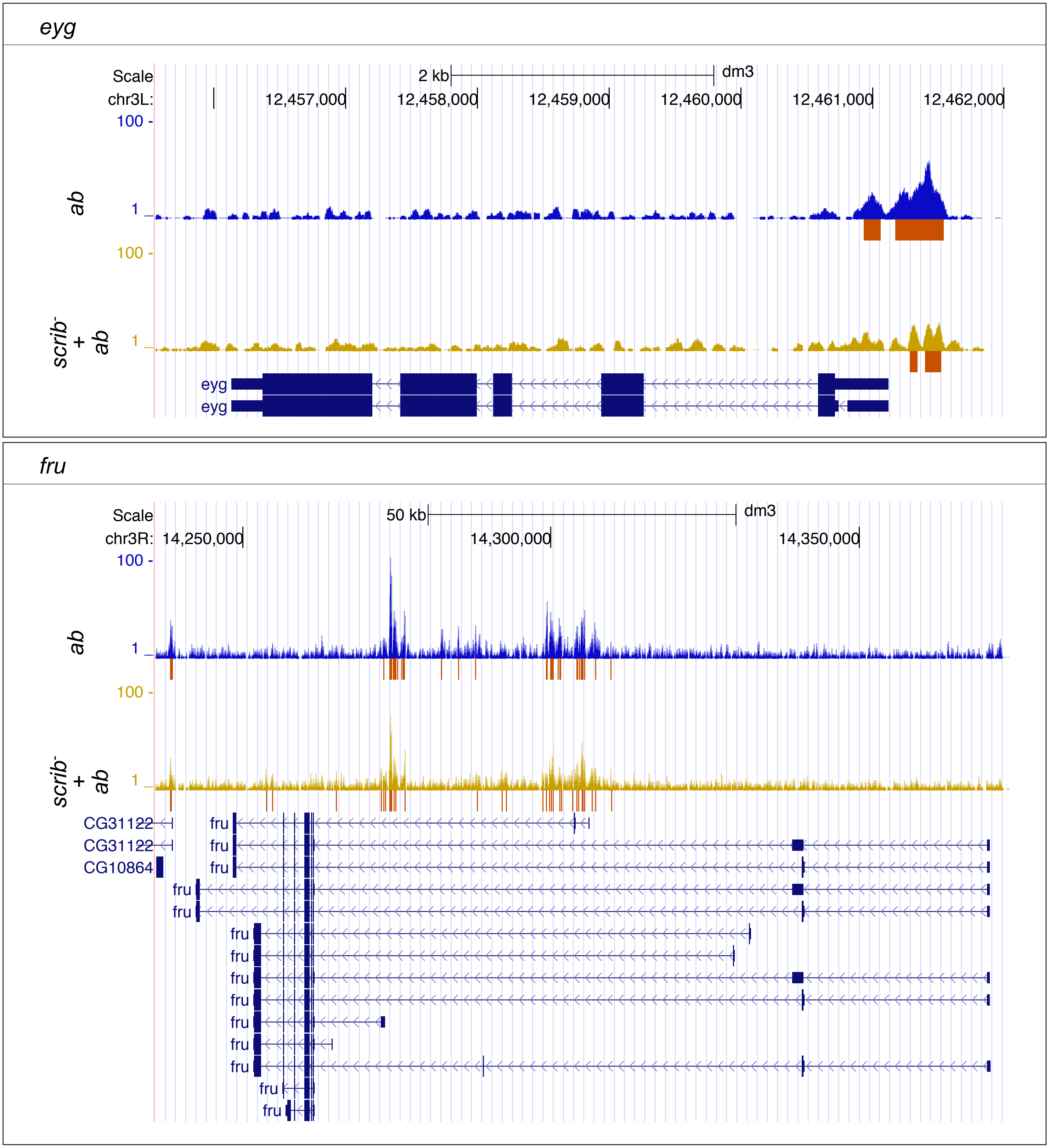
eyg
Scale
2 kb
dm3
chr3L:
12,457,000
12,458,000
12,459,000
12,460,000
12,461,000
12,462,000
100 -
ab
1
100 -
scrib⁻ + ab
1
eyg
eyg
fru
Scale
50 kb
dm3
chr3R:
14,250,000
14,300,000
14,350,000
100 -
ab
1
100 -
scrib⁻ + ab
1
CG31122
CG31122
CG10864
fru

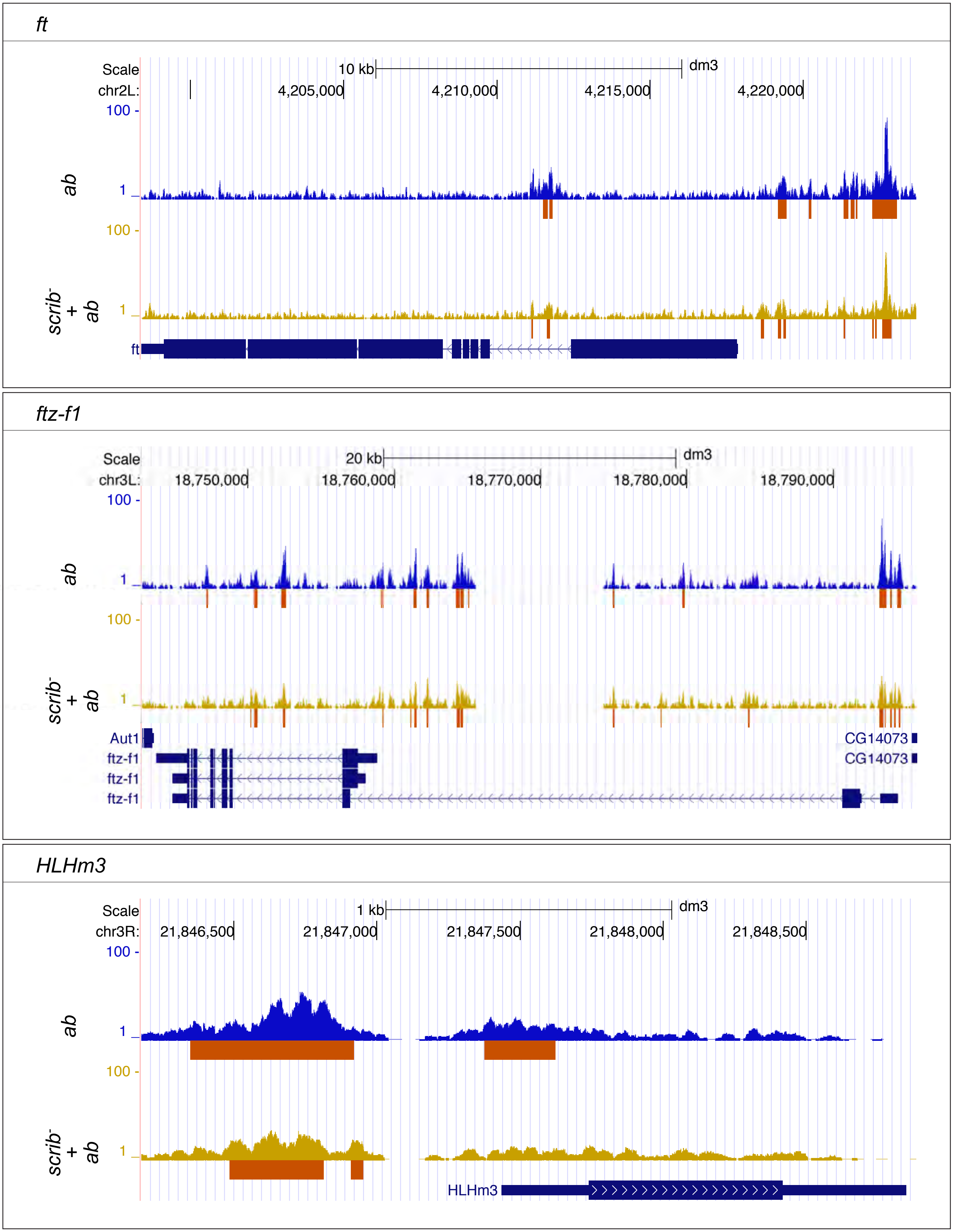
ft
Scale
10 kb
dm3
chr2L:
4,205,000
4,210,000
4,215,000
4,220,000
100 -
1
ab
scrib⁻ + ab
ft
ftz-f1
Scale
20 kb
dm3
chr3L:
18,750,000
18,760,000
18,770,000
18,780,000
18,790,000
Aut1
ftz-f1
CG14073
HLHm3
Scale
1 kb
dm3
chr3R:
21,846,500
21,847,000
21,847,500
21,848,000
21,848,500
HLHm3

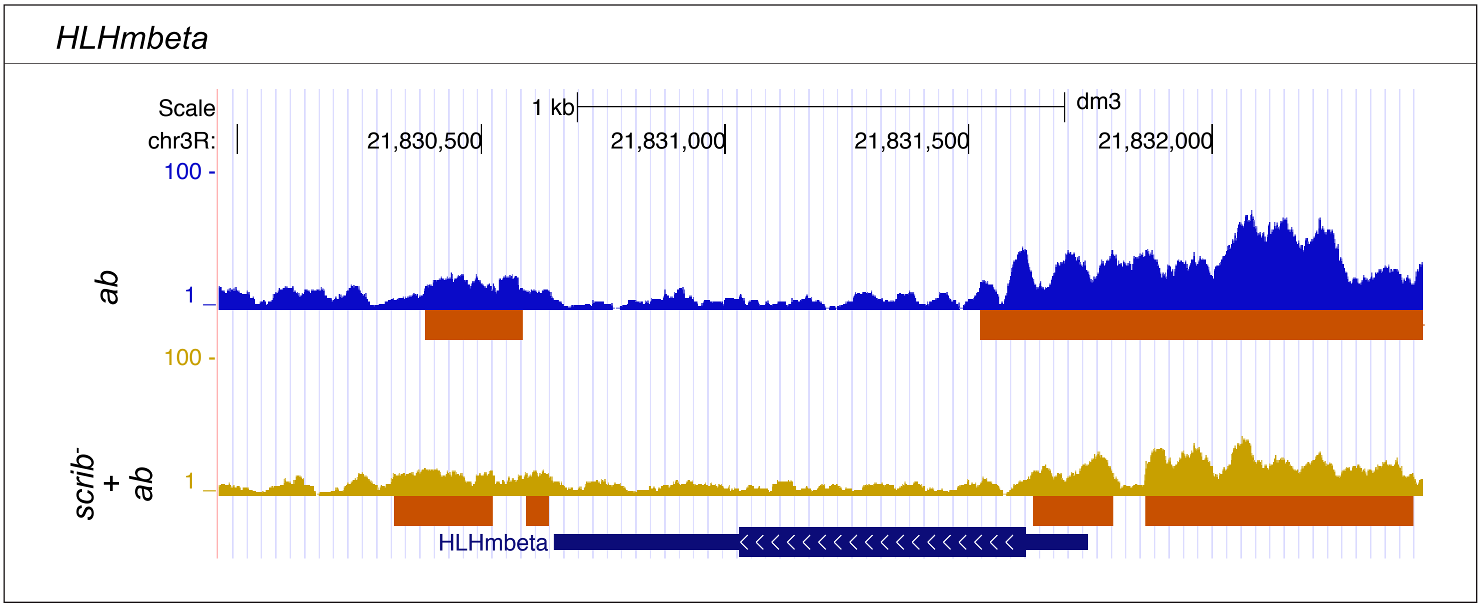
HLHmbeta
Scale
1 kb
dm3
chr3R:
21,830,500
21,831,000
21,831,500
21,832,000
100 -
1 _
ab
100 -
1 _
scrib- + ab
HLHmbeta

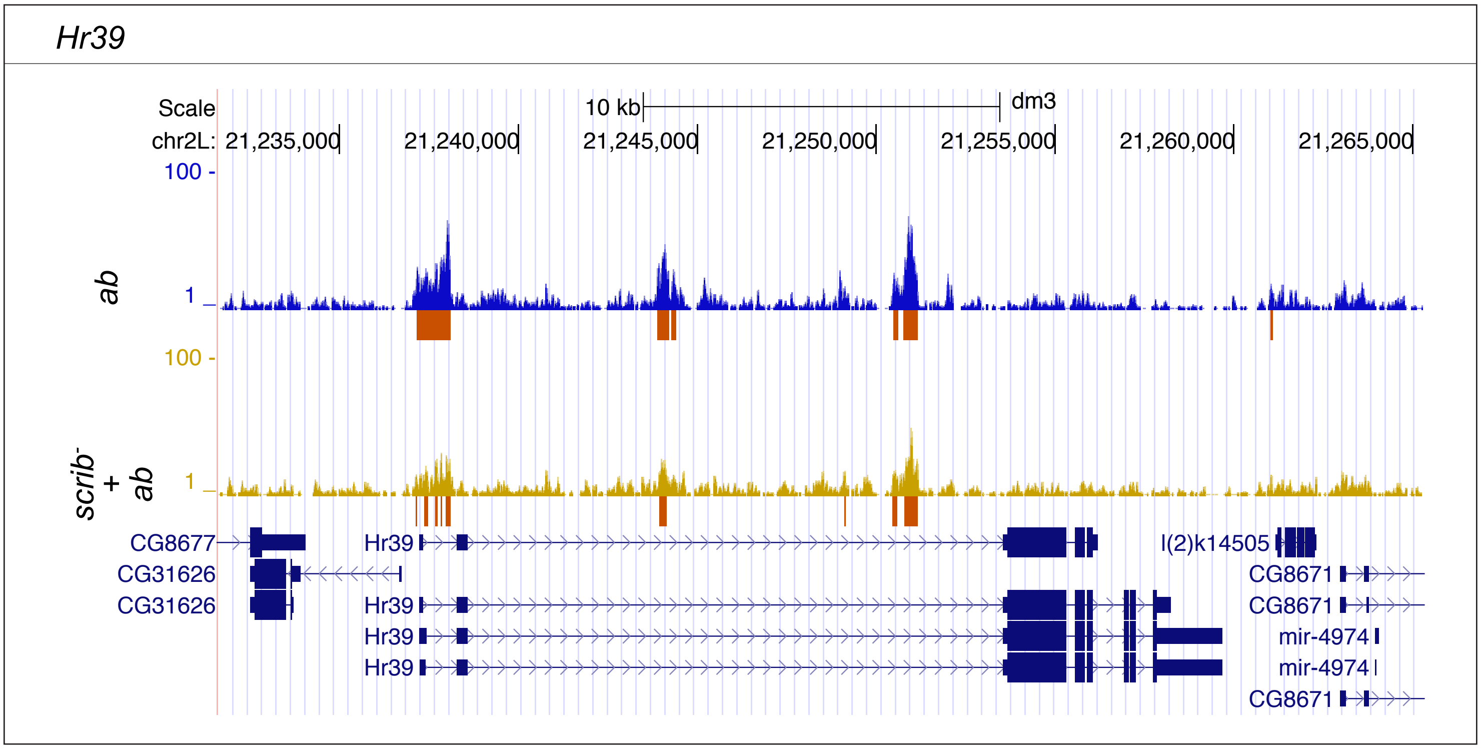
Hr39
Scale
10 kb
dm3
chr2L:
21,235,000
21,240,000
21,245,000
21,250,000
21,255,000
21,260,000
21,265,000
100 -
1 _
ab
100 -
1 _
scrib- + ab
CG8677
CG31626
CG31626
Hr39
Hr39
Hr39
Hr39
l(2)k14505
CG8671
CG8671
mir-4974
mir-4974
CG8671

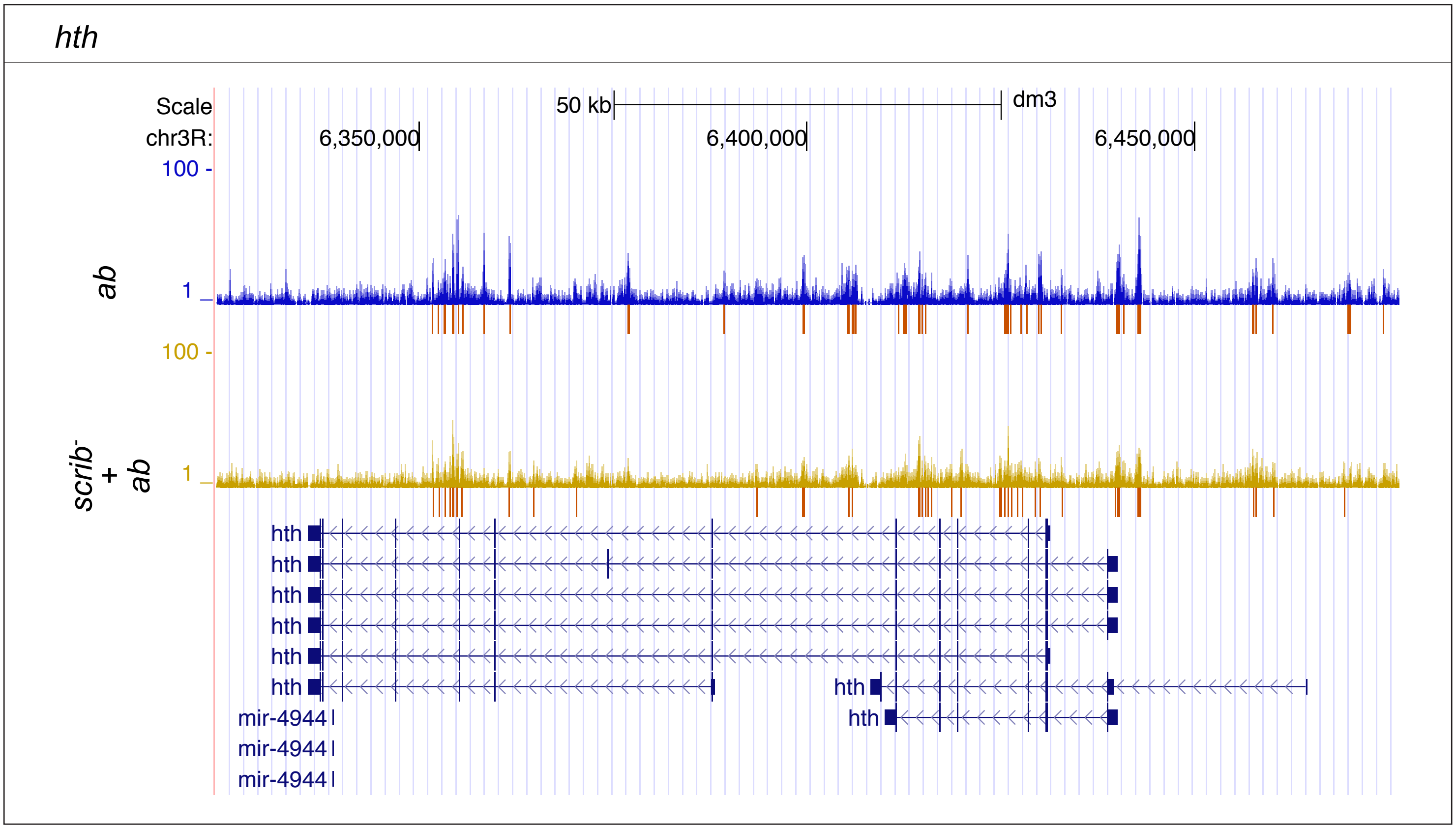

hth
Scale
50 kb
dm3
chr3R:
6,350,000
6,400,000
6,450,000
100 -
ab
1
100 -
scrib- + ab
1
hth
hth
hth
hth
hth
hth
hth
hth
mir-4944
mir-4944
mir-4944

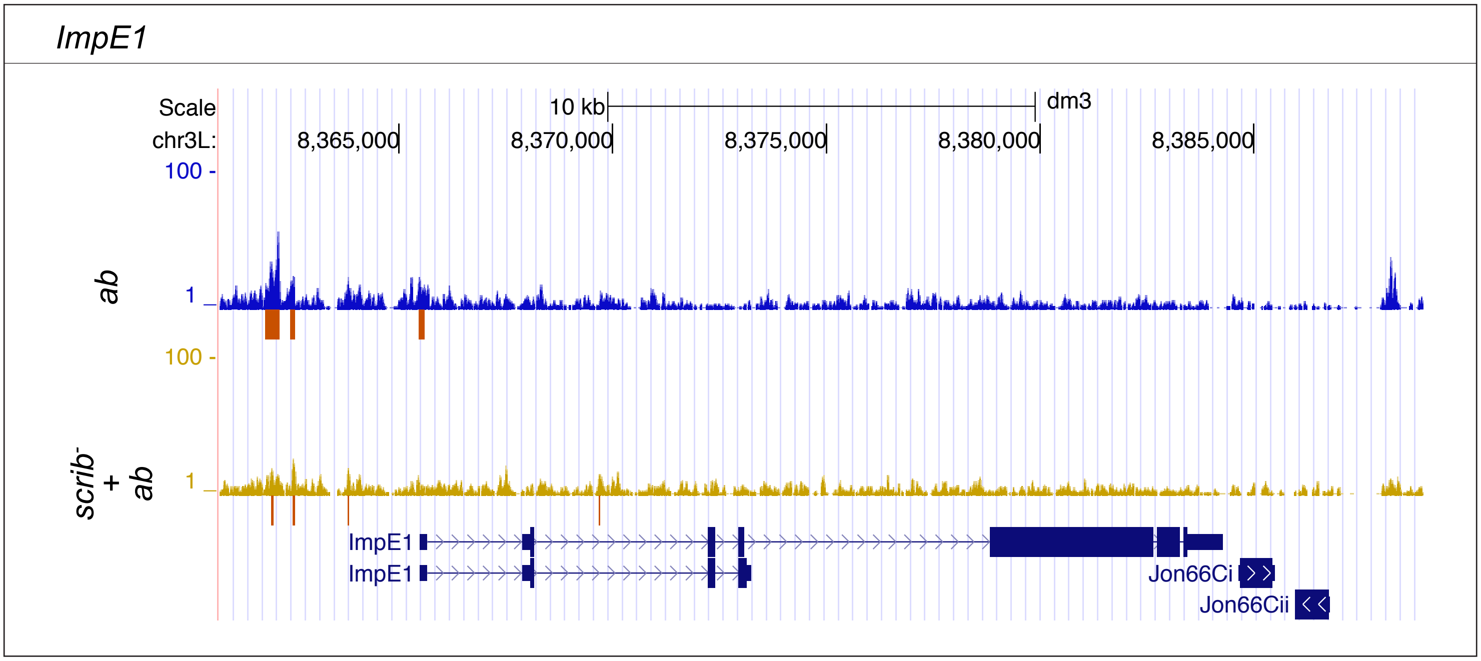

ImpE1
Scale
10 kb
dm3
chr3L:
8,365,000
8,370,000
8,375,000
8,380,000
8,385,000
100 -
ab
1
100 -
scrib- + ab
1
ImpE1
ImpE1
Jon66Ci
Jon66Cii

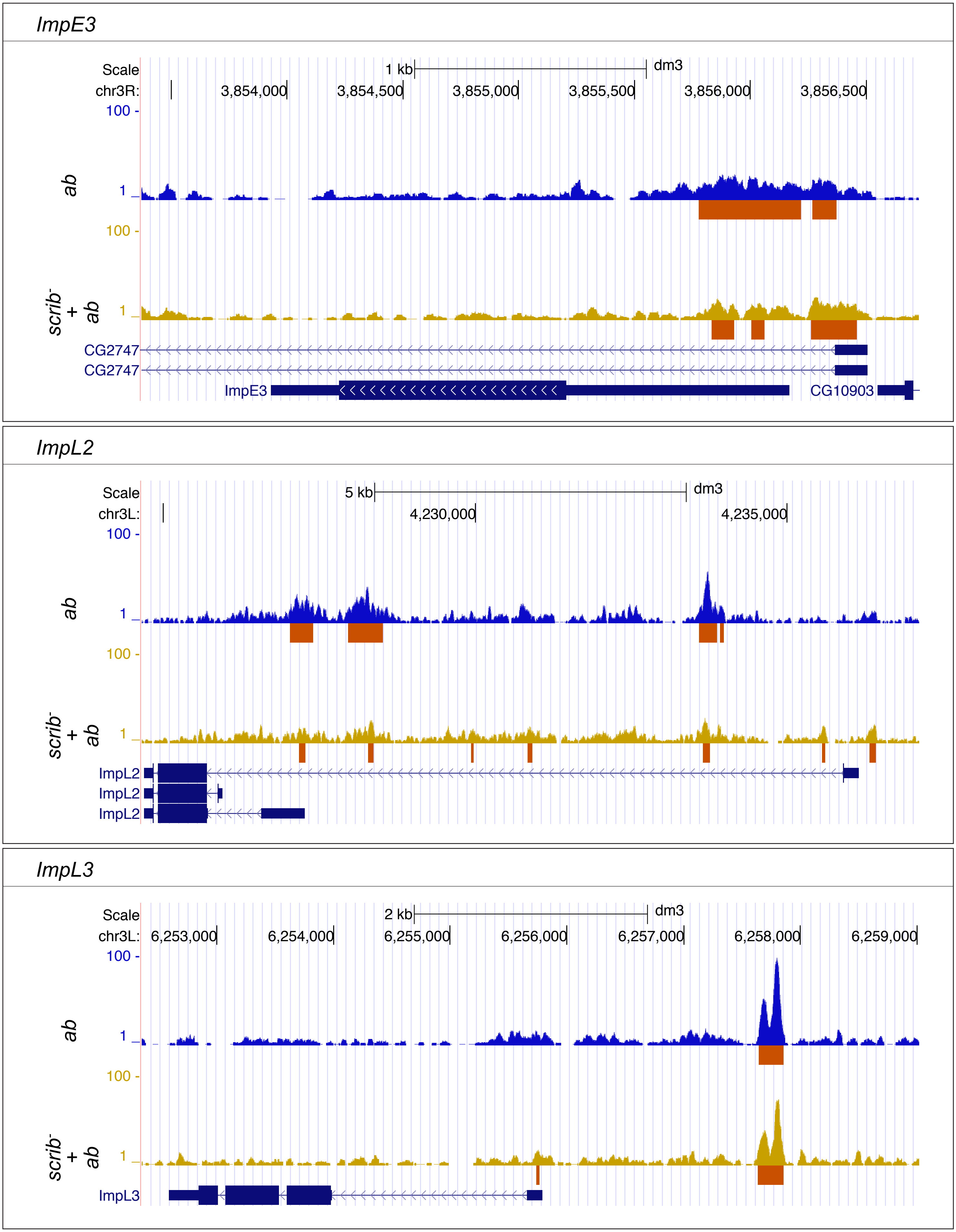
ImpE3
Scale
1 kb
dm3
chr3R:
3,854,000
3,854,500
3,855,000
3,855,500
3,856,000
3,856,500
100 -
1 _
ab
100 -
1 _
scrib⁻ + ab
CG2747
CG2747
ImpE3
CG10903
ImpL2
Scale
5 kb
dm3
chr3L:
4,230,000
4,235,000
100 -
1 _
ab
100 -
1 _
scrib⁻ + ab
ImpL2
ImpL2
ImpL2
ImpL3
Scale
2 kb
dm3
chr3L:
6,253,000
6,254,000
6,255,000
6,256,000
6,257,000
6,258,000
6,259,000
100 -
1 _
ab
100 -
1 _
scrib⁻ + ab
ImpL3

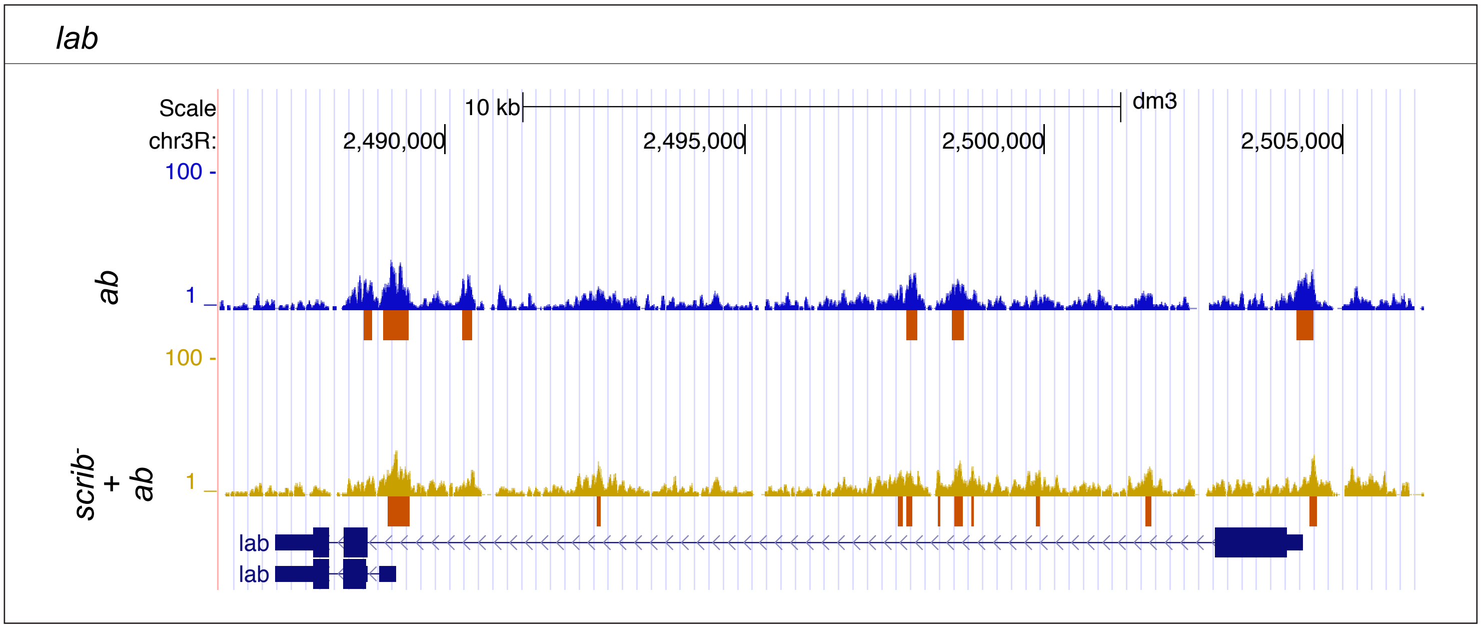
lab
Scale
10 kb
dm3
chr3R:
2,490,000
2,495,000
2,500,000
2,505,000
100 -
ab
1
100 -
scrib⁻ + ab
1
lab
lab

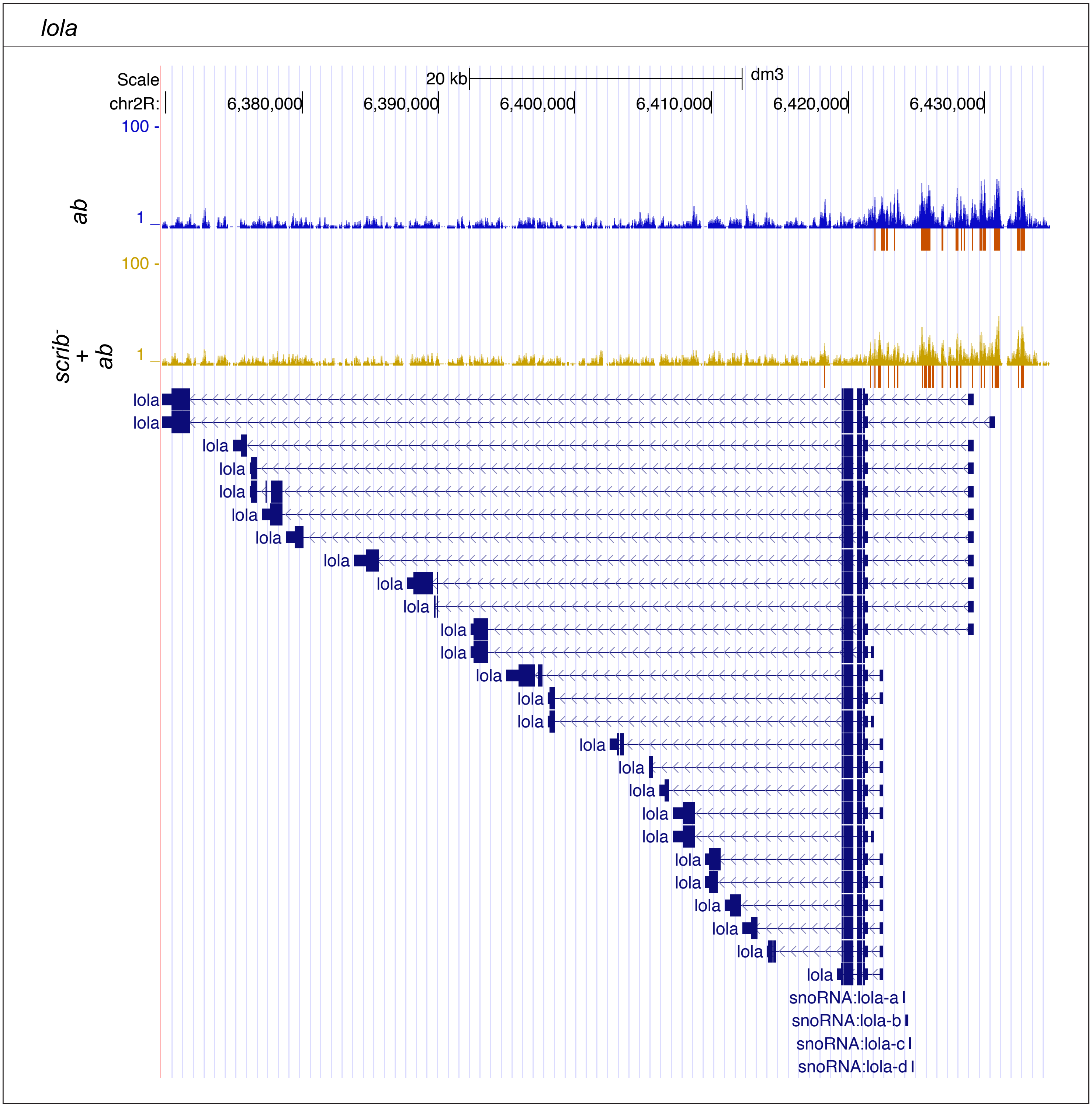
lola
Scale
20 kb
dm3
chr2R:
6,380,000
6,390,000
6,400,000
6,410,000
6,420,000
6,430,000
100 -
ab
1
100 -
scrib⁻ + ab
1
lola
snoRNA:lola-a
snoRNA:lola-b
snoRNA:lola-c
snoRNA:lola-d

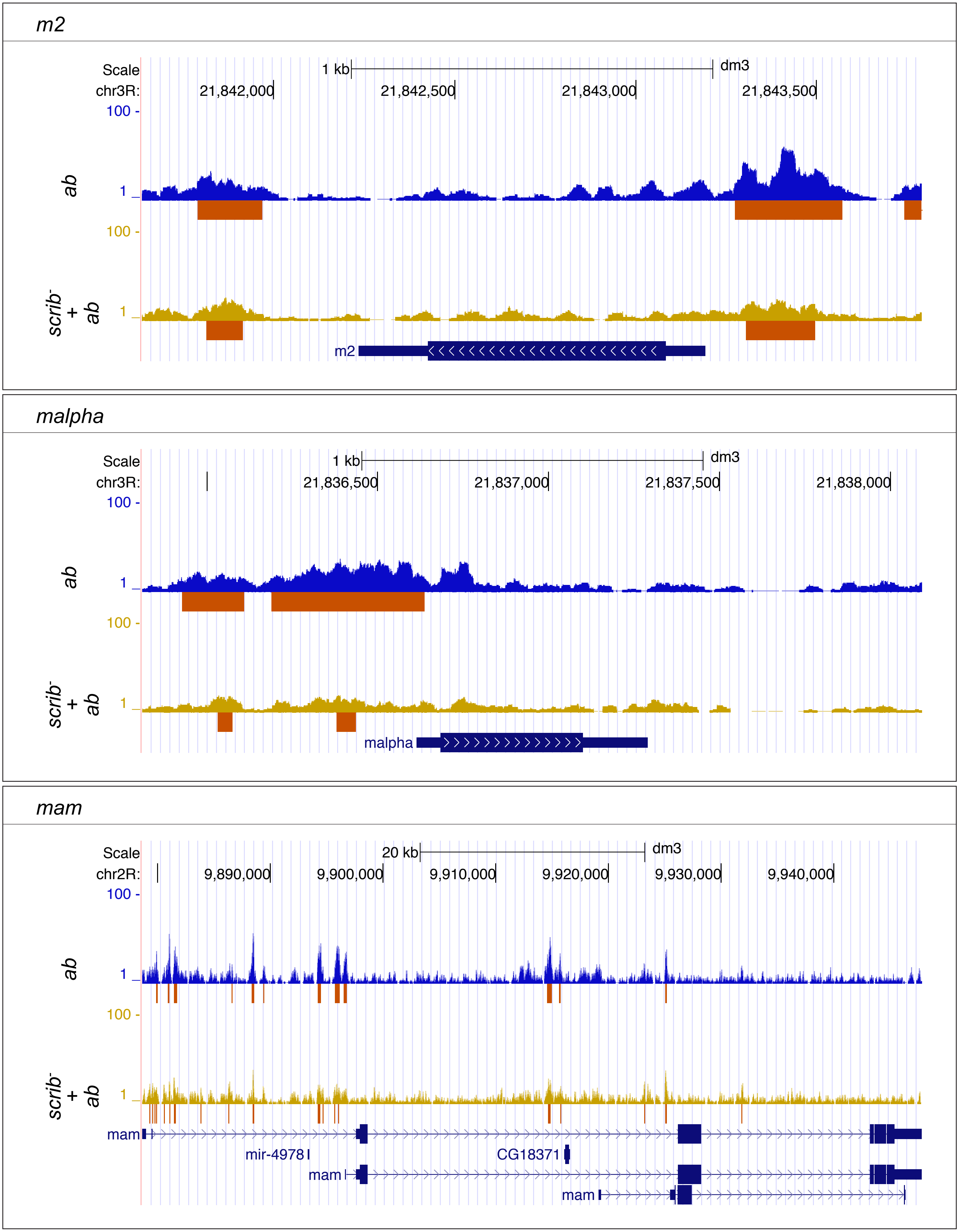
m2
Scale
1 kb
dm3
chr3R:
21,842,000
21,842,500
21,843,000
21,843,500
100 -
ab
1
100 -
scrib- + ab
1
m2
malpha
Scale
1 kb
dm3
chr3R:
21,836,500
21,837,000
21,837,500
21,838,000
100 -
ab
1
100 -
scrib- + ab
1
malpha
mam
Scale
20 kb
dm3
chr2R:
9,890,000
9,900,000
9,910,000
9,920,000
9,930,000
9,940,000
100 -
ab
1
100 -
scrib- + ab
1
mam
mir-4978
CG18371
mam
mam

mamo

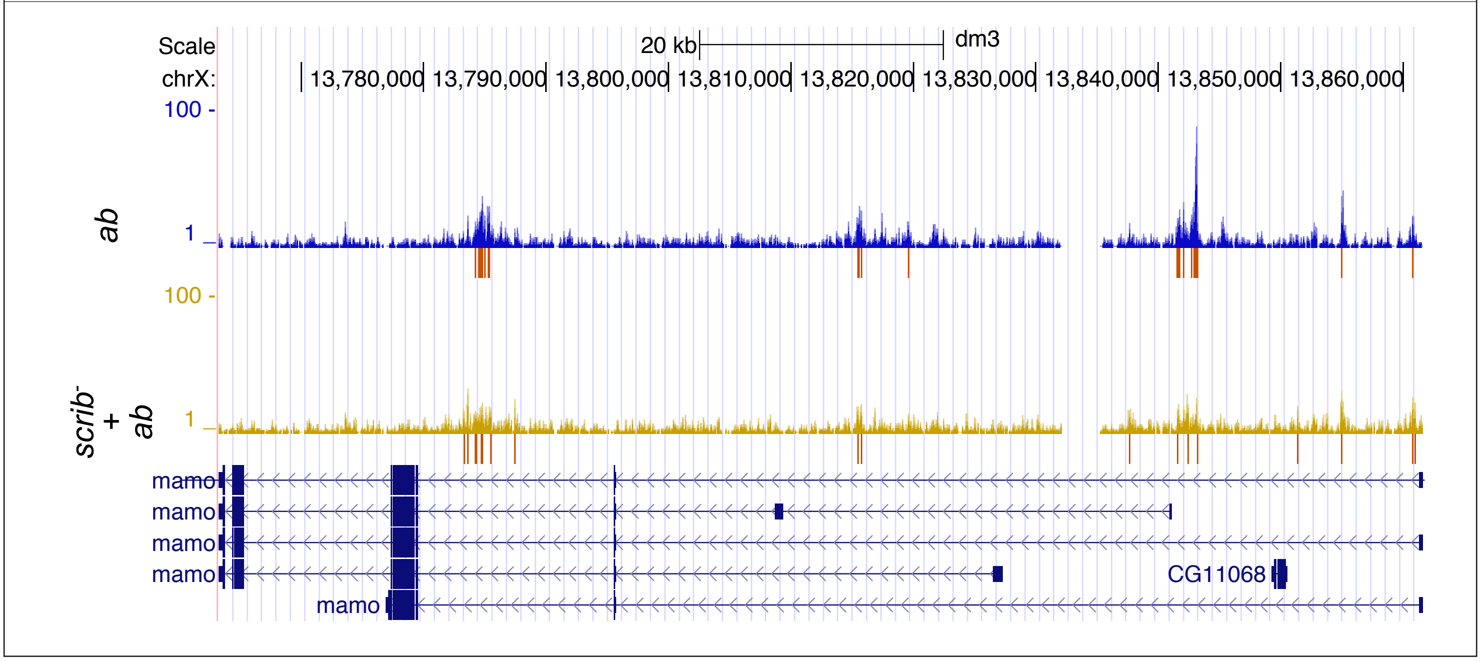

Scale
20 kb
dm3
chrX:
13,780,000
13,790,000
13,800,000
13,810,000
13,820,000
13,830,000
13,840,000
13,850,000
13,860,000
100 -
ab
1
100 -
scrib⁻ + ab
1
mamo
mamo
mamo
mamo
mamo
CG11068

Mmp1

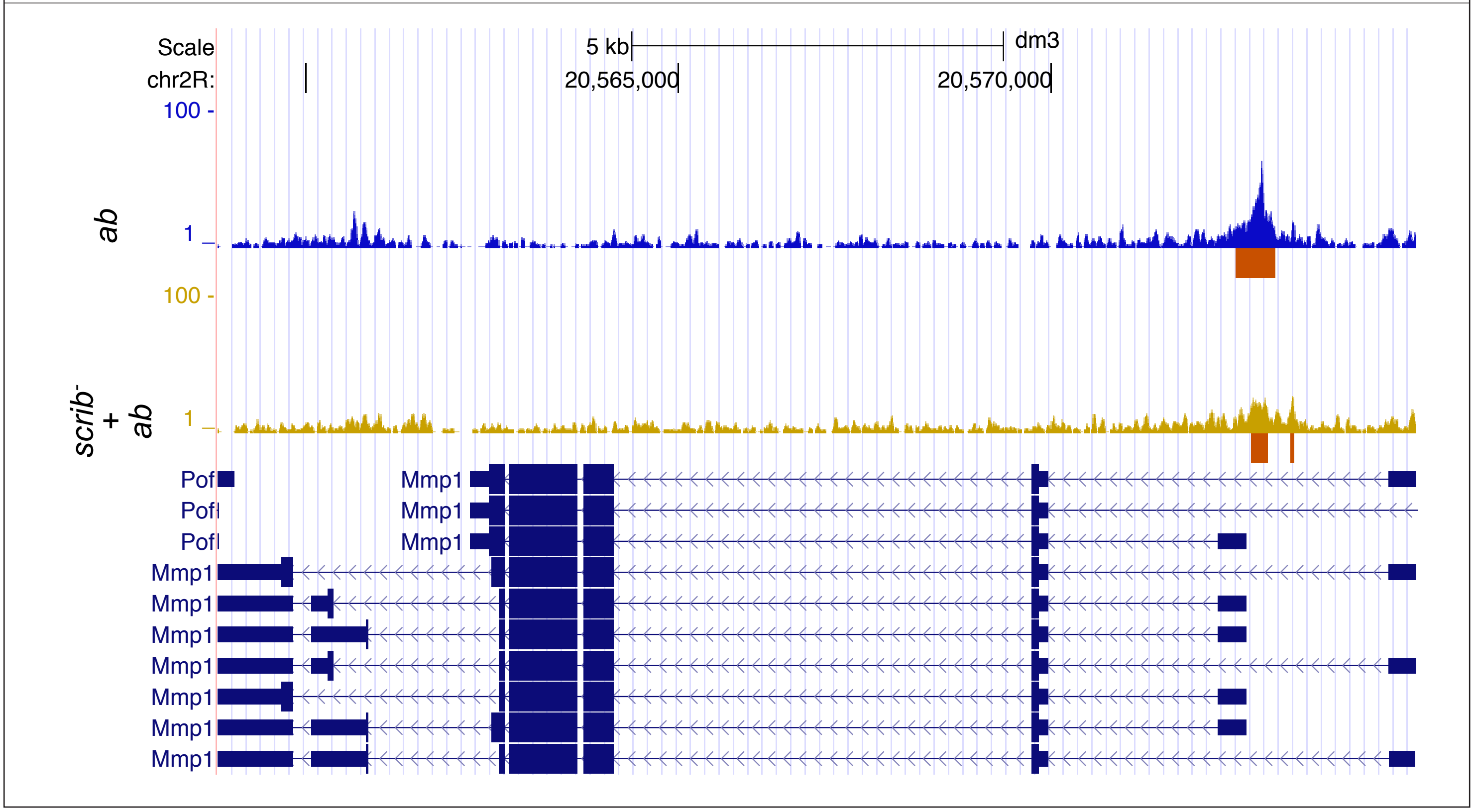

Scale
5 kb
dm3
chr2R:
20,565,000
20,570,000
100 -
ab
1
100 -
scrib⁻ + ab
1
Pof
Pof
Pof
Mmp1
Mmp1
Mmp1
Mmp1
Mmp1
Mmp1
Mmp1
Mmp1
Mmp1
Mmp1
Mmp1
Mmp1
Mmp1

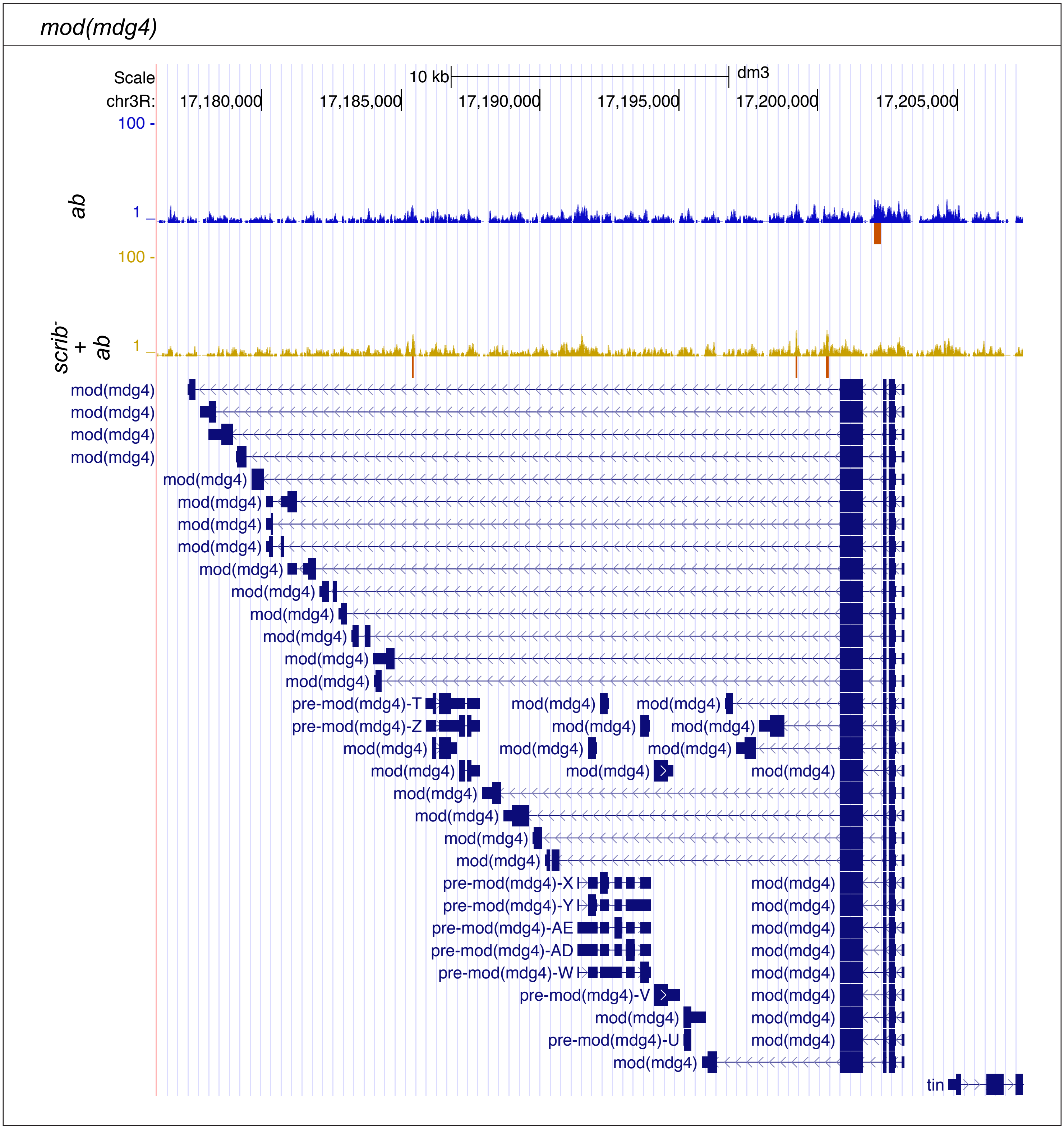
mod(mdg4)
Scale
10 kb
dm3
chr3R:
17,180,000
17,185,000
17,190,000
17,195,000
17,200,000
17,205,000
100 -
1
ab
100 -
1
scrib- + ab
mod(mdg4)
pre-mod(mdg4)-T
pre-mod(mdg4)-Z
pre-mod(mdg4)-X
pre-mod(mdg4)-Y
pre-mod(mdg4)-AE
pre-mod(mdg4)-AD
pre-mod(mdg4)-W
pre-mod(mdg4)-V
pre-mod(mdg4)-U
tin

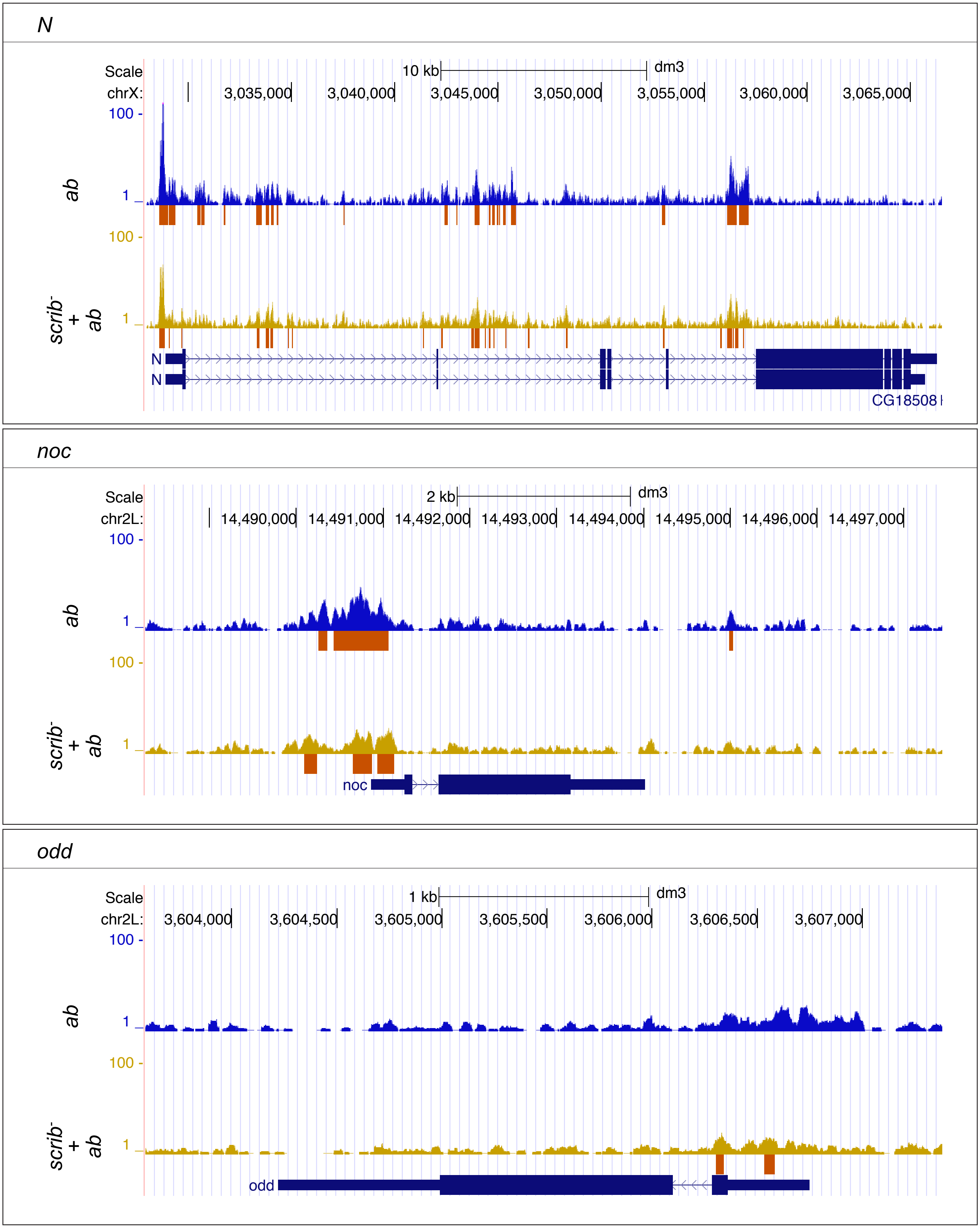

N
Scale
10 kb
dm3
chrX:
3,035,000
3,040,000
3,045,000
3,050,000
3,055,000
3,060,000
3,065,000
100 -
ab
1
100 -
scrib- + ab
1
N
N
CG18508
noc
Scale
2 kb
dm3
chr2L:
14,490,000
14,491,000
14,492,000
14,493,000
14,494,000
14,495,000
14,496,000
14,497,000
100 -
ab
1
100 -
scrib- + ab
1
noc
odd
Scale
1 kb
dm3
chr2L:
3,604,000
3,604,500
3,605,000
3,605,500
3,606,000
3,606,500
3,607,000
100 -
ab
1
100 -
scrib- + ab
1
odd

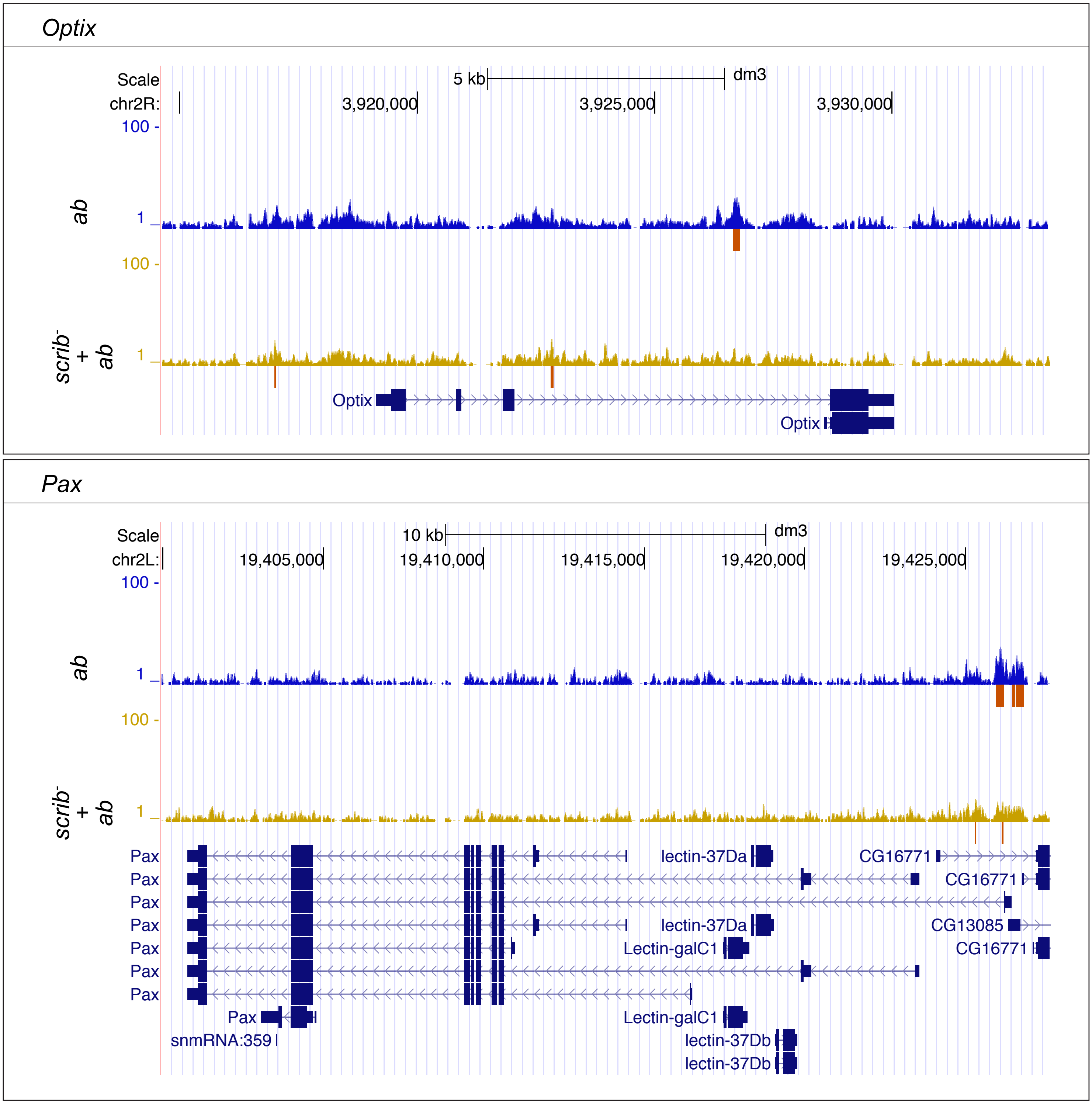
Optix
Scale
chr2R:
5 kb
dm3
3,920,000
3,925,000
3,930,000
100 -
ab
1
100 -
scrib- + ab
1
Optix
Optix
Pax
Scale
chr2L:
10 kb
dm3
19,405,000
19,410,000
19,415,000
19,420,000
19,425,000
100 -
ab
1
100 -
scrib- + ab
1
Pax
Pax
Pax
Pax
Pax
Pax
Pax
Pax
snmRNA:359
lectin-37Da
lectin-37Da
Lectin-galC1
Lectin-galC1
lectin-37Db
lectin-37Db
CG16771
CG16771
CG13085
CG16771

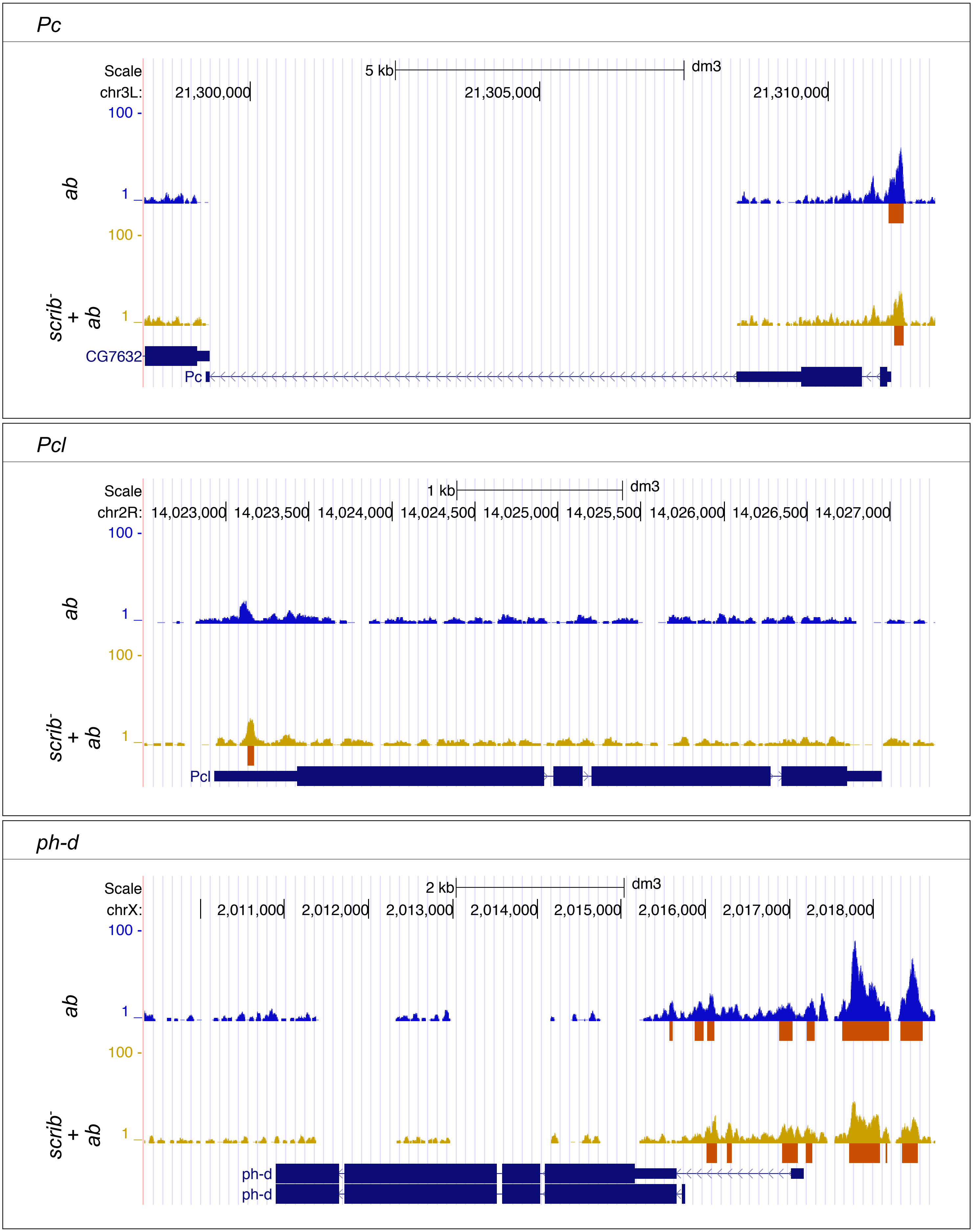
Pc
Scale
5 kb
dm3
chr3L:
21,300,000
21,305,000
21,310,000
100 -
1
ab
scrib-
+
ab
CG7632
Pc
Pcl
Scale
1 kb
dm3
chr2R:
14,023,000
14,023,500
14,024,000
14,024,500
14,025,000
14,025,500
14,026,000
14,026,500
14,027,000
100 -
1
ab
scrib-
+
ab
Pcl
ph-d
Scale
2 kb
dm3
chrX:
2,011,000
2,012,000
2,013,000
2,014,000
2,015,000
2,016,000
2,017,000
2,018,000
100 -
1
ab
scrib-
+
ab
ph-d
ph-d

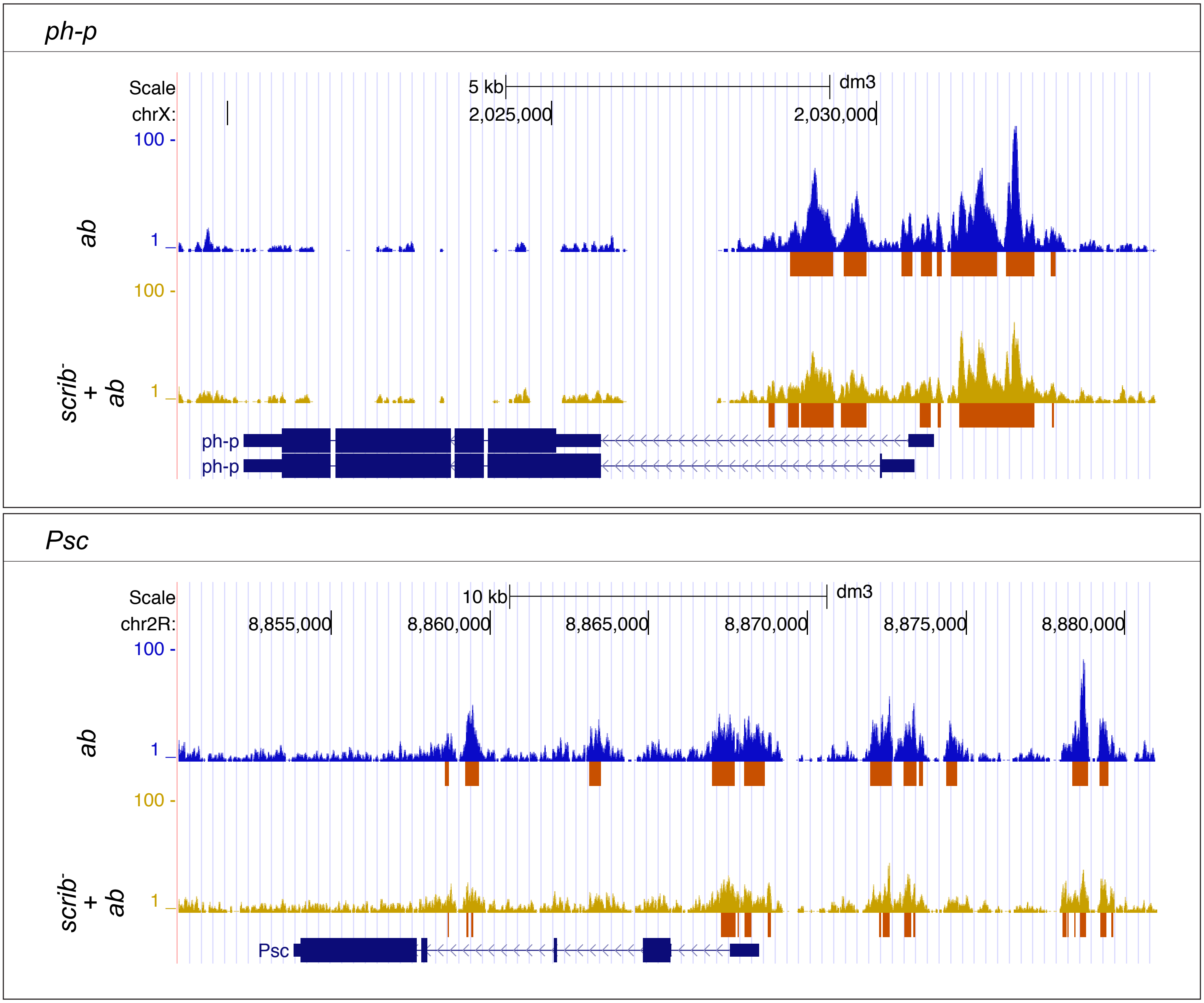

ph-p
Scale
5 kb
dm3
chrX:
2,025,000
2,030,000
100 -
1 _
ab
100 -
1 _
scrib- + ab
ph-p
ph-p
Psc
Scale
10 kb
dm3
chr2R:
8,855,000
8,860,000
8,865,000
8,870,000
8,875,000
8,880,000
100 -
1 _
ab
100 -
1 _
scrib- + ab
Psc

psq

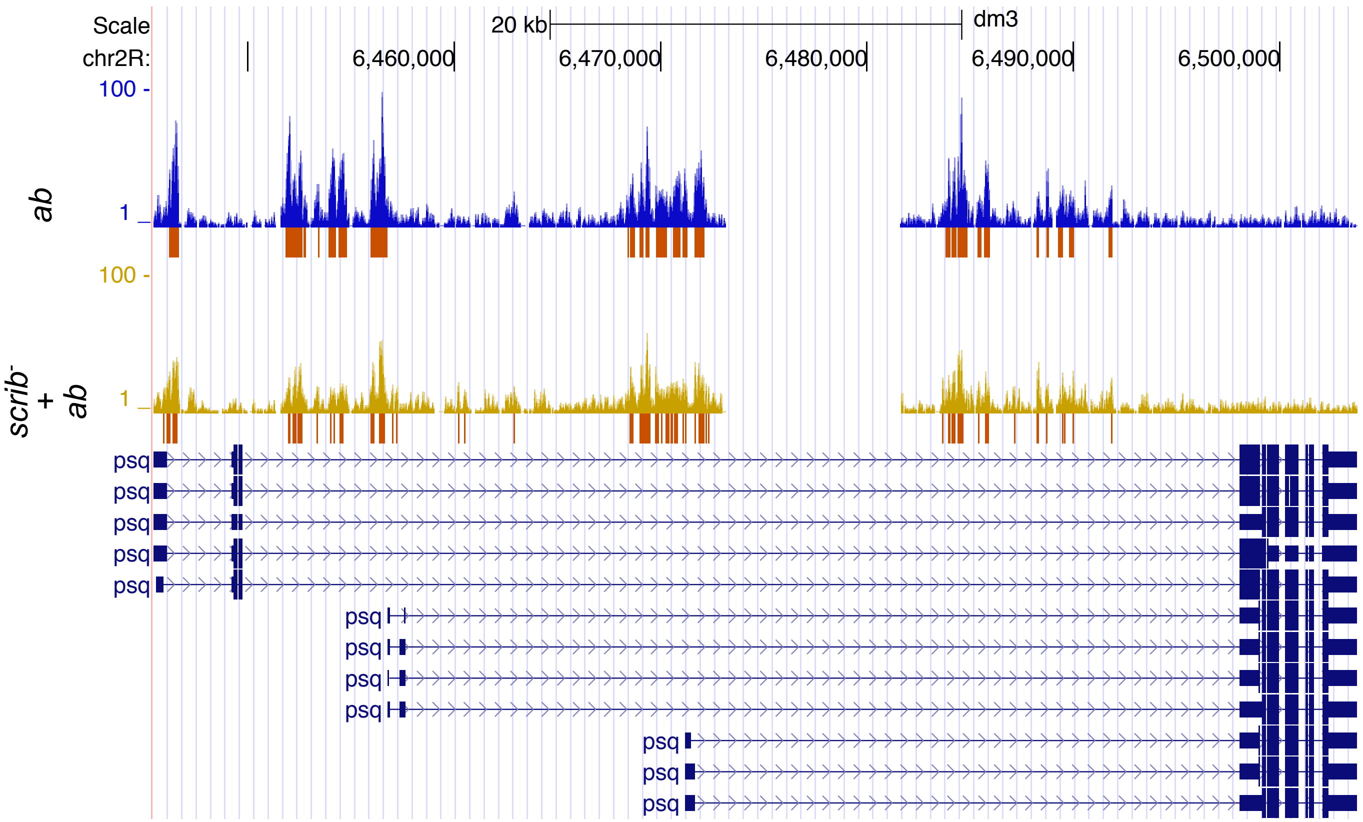
Scale
20 kb
dm3
chr2R:
6,460,000
6,470,000
6,480,000
6,490,000
6,500,000
100 -
1 _
ab
100 -
1 _
scrib- + ab
psq

Pten

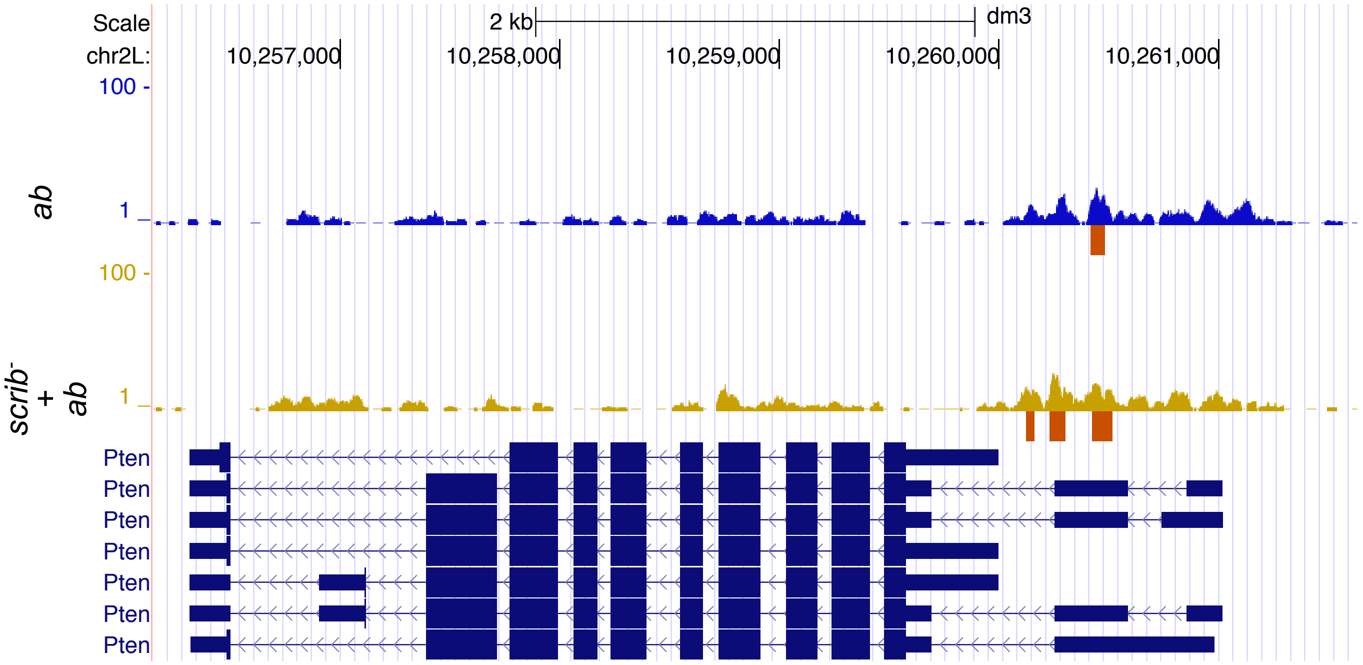
Scale
2 kb
dm3
chr2L:
10,257,000
10,258,000
10,259,000
10,260,000
10,261,000
100 -
1 _
ab
100 -
1 _
scrib- + ab
Pten

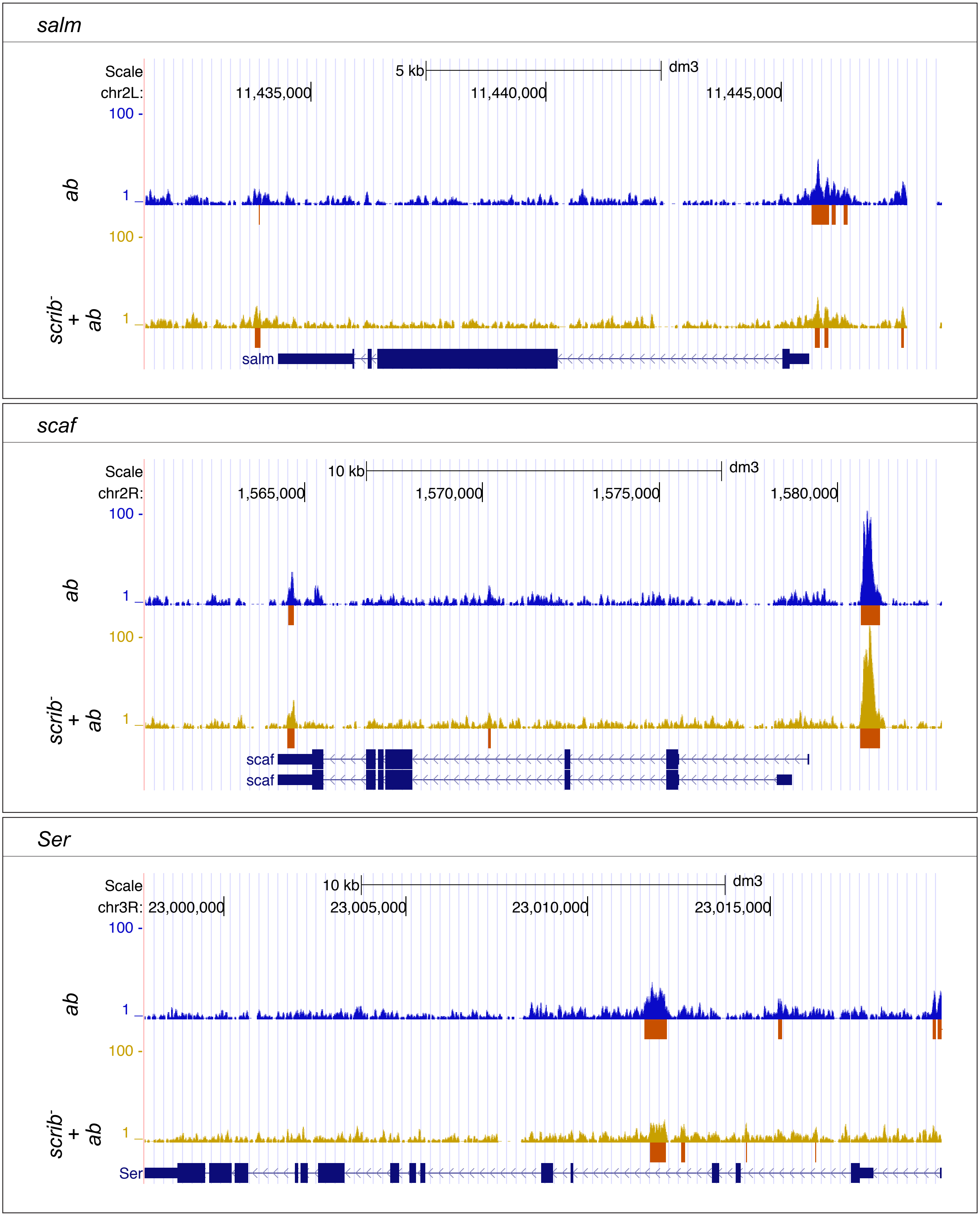

salm
Scale
chr2L:
5 kb
dm3
11,435,000
11,440,000
11,445,000
100 -
ab
1
100 -
scrib⁻ + ab
1
salm
scaf
Scale
chr2R:
10 kb
dm3
1,565,000
1,570,000
1,575,000
1,580,000
100 -
ab
1
100 -
scrib⁻ + ab
1
scaf
scaf
Ser
Scale
chr3R:
10 kb
dm3
23,000,000
23,005,000
23,010,000
23,015,000
100 -
ab
1
100 -
scrib⁻ + ab
1
Ser

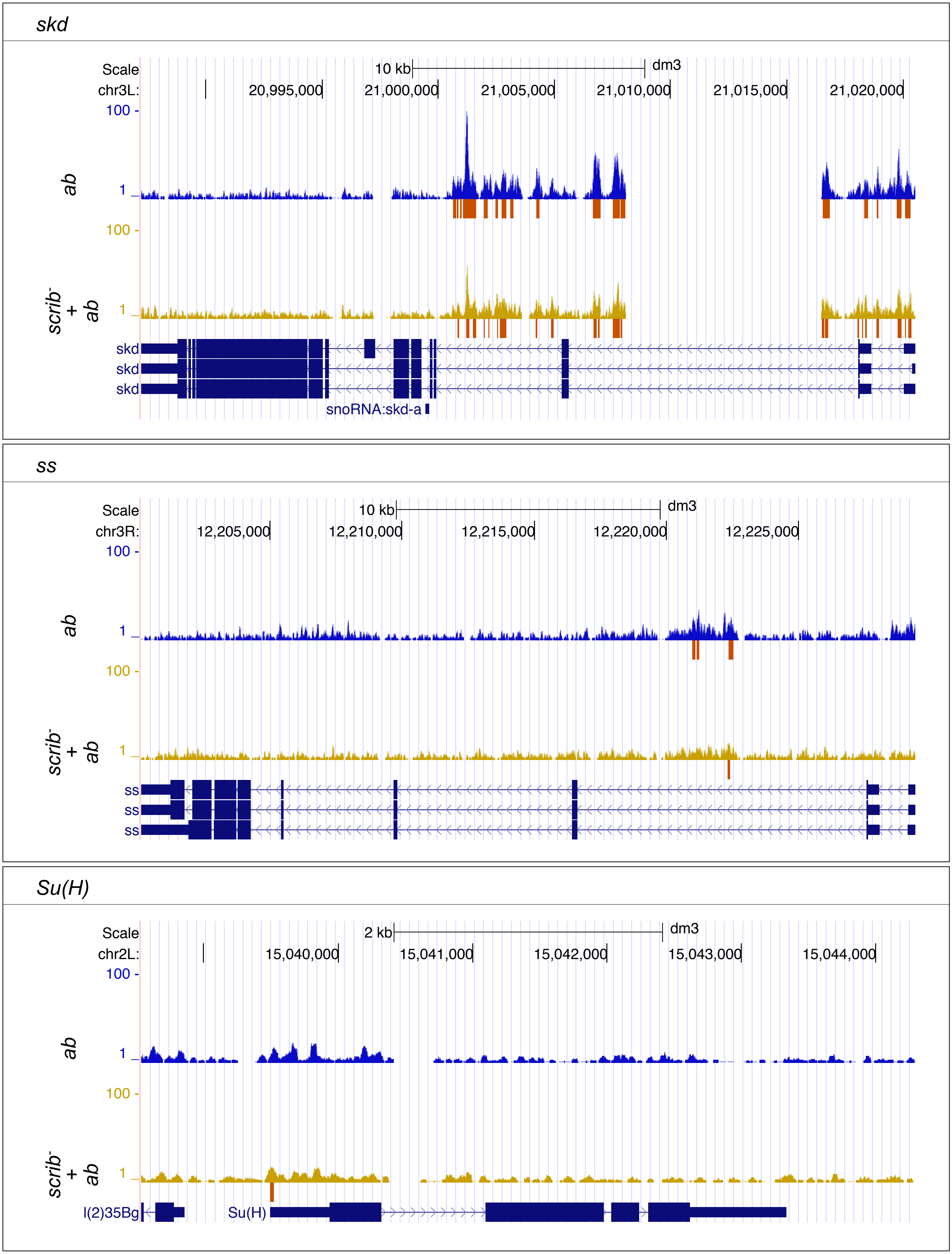
skd
Scale
10 kb
dm3
chr3L:
20,995,000
21,000,000
21,005,000
21,010,000
21,015,000
21,020,000
100 -
1
ab
100 -
1
scrib- + ab
skd
skd
skd
snoRNA:skd-a
ss
Scale
10 kb
dm3
chr3R:
12,205,000
12,210,000
12,215,000
12,220,000
12,225,000
100 -
1
ab
100 -
1
scrib- + ab
ss
ss
ss
Su(H)
Scale
2 kb
dm3
chr2L:
15,040,000
15,041,000
15,042,000
15,043,000
15,044,000
100 -
1
ab
100 -
1
scrib- + ab
l(2)35Bg
Su(H)

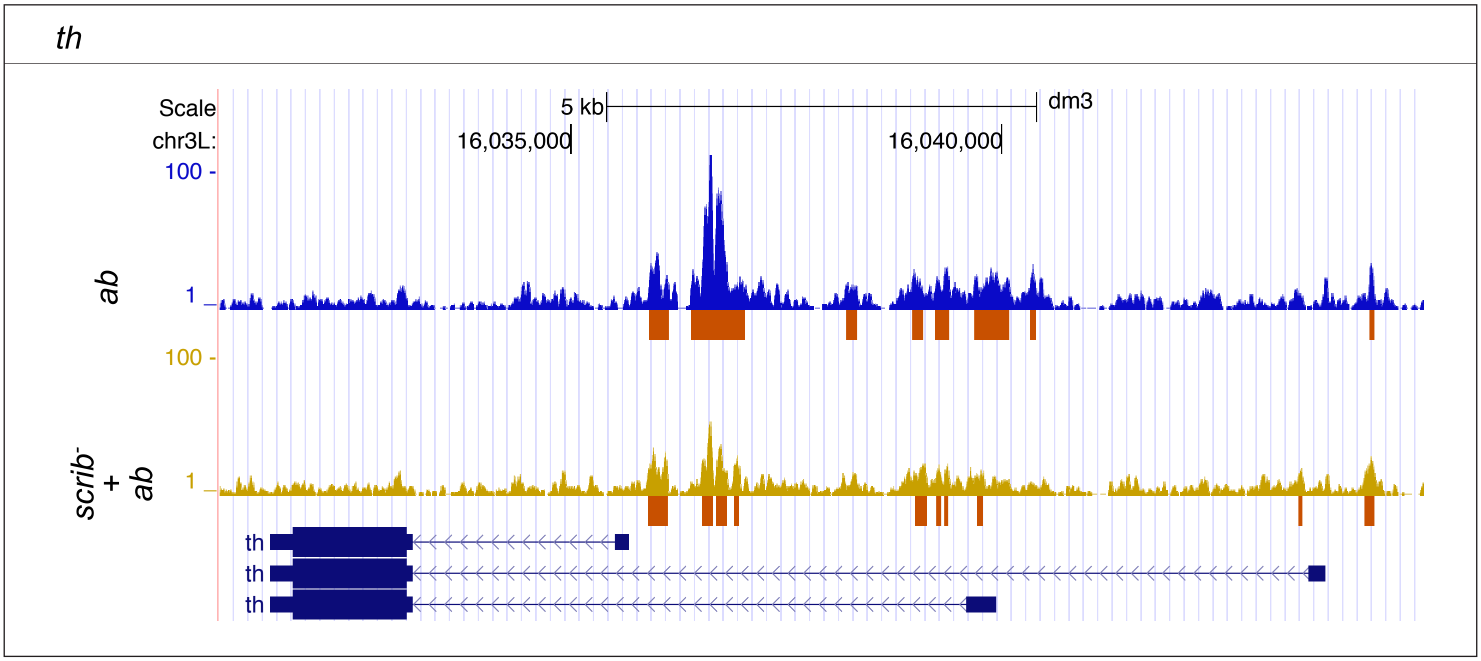
th
Scale
5 kb
dm3
chr3L:
16,035,000
16,040,000
100 -
ab
1
100 -
scrib⁻ + ab
1
th
th
th

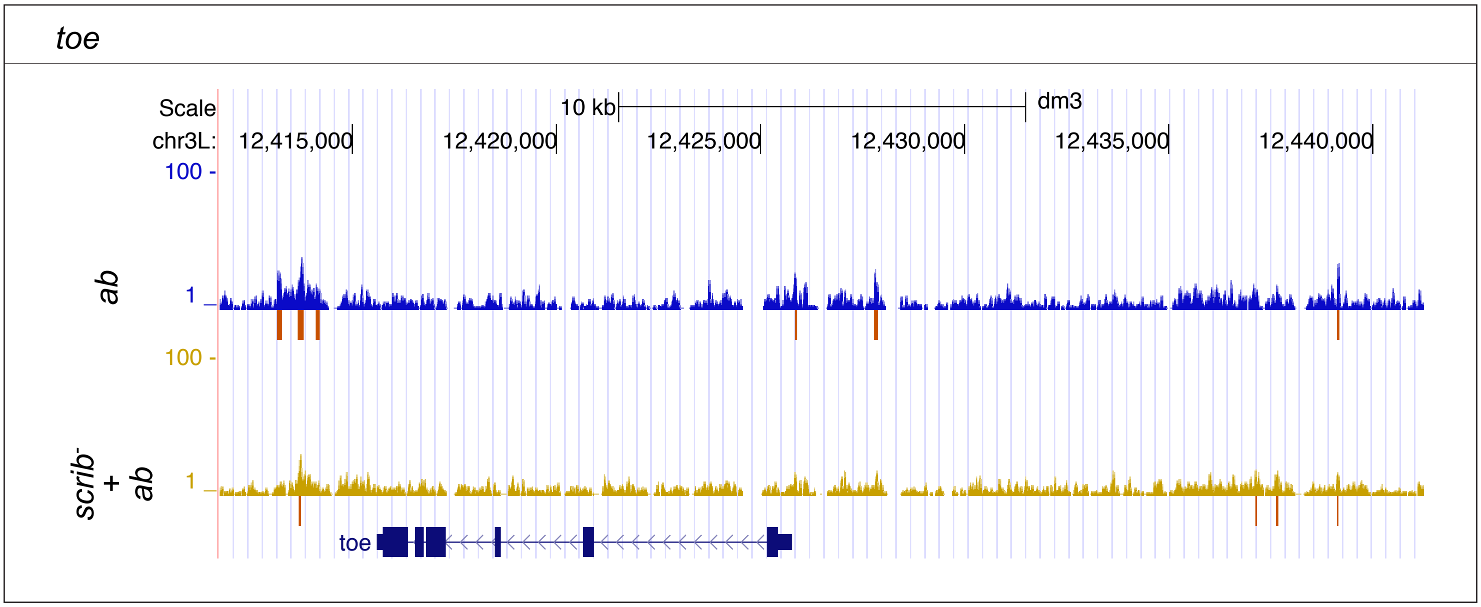
toe
Scale
10 kb
dm3
chr3L:
12,415,000
12,420,000
12,425,000
12,430,000
12,435,000
12,440,000
100 -
ab
1
100 -
scrib⁻ + ab
1
toe

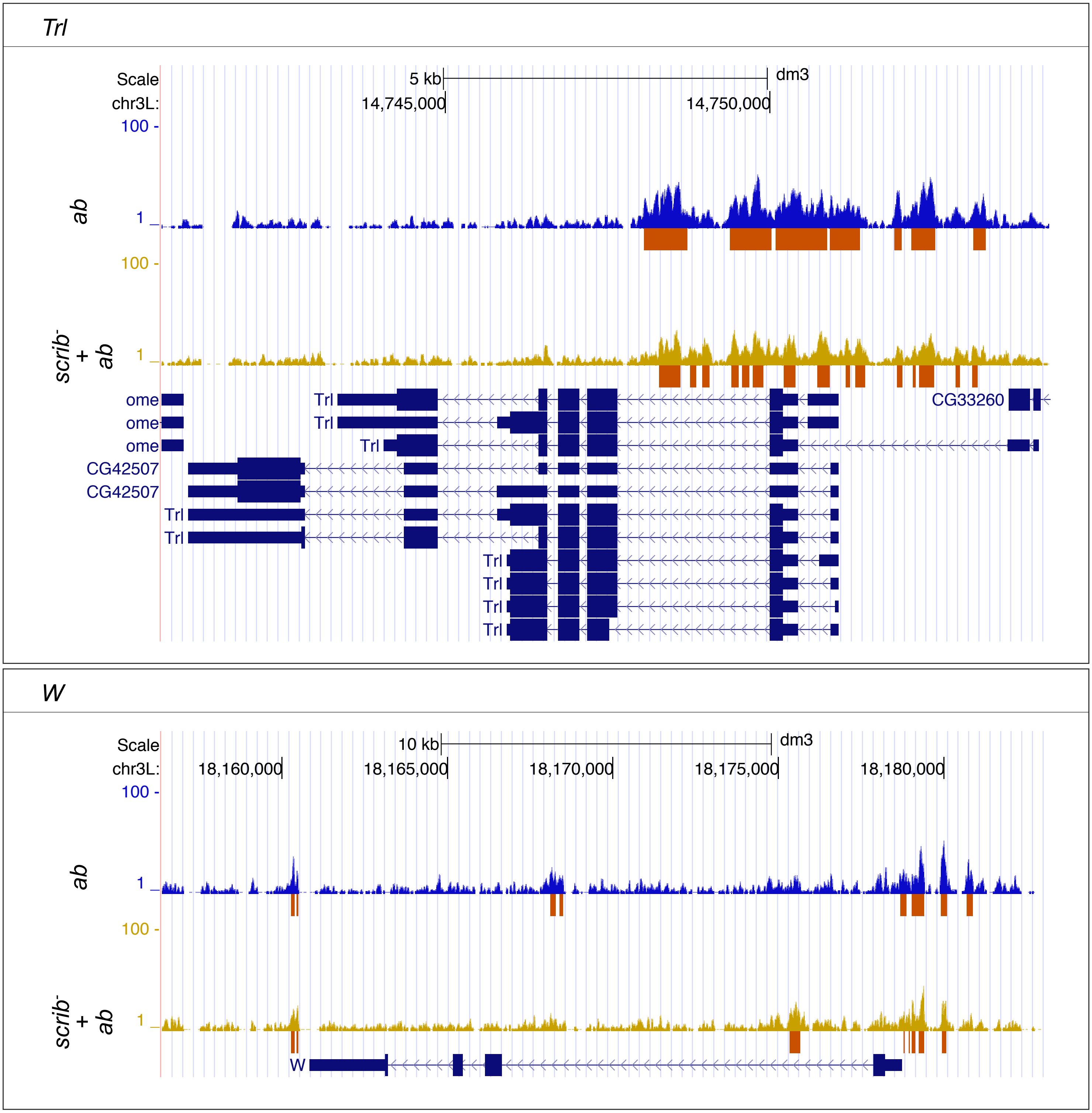
Trl
Scale
5 kb
dm3
chr3L:
14,745,000
14,750,000
100 -
ab
1
100 -
scrib⁻ + ab
1
ome
ome
ome
CG42507
CG42507
Trl
Trl
Trl
Trl
Trl
Trl
Trl
Trl
Trl
CG33260
W
Scale
10 kb
dm3
chr3L:
18,160,000
18,165,000
18,170,000
18,175,000
18,180,000
100 -
ab
1
100 -
scrib⁻ + ab
1
W

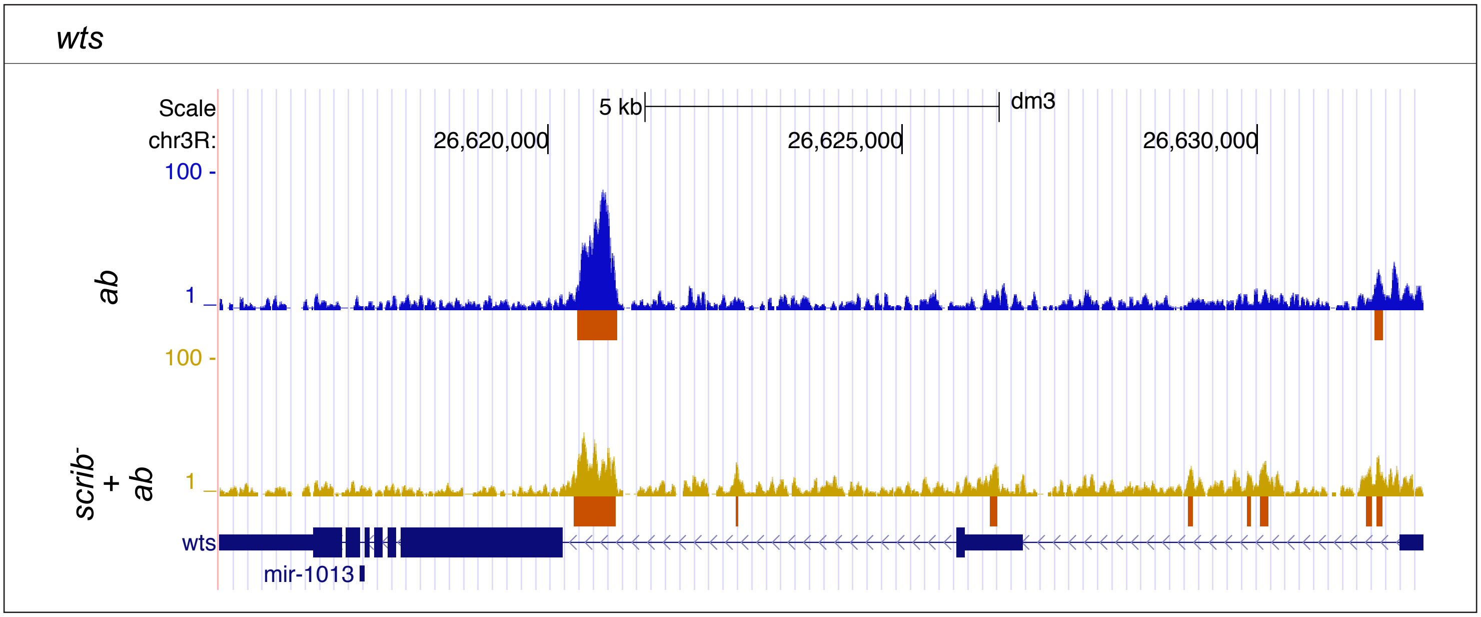

wts
Scale
5 kb
dm3
chr3R:
26,620,000
26,625,000
26,630,000
100 -
1
ab
100 -
1
scrib⁻ + ab
wts
mir-1013
