# Supplementary figures and images for: The BTB-zinc Finger Transcription Factor Abrupt Acts as an Epithelial Oncogene in Drosophila melanogaster through Maintaining a Progenitor-like Cell State
Source: PLoS Genet. 2013 Jul 18;9(7):e1003627. doi: 10.1371/journal.pgen.1003627 (PMC3715428; doi:10.1371/journal.pgen.1003627)

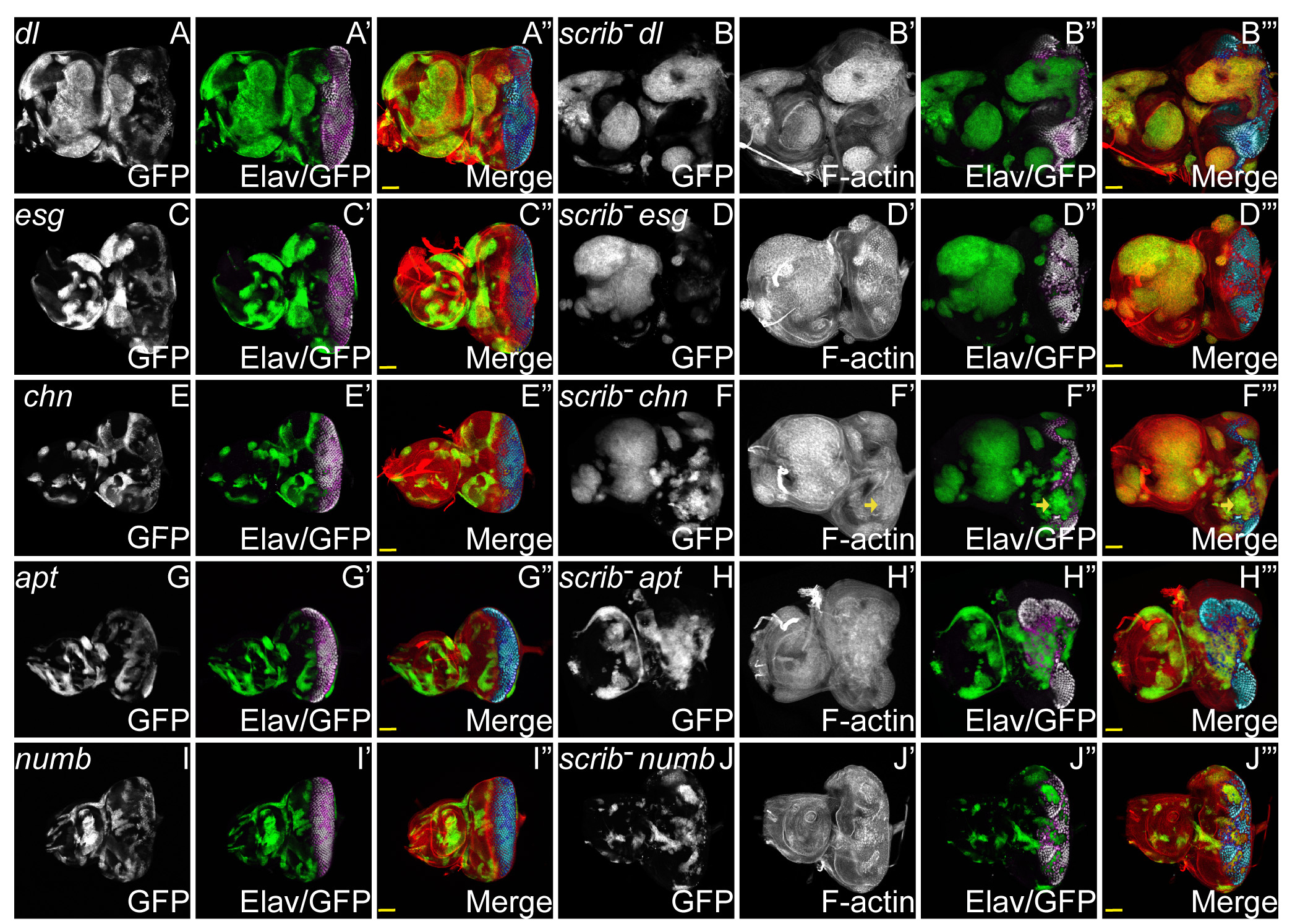

Supplement: Figure S1 — Overexpression phenotypes of confirmed scrib − interactors in eye/antennal disc clones. Mosaic eye/antennal discs (anterior to the left in this and all subsequent figures) generated with ey-FLP and taken from larvae ∼5 days AEL. Clones are positively marked by GFP (white, or green in merges), tissue morphology is shown by F-actin (red in merges), and cell fate by Elav expression (white or pale blue, changing to magenta or dark blue when overlaid with GFP). GFP (panels A–J), GFP/Elav merges (panels A′,B″,C′,D″,E′,F″,G′,H″,I′J″), F-actin (B′,D′,F′,H′,J′) and GFP/Elav/F-actin merges (panels A″,B″′,C″,D″′,E″,F″′,G″,H″′,I″J″′). (A,B) dl-expressing clones and mosaic discs are overgrown, especially within the antennal region (A). The expression of dl in scrib1 clones also promotes overgrowth within the antennal region, and in the eye disc, clonal tissue also overgrows and does not express Elav (B). (C,D) esg-expressing clones are not overgrown (C), however, the expression of esg in scrib1 clones promotes large overgrowths especially within the antennal region (D). (E,F) chn-expressing clones are not overgrown (E), however, the expression of chn in scrib1 clones promotes antennal disc overgrowth, as well as overgrowth of eye disc tissue that does not express Elav (F, arrow). (G,H) apt-expressing clones are not overgrown (G), however, the expression of apt in scrib1 clones promotes mild clonal overgrowth although differentiation is not completely blocked (H). (I,J) Neither numb-expressing clones (I), nor scrib1 clones expressing numb, are overgrown (J). Yellow scale bar = 50 µm. (JPG) [file pgen.1003627.s005.jpg]

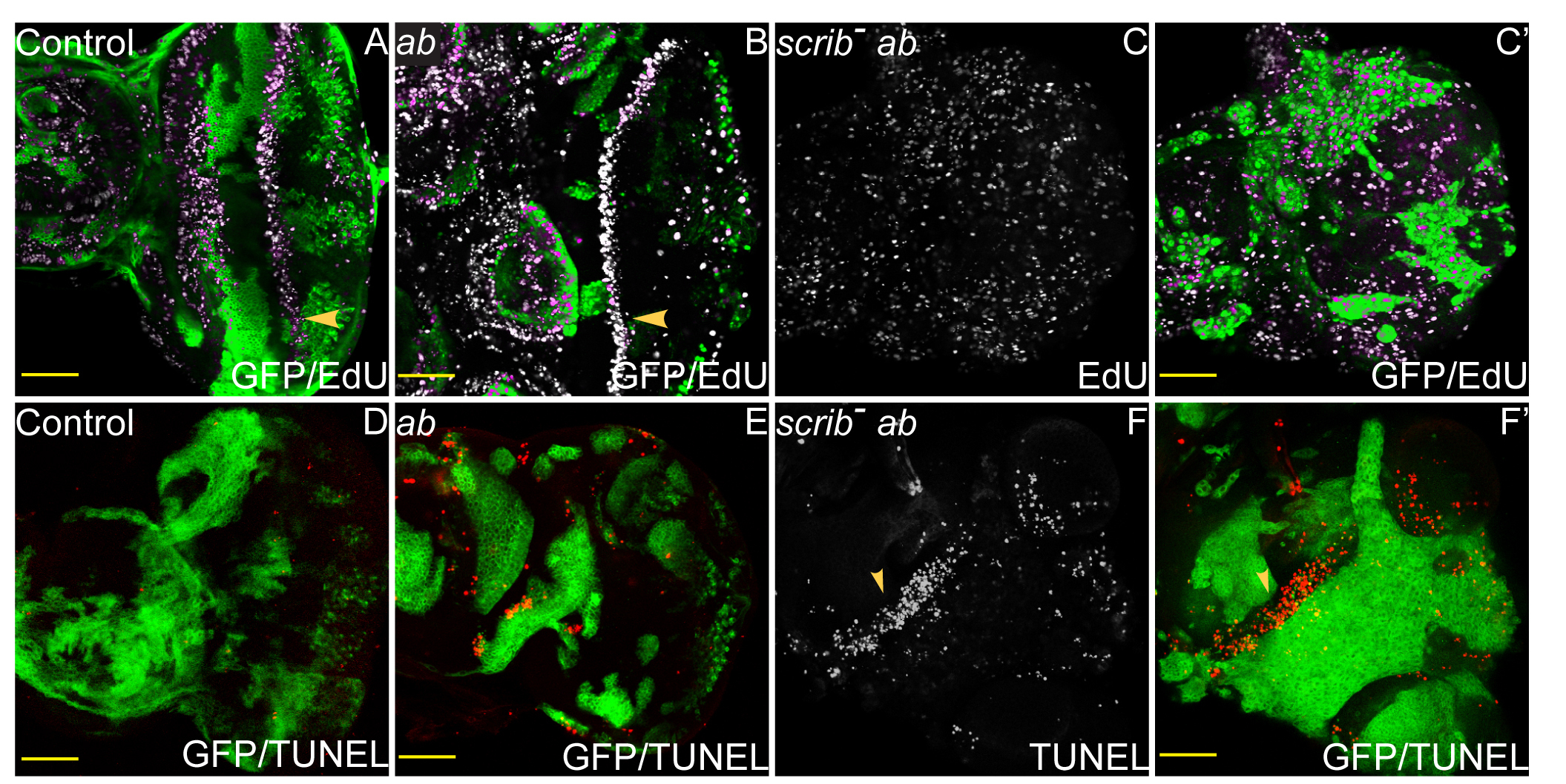

Supplement: Figure S2 — Proliferation and apoptosis in scrib−+ab tumours. ey-FLP induced eye/antennal disc clones marked by GFP (green). EdU (A–C) labeling is white (and magenta when overlayed with GFP in merged images), and TUNEL (D–F) is white (red in merged images, and appears yellow when overlayed with GFP in merged images). Arrowheads in A,B indicate the second mitotic wave. EdU (panel C), GFP/EdU merges (panels A,B,C′), GFP/TUNEL merges (panel D,E,F′), and TUNEL (panel F). (A,D) Wild type mosaic discs show the normal pattern of cell proliferation (A) and cell death (D). (B,E) ab overexpressing eye disc clones do not ectopically proliferate (B), but induce increased cell death in wild type cells along the clonal borders (E). (C,F) scrib1+ab clones ectopically proliferate, and disrupt the normal pattern of cell proliferation in the eye disc (C), and induce increased cell death in surrounding wild type tissue (F, arrowhead). Yellow scale bar = 50 µm. (JPG) [file pgen.1003627.s006.jpg]

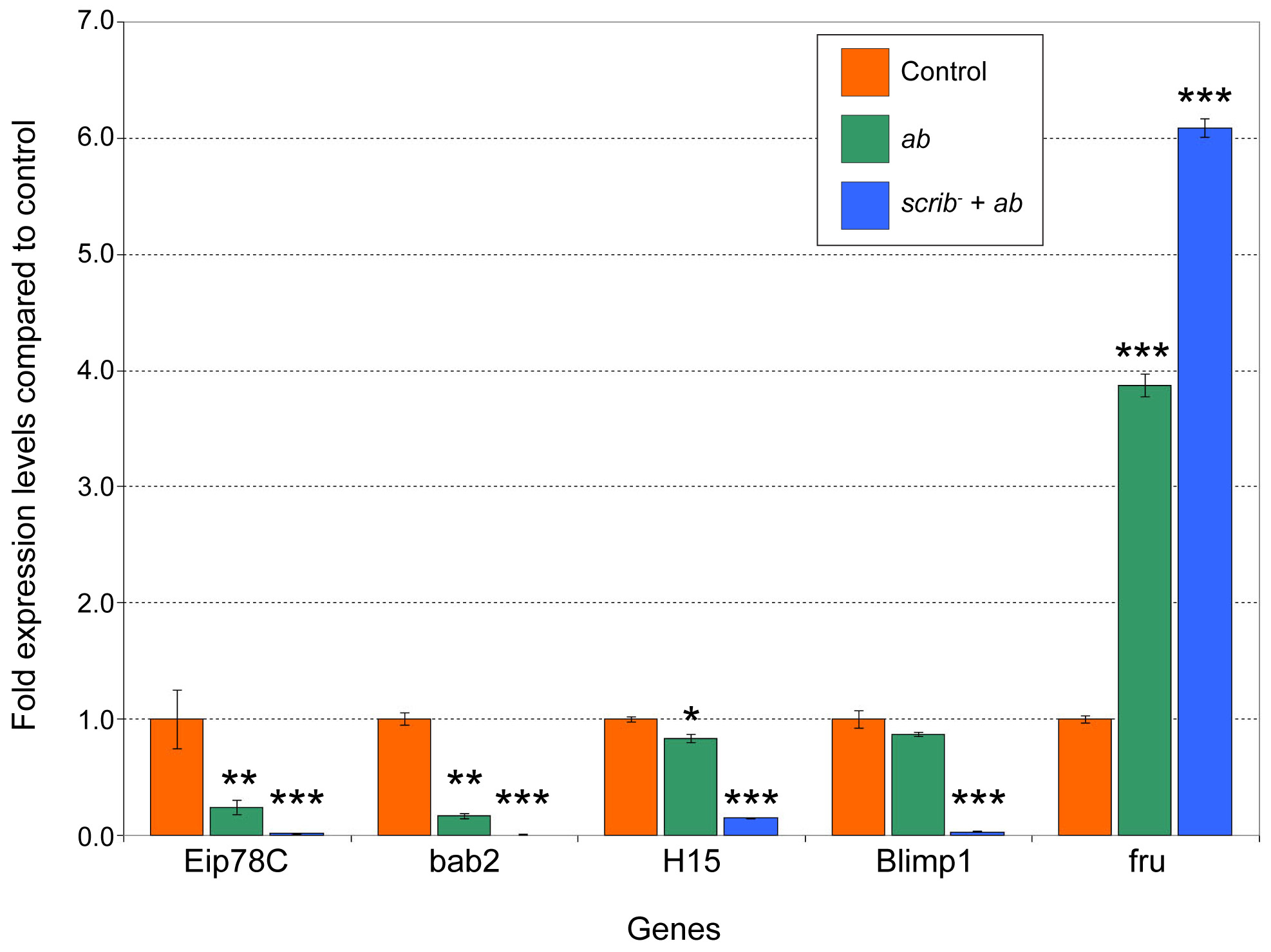

Supplement: Figure S3 — Validation of the expression array by quantitative real-time PCR of selected genes. Expression levels, as determined by quantitative real-time PCR (see Materials and Methods), are shown for 5 genes (Eip78C, bab2, H15, Blimp-1, fru) in ab-expressing discs and in scrib1+ab discs, compared to the expression level in control discs containing wild type FRT82B clones (assigned an expression level of 1). The expression levels of CG6044, a gene that is expressed in the eye/antennal disc but did not significantly change in expression across the arrays, were used for normalisation. All five genes confirm the results from the expression array, with Eip78C, bab2, H15 and Blimp-1 being repressed in ab-expressing discs, and even further repressed in scrib1+ab tumours, whilst fru expression is increased upon ab overexpression, and even further increased in scrib1+ab tumours. ANOVA was performed for each primer pair; * p<0.01, ** p<0.001, *** p<0.0001 compared to the FRT82B control (n = 3). The changes in expression between ab and scrib−+ab samples were also all highly significant (p<0.0001). Error bars indicate 1 s.d. (JPG) [file pgen.1003627.s007.jpg]

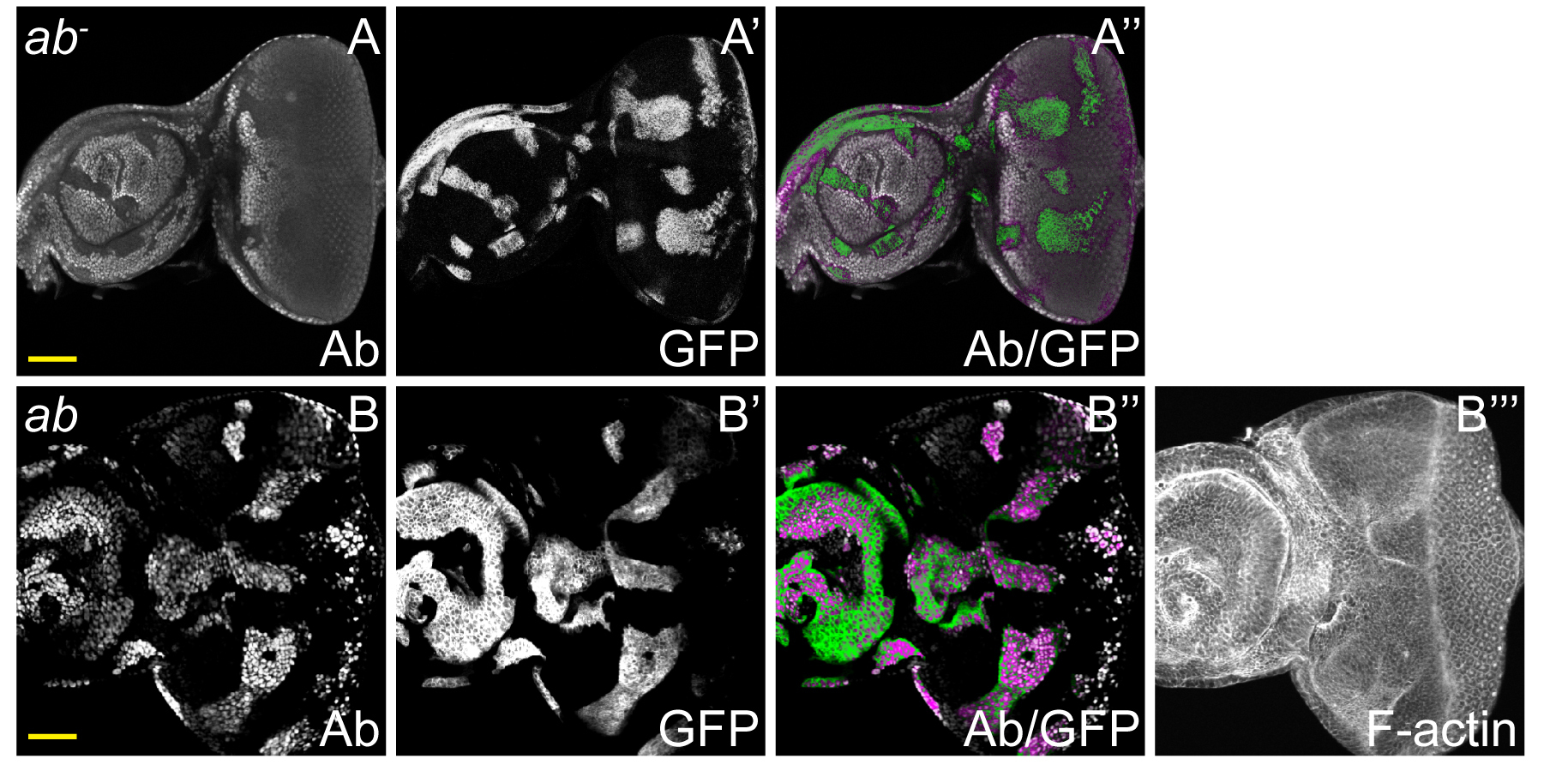

Supplement: Figure S4 — The Ab antibody shows high specificity. Mosaic eye/antennal disc clones generated with ey-FLP. Clones are positively marked by GFP (white or green in merges), and Ab protein levels are shown in white (magenta when overlaid with GFP in the merges). Ab (panels A,B), GFP (panel A′B′) and merges (panels A″,B″). Tissue morphology in B is shown by F-actin (white, panel B″′). (A) Ab is endogenously expressed in the antennal disc, the anterior portion of the eye disc and the peripodial membrane, except in ab1D mutant clones, which show greatly reduced levels of Ab. (B) Clones of tissue overexpressing ab show greatly elevated levels of Ab protein. Yellow scale bar = 50 µm. (JPG) [file pgen.1003627.s008.jpg]

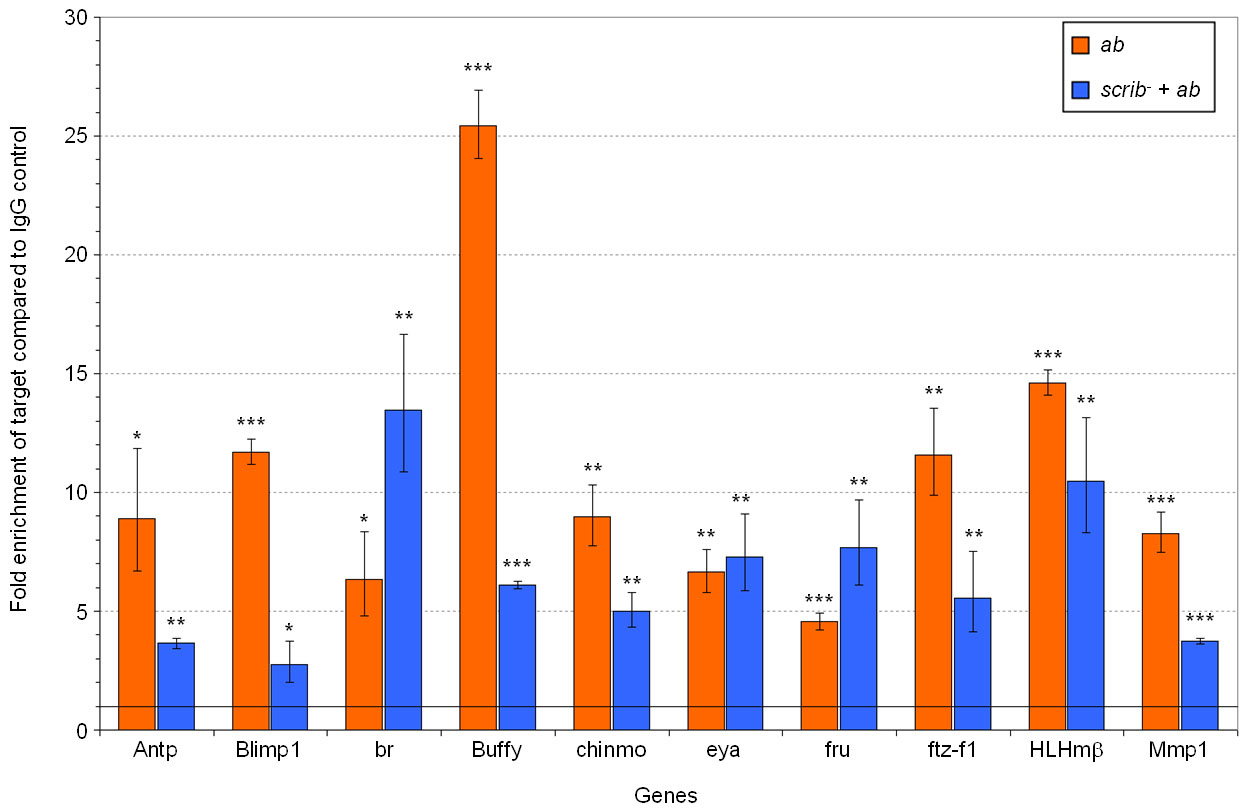

Supplement: Figure S5 — ChIP validation for selected representative genes. Chromatin was immunoprecipitated with the Ab antibody from ab and scrib −+ab mosaic eye/antennal discs, and then used for quantitative real-time PCR. Fold enrichment for all target genes was determined compared to rabbit IgG control immunoprecipitations, which were assigned an expression level of 1 (represented by the line on the graph). Representative genes were included from most functional categories; ecdysone response (Blimp-1, ftz-F1), BTB-ZF (br, chinmo, fru), cell fate (Antp, eya), Notch signalling (HLHmβ), cell death/survival (Buffy) and JNK signaling (Mmp1). Enrichment of all genes was observed in the Ab antibody immunoprecipitation compared to the IgG control from ab and scrib−+ab mosaic eye/antennal imaginal discs. T-test comparing each Ab immunoprecipitation to the IgG control; * p<0.01, ** p<0.001, *** p<0.0001 (n = 3 for both Ab immunoprecipitation and the IgG control). Error bars indicate 1 s.d. (JPG) [file pgen.1003627.s009.jpg]

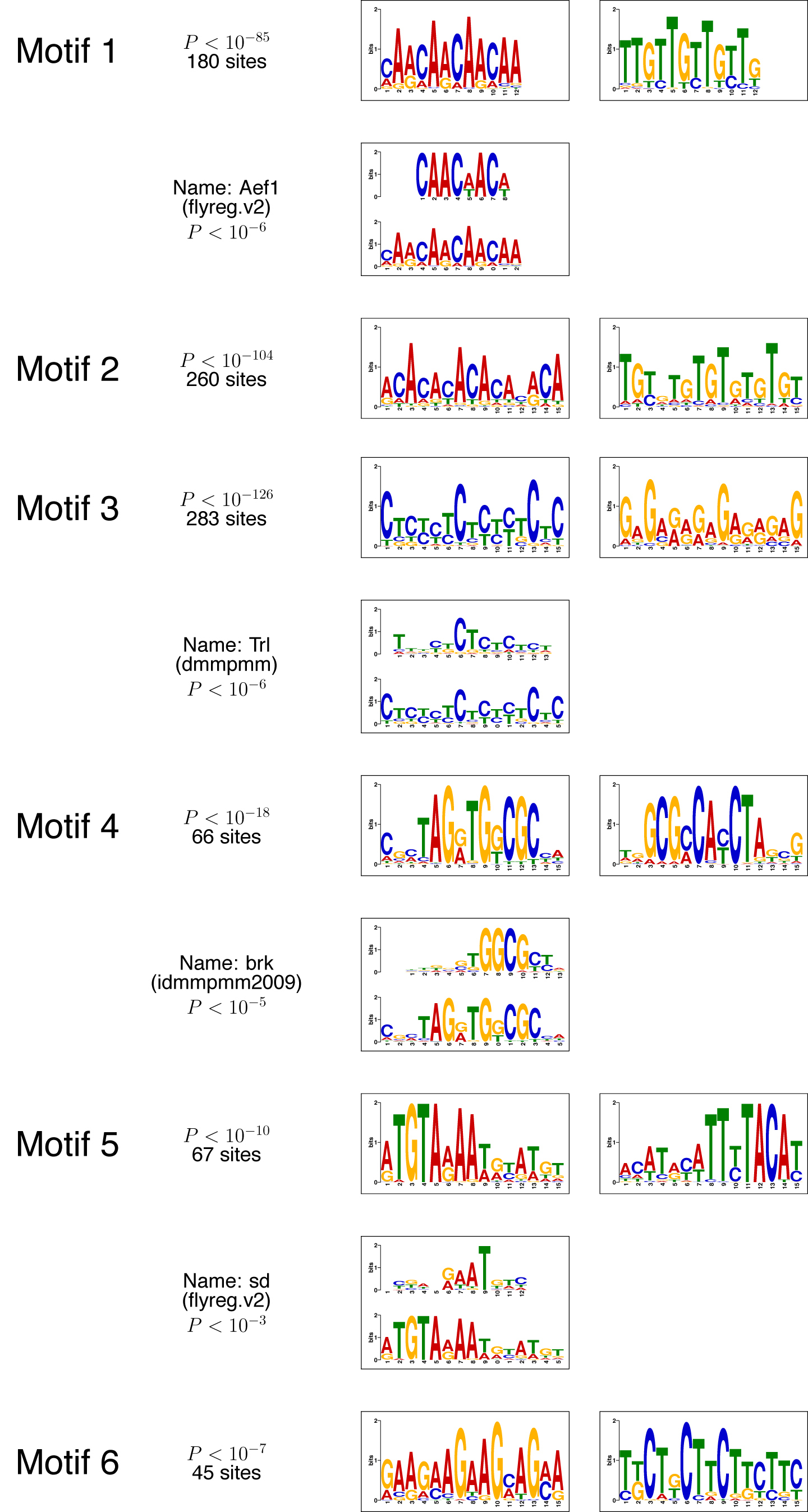

Supplement: Figure S6 — Enriched sequence motifs amongst the top peaks from the ab alone overexpression sample. The strongest peaks were selected with a height of 40 or more reads from the ChIP-Seq profile, resulting in 1629 regions with an average length of 84.8 bp. Enriched motifs were identified using MEME, and potential transcription factors capable of recognizing the enriched motifs were identified using the TOMTOM program (MEME suite). (JPG) [file pgen.1003627.s010.jpg]

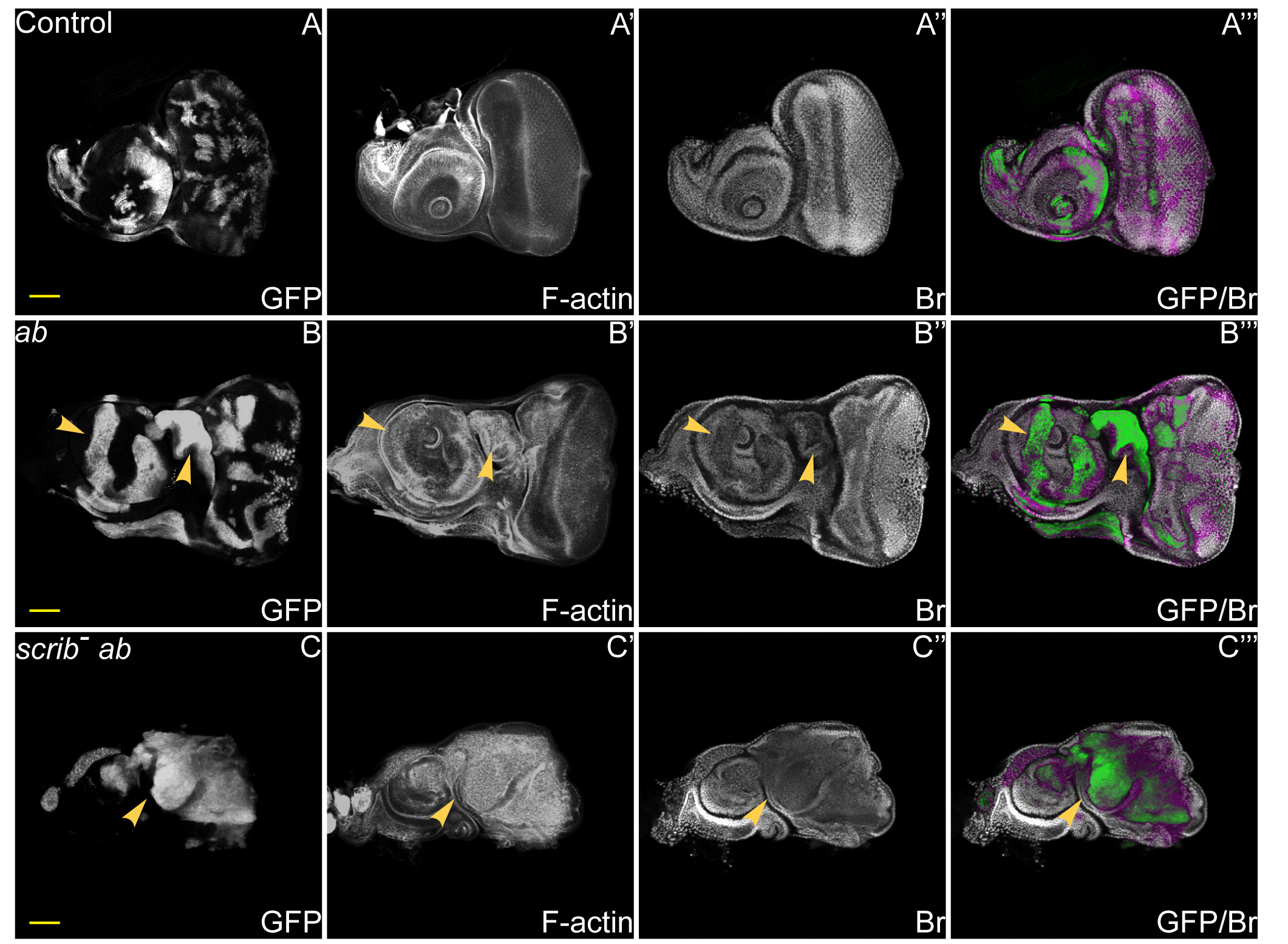

Supplement: Figure S7 — Br is repressed in ab-expressing cells. Mosaic eye/antennal disc clones generated with ey-FLP. Clones are positively marked by GFP (white, or green in merges), and tissue morphology is shown by F-actin (white). Br levels are shown in white (magenta when overlaid with GFP in the merges). GFP (panels A–C), F-actin (panels A′–C′), Br (panels A″–C″), and merges (panels A″′–C″′). (A) Br is expressed throughout the eye/antennal disc and peripodial membrane. (B,C) Br is repressed in ab-expressing clones (B, arrowheads) and in scrib1+ab clones (C, arrowheads). Yellow scale bar = 50 µm. (JPG) [file pgen.1003627.s011.jpg]

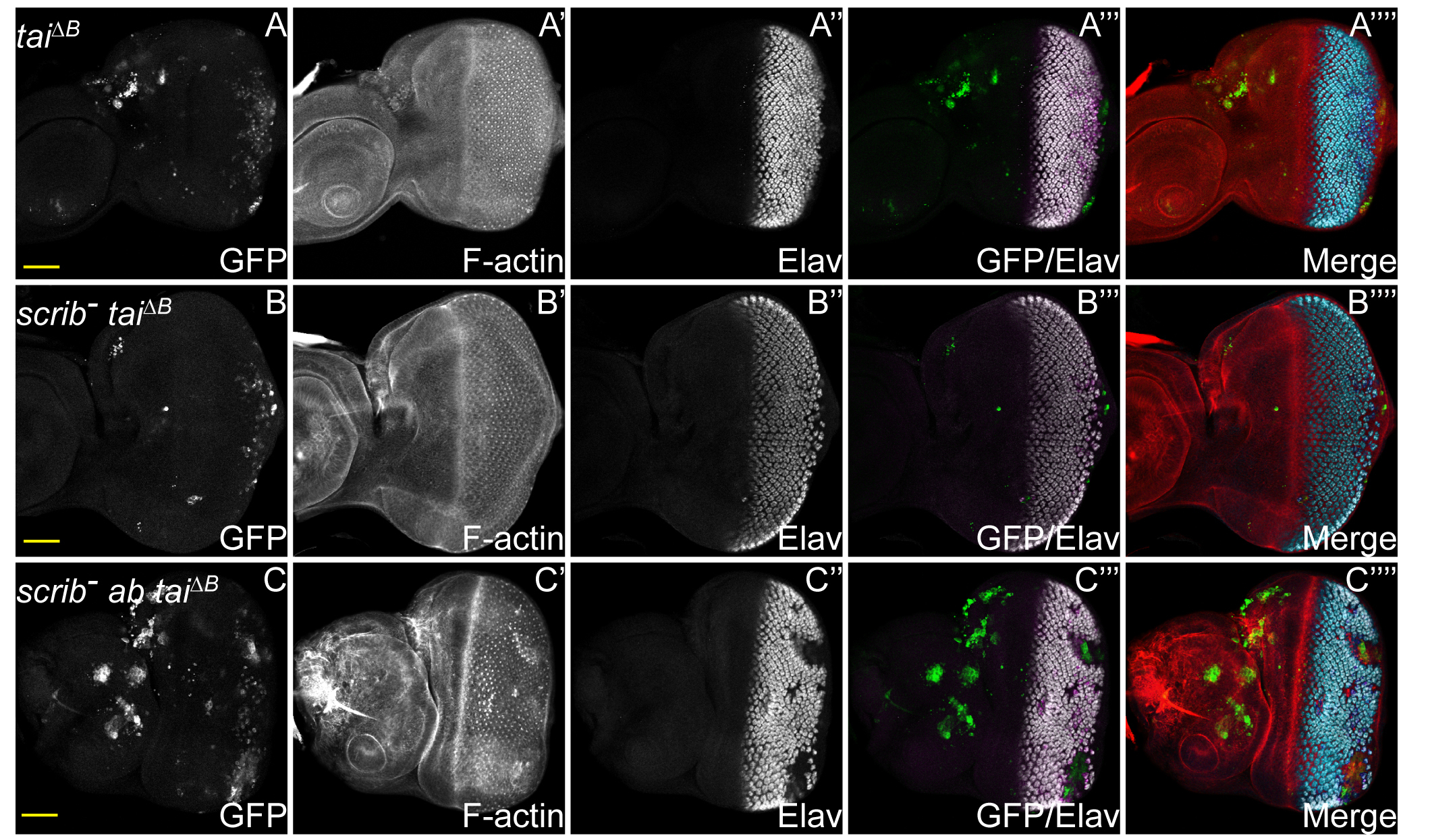

Supplement: Figure S8 — Overexpression of taiΔB in scrib−+ab tumours prevents tumour overgrowth. Mosaic eye/antennal disc clones generated with ey-FLP. Clones are positively marked by GFP (white, or green in merges), tissue morphology is shown by F-actin (red in merges), and cell fate by Elav expression (white or pale blue, changing to magenta or dark blue when overlaid with GFP). GFP (panels A–C), F-actin (panels A′–C′), Elav (panels A″–C″), GFP/Elav merges (panels A″′–C″′) and GFP/Elav/F-actin merges (panels A″″–C″″). (A) Expression of taiΔB in clones results in small clones. (B,C) Expression of taiΔB in scrib1 clones (B) or scrib1+ab clones (C) reduces clonal overgrowth and results in the eclosion of adult flies (data not shown). (JPG) [file pgen.1003627.s012.jpg]

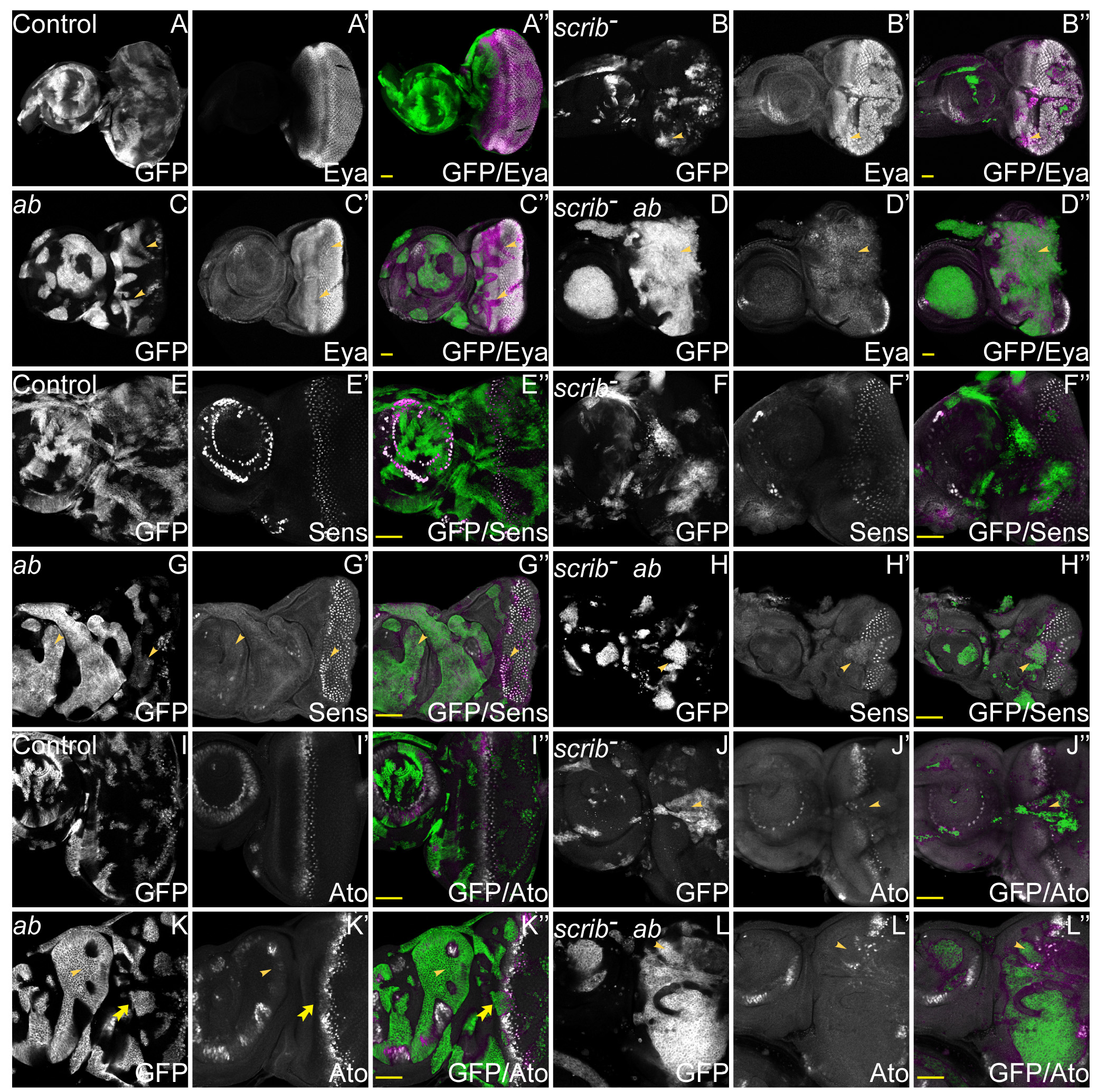

Supplement: Figure S9 — Eya, Sens and Ato expression are reduced in scrib −+ab tumours. ey-FLP induced eye/antennal disc clones at ∼5 days AEL. Clones are marked by GFP (white, or green in merges), and cell fate is shown by the expression of Eya, Sens and Ato (all white, and magenta when overlaid with GFP in the merges) in control clones (A,E,I), scrib1 clones (B,F,J), ab-expressing clones (C,G,K), and scrib1+ab clones (D,H,L). GFP (panels A–L), Eya (panels A′–D′), Sens (panels E′–H′), Ato (panels I′–L′), and merges (panels A″–L″). (A–D) Eya expression is not altered in scrib1 clones (B, arrowhead), or ab overexpressing clones (C, arrowhead). scrib1+ab clones have greatly reduced levels of Eya (D, arrowhead). (E–H) Sens expression is disrupted and reduced in scrib1 clones, and repressed in ab overexpressing clones in the antennal but not in the eye disc (G, arrowheads). scrib1+ab clones do not express Sens (H, arrowhead). (I–L) Ato expression is disrupted and slightly reduced in scrib1 clones (J, arrowhead), and repressed in ab overexpressing clones within the antennal disc (K, arrowhead), and reduced in eye disc clones (K, arrow). scrib1+ab clones do not express Ato (L, arrowhead). Yellow scale bar = 50 µm. (JPG) [file pgen.1003627.s013.jpg]

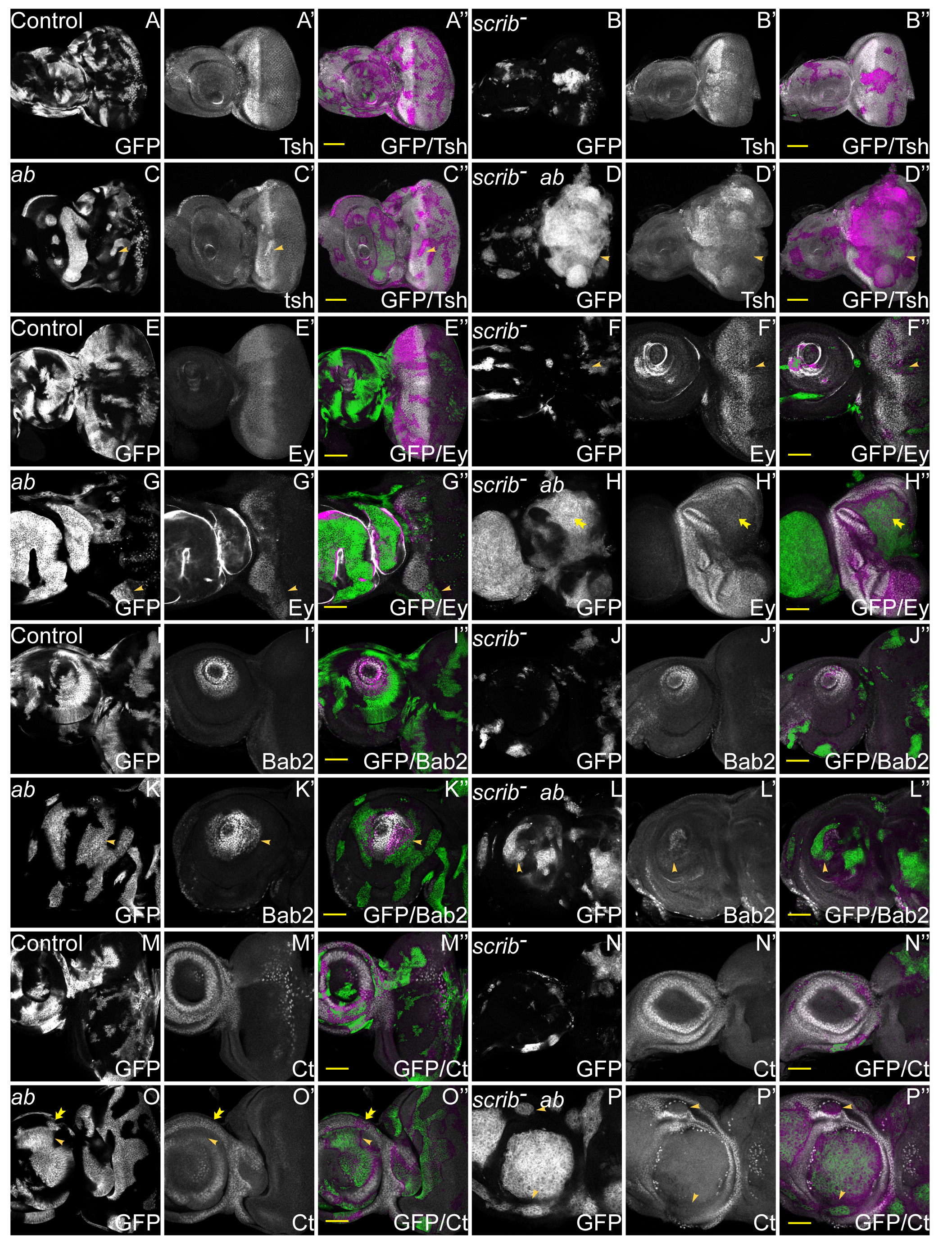

Supplement: Figure S10 — Tsh and Ey expression is not substantially altered in scrib −+ab tumours, however, Bab2 and Ct expression is reduced. ey-FLP induced eye/antennal disc clones at ∼5 days AEL. Clones are marked by GFP (white, or green in merges), and cell fate is shown by the expression of Tsh, Ey, Bab2 and Ato (all white, and magenta when overlaid with GFP in the merges) in control clones (A,E,I,M), scrib1 clones (B,F,J,N), ab-expressing clones (C,G,K,O), and scrib1+ab clones (D,H,L,P). GFP (panels A–P), Tsh (panels A′–D′), Ey (panels E′–H′), Bab2 (panels I′–L′), Ct (panels M′–P′), and merges (panels A″–P″). (A–D) Tsh expression exhibits only slight perturbations in scrib1 clones, sometimes extending more posteriorly within large mutant clones spanning the normal expression domain (B), and is slightly increased in ab overexpressing clones (C, arrowhead). scrib1+ab clones express Tsh in the anterior portion of the eye disc, although it is repressed more posteriorly, as in control clones (D, arrowhead). (E–H) Ey expression is slightly reduced in scrib1 clones (F, arrowhead), and ab overexpressing clones (G, arrowhead). scrib1+ab clones express Ey in the anterior portion of the eye disc, although its expression is repressed, as it is in control clones, more posteriorly (H, arrow). (I–L) Bab2 expression is not altered in scrib1 clones (J), and although expanded, or ectopic, domains of Bab2 expression are sometimes associated with ab overexpressing clones in the antenna, the levels of Bab2 within the clones are slightly reduced compared to adjacent wild type tissue (K; arrowhead showing slightly enlarged Bab2 domain of expression, although levels of Bab2 in the ab-expressing clone are lower than the more highly expressing wild type tissue adjacent to the clone). scrib1+ab clones do not express Bab2 (L, arrowhead). (M–P) Ct expression is not altered in scrib1 clones (N), and is repressed in more distally located ab overexpressing clones (O, arrowhead), although expression is unaffected in [file pgen.1003627.s014.jpg]

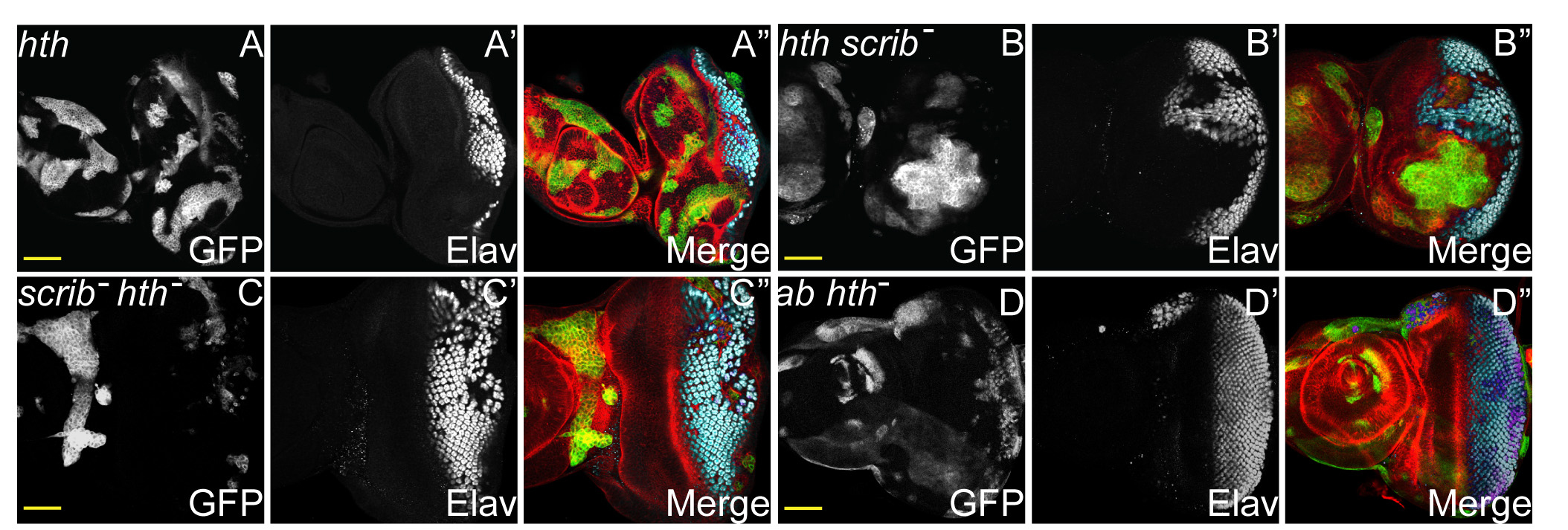

Supplement: Figure S11 — Hth is not sufficient or required for Ab-mediated tumour overgrowth. ey-FLP induced eye/antennal disc clones at 5 days AEL. Clones are marked by GFP (white, or green in merges), and Elav is shown in white (blue in merges, and dark blue when overlaid with GFP). GFP (panels A–D), Elav (panels A′–D′), and merges (panels A″–D″). (A) hth overexpressing clones do not express Elav. (B) Overexpressing hth in scrib1 clones blocks Elav expression and promotes clonal overgrowth, however, the larvae pupate and do not undergo an extended larval stage of development. (C) scrib1 hthP2 mutant clones are similar to hthP2 mutant clones alone (see Figure 5B ), although photoreceptor differentiation is disrupted in posteriorly localised clones. (D) hthP2 mutant clones overexpressing ab are similar to hthP2 mutant clones (see Figure 5B ). (JPG) [file pgen.1003627.s015.jpg]

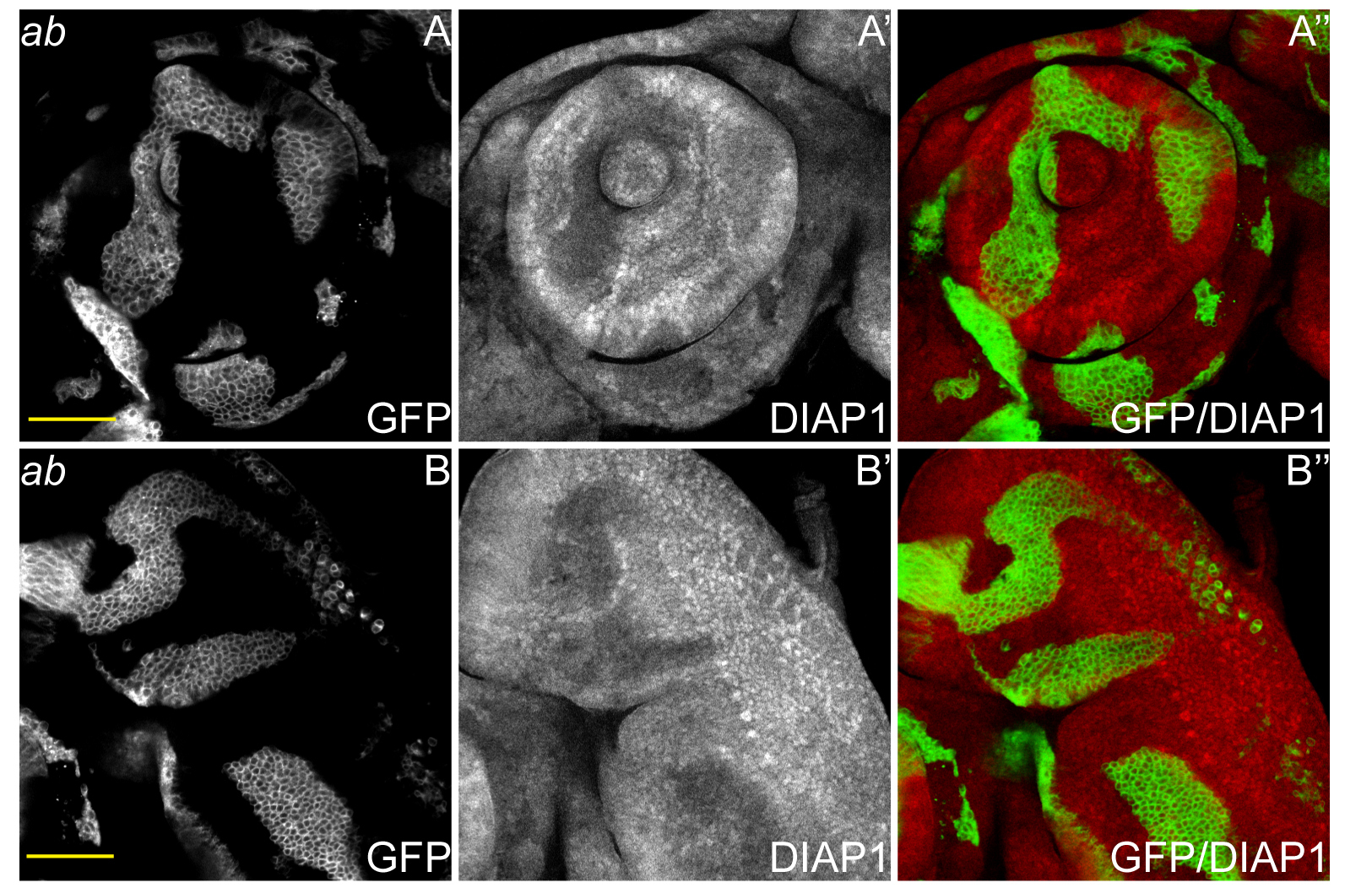

Supplement: Figure S12 — ab overexpression downregulates DIAP1 levels. Mosaic antennal disc clones (A) and eye disc clones (B) overexpressing ab, and positively marked by GFP (white, or green in the merges). Diap1 is shown in white (or red in merges). GFP (panels A,B), Diap1 (panels A′B′), and merges (panels A″,B″). (A,B) ab-overexpressing clones downregulate DIAP1 (red in merges), in both the antennal disc (A) and eye disc (B). Yellow scale bar = 50 µm. (JPG) [file pgen.1003627.s016.jpg]

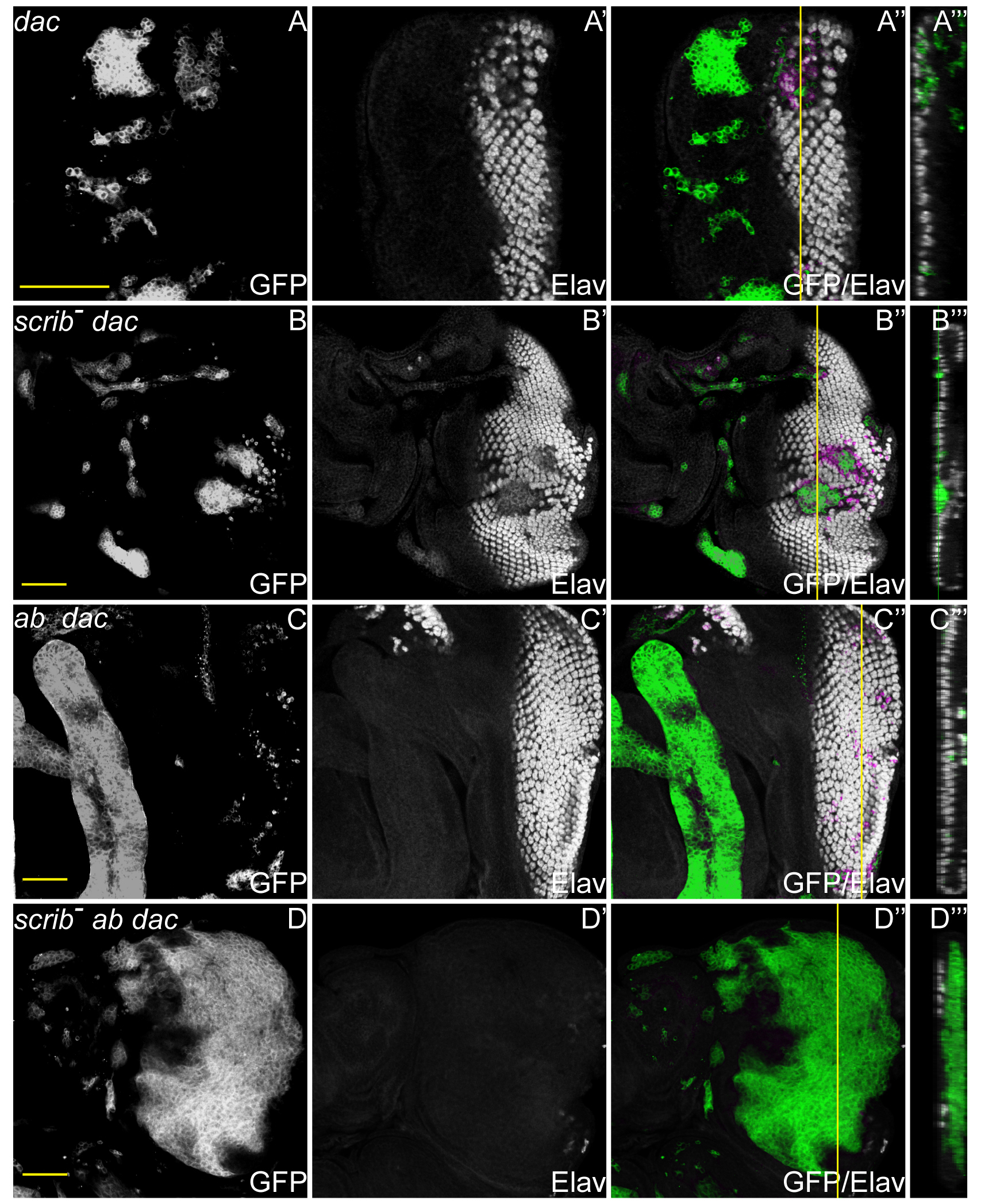

Supplement: Figure S13 — Overexpression of dac does not restrain scrib −+ab tumour overgrowth. ey-FLP clones are positively marked by GFP (white, or green in merges) in all panels. Elav is shown in white (magenta when overlaid with GFP in the merges). Yellow lines indicate the positions of virtual cross sections (shown on the right). GFP (panels A–D), Elav (panels A′–D′), merges (panels A″–D″), and virtual cross sections of the merges (panels A″′–D”′). (A) Overexpressing dac in mosaic discs results in small cyst-like clones that disrupt the normal pattern of photoreceptor differentiation. (B) Overexpressing dac in scrib1 mutant clones produces a similar phenotype as scrib1 clones alone (the mutant clone failing to express Elav is located above the disc proper). (C) Overexpressing ab and dac together in clones generates large clones in antenna, similar to ab overexpressing clones alone, but very small clones in the eye disc. (D) Overexpressing dac in scrib1+ab clones does not restrain clone overgrowth, and large neoplastic tumours are produced, which do not express Elav. Yellow scale bar = 50 µm. (JPG) [file pgen.1003627.s017.jpg]

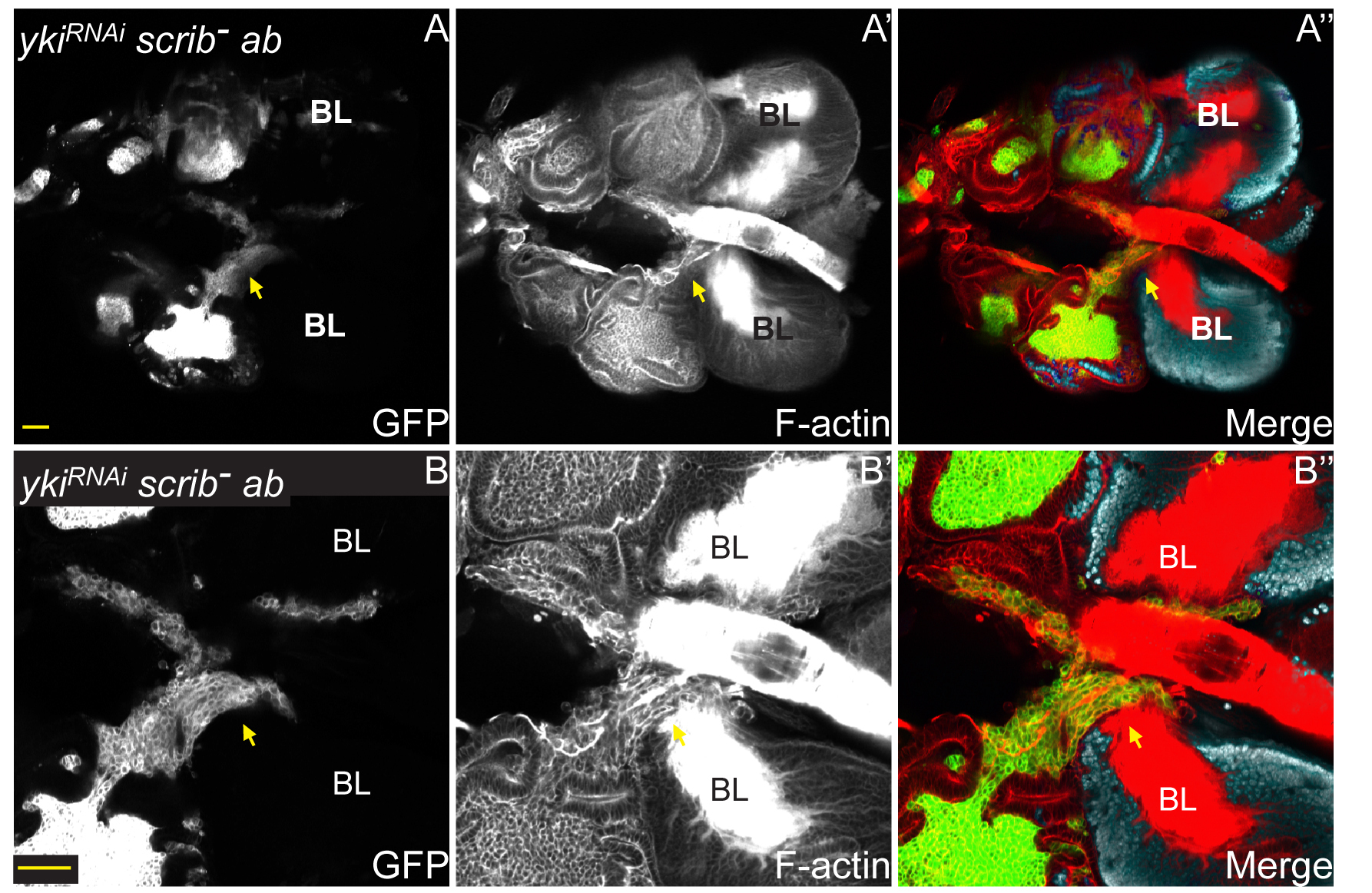

Supplement: Figure S14 — Knockdown of yki in scrib −+ab tumours does not prevent cell migration between the brain lobes. Eye/antennal discs still attached to the brain lobes (BL) containing ey-FLP induced scrib1 clones that express both ab and ykiRNAi (A, and a higher magnification of the region between the brain lobes in B). ey-FLP clones are positively marked by GFP (white, or green in merges) and F-actin is shown in white (red in the merges). Elav is shown in blue in the merges. GFP (panels A,B), F-actin (panels A′,B′), and GFP/F-actin/Elav merges (panels A″,B″). Mutant tissue overgrowth is restrained compared with scrib−+ab tumours, however tissue is still observed between the brain lobes (arrows), consistent with it migrating and merging with the brain lobes. Yellow scale bar = 50 µm. (JPG) [file pgen.1003627.s018.jpg]

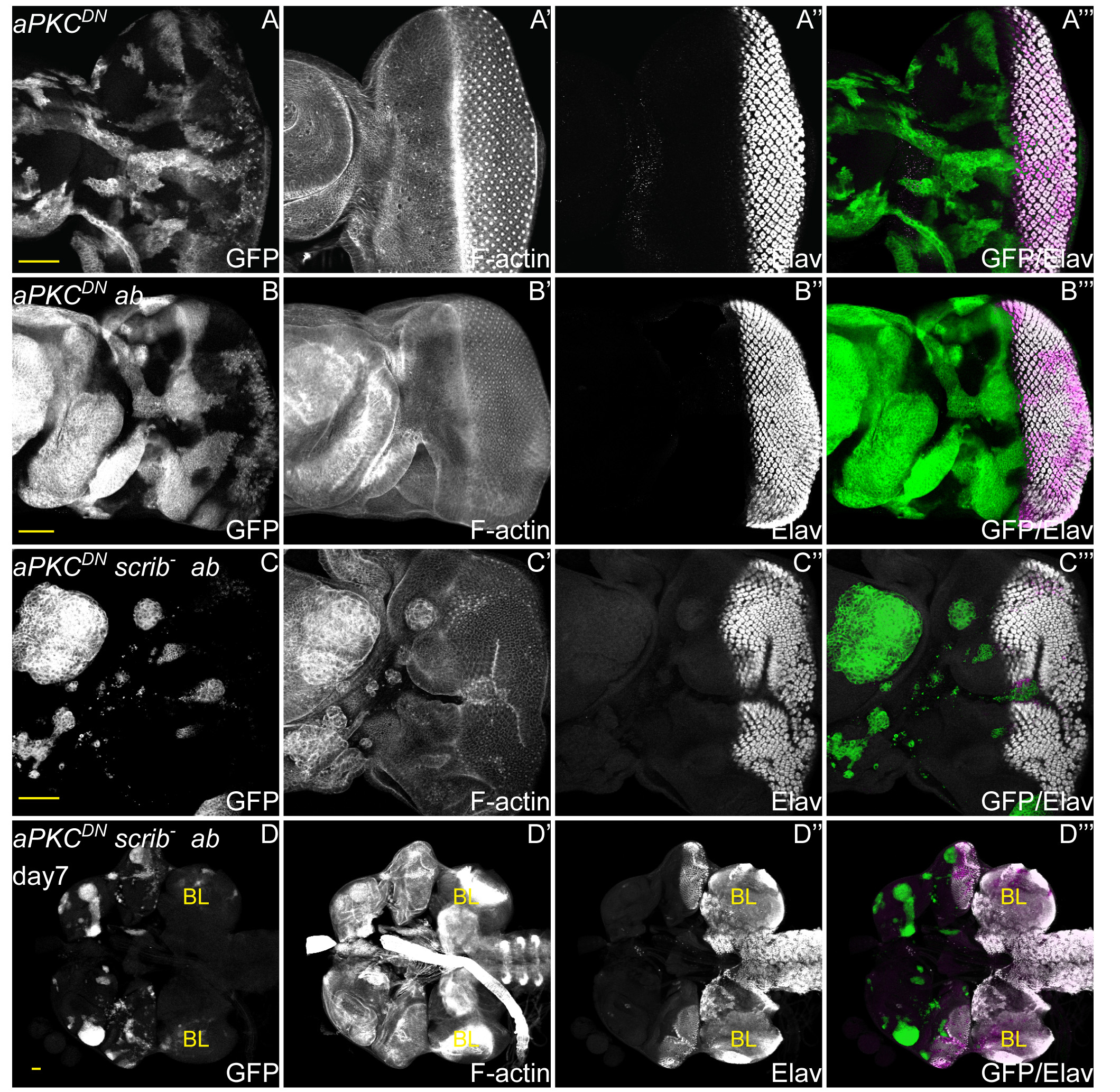

Supplement: Figure S15 — Expression of aPKCDN in scrib −+ab tumours restrains tumour overgrowth. ey-FLP clones at day 5 AEL (A–C), and with brain lobes (BL) attached at day 7 (D). Mutant clones are positively marked by GFP (white, or green in merges). Elav is shown in white (magenta when overlaid with GFP in the merges), and cell morphology is indicated by F-actin (white). GFP (panels A–D), F-actin (panels A′–D′), Elav (panels A″–D″) and GFP/Elav merges (panels A″′–D′″). (A) Overexpressing aPKCCAAX-DN in clones does not produce a discernible phenotype. (B) Clones co-overexpressing aPKCCAAX-DN and ab are similar to ab-expressing clones alone (compare to Figure 1E ). (C,D) Overexpressing aPKCCAAX-DN in scrib1+ab clones restrains clonal tissue overgrowth and GFP specks are observed, consistent with cells undergoing cell death (C). Larvae still enter an extended larval stage of development, however, tumour overgrowth (D) is reduced compared to scrib1+ab tumours (eg. Figure 8F ). Yellow scale bar = 50 µm. (JPG) [file pgen.1003627.s019.jpg]

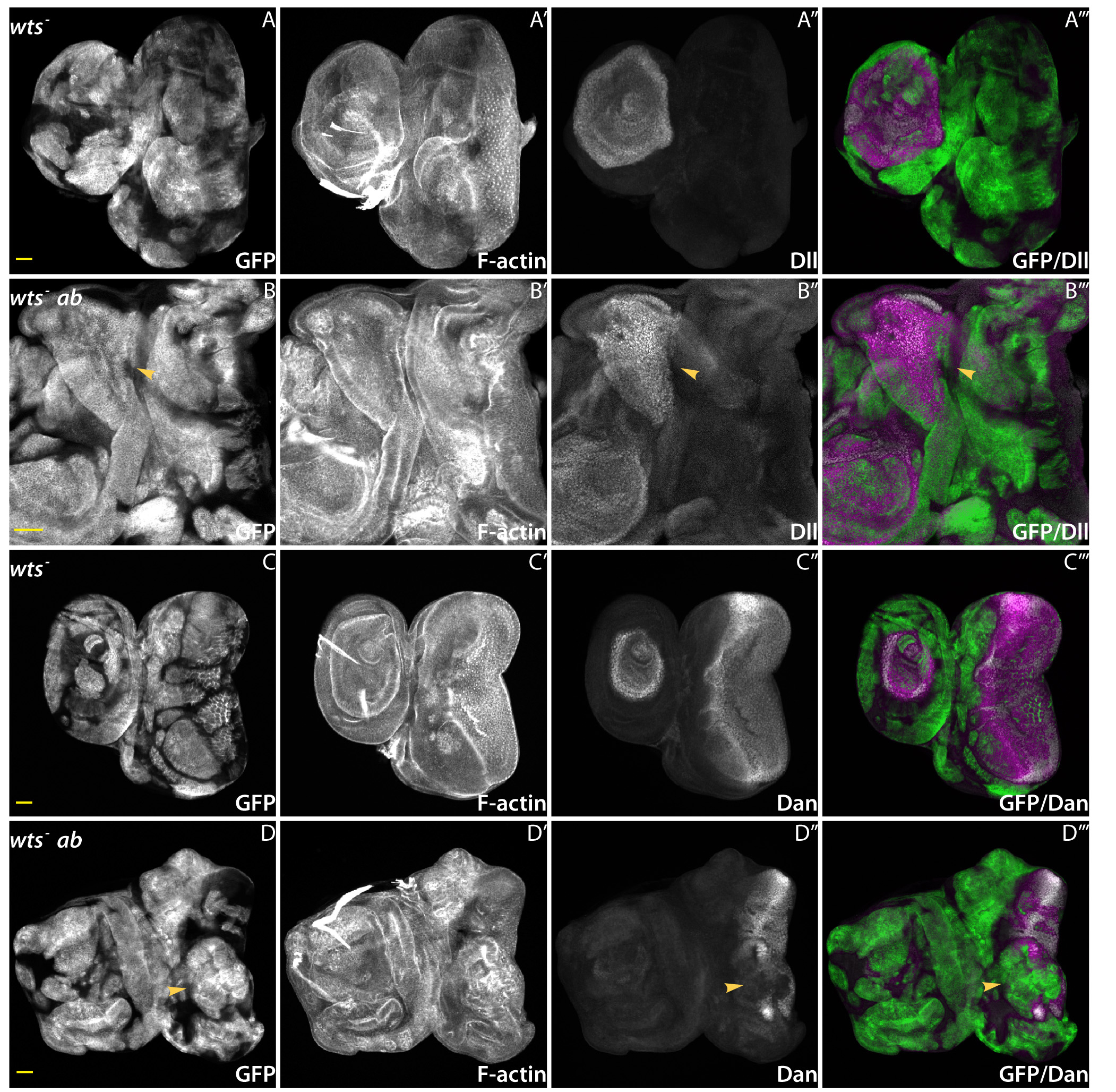

Supplement: Figure S16 — wtsX1+ab tumours retain Dll expression, but do not express Dan. ey-FLP induced eye/antennal disc clones marked by GFP (white, or green in merges). The cell fate markers Dll and Dan are shown in white (magenta when overlaid with GFP in merged images). F-actin (white) shows cell morphology. GFP (panels A–D), F-actin (panels A′–D′), Dll (panels A″,B″), Dan (panels C″,D″), GFP/Dll merges (panels A″′,B″′) and GFP/Dan merges (panels C″′,D′″). (A) wtsX1 clones exhibit the normal pattern of Dll in the eye/antennal disc. (B) wtsX1+ab clones retain Dll expression, often resulting in ectopic domains of Dll-expressing tissue (B, arrowhead), similar to ab expressing clones, or scrib1+ab tumours. (C) wtsX1 clones exhibit the normal pattern of Dan in the eye/antennal disc. (D) wtsX1+ab clones do not express Dan in the antenna, and the overgrowths in the eye disc are also characterised by a loss of Dan (D, arrowhead). Yellow scale bar = 50 µm. (JPG) [file pgen.1003627.s020.jpg]
