# Supplementary material for: The BTB-zinc Finger Transcription Factor Abrupt Acts as an Epithelial Oncogene in Drosophila melanogaster through Maintaining a Progenitor-like Cell State
Source: PLoS Genet. 2013 Jul 18;9(7):e1003627. doi: 10.1371/journal.pgen.1003627 (PMC3715428; doi:10.1371/journal.pgen.1003627)
Supplement: Table S1 — Transcription factor matrices enriched amongst peak sequences associated with the promoter regions or introns of potential target genes. (DOC) [file pgen.1003627.s023.doc]

**Table S1. Transcription factor matrices enriched amongst peak sequences associated with the promoter regions or introns of potential target genes.**

|  | **Ranking** | **TF matrix** | **Transcription Factor** | **Ratio # sites in ChIPSeq *vs* # sites in genome** | **# sites in ChIPSeq** | **# sites in genome** |
| --- | --- | --- | --- | --- | --- | --- |
| ***ab*-specific** | 1 | Trl | Trl | 0.00809237 | 239 | 29534 |
|  | 2 | I$GAGAFACTOR_Q6 | GAGA | 0.00739142 | 250 | 33823 |
|  | 3 | V$MZF1_02 | MZF1 | 0.00737379 | 13 | 1763 |
|  | 4 | SP1 | SP1 | 0.00701754 | 46 | 6555 |
|  | 5 | V$MAZ_Q6 | MAZ | 0.00629664 | 81 | 12864 |
|  | 6 | V$HMX1_01 | HMX1 | 0.00627138 | 11 | 1754 |
|  | 7 | V$AHR_Q5 | AHR | 0.0060111 | 78 | 12976 |
|  | 8 | V$MYCMAX_03 | MYCMAX | 0.00591424 | 12 | 2029 |
|  | 9 | V$AHRHIF_Q6 | AHR | 0.00567677 | 179 | 31532 |
|  | 10 | V$SPZ1_01 | SPZ1 | 0.00561404 | 16 | 2850 |
| **Common** | 1 | V$MZF1_02 | MZF1 | 0.146909 | 259 | 1763 |
|  | 2 | Trl | Trl | 0.146882 | 4338 | 29534 |
|  | 3 | I$GAGAFACTOR_Q6 | GAGA | 0.145315 | 4915 | 33823 |
|  | 4 | V$VDR_Q3 | VDR | 0.135649 | 164 | 1209 |
|  | 5 | PLAG1 | PLAG1 | 0.133929 | 15 | 112 |
|  | 6 | SP1 | SP1 | 0.123722 | 811 | 6555 |
|  | 7 | I$ADF1_Q6_01 | ADF1 | 0.117825 | 39 | 331 |
|  | 8 | V$MAZ_Q6 | MAZ | 0.114195 | 1469 | 12864 |
|  | 9 | CTCF | CTCF | 0.107143 | 12 | 112 |
|  | 10 | V$LDSPOLYA_B | LDSPOLYA | 0.105173 | 246 | 2339 |
| ***scrib*-specific** | 1 | V$IRF2_01 | IRF2 | 0.00367647 | 12 | 3264 |
|  | 2 | V$ATF_B | ATF | 0.00325733 | 11 | 3377 |
|  | 3 | CREB1 | CREB1 | 0.00311526 | 31 | 9951 |
|  | 4 | V$CREB_01 | CREB | 0.00298238 | 43 | 14418 |
|  | 5 | V$CREL_01 | CREL | 0.00294118 | 29 | 9860 |
|  | 6 | V$NFKAPPAB65_01 | NFKAPPAB6 | 0.00293794 | 16 | 5446 |
|  | 7 | V$CREB_Q4 | CREB | 0.002886 | 12 | 4158 |
|  | 8 | V$CREB_Q4_01 | CREB | 0.0028643 | 16 | 5586 |
|  | 9 | N$CES2_01 | CES2 | 0.00282965 | 15 | 5301 |
|  | 10 | REL | REL | 0.00281096 | 24 | 8538 |
|  | 11 | RELA | RELA | 0.00280617 | 14 | 4989 |
|  | 12 | gt | Gt | 0.00280153 | 19 | 6782 |
|  | 13 | V$TFE_Q6 | TFE | 0.00278293 | 54 | 19404 |
|  | 14 | V$AHR_Q5 | AHR | 0.00269729 | 35 | 12976 |
|  | 15 | V$WHN_B | WHN | 0.00257645 | 23 | 8927 |
|  | **16** | **V$AP1_C** | **AP1** | **0.002457** | **36** | **14652** |
|  | 17 | RUNX1 | RUNX1 | 0.00244044 | 21 | 8605 |
|  | 18 | V$CHX10_01 | CHX10 | 0.00237624 | 12 | 5050 |
|  | 19 | V$FAC1_01 | FAC1 | 0.00233372 | 36 | 15426 |
|  | 20 | V$CREBATF_Q6 | CREBATF | 0.00233294 | 28 | 12002 |
